# Supplementary material for: Comparative transcriptomics of social insect queen pheromones
Source: Nat Commun. 2019 Apr 8;10:1593. doi: 10.1038/s41467-019-09567-2 (PMC6453924; doi:10.1038/s41467-019-09567-2)
Supplement: Supplementary file 1 — Supplementary Information [file 41467_2019_9567_MOESM1_ESM.pdf]

Supplementary Information

Comparative transcriptomics of social insect queen pheromones

Holman et al.

Supplementary Figures and Supplementary Tables

All of the figures and tables in this document can also be viewed online at **https://lukeholman.github.io/queen-pheromone-RNAseq/statistical-analysis.html**, along with the R code used to generate them. The long tables are easier to navigate in the online version.

Supplementary figures

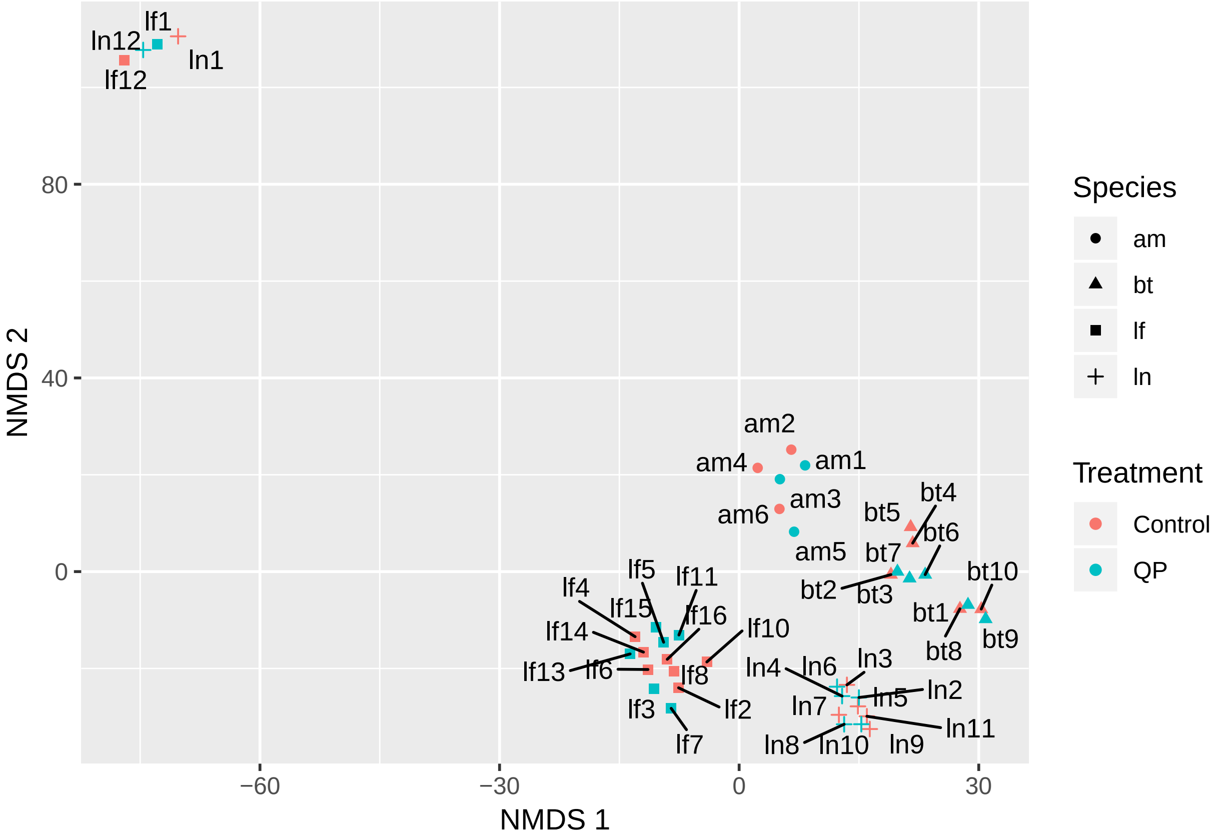

Supplementary Figure 1: After reducing the transcriptome data to two axes using non-metric multidimensional scaling, four *Lasius* samples were clear outliers.

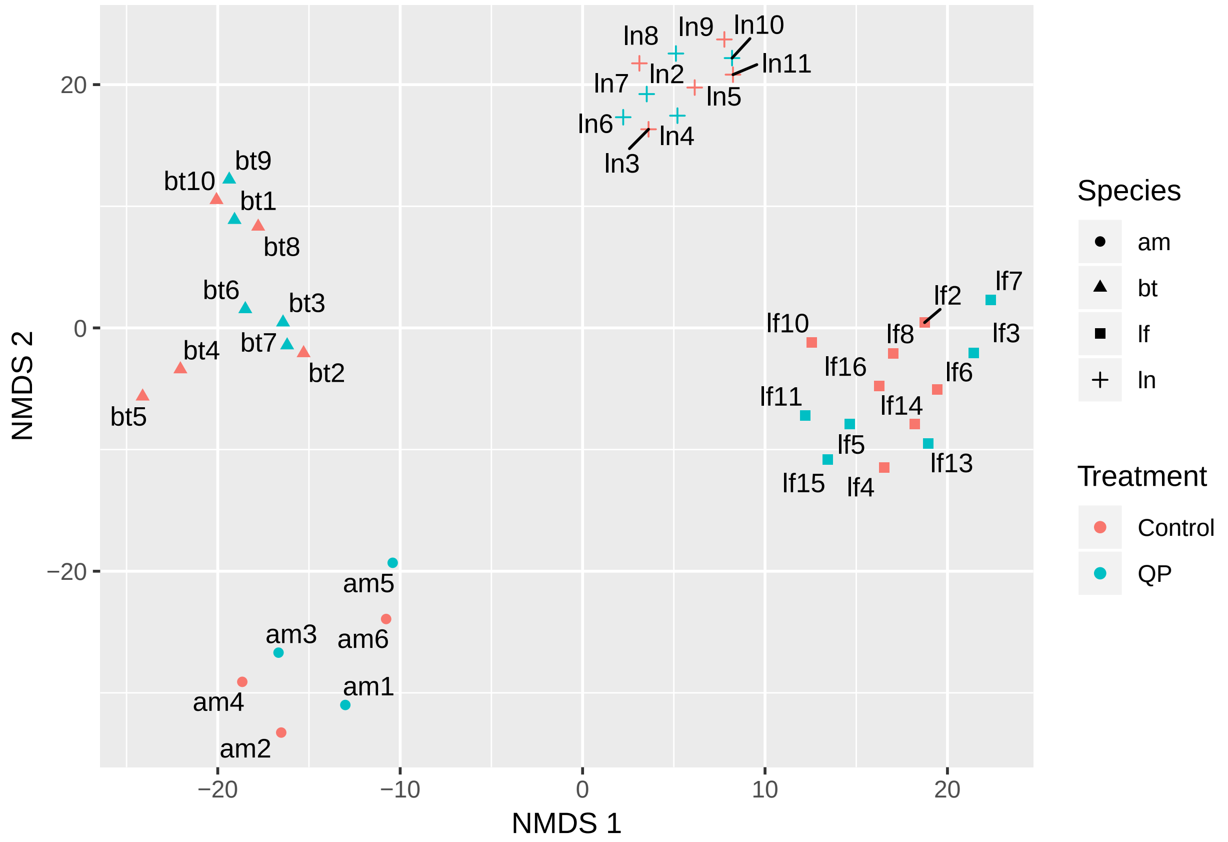

Supplementary Figure 2: With the four problematic samples removed, the samples cluster according to species with no obvious outliers.

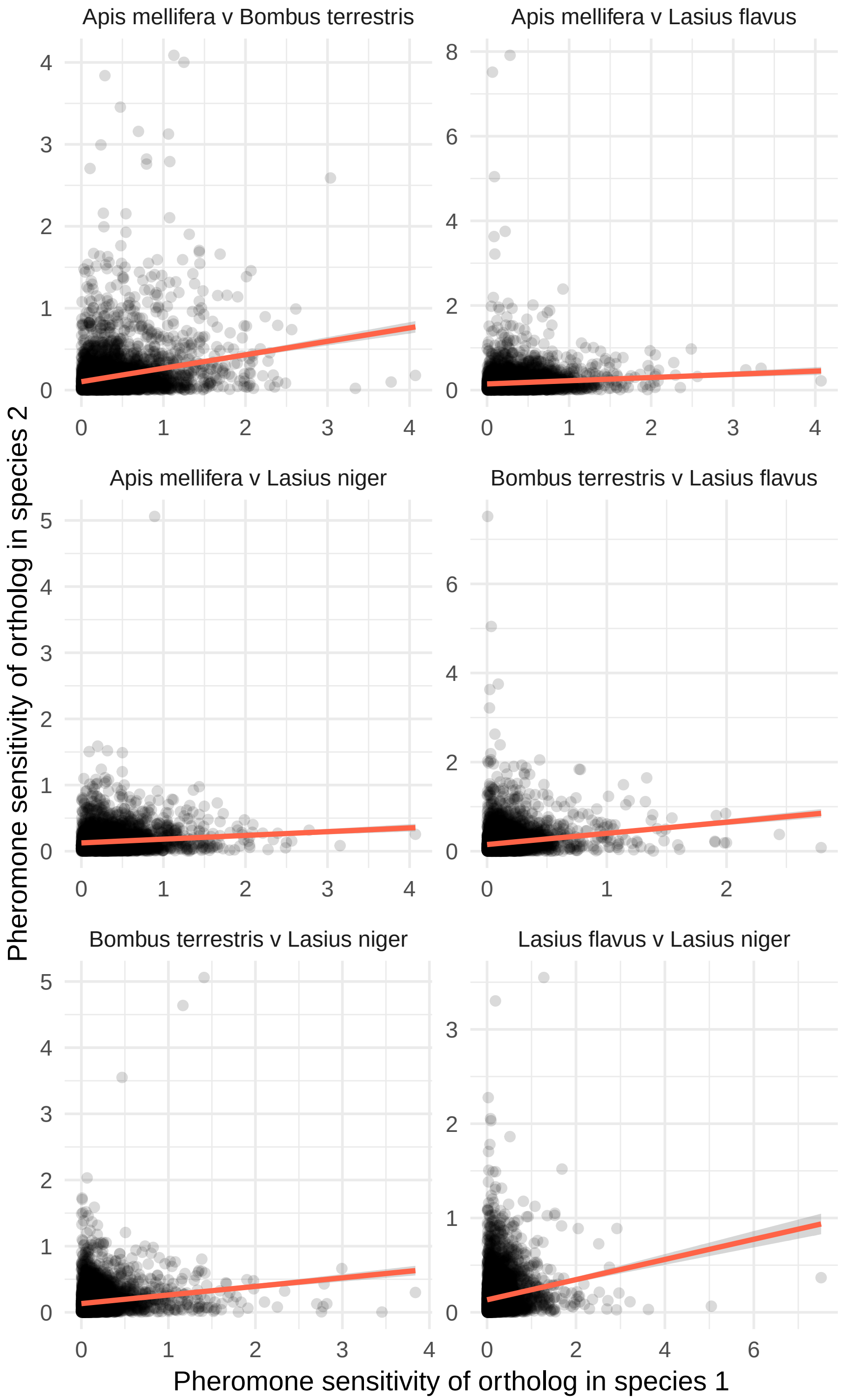

Supplementary Figure 3: Each scatterplot shows the correlation in pheromone sensitivity across pairs of orthologous genes, for each of the six possible species pairs. Species 1 refers to the first-listed species, such that in the top-left panel, *Apis mellifera* is plotted on the x-axis and *Bombus terrestris* is on the y-axis. The regression lines are from a simple linear regression, and the grey zone around the line shows its 95% confidence intervals.

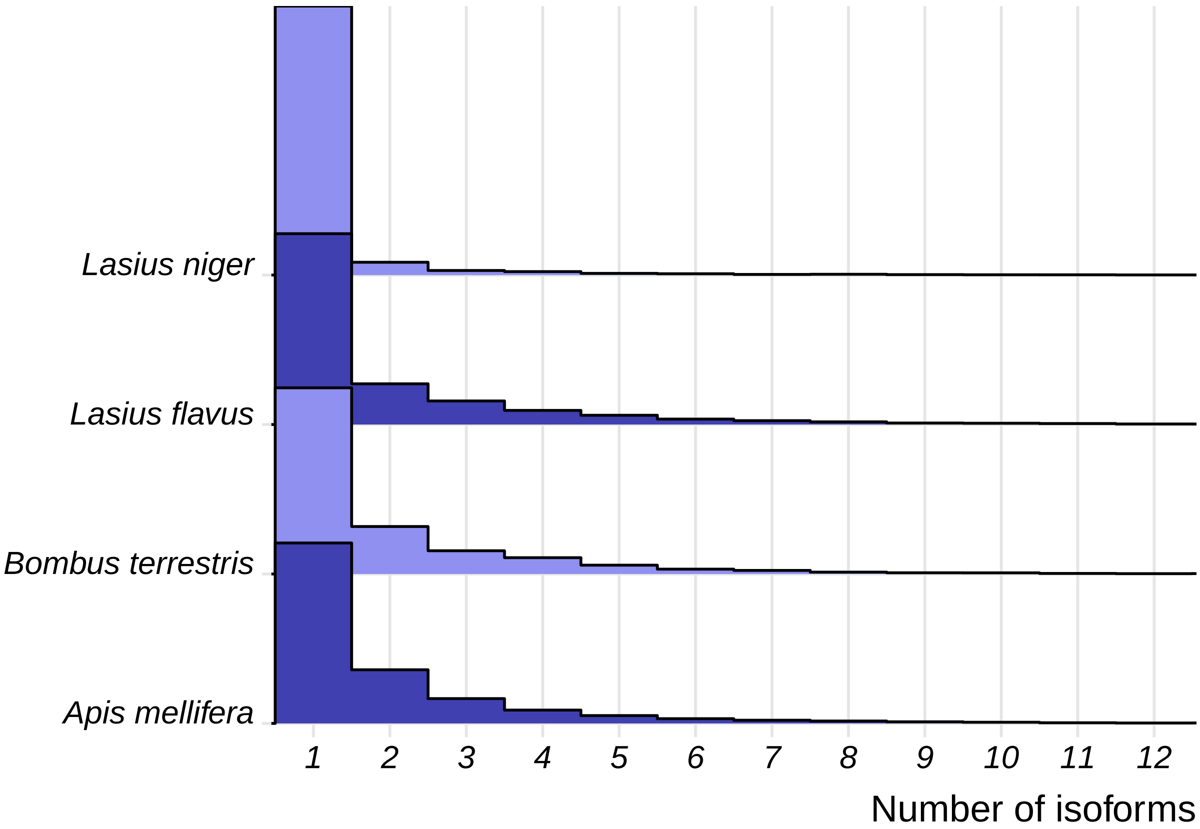

Supplementary Figure 4: Distribution of isoform numbers per gene for each of the four species.

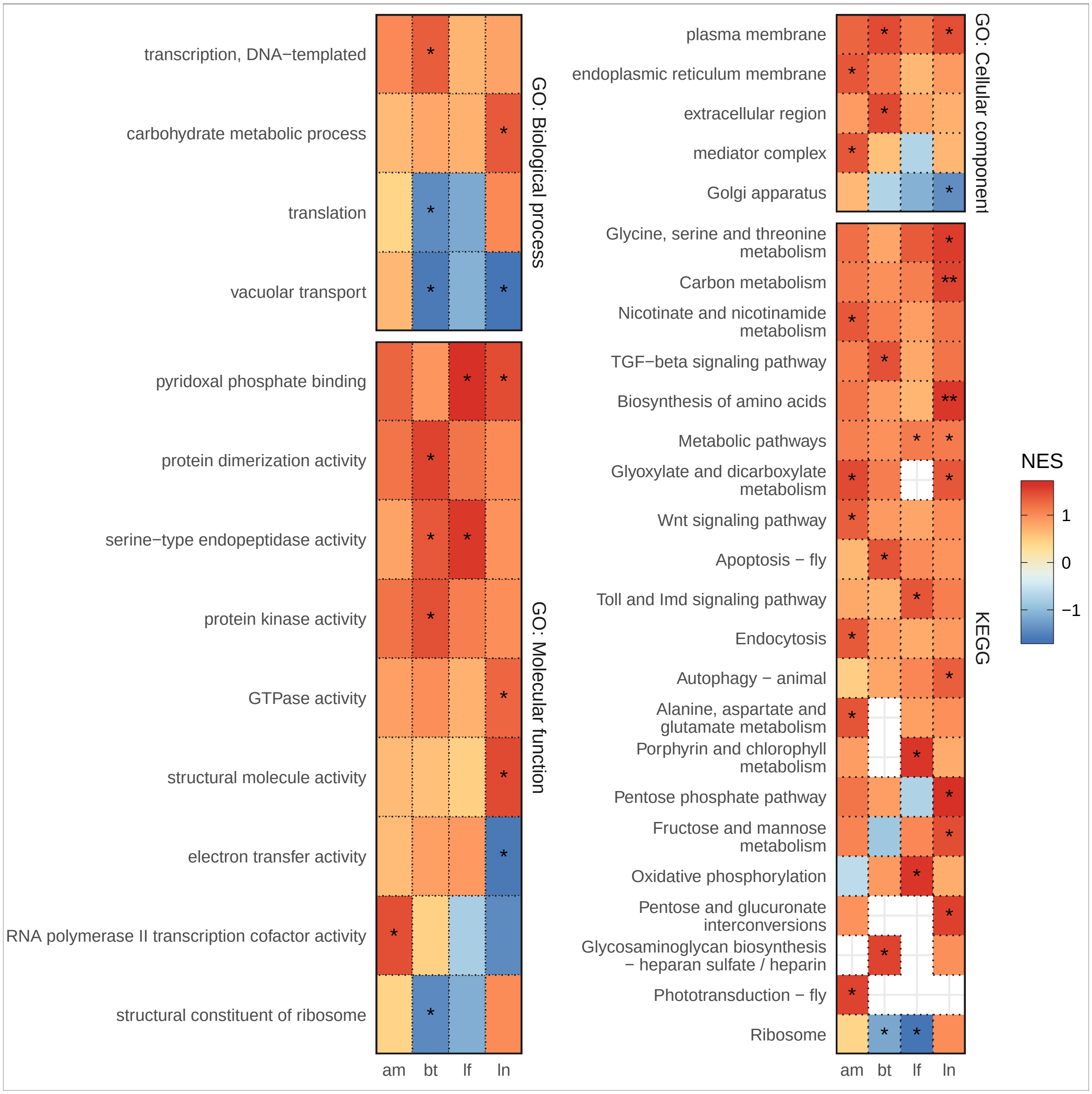

Supplementary Figure 5: Genes for which alternative splicing is strongly affected by queen pheromone tend to have similar Gene Ontology and KEGG terms in ants and bees, although the data do not provide strong evidence for or against inter-species similarity. The colour shows the normalised expression score from a GSEA (gene set enrichment analysis) test implemented in the R package `fgsea`; positive (red) values indicate that the GO or KEGG term is over-represented among genes whose splicing is strongly affected by queen pheromone, and negative (blue) values indicate under-representation among those genes. Asterisks denote statistically significant enrichment ($p < 0.05$), and double asterisks mark results that remained significant after adjusting the p-values for multiple testing using the Benjamini-Hochberg method. Empty squares denote cases where we did not find at least 5 alternatively spliced genes annotated with the focal term.

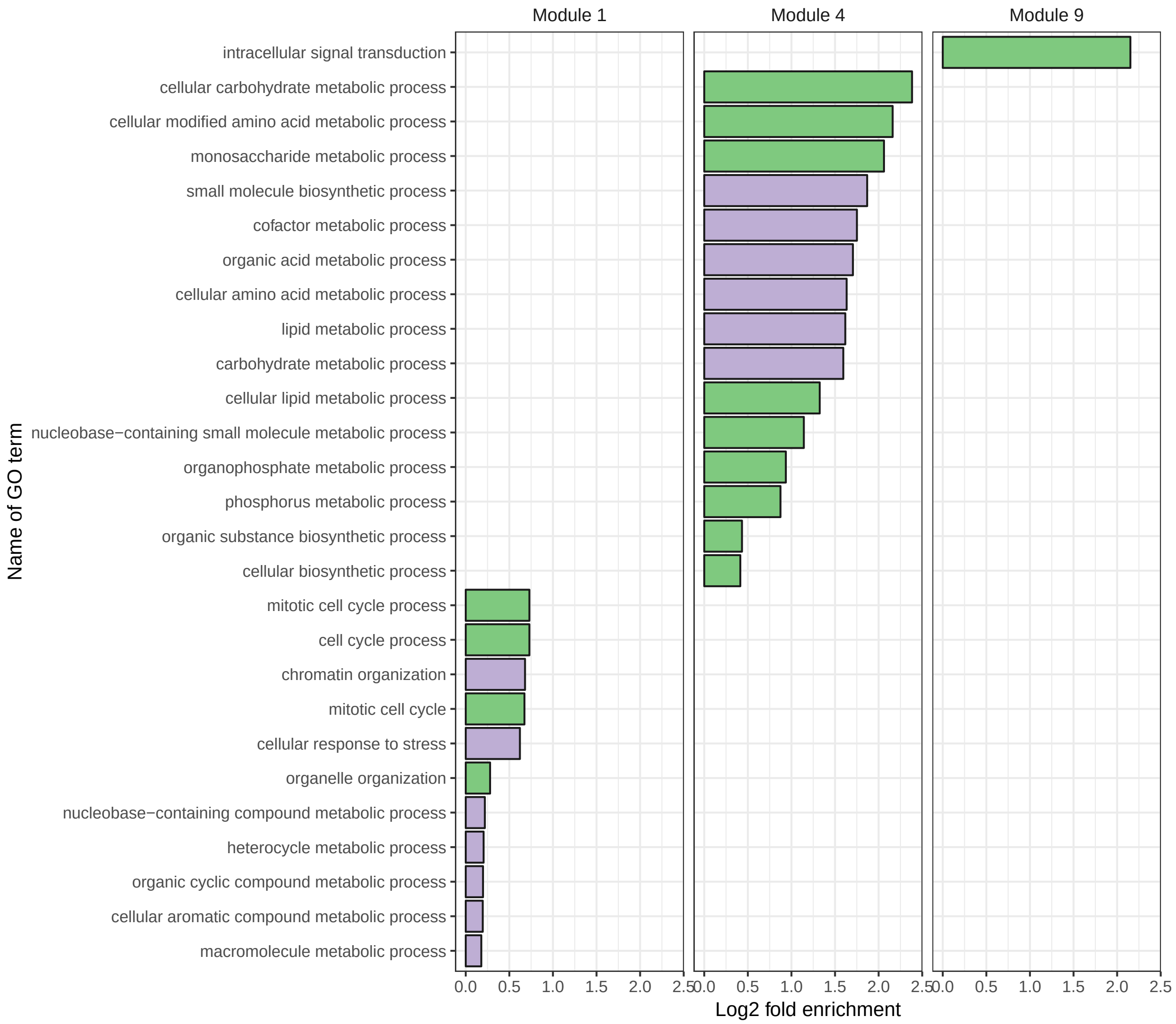

Supplementary Figure 6: Comparable figure to Figure 4, showing the results of GO: Biological process enrichment analysis instead of KEGG pathways.

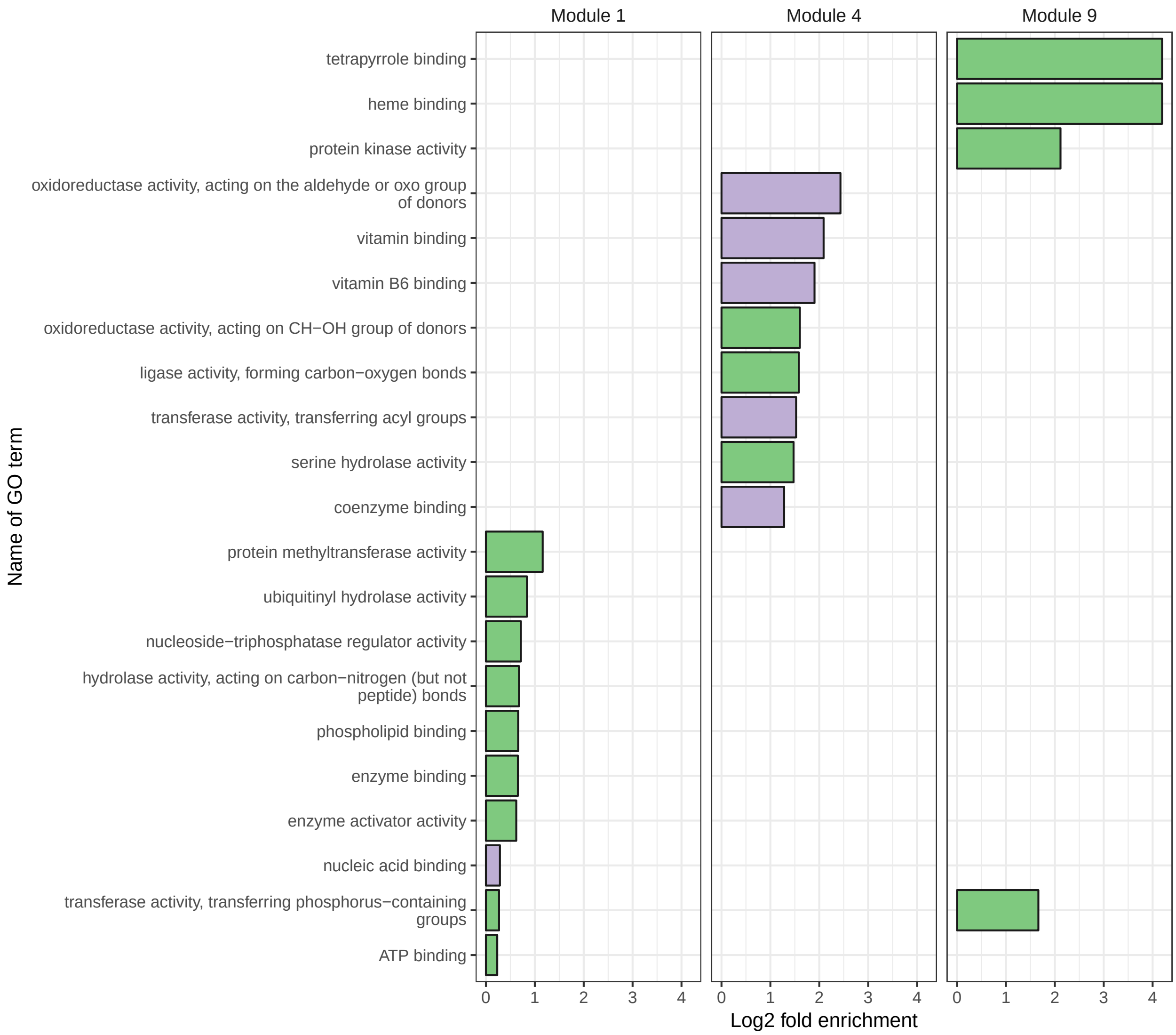

Supplementary Figure 7: Comparable figure to Figure 4, showing the results of GO: Molecular function enrichment analysis instead of KEGG pathways.

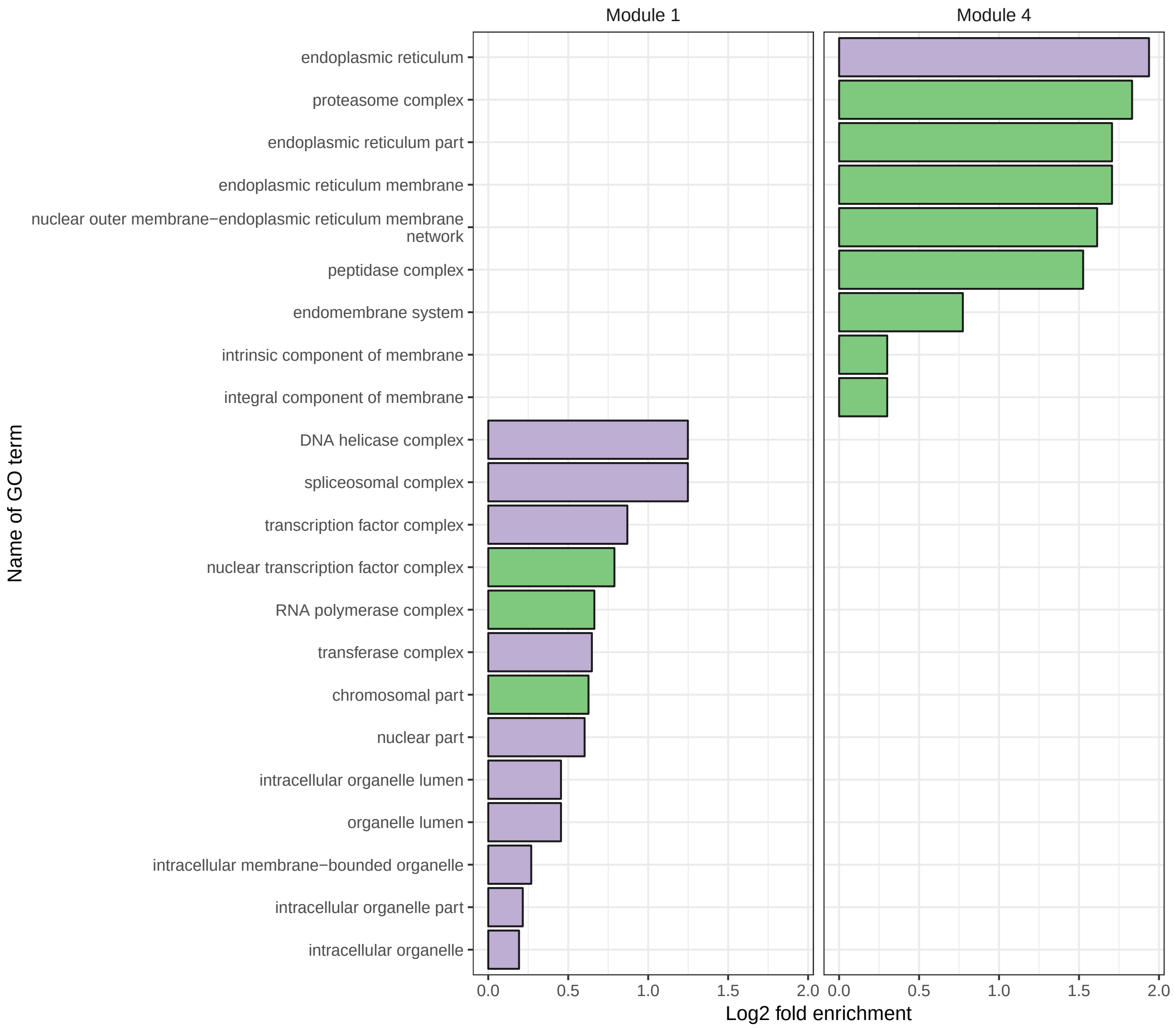

Supplementary Figure 8: Comparable figure to Figure 4, showing the results of GO: Cellular component enrichment analysis instead of KEGG pathways. Module 9 is missing because no GO:CC terms were significantly enriched.

Supplementary tables

Supplementary Table 1: Number of sequencing libraries for each combination of species and treatment, after removing the four problematic libraries. Each library was prepared from a pool containing equal amounts of cDNA from five individual workers, taken from the same colony.

Species	Treatment	Number of RNAseq libraries
am	Control	3
am	QP	3
bt	Control	5
bt	QP	5
lf	Control	7
lf	QP	6
ln	Control	5
ln	QP	5

Supplementary Table 2: List of the 322 significantly differentially expressed genes (EBseq; FDR-corrected posterior probability of differential expression $p < 0.05$) in *Apis mellifera*, listed in order of fold change in gene expression on a Log_2 scale. Positive fold change values indicate higher expression in the control, while negative values indicate higher expression in the queen pheromone treatment.

Gene	Name	Log2 FC
GB55204	Major royal jelly protein 3	6.480
GB51373	bypass of stop codon protein 1-like	5.385
GB50604	uncharacterized protein LOC724113	3.775
102655911	uncharacterized protein LOC102655911	-3.376
GB49819	branched-chain-amino-acid aminotransferase, cytosolic-like	2.657
102656917	uncharacterized LOC102656917, transcript variant X1	2.609
GB54417	dehydrogenase/reductase SDR family member 11-like isoform X1	2.242
GB45565	chymotrypsin-2	2.052
GB53886	protein G12-like isoform X4	1.983
100576536	uncharacterized protein LOC100576536	1.945
GB41540	venom carboxylesterase-6-like	-1.721
GB54690	uncharacterized protein LOC408547	-1.583
GB54150	uncharacterized protein LOC408462	-1.560
GB43639	uncharacterized protein LOC100577506 isoform X1	-1.557
GB49548	serine/threonine-protein phosphatase 2B catalytic subunit 3-like isoform X11	-1.455
GB49878	probable cytochrome P450 6a14 isoformX1	1.393
GB53414	serine/threonine-protein kinase ICK-like isoform X2	1.357
102654781	protein G12-like	1.324
102656058	uncharacterized protein PF11_0213-like	-1.311
GB53957	U6 snRNA-associated Sm-like protein LSm1-like	1.276
GB50413	protein TBRG4-like isoform X1	-1.261
102653931	uncharacterized LOC102653931, transcript variant X2	-1.258
GB53876	interaptin-like	-1.209
GB42705	protein archease-like	1.184
101664701	PI-PLC X domain-containing protein 1-like isoform X1	1.143
GB42523	uncharacterized LOC100577781, transcript variant X2	-1.130
102654405	protein G12-like	1.115
100578075	uncharacterized LOC100578075	-1.110
GB52251	multifunctional protein ADE2, transcript variant X2	1.088
GB40764	uncharacterized protein LOC414021 isoform X7	-1.086
GB55648	Down syndrome cell adhesion molecule-like protein Dscam2-like isoform X7	-1.084
726446	uncharacterized protein LOC726446	-1.063
GB54467	probable G-protein coupled receptor 52 isoform 1	-1.060
GB46985	60S ribosomal protein L12 isoform X1	1.045
GB55191	uncharacterized protein LOC100576289	-1.045
GB54890	kynurenine 3-monooxygenase isoform X2	1.016
GB55640	retinol dehydrogenase 12-like	-1.006
724802	protein Asterix-like	0.996
GB40010	titin-like isoform X2	-0.967
102654949	uncharacterized protein LOC102654949	0.967
GB43234	histone deacetylase 5 isoform X8	-0.965
GB52266	furin-like protease 2-like	-0.927
GB41706	ice-structuring glycoprotein-like	-0.926
GB42673	retinol dehydrogenase 10-A-like isoform X4	0.923
GB55030	uncharacterized protein LOC725074	0.921
551123	RNA-binding protein Musashi homolog Rbp6-like isoform X1	-0.920
409728	40S ribosomal protein S5 isoform X1	0.917
GB45028	venom dipeptidyl peptidase 4	-0.898
GB53422	ufm1-specific protease 1-like isoform X2	0.892
GB48933	methenyltetrahydrofolate synthase domain-containing protein-like	-0.876
GB53077	cysteine-rich protein 1-like	0.871
GB41301	annexin-B9-like	0.862
GB51748	dentin sialophosphoprotein	-0.861
102654594	WD repeat-containing protein 18-like	0.852
GB50356	60S acidic ribosomal protein P2	0.837
GB44091	LOW QUALITY PROTEIN: uncharacterized protein LOC408779	-0.836
GB41151	protein MNN4-like	-0.834
409202	ribosomal protein S9, transcript variant X2	0.834

(continued)

Gene	Name	Log2 FC
GB44340	small ubiquitin-related modifier 3 isoform 1	0.833
GB49173	4-aminobutyrate aminotransferase, mitochondrial-like isoform X2	-0.826
GB40769	dehydrogenase/reductase SDR family member 11-like	0.823
GB54243	LOW QUALITY PROTEIN: carbonyl reductase [NADPH] 1-like	0.819
724531	40S ribosomal protein S28-like	0.812
GB51744	uncharacterized protein LOC724439	0.811
GB51947	uncharacterized protein LOC724835 isoform X2	-0.807
GB40875	60S ribosomal protein L10 isoform X1	0.806
102655694	glutathione S-transferase-like	0.803
GB55827	40S ribosomal protein S21-like isoform X1	0.801
102654426	60S ribosomal protein L18-like	0.801
GB49988	SRR1-like protein-like isoform X2	-0.800
GB55963	uncharacterized protein LOC725224 isoform X1	-0.797
GB50867	cell differentiation protein RCD1 homolog isoform X2	0.794
GB43256	ATP-binding cassette sub-family D member 1-like	0.792
GB41211	ATP-binding cassette sub-family E member 1	0.787
GB52314	gamma-tubulin complex component 4	-0.785
102654251	uncharacterized protein LOC102654251	-0.782
726860	cytochrome b5-like isoform 1	0.778
GB46039	tubulin alpha-1 chain-like	0.773
GB53358	protein transport protein Sec61 subunit gamma-like isoform X3	0.768
GB48699	60S ribosomal protein L11-like	0.767
GB44311	actin related protein 1	0.762
102655603	transmembrane emp24 domain-containing protein 7-like	0.762
GB49170	40S ribosomal protein S15Aa-like isoform 1	0.758
GB47736	alkyldihydroxyacetonephosphate synthase-like	0.753
GB49013	RNA-binding protein 8A	0.752
724485	probable small nuclear ribonucleoprotein E-like	0.749
GB53000	ubiquitin-60S ribosomal protein L40 isoform 2	0.749
725936	titin-like	-0.748
GB50158	60S ribosomal protein L4 isoform 1	0.748
GB52432	KN motif and ankyrin repeat domain-containing protein 3-like isoform X3	-0.746
724757	histone H4-like	0.745
GB53219	40S ribosomal protein S17	0.742
100577623	putative uncharacterized protein DDB_G0282133-like isoform X2	-0.742
GB51038	60S ribosomal protein L23	0.739
GB40284	cytochrome P450 6a2	0.737
GB50709	40S ribosomal protein S19a	0.735
GB50977	probable tubulin polyglutamylase TTLL2-like	-0.734
GB42537	40S ribosomal protein S15	0.734
GB42467	phospholipase B1, membrane-associated-like isoform X2	0.733
GB41886	protein transport protein Sec61 subunit alpha isoform 2	0.729
GB51201	40S ribosomal protein S12 isoform X1	0.724
GB55183	ankyrin repeat domain-containing protein SOWAHB-like isoform X5	-0.724
GB54814	60S ribosomal protein L31 isoform 1	0.721
GB49159	probable nuclear transport factor 2-like isoform 3	0.717
GB52512	60S ribosomal protein L28	0.714
GB41142	probable dolichyl pyrophosphate Glc1Man9GlcNAc2 alpha-1,3-glucosyltransferase-like isoform X2	0.712
GB51359	60S ribosomal protein L27a isoform X1	0.710
GB50519	transmembrane emp24 domain-containing protein eca-like	0.710
GB47638	ER membrane protein complex subunit 3-like	0.705
GB51046	probable signal peptidase complex subunit 2-like	0.701
GB54973	selT-like protein-like isoform 1	0.693
GB44661	intracellular protein transport protein USO1 isoform X9	-0.691
GB53953	mitochondrial coenzyme A transporter SLC25A42-like isoformX1	-0.688
GB48289	uncharacterized protein LOC726292 isoform X1	0.688
GB47808	DEP domain-containing protein 5 isoform X4	-0.685
GB45937	intracellular protein transport protein USO1 isoform X2	-0.684
GB53750	UPF0454 protein C12orf49 homolog isoform X2	0.682
GB50455	ubiquitin-conjugating enzyme E2-17 kDa-like	0.676
GB42356	arginine-glutamic acid dipeptide repeats protein-like	-0.675
GB51072	40S ribosomal protein S4-like isoform 1	0.669

(continued)

Gene	Name	Log2 FC
GB55268	43 kDa receptor-associated protein of the synapse homolog isoform X3	-0.669
GB43086	uncharacterized protein LOC726486	0.668
102655259	5-methylcytosine rRNA methyltransferase NSUN4-like isoform X1	-0.667
GB55639	40S ribosomal protein S3	0.666
GB41159	bifunctional dihydrofolate reductase-thymidylate synthase	0.665
GB42354	ATP-dependent Clp protease ATP-binding subunit clpX-like, mitochondrial-like isoform X4	-0.663
GB42696	60S ribosomal protein L35a isoform X3	0.656
GB42088	40S ribosomal protein S29-like isoform X2	0.652
GB53948	uncharacterized protein LOC410057 isoform X1	-0.651
GB47553	electron transfer flavoprotein subunit alpha, mitochondrial-like isoform 1	0.650
100577163	slit homolog 2 protein-like	0.650
GB52627	protein pigeon-like	-0.650
GB54020	apolipoprotein D-like	0.650
102655440	uncharacterized protein LOC102655440	-0.649
GB51009	T-complex protein 1 subunit delta-like isoform 1	0.649
GB49583	40S ribosomal protein S14	0.647
GB41039	60S ribosomal protein L17 isoform 1	0.647
GB46627	paraplegin-like	-0.645
GB54174	E3 ubiquitin-protein ligase RING1 isoform 1	0.643
GB41240	aquaporin AQPAn.G-like isoform X3	-0.641
GB51440	proteoglycan 4-like	-0.641
GB45433	small ribonucleoprotein particle protein B	0.638
GB51603	peptidyl-alpha-hydroxyglycine alpha-amidating lyase 1-like isoform X5	-0.638
100191002	ribosomal protein L41	0.638
GB43989	serine-threonine kinase receptor-associated protein-like	0.637
GB53799	proteasome subunit alpha type-2	0.636
GB43141	uncharacterized protein LOC413428	-0.636
GB44999	chascon-like	-0.633
GB49154	bcl-2-related ovarian killer protein homolog A	0.632
GB50189	epsilon-sarcoglycan	-0.630
GB41150	40S ribosomal protein S2 isoform 2	0.628
GB50917	60S acidic ribosomal protein P1	0.627
GB48201	39S ribosomal protein L53, mitochondrial	0.627
GB44575	ankyrin repeat and zinc finger domain-containing protein 1-like isoform X1	-0.626
GB46776	40S ribosomal protein S11 isoform X1	0.624
GB49789	28S ribosomal protein S29, mitochondrial isoformX1	-0.621
GB46750	40S ribosomal protein S16	0.620
GB44749	60S ribosomal protein L9	0.618
GB44931	evolutionarily conserved signaling intermediate in Toll pathway, mitochondrial-like	-0.613
GB46845	60S ribosomal protein L37a	0.611
GB43379	membrane-bound transcription factor site-2 protease-like	0.609
GB45369	receptor of activated protein kinase C 1, transcript variant X3	0.606
GB52698	synaptobrevin-like isoformX1	0.605
724829	immediate early response 3-interacting protein 1-like isoform X1	0.605
GB49536	gamma-secretase subunit Aph-1	0.604
GB55628	probable RNA-binding protein EIF1AD-like isoform X1	0.603
GB50832	THO complex subunit 4-like	0.602
GB50929	mitochondrial import receptor subunit TOM40 homolog 1-like isoform 1	0.601
GB51065	40S ribosomal protein S10-like isoform 1	0.601
GB54984	chromatin complexes subunit BAP18-like isoform X1	0.600
GB43180	minor histocompatibility antigen H13-like	0.597
GB49365	gamma-secretase subunit pen-2 isoform 1	0.595
GB51543	60S ribosomal protein L13a isoform 2	0.594
GB54341	RNA-binding protein 33-like	-0.594
102655912	L-aminoadipate-semialdehyde dehydrogenase-phosphopantetheinyl transferase-like	0.593
GB53420	uncharacterized protein LOC100576355 isoformX2	-0.591
GB48370	ATP-binding cassette sub-family B member 7, mitochondrial isoform X1	-0.591
726369	peptidyl-tRNA hydrolase 2, mitochondrial-like isoform 1	-0.591
GB46478	tectonin beta-propeller repeat-containing protein isoform X1	-0.591
GB42736	TM2 domain-containing protein CG10795-like	0.588
GB49087	formin-binding protein 1 homolog isoform X7	-0.588

(continued)

Gene	Name	Log2 FC
GB50753	uncharacterized LOC408705	-0.586
GB46123	endonuclease G, mitochondrial-like	-0.586
GB48574	thioredoxin-2 isoform 1	0.586
GB43232	transmembrane protein 222-like isoform 1	0.586
GB45285	eukaryotic translation initiation factor 3 subunit F-like	0.584
GB44631	uroporphyrinogen-III synthase-like	0.582
GB49994	60S ribosomal protein L26	0.581
GB52563	ATP-dependent helicase brm	-0.579
GB54723	uncharacterized protein LOC726790 isoform X1	-0.578
GB53668	translocator protein-like	0.577
GB45374	40S ribosomal protein S23-like	0.576
GB46984	ribonuclease UK114-like isoform 1	0.572
102655352	uncharacterized protein LOC102655352	-0.572
GB45037	beta-lactamase-like protein 2-like isoform X2	-0.572
GB52107	tubulin alpha-1 chain-like	0.571
GB54139	flocculation protein FLO11-like	-0.565
410017	protein OPI10 homolog	0.564
GB49177	60S ribosomal protein L27 isoform X2	0.557
GB54221	transmembrane protein 50A-like	0.556
GB54979	60S ribosomal protein L21	0.555
GB48111	proteasome subunit beta type-1	0.552
GB48745	5'-nucleotidase domain-containing protein 3-like	-0.550
GB47079	hexokinase type 2-like isoform X3	-0.549
GB47441	V-type proton ATPase 21 kDa proteolipid subunit-like	0.549
GB41207	26S proteasome non-ATPase regulatory subunit 14	0.549
GB50274	transitional endoplasmic reticulum ATPase TER94	0.544
GB51683	annexin-B9-like isoform X1	0.544
GB54952	proteasome subunit alpha type-1-like	0.543
GB52253	protein PRRC2C-like isoform X2	-0.542
GB41648	protein chibby homolog 1-like	0.542
GB41363	26S protease regulatory subunit 6B isoform 1	0.541
GB53247	transmembrane emp24 domain-containing protein-like	0.541
GB48983	RING finger protein 121-like isoform X3	0.541
GB50873	60S ribosomal protein L30 isoform 1	0.540
GB54255	uncharacterized protein LOC551488	0.540
GB48810	60S ribosomal protein L8	0.537
GB41894	uncharacterized protein LOC411277 isoform X28	-0.536
GB49021	cuticular protein precursor	0.536
GB50131	phosphatidate phosphatase PPAPDC1A-like isoform X2	0.535
GB41811	filaggrin-like isoform X3	-0.534
GB51484	protein mago nashi	0.528
GB46705	muscle M-line assembly protein unc-89 isoform X5	-0.526
GB45978	dynein light chain Tctex-type isoform X2	0.526
GB43449	signal recognition particle 9 kDa protein	0.526
GB48150	actin-related protein 2/3 complex subunit 1A	0.525
GB54854	proteasome maturation protein-like	0.523
GB51545	dystrophin, isoforms A/C/F/G/H-like	-0.523
GB49095	high affinity copper uptake protein 1-like isoformX1	0.523
GB43638	protein enhancer of sevenless 2B	0.522
GB51994	proteasome subunit beta type-6-like	0.520
GB53194	60S ribosomal protein L14 isoform X2	0.519
102656618	uncharacterized protein LOC102656618 isoform X1	-0.518
GB40539	40S ribosomal protein S20	0.518
GB41631	60S ribosomal protein L34 isoform X2	0.518
GB43938	cytosolic endo-beta-N-acetylglucosaminidase-like isoform X4	0.516
GB45878	tRNA-dihydrouridine(16/17) synthase [NAD(P)(+)]-like isoform X3	-0.516
GB44039	malate dehydrogenase, cytoplasmic-like isoform 1	0.513
GB55781	LOW QUALITY PROTEIN: uncharacterized protein LOC551170	-0.513
GB45526	eukaryotic translation initiation factor 6 isoform 1	0.510
GB52789	60S ribosomal protein L22 isoform 1	0.507
GB53626	myotrophin-like isoform 2	0.507
GB49364	splicing factor U2af 38 kDa subunit	0.505

(continued)

Gene	Name	Log2 FC
GB44984	U5 small nuclear ribonucleoprotein 40 kDa protein-like isoform X1	0.504
GB50271	zinc transporter 1-like	0.503
GB49377	40S ribosomal protein S3a	0.501
GB50874	transcription factor Ken 2	-0.501
GB44147	60S ribosomal protein L15	0.498
GB46141	LOW QUALITY PROTEIN: vacuolar protein sorting-associated protein 29-like	0.494
GB51963	mitochondrial ribonuclease P protein 1 homolog	-0.491
GB55901	ribosome biogenesis protein NSA2 homolog isoform X1	0.488
GB42036	protein SEC13 homolog isoform X2	0.485
GB40877	translocon-associated protein subunit delta	0.485
GB44205	proteasome subunit beta type-5-like	0.484
GB54151	uncharacterized protein LOC408463 isoform X12	-0.484
GB54590	polyadenylate-binding protein 1-like isoform X2	0.483
GB41157	RPII140-upstream gene protein-like	-0.481
GB48423	small nuclear ribonucleoprotein F isoform 2	0.480
GB49608	protein angel-like isoform X1	-0.475
GB49812	RING-box protein 1A isoform X1	0.461
GB43697	mediator of RNA polymerase II transcription subunit 16 isoform X3	0.457
GB41553	Golgi phosphoprotein 3 homolog rotini-like isoform X1	0.455
GB43548	40S ribosomal protein SA	0.449
GB45181	probable Bax inhibitor 1	0.447
GB53086	alcohol dehydrogenase class-3 isoform X2	0.446
GB41724	uncharacterized protein LOC727081	-0.446
GB54533	protein unc-13 homolog D isoform X5	-0.445
GB40882	40S ribosomal protein S13 isoform X1	0.445
GB50230	V-type proton ATPase subunit e 2-like	0.445
102654691	protein translation factor SUI1 homolog	0.445
GB51787	myosin light chain alkali-like isoform X5	0.444
GB41908	PERQ amino acid-rich with GYF domain-containing protein CG11148-like isoform X3	-0.443
GB53138	inorganic pyrophosphatase-like	0.437
GB48250	putative gamma-glutamylcyclotransferase CG2811-like isoform X4	0.436
GB46763	excitatory amino acid transporter 3	0.436
GB53415	WW domain-binding protein 2-like isoform X1	0.432
GB47606	ER membrane protein complex subunit 4-like isoform 1	0.431
GB42675	adenylate cyclase type 2-like	-0.430
GB44312	hydroxyacylglutathione hydrolase, mitochondrial-like isoform X2	0.428
102654127	neurochondrin homolog	-0.423
GB47938	uncharacterized protein LOC412825 isoform X1	-0.421
GB55056	spermatogenesis-associated protein 20 isoform X2	-0.420
GB41084	60S ribosomal protein L38	0.412
GB47810	regulator of gene activity protein isoform X3	0.410
GB40946	serine/threonine-protein phosphatase 2A 65 kDa regulatory subunit A alpha isoform-like isoform X1	0.409
GB42780	CCHC-type zinc finger protein CG3800-like isoform X3	0.409
GB43229	GTP-binding nuclear protein Ran isoform X1	0.407
GB50244	NHL repeat-containing protein 2 isoform X4	-0.406
GB45684	protein spire-like isoform X4	-0.406
GB52073	probable citrate synthase 1, mitochondrial-like	-0.402
GB45856	protein GPR107-like isoform X4	0.401
GB47542	eukaryotic translation initiation factor 3 subunit J isoform 1	0.399
GB53243	LOW QUALITY PROTEIN: probable phosphorylase b kinase regulatory subunit beta-like	-0.395
GB55892	glutamate-rich WD repeat-containing protein 1-like	0.395
GB43105	casein kinase II subunit alpha isoform X6	0.390
GB43537	probable 28S ribosomal protein S16, mitochondrial	0.388
GB44496	probable serine incorporator isoformX1	0.376
GB43855	LOW QUALITY PROTEIN: coatomer subunit beta'	0.374
GB40073	COP9 signalosome complex subunit 8-like	0.373
GB47100	putative glutamate synthase [NADPH]-like isoform X4	-0.372
GB42786	microtubule-associated protein RP/EB family member 1-like isoform X4	0.372
GB54789	GMP synthase [glutamine-hydrolyzing]	0.371
GB40767	phosphoglycolate phosphatase-like	-0.370
GB52212	polyubiquitin-A-like isoform X2	0.370
GB52256	60S ribosomal protein L5	0.370

(continued)

Gene	Name	Log2 FC
GB50925	prostaglandin E synthase 2-like	-0.360
GB53725	splicing factor 3B subunit 1-like isoform X2	-0.355
GB44870	zinc finger protein 706-like isoform X3	0.355
GB45375	rhomboid-7 isoform X1	0.346
GB45044	uncharacterized protein LOC409396 isoform X5	-0.334
GB50909	dual 3',5'-cyclic-AMP and -GMP phosphodiesterase 11-like, transcript variant X4	-0.333
GB44907	myeloid leukemia factor isoform X3	0.331
GB44333	flocculation protein FLO11-like isoform X1	-0.322
GB46562	40S ribosomal protein S24-like isoform X2	0.321
GB45017	RNA pseudouridylate synthase domain-containing protein 2-like isoform X3	-0.317
GB48312	pre-mRNA-splicing factor RBM22-like	0.316
GB47103	elongation factor 1-beta'	0.310
GB48207	proteasomal ubiquitin receptor ADRM1 homolog isoform X1	0.270
GB40887	V-type proton ATPase subunit E isoform 3	0.266
GB41152	uncharacterized protein C6orf106 homolog	0.213
GB45678	1-acylglycerol-3-phosphate O-acyltransferase ABHD5-like isoform X1	-0.192
GB44576	ester hydrolase C11orf54 homolog	0.189

Supplementary Table 3: The single significantly differentially expressed gene (EBseq; FDR-corrected posterior probability of differential expression $p < 0.05$) in *Bombus terrestris*. Positive fold change values indicate higher expression in the control, while negative values indicate higher expression in the queen pheromone treatment. The second and third columns give the best BLAST hit for this gene in *A. mellifera* plus the name of the *A. mellifera* putative ortholog.

Gene	Apis BLAST	Name	Log2 FC
100648170	GB48391	mucin-2-like	1.071

Supplementary Table 4: List of the 290 significantly differentially expressed genes (EBseq; FDR-corrected posterior probability of differential expression $p < 0.05$) in *Lasius flavus*, listed in order of fold change in gene expression on a Log_2 scale. Positive fold change values indicate higher expression in the control, while negative values indicate higher expression in the queen pheromone treatment. The second and third columns give the best BLAST hit for this gene in *A. mellifera* plus the name of the *A. mellifera* putative ortholog.

Gene	Apis BLAST	Name	Log2 FC
TRINITY_DN19074_c0_g2	GB43902	hexaprenyldihydroxybenzoate methyltransferase, mitochondrial-like	7.514
TRINITY_DN2701_c0_g1	GB52729	aspartate–tRNA ligase, cytoplasmic	7.481
TRINITY_DN14108_c0_g2			7.468
TRINITY_DN36041_c0_g1			6.629
TRINITY_DN13621_c0_g1			6.549
TRINITY_DN18780_c0_g2			-6.440
TRINITY_DN14430_c0_g1			6.278
TRINITY_DN32671_c0_g2			6.121
TRINITY_DN14910_c0_g1	XP_016769216.1		5.800
TRINITY_DN19071_c0_g1			-5.776
TRINITY_DN5663_c0_g2	XP_006568418.2		-5.365
TRINITY_DN13527_c1_g3			5.259
TRINITY_DN2506_c0_g2			5.133
TRINITY_DN9902_c0_g2			5.069
TRINITY_DN6503_c0_g3	551397	28S ribosomal protein S18a, mitochondrial isoform 2	5.044
TRINITY_DN10699_c0_g1			4.901
TRINITY_DN5102_c0_g1	XP_016771437.1		4.901
TRINITY_DN6994_c0_g1			4.859
TRINITY_DN13376_c3_g2			4.858
TRINITY_DN9565_c0_g3	GB45250	uncharacterized protein LOC409595	4.811
TRINITY_DN1013_c0_g1	GB52059	eukaryotic translation initiation factor 4H-like isoform X1	4.709
TRINITY_DN11616_c0_g1			4.696
TRINITY_DN1845_c0_g2	GB52253	protein PRRC2C-like isoform X2	4.658
TRINITY_DN7242_c0_g1			-4.646
TRINITY_DN31547_c0_g3			4.613
TRINITY_DN13376_c3_g3			4.569
TRINITY_DN6298_c0_g3			4.553
TRINITY_DN2720_c0_g2			-4.522
TRINITY_DN2583_c0_g2			4.460
TRINITY_DN24980_c0_g3			-4.393
TRINITY_DN12959_c0_g3			4.390
TRINITY_DN4813_c0_g1			4.322
TRINITY_DN6331_c0_g2			4.303
TRINITY_DN10925_c0_g1			4.278
TRINITY_DN14174_c1_g3			4.233
TRINITY_DN23574_c0_g3			4.173
TRINITY_DN6054_c0_g1	XP_016771772.1		4.143
TRINITY_DN32287_c0_g1			4.133
TRINITY_DN1060_c0_g1			4.115
TRINITY_DN6965_c0_g3			4.097
TRINITY_DN5087_c0_g1	XP_003249576.2		4.069
TRINITY_DN10904_c0_g1			4.050
TRINITY_DN4900_c0_g1			4.049
TRINITY_DN14065_c0_g2			4.015
TRINITY_DN2102_c0_g6	GB40389	profilin	3.751
TRINITY_DN12195_c1_g3	102656074	reticulon-4-like isoform X6	3.710
TRINITY_DN2302_c0_g2			3.676
TRINITY_DN9563_c1_g3	XP_016770117.1		3.347
TRINITY_DN11562_c2_g1	XP_016769630.1		3.211
TRINITY_DN6503_c0_g2	551397	28S ribosomal protein S18a, mitochondrial isoform 2	3.210
TRINITY_DN3428_c0_g1	GB53155	maternal embryonic leucine zipper kinase-like	3.168
TRINITY_DN11833_c0_g2			3.162
TRINITY_DN1150_c0_g2			3.151
TRINITY_DN14156_c9_g2	XP_016769763.1		3.146
TRINITY_DN7447_c0_g1	GB10293	aubergine	3.125
TRINITY_DN12563_c0_g2	XP_016768561.1		3.097
TRINITY_DN19328_c0_g2	GB49105	ecdysteroid-regulated gene E74 isoform X10	2.921

(continued)

Gene	Apis BLAST	Name	Log2 FC
TRINITY_DN19071_c0_g5			-2.920
TRINITY_DN3355_c0_g1	XP_016772030.1		2.912
TRINITY_DN1453_c0_g2			2.891
TRINITY_DN34334_c0_g1			-2.831
TRINITY_DN9365_c0_g1			2.823
TRINITY_DN2907_c0_g1	GB52114	protein trachealess-like isoform X7	2.821
TRINITY_DN29060_c0_g1			2.784
TRINITY_DN5450_c0_g3			2.778
TRINITY_DN6679_c1_g1			-2.777
TRINITY_DN13106_c0_g1			-2.766
TRINITY_DN6249_c0_g2			2.762
TRINITY_DN12570_c0_g2	XP_016772046.1		2.752
TRINITY_DN29060_c0_g2			-2.748
TRINITY_DN1453_c0_g1			2.718
TRINITY_DN4551_c0_g1			2.665
TRINITY_DN5934_c0_g2			2.664
TRINITY_DN21319_c0_g1			2.647
TRINITY_DN16415_c0_g2			2.644
TRINITY_DN6639_c0_g2	GB51740	CD63 antigen	2.628
TRINITY_DN8686_c0_g6			2.525
TRINITY_DN27412_c0_g1			2.511
TRINITY_DN7556_c0_g1			2.479
TRINITY_DN12097_c3_g11			2.467
TRINITY_DN19324_c0_g1			2.464
TRINITY_DN5257_c0_g2	GB51614	probable methylthioribulose-1-phosphate dehydratase-like	2.451
TRINITY_DN3033_c0_g1	GB47735	endonuclease III-like protein 1-like	2.441
TRINITY_DN23065_c0_g1			2.434
TRINITY_DN30835_c0_g2			2.337
TRINITY_DN30278_c0_g7			2.328
TRINITY_DN13221_c0_g7			2.244
TRINITY_DN13083_c0_g1			2.202
TRINITY_DN12097_c3_g6			2.167
TRINITY_DN6372_c0_g3			2.154
TRINITY_DN13237_c2_g6			-2.137
TRINITY_DN3870_c0_g2			2.108
TRINITY_DN7016_c0_g2	GB47843	uncharacterized protein LOC100576559 isoform X2	1.972
TRINITY_DN12195_c1_g4	102656074	reticulon-4-like isoform X6	-1.727
TRINITY_DN15459_c0_g1			1.644
TRINITY_DN7587_c0_g2	GB52590	fatty acid synthase-like isoform 1	-1.363
TRINITY_DN3266_c0_g1			1.320
TRINITY_DN5353_c0_g1	GB43825	lysosomal aspartic protease	1.297
TRINITY_DN7865_c0_g1	XP_016770671.1		1.257
TRINITY_DN13667_c2_g1			1.226
TRINITY_DN9568_c0_g3			-1.068
TRINITY_DN8668_c0_g1	GB52590	fatty acid synthase-like isoform 1	-1.025
TRINITY_DN8075_c0_g1			-1.023
TRINITY_DN8944_c1_g2			-0.955
TRINITY_DN13195_c1_g2			0.950
TRINITY_DN11726_c1_g1	GB52590	fatty acid synthase-like isoform 1	-0.932
TRINITY_DN14247_c8_g2	GB52590	fatty acid synthase-like isoform 1	-0.926
TRINITY_DN61_c0_g1	GB45775	pancreatic triacylglycerol lipase-like isoform X2	-0.888
TRINITY_DN2623_c0_g1	GB43825	lysosomal aspartic protease	0.862
TRINITY_DN12575_c0_g1	GB55263	putative fatty acyl-CoA reductase CG5065-like	-0.832
TRINITY_DN14020_c0_g1	GB46188	trichohyalin-like isoform X1	0.728
TRINITY_DN13013_c0_g1	XP_016768441.1		0.710
TRINITY_DN12756_c2_g1			0.691
TRINITY_DN9649_c0_g1	NP_001305411.1		0.685
TRINITY_DN13574_c1_g2	GB40681	elongation of very long chain fatty acids protein 1-like	-0.679
TRINITY_DN9287_c0_g1	XP_016768964.1		0.671
TRINITY_DN13318_c0_g1	GB46888	alpha-methylacyl-CoA racemase-like	-0.612
TRINITY_DN3111_c0_g1			0.588
TRINITY_DN13226_c0_g1	GB47475	protein lethal(2)essential for life-like isoform 1	0.586

(continued)

Gene	Apis BLAST	Name	Log2 FC
TRINITY_DN5134_c0_g1	XP_016770229.1		0.578
TRINITY_DN12647_c0_g1	XP_016773029.1		-0.572
TRINITY_DN14059_c0_g1	XP_016768888.1		0.567
TRINITY_DN13252_c1_g1	GB50415	diacylglycerol kinase theta-like isoform X7	0.559
TRINITY_DN13742_c0_g1	GB46657	galactokinase-like	-0.518
TRINITY_DN13982_c0_g1	XP_016769706.1		-0.517
TRINITY_DN32324_c0_g1	102656101	uncharacterized protein LOC102656101	0.517
TRINITY_DN12496_c0_g1	GB54423	uncharacterized protein LOC551958	0.511
TRINITY_DN11328_c0_g1	GB51479	ras guanine nucleotide exchange factor P-like isoform X3	0.509
TRINITY_DN6523_c0_g1	GB40976	heat shock protein 90	-0.504
TRINITY_DN12344_c0_g1			0.503
TRINITY_DN15124_c0_g1			0.500
TRINITY_DN12742_c1_g1	XP_016767680.1		0.490
TRINITY_DN11193_c0_g1	GB53045	ATP-binding cassette sub-family G member 1-like isoform X1	-0.481
TRINITY_DN10448_c0_g2	GB41603	PTB domain-containing adapter protein ced-6 isoform X2	-0.474
TRINITY_DN1154_c0_g1			0.474
TRINITY_DN14164_c3_g1	GB55490	uncharacterized protein LOC410793	-0.473
TRINITY_DN14136_c5_g1	GB55016	quinone oxidoreductase-like isoform X2	0.465
TRINITY_DN9952_c0_g1	GB43823	chemosensory protein 1 precursor	0.462
TRINITY_DN8450_c0_g1	GB46286	zinc carboxypeptidase A 1-like isoform X1	0.454
TRINITY_DN12807_c0_g1	XP_016769434.1		0.450
TRINITY_DN13250_c5_g1	GB45937	intracellular protein transport protein USO1 isoform X2	0.445
TRINITY_DN6544_c0_g1	GB52074	6-phosphogluconate dehydrogenase, decarboxylating	-0.445
TRINITY_DN13581_c2_g2	GB42792	uncharacterized protein LOC409805 isoform X3	0.444
TRINITY_DN36181_c0_g1			0.442
TRINITY_DN2719_c0_g1	GB54446	arginine kinase isoform X2	0.441
TRINITY_DN13519_c0_g1	GB42797	protein takeout-like	0.440
TRINITY_DN7060_c0_g1	GB49607	lysosome-associated membrane glycoprotein 1-like isoform 2	-0.438
TRINITY_DN1392_c0_g1	GB44205	proteasome subunit beta type-5-like	-0.434
TRINITY_DN10466_c0_g1	GB44431	26S protease regulatory subunit 4 isoform 1	-0.428
TRINITY_DN13148_c0_g1	GB44213	filamin-like	0.426
TRINITY_DN13702_c3_g1	XP_016769732.1		0.423
TRINITY_DN12417_c0_g2	GB45456	flocculation protein FLO11-like isoform X2	0.422
TRINITY_DN23399_c0_g1			0.417
TRINITY_DN11737_c0_g1	XP_016766478.1		-0.416
TRINITY_DN12348_c0_g1	GB44703	proteasome activator complex subunit 4-like	-0.415
TRINITY_DN13581_c1_g3	XP_016767189.1		0.406
TRINITY_DN11774_c0_g1	XP_016772498.1		0.387
TRINITY_DN3048_c0_g1	GB40770	dehydrogenase/reductase SDR family member 11-like isoform X2	0.387
TRINITY_DN13700_c5_g3	GB42840	leukocyte receptor cluster member 8 homolog isoform X4	0.387
TRINITY_DN13455_c0_g1	GB45128	trifunctional enzyme subunit alpha, mitochondrial-like	-0.380
TRINITY_DN14019_c2_g1	409060	neurofilament heavy polypeptide-like isoform X2	0.377
TRINITY_DN11786_c1_g1	GB51214	troponin T, skeletal muscle	0.374
TRINITY_DN9982_c0_g1	GB51787	myosin light chain alkali-like isoform X5	0.372
TRINITY_DN27322_c0_g1	GB40866	heat shock protein cognate 4	-0.369
TRINITY_DN5956_c0_g1			0.366
TRINITY_DN6208_c0_g1	GB49757	fatty acid binding protein	0.359
TRINITY_DN11594_c0_g1	GB52643	poly(U)-specific endoribonuclease homolog	0.355
TRINITY_DN9687_c0_g1			0.350
TRINITY_DN14119_c3_g1	726668	PDZ and LIM domain protein 3 isoform X7	0.348
TRINITY_DN5256_c0_g1			0.345
TRINITY_DN10396_c0_g1	GB42607	cytochrome b5-like isoform X1	-0.344
TRINITY_DN5623_c0_g1	GB54817	muscle-specific protein 20	0.343
TRINITY_DN10620_c0_g1	GB42732	long-chain-fatty-acid–CoA ligase 3-like isoform X2	-0.325
TRINITY_DN12788_c0_g1	XP_016771468.1		0.325
TRINITY_DN12757_c0_g1	GB55610	MOSC domain-containing protein 2, mitochondrial-like	0.324
TRINITY_DN10923_c0_g1	GB40141	venom serine carboxypeptidase	-0.321
TRINITY_DN10232_c0_g1			-0.319
TRINITY_DN12105_c0_g1	XP_016768441.1		0.318
TRINITY_DN7549_c0_g1	XP_016768456.1		0.315
TRINITY_DN3036_c0_g1	GB52326	chemosensory protein 4 precursor	0.313

(continued)

Gene	Apis BLAST	Name	Log2 FC
TRINITY_DN12756_c2_g4	XP_016770894.1		0.311
TRINITY_DN14002_c3_g1	XP_016770982.1		0.307
TRINITY_DN27284_c0_g1	GB50274	transitional endoplasmic reticulum ATPase TER94	-0.306
TRINITY_DN12138_c0_g1	GB47306	sulfhydryl oxidase 1-like	0.305
TRINITY_DN13865_c0_g1	GB47963	probable E3 ubiquitin-protein ligase HERC4-like isoform X3	0.302
TRINITY_DN8423_c0_g1	XP_016768214.1		-0.298
TRINITY_DN27569_c0_g1	GB52736	ATP synthase subunit beta, mitochondrial isoform X1	0.297
TRINITY_DN14286_c2_g1	GB54861	LOW QUALITY PROTEIN: counting factor associated protein D-like	-0.294
TRINITY_DN14128_c1_g1	XP_006568818.2		0.291
TRINITY_DN11359_c0_g1	XP_016768872.1		-0.287
TRINITY_DN9072_c0_g1	XP_016768321.1		-0.287
TRINITY_DN28113_c0_g1			-0.285
TRINITY_DN10911_c0_g1	XP_016770213.1		-0.277
TRINITY_DN12726_c3_g1	GB42787	dentin sialophosphoprotein-like isoform X4	-0.274
TRINITY_DN6072_c0_g1	XP_016771431.1		-0.273
TRINITY_DN13789_c0_g2	XP_016767109.1		0.273
TRINITY_DN14037_c0_g1	XP_016767155.1		0.272
TRINITY_DN13738_c0_g1	XP_016772667.1		0.271
TRINITY_DN993_c0_g3			0.270
TRINITY_DN7531_c0_g3	GB51710	eukaryotic initiation factor 4A-like isoformX2	0.260
TRINITY_DN11232_c0_g1	GB40240	myosin regulatory light chain 2	0.258
TRINITY_DN12074_c1_g1	GB47462	protein disulfide-isomerase A3 isoform 2	-0.256
TRINITY_DN11684_c0_g1	GB55537	transketolase isoform 1	-0.248
TRINITY_DN14138_c2_g1	GB48850	fatty-acid amide hydrolase 2-B-like	-0.248
TRINITY_DN13963_c1_g1	XP_016768450.1		0.247
TRINITY_DN12180_c0_g1	XP_016772046.1		0.246
TRINITY_DN13233_c1_g1	XP_016768217.1		-0.244
TRINITY_DN12080_c1_g1	XP_016769481.1		-0.241
TRINITY_DN11171_c0_g1	GB42468	phospholipase B1, membrane-associated-like isoform X1	-0.239
TRINITY_DN13982_c0_g3	XP_016769706.1		-0.237
TRINITY_DN14752_c0_g1	GB50123	myophilin-like	0.226
TRINITY_DN8270_c0_g1	GB43276	aminopeptidase N-like isoform X1	0.226
TRINITY_DN12844_c1_g1	GB47885	probable cytochrome P450 304a1	-0.224
TRINITY_DN11885_c1_g1	GB55598	troponin I isoform X23	0.218
TRINITY_DN8742_c0_g1	XP_016767150.1		-0.216
TRINITY_DN14111_c0_g1	GB46705	muscle M-line assembly protein unc-89 isoform X5	0.213
TRINITY_DN14002_c4_g1			0.212
TRINITY_DN7802_c0_g1	GB41358	elongation factor 1-alpha	-0.210
TRINITY_DN14006_c0_g1	XP_016767101.1		-0.208
TRINITY_DN8847_c0_g1	GB46772	very-long-chain enoyl-CoA reductase-like	-0.201
TRINITY_DN14179_c1_g1	GB40461	calreticulin	-0.186
TRINITY_DN7523_c0_g1			-0.183
TRINITY_DN12909_c0_g1	XP_016770377.1		0.181
TRINITY_DN8174_c0_g1	GB47880	superoxide dismutase 1	-0.175
TRINITY_DN14199_c2_g1			-0.173
TRINITY_DN3676_c0_g2	GB49773	sequestosome-1	0.168
TRINITY_DN3648_c0_g1	GB45181	probable Bax inhibitor 1	-0.167
TRINITY_DN9738_c0_g1	GB44206	death-associated protein 1-like	-0.167
TRINITY_DN3697_c0_g1	GB54368	prostaglandin E synthase 3-like isoform X2	-0.165
TRINITY_DN13223_c0_g1	GB54315	uncharacterized protein LOC724126	-0.157
TRINITY_DN9957_c0_g1	GB43831	ATP-binding cassette sub-family D member 3-like	-0.157
TRINITY_DN1533_c0_g1	XP_392401.3		-0.155
TRINITY_DN12307_c0_g1	GB47029	uncharacterized protein LOC724558	-0.153
TRINITY_DN13021_c0_g1	XP_006571535.2		-0.144
TRINITY_DN11571_c0_g2	XP_001119981.3		-0.138
TRINITY_DN14152_c0_g10	XP_016771978.1		0.136
TRINITY_DN13002_c1_g1	XP_016767675.1		-0.131
TRINITY_DN13997_c1_g2	XP_016771269.1		0.130
TRINITY_DN7931_c0_g1	GB49321	D-arabinitol dehydrogenase 1-like	-0.128
TRINITY_DN12756_c2_g5	XP_016770894.1		-0.126
TRINITY_DN1575_c0_g1			0.124

(continued)

Gene	Apis BLAST	Name	Log2 FC
TRINITY_DN1616_c0_g1	GB41545	MD-2-related lipid-recognition protein-like	0.120
TRINITY_DN12630_c0_g1	GB45258	isocitrate dehydrogenase [NADP] cytoplasmic isoform 2	-0.119
TRINITY_DN14083_c3_g1	GB55263	putative fatty acyl-CoA reductase CG5065-like	-0.117
TRINITY_DN4494_c0_g2	GB47990	tropomyosin-1-like	0.116
TRINITY_DN1524_c0_g1	GB46920	iron-sulfur cluster assembly enzyme ISCU, mitochondrial	0.115
TRINITY_DN13221_c0_g9	XP_016769341.1		0.107
TRINITY_DN13959_c2_g1	GB49688	peroxidase isoformX2	0.104
TRINITY_DN12634_c0_g1	GB43575	trehalase-like isoform X2	-0.093
TRINITY_DN13844_c1_g1	XP_016767538.1		-0.092
TRINITY_DN10322_c0_g1	XP_016769014.1		-0.088
TRINITY_DN13381_c0_g1	GB51633	protein HIRA homolog	0.084
TRINITY_DN12970_c0_g2	GB44208	WD repeat-containing protein 37-like isoform X4	0.083
TRINITY_DN3647_c0_g1	GB53550	heat shock protein beta-1-like isoform X3	-0.081
TRINITY_DN8558_c0_g1	GB46713	translation elongation factor 2-like isoform 1	-0.079
TRINITY_DN11372_c0_g1	GB53755	juvenile hormone esterase precursor	0.071
TRINITY_DN6049_c0_g1			0.068
TRINITY_DN10813_c0_g1	GB51753	uncharacterized protein LOC100576760 isoform X2	-0.066
TRINITY_DN10427_c0_g1	GB51782	carboxypeptidase Q-like isoform 1	-0.065
TRINITY_DN8685_c0_g4	GB43825	lysosomal aspartic protease	-0.065
TRINITY_DN13047_c0_g1	XP_016768229.1		0.061
TRINITY_DN6208_c0_g2	GB49757	fatty acid binding protein	-0.056
TRINITY_DN13652_c0_g1	GB42422	ADP/ATP translocase	0.054
TRINITY_DN13884_c0_g2	GB47405	neutral alpha-glucosidase AB-like isoform 2	0.051
TRINITY_DN13634_c0_g1	GB45913	protein lethal(2)essential for life-like	-0.049
TRINITY_DN1723_c0_g1	GB52324	chemosensory protein 3 precursor	0.048
TRINITY_DN18895_c0_g1	GB55581	membrane-associated progesterone receptor component 1-like isoform 2	-0.044
TRINITY_DN8126_c0_g1	GB40779	transaldolase	-0.044
TRINITY_DN10665_c0_g1	GB42829	juvenile hormone epoxide hydrolase 1	-0.042
TRINITY_DN8184_c0_g1			0.040
TRINITY_DN11577_c0_g1	GB55096	NADP-dependent malic enzyme isoform X3	0.040
TRINITY_DN18992_c0_g1	XP_016772082.1		0.039
TRINITY_DN11030_c0_g1	GB49240	aldehyde dehydrogenase, mitochondrial isoform 1	-0.038
TRINITY_DN11459_c0_g1	GB50598	aldose reductase-like isoform 1	-0.038
TRINITY_DN13961_c1_g3	GB52588	conserved oligomeric Golgi complex subunit 7	-0.037
TRINITY_DN12872_c0_g1	GB53333	V-type proton ATPase catalytic subunit A-like isoform X3	0.035
TRINITY_DN13271_c0_g1	GB40312	choline/ethanolamine kinase-like isoform X4	0.030
TRINITY_DN13702_c8_g1	GB47395	uncharacterized protein CG7816-like	-0.030
TRINITY_DN13400_c0_g1	GB54421	uncharacterized protein DDB_G0287625-like	0.023
TRINITY_DN10682_c0_g1	GB50252	GTP-binding protein SAR1b-like isoform X4	-0.021
TRINITY_DN8055_c0_g1			-0.021
TRINITY_DN9151_c0_g1	GB45147	clavesin-2-like	0.021
TRINITY_DN11885_c3_g1	GB47880	superoxide dismutase 1	-0.019
TRINITY_DN12979_c1_g1	GB49347	prostaglandin reductase 1-like	0.013
TRINITY_DN14085_c1_g1	XP_016769919.1		-0.011
TRINITY_DN11118_c1_g1	GB44422	uncharacterized protein LOC412543 isoform X3	-0.010
TRINITY_DN14002_c1_g1			0.004
TRINITY_DN7306_c0_g2	XP_016769332.1		-0.004
TRINITY_DN13747_c0_g1	XP_016769944.1		-0.003
TRINITY_DN10790_c0_g1			0.000

Supplementary Table 5: List of the 135 significantly differentially expressed genes (EBseq; FDR-corrected posterior probability of differential expression $p < 0.05$) in *Lasius niger*, listed in order of fold change in gene expression on a Log_2 scale. Positive fold change values indicate higher expression in the control, while negative values indicate higher expression in the queen pheromone treatment. The second and third columns give the best BLAST hit for this gene in *A. mellifera* plus the name of the *A. mellifera* putative ortholog.

Gene	Apis BLAST	Name	Log2 FC
XLOC_001009			5.916
RF55_9944	GB55171	major royal jelly protein 1 isoform X1	5.060
RF55_873	XP_016773511.1		4.638
XLOC_000784			3.626
RF55_874	XP_016766165.1		3.550
XLOC_016588			3.077
RF55_9436			2.813
RF55_3510	GB53672	failed axon connections isoform X2	-2.471
RF55_15864			-2.439
XLOC_020552			2.437
RF55_783			2.206
RF55_6001			-2.150
XLOC_013573			2.150
RF55_4870	XP_006563262.2		-2.031
XLOC_022706			-1.091
RF55_7689	XP_003250465.2		-1.030
RF55_19841	GB52590	fatty acid synthase-like isoform 1	-1.015
RF55_21338	GB52590	fatty acid synthase-like isoform 1	-0.923
RF55_13568	XP_006571191.2		0.882
RF55_2210	GB49869	microsomal triglyceride transfer protein large subunit isoform X1	-0.879
RF55_6639	GB48784	cytochrome c	0.779
RF55_15245	GB43617	uncharacterized membrane protein DDB_G0293934-like isoform X1	0.734
RF55_14443	GB51356	cytochrome P450 4G11	0.726
RF55_5140	XP_016767978.1		0.656
XLOC_003947			0.655
XLOC_019296			-0.643
XLOC_005895			-0.625
XLOC_010120			0.619
RF55_3431	GB47849	pyrroline-5-carboxylate reductase 2-like isoform X2	0.611
RF55_6542	GB52074	6-phosphogluconate dehydrogenase, decarboxylating	-0.603
RF55_6567	552211	protein THEM6-like	-0.597
RF55_11093	GB51174	uncharacterized protein DDB_G0284459-like	0.593
RF55_9960	GB52023	cytochrome P450 6AQ1 isoform X3	-0.584
RF55_14139	XP_016768457.1		0.561
RF55_16317	XP_016770827.1		-0.523
RF55_16054	GB55082	protein PBDC1-like	0.489
XLOC_004490			-0.474
RF55_9918	GB47885	probable cytochrome P450 304a1	0.470
XLOC_015751			-0.442
RF55_12610	GB40866	heat shock protein cognate 4	-0.432
RF55_11067	GB46772	very-long-chain enoyl-CoA reductase-like	-0.431
RF55_6451	GB55598	troponin I isoform X23	0.430
RF55_10641	XP_016769078.1		-0.430
RF55_4656	GB42792	uncharacterized protein LOC409805 isoform X3	0.415
RF55_4036	GB44208	WD repeat-containing protein 37-like isoform X4	0.407
XLOC_002901			-0.398
RF55_16927	XP_006564499.2		0.394
RF55_4554	GB47990	tropomyosin-1-like	0.393
RF55_15079	GB41028	ATP synthase subunit alpha, mitochondrial isoform 1	0.375
RF55_3507	GB41333	DNA-directed RNA polymerase III subunit RPC1-like isoform X1	-0.375
RF55_5177	GB43902	hexaprenyldihydroxybenzoate methyltransferase, mitochondrial-like	-0.368
RF55_13988	GB55263	putative fatty acyl-CoA reductase CG5065-like	-0.362
RF55_10649			0.359
RF55_2038	GB51787	myosin light chain alkali-like isoform X5	0.350
RF55_3707	GB55537	transketolase isoform 1	-0.342
XLOC_005759			0.330
RF55_10912	GB47880	superoxide dismutase 1	-0.324

(continued)

Gene	Apis BLAST	Name	Log2 FC
RF55_18605	XP_016770827.1		-0.315
RF55_17057	GB52590	fatty acid synthase-like isoform 1	-0.312
RF55_3648	XP_016768682.1		-0.310
RF55_5902	GB55302	trehalose transporter 1 isoform X6	0.308
RF55_12242	GB41912	trans-1,2-dihydrobenzene-1,2-diol dehydrogenase-like	-0.286
RF55_10676	GB40240	myosin regulatory light chain 2	0.279
RF55_4654	GB45673	alpha-N-acetylgalactosaminidase-like	0.277
XLOC_012799			0.273
XLOC_018477			-0.268
RF55_6754	XP_016772080.1		0.254
RF55_3967	GB46039	tubulin alpha-1 chain-like	-0.251
RF55_1582	GB45012	adenosylhomocysteinase-like	0.248
RF55_2761	XP_003249233.2		0.246
RF55_2493	GB45913	protein lethal(2)essential for life-like	-0.244
RF55_15035	GB51356	cytochrome P450 4G11	-0.223
RF55_5799	GB50123	myophilin-like	0.216
RF55_5341	GB50508	fibrillin-2	-0.215
RF55_598	GB40021	probable serine/threonine-protein kinase clkA-like	-0.215
RF55_5109	XP_016767675.1		-0.211
RF55_2407	XP_016768440.1		0.207
RF55_3343	GB46290	acetyl-coenzyme A synthetase-like	-0.205
RF55_10752	XP_016771487.1		0.204
RF55_19196	GB41311	actin, indirect flight muscle-like	0.202
RF55_5837	GB43879	aquaporin AQPcic-like isoform X2	-0.191
RF55_13604	XP_016767981.1		0.185
RF55_3994	GB49175	4-hydroxyphenylpyruvate dioxygenase-like	0.181
RF55_18796	GB53412	fatty acid synthase-like	-0.180
XLOC_001770			0.175
RF55_1934	GB54827	synaptotagmin 1	-0.169
RF55_5219	GB51753	uncharacterized protein LOC100576760 isoform X2	-0.163
RF55_778	GB42422	ADP/ATP translocase	0.154
RF55_10519	GB54446	arginine kinase isoform X2	0.141
RF55_364			0.137
RF55_2231	XP_016773593.1		-0.134
XLOC_008839			-0.132
RF55_5431			-0.129
RF55_5198	XP_016768517.1		0.124
RF55_11370	XP_016772844.1		-0.115
RF55_13251	GB52590	fatty acid synthase-like isoform 1	-0.113
RF55_6842	GB43052	paramyosin, long form-like	0.109
RF55_6077	GB54423	uncharacterized protein LOC551958	-0.108
RF55_6180	GB40758	icarapin-like	0.108
XLOC_005990			-0.099
RF55_10150	XP_016769706.1		0.099
RF55_1045	GB54861	LOW QUALITY PROTEIN: counting factor associated protein D-like	-0.097
RF55_3186	XP_016770377.1		0.092
XLOC_020265			-0.089
XLOC_019117			-0.085
RF55_4175	GB44311	actin related protein 1	0.083
XLOC_016272			-0.079
RF55_5206	GB40735	fructose-bisphosphate aldolase-like isoform X1	0.071
RF55_4024	XP_016767817.1		0.069
RF55_9453	GB55096	NADP-dependent malic enzyme isoform X3	-0.067
RF55_8355	GB42809	translationally-controlled tumor protein homolog isoform 1	0.063
RF55_3575	XP_016768967.1		-0.062
XLOC_011290			0.061
RF55_5559	GB52107	tubulin alpha-1 chain-like	0.061
RF55_3308			-0.059
XLOC_020794			0.059
XLOC_018966			0.059
RF55_1104	XP_016769017.1		0.048
RF55_652	GB54354	uncharacterized protein DDB_G0274915-like isoform X2	0.045

(continued)

Gene	Apis BLAST	Name	Log2 FC
RF55_3854	XP_006571125.2		-0.036
RF55_6873	GB47106	NADH-ubiquinone oxidoreductase 75 kDa subunit, mitochondrial	-0.035
RF55_15158	GB42794	circadian clock-controlled protein-like isoform 1	-0.034
RF55_4888	GB40779	transaldolase	0.034
XLOC_019193			0.026
XLOC_013126			-0.025
XLOC_017016			0.025
XLOC_016458			-0.024
XLOC_013131			-0.013
RF55_10886	GB41427	catalase	-0.012
XLOC_003165			-0.010
RF55_9333	GB49688	peroxidase isoformX2	0.008
XLOC_004885			-0.005
RF55_5677	GB46713	translation elongation factor 2-like isoform 1	0.004
RF55_14822	GB43823	chemosensory protein 1 precursor	0.004
RF55_11002	XP_016768909.1		0.002

Supplementary Table 6: All orthologous genes that were significantly differentially expressed between pheromone treatments in more than one species. The FC columns give the Log_2 fold-change in expression for each species where the focal gene was significantly differentially expressed, where positive numbers mean it was expressed at a higher level in control animals. The last column highlights genes that responded to treatment in a consistent or inconsistent direction across species. *B. terrestris* is omitted because neither of its differentially expressed genes were significantly affected by treatment in the other three species.

name	Apis FC	L. flavus FC	L. niger FC	Consistent
myosin light chain alkali-like isoform X5	0.444	0.372	0.350	Yes
proteasome subunit beta type-5-like	0.484	-0.434		No
probable Bax inhibitor 1	0.447	-0.167		No
intracellular protein transport protein USO1 isoform X2	-0.684	0.445		No
muscle M-line assembly protein unc-89 isoform X5	-0.526	0.213		No
transitional endoplasmic reticulum ATPase TER94	0.544	-0.306		No
actin related protein 1	0.762		0.083	Yes
tubulin alpha-1 chain-like	0.773		-0.251	No
Unknown gene 1		-0.088	0.048	No
uncharacterized protein LOC100576760 isoform X2		-0.066	-0.163	Yes
myosin regulatory light chain 2		0.258	0.279	Yes
NADP-dependent malic enzyme isoform X3		0.040	-0.067	No
transketolase isoform 1		-0.248	-0.342	Yes
troponin T, skeletal muscle		0.374	0.185	Yes
uncharacterized protein LOC551958		0.511	-0.108	No
probable cytochrome P450 304a1		-0.224	0.470	No
Unknown gene 2		0.181	0.092	Yes
WD repeat-containing protein 37-like isoform X4		0.083	0.407	Yes
Unknown gene 3		-0.131	-0.211	Yes
uncharacterized protein LOC409805 isoform X3		0.444	0.415	Yes
ADP/ATP translocase		0.054	0.154	Yes
peroxidase isoformX2		0.104	0.008	Yes
neurofilament heavy polypeptide-like isoform X2		0.377	0.069	Yes
fatty acid synthase-like isoform 1		-0.926	-1.015	Yes
LOW QUALITY PROTEIN: counting factor associated protein D-like		-0.294	-0.097	Yes
myophilin-like		0.226	0.216	Yes
Unknown gene 4		0.039	0.254	Yes
hexaprenyldihydroxybenzoate methyltransferase, mitochondrial-like		7.514	-0.368	No
arginine kinase isoform X2		0.441	0.141	Yes
heat shock protein cognate 4		-0.369	-0.432	Yes
tropomyosin-1-like		0.116	0.393	Yes
6-phosphogluconate dehydrogenase, decarboxylating		-0.445	-0.603	Yes
transaldolase		-0.044	0.034	No
superoxide dismutase 1		-0.175	-0.324	Yes
translation elongation factor 2-like isoform 1		-0.079	0.004	No
very-long-chain enoyl-CoA reductase-like		-0.201	-0.431	Yes
Unknown gene 5		0.350	0.359	Yes

Supplementary Table 7: The overlap between the lists of significantly differently expressed orthologous genes was significantly higher than expected for *L. flavus* and *L. niger*, suggesting that queen pheromone has conserved effects on gene expression between these two species (results based on a hypergeometric test). For the other two species pairs, the number of overlapping genes was not higher or lower than expected under the null hypothesis that queen pheromone affects a random set of genes in each species. The last column gives the number of genes that overlapped, divided by the maximum number that *could* have overlapped given the numbers of orthologous genes that were significant in each species.

Species	Test	p	% of maximum possible overlap
Apis and L. flavus	Overlap is higher than expected:	0.1914514	5.8
Apis and L. niger	Overlap is higher than expected:	0.2616356	6.1
L. flavus and L. niger	Overlap is higher than expected:	0.0000000	42.3

Supplementary Table 8: List of genes that appear in the top n-most pheromone-sensitive genes for 3 or 4 species. To generate the table, we ranked genes by the absolute value of their log fold change in response to queen pheromone, then listed the gene names that appeared in 3-4 species. For non-*Apis* species, we found the gene names by comparison with the *Apis* genome by BLAST. This exercise was performed with $n = 100$, 200 ... 500, and the third column lists the smallest n for which the gene in question appeared (for example, the gene *protein takeout-like* appeared for all 4 species when inspecting the top 200+ genes).

Name	Appears in	Size of gene set
protein takeout-like	4 species	200
glucose dehydrogenase [FAD, quinone]	4 species	300
histone-lysine N-methyltransferase SETMAR-like	4 species	300
serotonin receptor	4 species	500
titin-like	4 species	500
uncharacterized protein LOC102656088	4 species	500
histone-lysine N-methyltransferase SETMAR-like	3 species	200
2-oxoglutarate dehydrogenase, mitochondrial-like isoform X5	3 species	300
probable serine/threonine-protein kinase DDB_G0282963 isoform X4	3 species	300
titin-like	3 species	300
elongation of very long chain fatty acids protein 6-like	3 species	400
ligand-gated chloride channel homolog 3 precursor	3 species	400
odorant receptor Or2-like	3 species	400
putative odorant receptor 13a-like	3 species	400
serotonin receptor	3 species	400
trypsin-1	3 species	400
uncharacterized protein LOC100576902	3 species	400
uncharacterized protein LOC102655422	3 species	400
uncharacterized protein LOC102656088	3 species	400
metabotropic glutamate receptor 7 isoform X3	3 species	500
probable cytochrome P450 305a1	3 species	500
protein NPC2 homolog	3 species	500
suppressor protein SRP40-like	3 species	500
uncharacterized protein LOC100577132	3 species	500
uncharacterized protein LOC102656830	3 species	500
uncharacterized protein LOC724216	3 species	500

Supplementary Table 9: Results of a permutation test examining the number of overlaps in the top n-most pheromone-sensitive genes for each pair of species. To generate the table, we ranked genes by the absolute value of their log fold change in response to queen pheromone, then took the top n-most pheromone-sensitive genes for each species, and counted the observed and expected number of overlaps (the expected number was estimated by bootstrapping with 10ˆ5 replicates). The O/E column gives the ratio of observed to expected, where numbers >1 indicate more overlap than expected. The one-tailed p-value was estimated as the proportion of bootstrap replicates showing more overlap than in the real dataset. This exercise was performed with $n = 100, 200 \ldots\ 500$.

Species pair	Size of gene set	Obs. overlaps	Exp. overlaps	O/E	p-value	
am-bt	100	1	0.9673	1.0338054	0.2481	
am-lf	100	2	0.9660	2.0703934	0.0737	
am-ln	100	2	0.9870	2.0263425	0.0779	
bt-lf	100	1	1.6086	0.6216586	0.4794	
bt-ln	100	4	1.6498	2.4245363	0.0214	sig
lf-ln	100	6	2.2807	2.6307713	0.0071	sig
am-bt	200	8	3.8312	2.0881186	0.0161	sig
am-lf	200	3	3.7871	0.7921629	0.5256	
am-ln	200	2	3.8231	0.5231357	0.7423	
bt-lf	200	4	6.3663	0.6283084	0.7699	
bt-ln	200	14	6.5862	2.1256567	0.0025	sig
lf-ln	200	14	8.6248	1.6232260	0.0210	sig
am-bt	300	18	8.5172	2.1133706	0.0006	sig
am-lf	300	6	8.3141	0.7216656	0.7369	
am-ln	300	8	8.4972	0.9414866	0.4789	
bt-lf	300	13	14.1987	0.9155768	0.5613	
bt-ln	300	28	14.6683	1.9088783	0.0006	sig
lf-ln	300	26	18.3796	1.4146119	0.0257	sig
am-bt	400	27	14.9963	1.8004441	0.0004	sig
am-lf	400	10	14.6418	0.6829761	0.8784	
am-ln	400	19	14.8848	1.2764700	0.1068	
bt-lf	400	24	24.9929	0.9602727	0.5264	
bt-ln	400	47	25.7413	1.8258596	0.0001	sig
lf-ln	400	38	31.2715	1.2151640	0.0799	
am-bt	500	36	23.1804	1.5530362	0.0028	sig
am-lf	500	16	22.6013	0.7079239	0.9101	
am-ln	500	22	22.9017	0.9606274	0.5178	
bt-lf	500	42	38.4808	1.0914534	0.2385	
bt-ln	500	62	39.7228	1.5608165	0.0000	sig
lf-ln	500	58	47.0932	1.2316003	0.0352	sig

Supplementary Table 10: Results of Spearman's rank correlations, testing whether the absolute log fold difference between pheromones treatments is correlated for a given pair of species. Positive coefficients (ρ, written as rho) indicate that on average, orthologous genes have similar sensitivity to queen pheromones. The p-values have been corrected for multiple testing with the Benjamini-Hochberg method.

Species1	Species2	rho	p	sig
Apis mellifera	Bombus terrestris	0.136	0e+00	***
Apis mellifera	Lasius flavus	0.100	0e+00	***
Apis mellifera	Lasius niger	0.077	2e-07	***
Bombus terrestris	Lasius flavus	0.159	0e+00	***
Bombus terrestris	Lasius niger	0.127	0e+00	***
Lasius flavus	Lasius niger	0.194	0e+00	***

Supplementary Table 11: List of genes showing statistically significant pheromone-induced alternative splicing in *A. mellifera.* These genes were defined as those that have at least two isoforms that are differentially expressed following pheromone treatment (EBseq; posterior probability of differential expression $p < 0.05$), and for which one isoform increases in expression while another decreases. The last two columns show the fold changes of the most down-regulated and most up-regulated isoforms, on a Log_2 scale.

Gene	Name	Lowest FC	Highest FC
GB44254	uncharacterized protein LOC411586	-10.211	7.329
GB55598	troponin I	-8.618	7.426
GB55650	ryanodine receptor 44F	-8.652	6.121
GB42000	cAMP-specific 3',5'-cyclic phosphodiesterase, isoforms N/G	-7.846	6.465
GB49567	sestrin-1-like isoformX1	-6.738	6.330
GB46113	uncharacterized protein LOC726188	-6.722	6.205
GB55237	disco-interacting protein 2	-6.413	5.787
GB44556	uncharacterized protein LOC411962	-5.319	6.426
GB50923	serine-protein kinase ATM	-5.498	6.155
GB50941	phosphatidate phosphatase LPIN2-like	-6.138	5.369
GB55848	DNA ligase 1-like	-6.025	5.365
GB42142	nuclear hormone receptor FTZ-F1	-6.048	5.254
GB45259	zinc finger protein 91-like	-5.714	5.492
GB52595	zinc finger and BTB domain-containing protein 20-like	-6.006	5.148
GB55225	sushi, von Willebrand factor type A, EGF and pentraxin domain-containing protein 1	-3.583	7.361
GB55102	F-box only protein 28-like	-5.678	5.133
GB42895	trichohyalin-like	-6.075	4.690
GB44373	protein zyg-11 homolog B-like	-2.715	7.860
GB49111	neuropathy target esterase sws	-1.862	8.360
GB46271	protein BCL9 homolog	-3.147	6.936
GB53431	inositol hexakisphosphate kinase 2-like	-6.342	3.337
GB44336	protein couch potato-like	-2.183	7.354
GB45277	multidrug resistance-associated protein 4-like	-8.114	1.337
GB40263	dentin sialophosphoprotein-like	-3.060	6.272
725417	uncharacterized protein LOC725417	-2.831	6.160
GB55517	uncharacterized protein LOC410000	-3.276	5.377
GB55429	CREB-regulated transcription coactivator 1-like	-5.373	3.184
GB55998	beta-1,4-N-acetylgalactosaminyltransferase bre-4	-2.728	5.596
GB52604	LIM domain-binding protein 2-like	-2.287	5.868
GB41734	reversion-inducing-cysteine-rich protein with kazal motifs	-5.970	1.939
GB49535	calcium/calmodulin-dependent protein kinase II	-1.995	5.806
GB45662	conserved oligomeric Golgi complex subunit 4-like	-5.358	2.141
GB40310	uncharacterized protein LOC411575	-0.945	6.458
GB51117	rho GTPase-activating protein 18-like	-0.971	6.356
GB41129	protein LMBR1L-like	-5.620	1.443
GB44876	uncharacterized protein LOC100576421	-5.365	1.641
GB50244	NHL repeat-containing protein 2	-0.937	5.958
GB47138	calcium-activated potassium channel slowpoke-like	-0.904	5.950
GB50099	uncharacterized abhydrolase domain-containing protein DDB_G0269086-like	-1.509	5.081
GB41908	PERQ amino acid-rich with GYF domain-containing protein CG11148-like	-1.005	5.516
GB52999	neurofilament heavy polypeptide-like	-2.739	3.679
GB51651	putative inorganic phosphate cotransporter-like	-5.417	0.931
GB50877	tyrosine-protein kinase Dnt	-2.506	3.157
GB43220	transcription termination factor 2	-3.223	1.834
GB40565	ribosomal protein S6 kinase alpha-5	-2.313	2.662
GB55467	neural cell adhesion molecule L1-like	-3.334	1.457
GB48034	protein numb-like, transcript variant X5	-1.013	3.770
GB55570	transmembrane protein 53-like	-2.069	2.656
GB49417	PITH domain-containing protein GA19395-like	-2.184	2.246
GB43282	guanine nucleotide-binding protein G(q) subunit alpha-like	-2.360	2.011
GB48573	probable multidrug resistance-associated protein lethal(2)03659-like	-2.129	1.987
GB50366	uncharacterized protein LOC551450	-0.841	1.144
GB46121	ubiquitin fusion degradation protein 1 homolog	-0.951	0.918
GB52052	venom carboxylesterase-6-like	-0.702	0.910

Supplementary Table 12: List of genes showing statistically significant pheromone-induced alternative splicing in *Lasius flavus*. These genes were defined as those that have at least two isoforms that are differentially expressed following pheromone treatment (EBseq; posterior probability of differential expression $p < 0.05$), and for which one isoform increases in expression while another decreases. The last two columns show the fold changes of the most down-regulated and most up-regulated isoforms, on a Log_2 scale.

Gene	Amel ortholog	Name	Lowest FC	Highest FC
TRINITY_DN11346_c0_g1	GB49250	heme oxygenase	-9.173	4.281
TRINITY_DN14030_c0_g1	XP_016769715.1		-4.570	6.106
TRINITY_DN13728_c1_g1	GB44606	AMP deaminase 2-like	-5.615	4.228
TRINITY_DN13759_c0_g1	XP_016766389.1		-3.781	5.565
TRINITY_DN13897_c0_g1	GB41293	histone acetyltransferase KAT8	-4.642	2.686
TRINITY_DN14126_c1_g2	GB43039	restin homolog	-1.879	5.360
TRINITY_DN12581_c0_g2	XP_016767063.1		-4.108	2.797
TRINITY_DN14084_c0_g1	XP_016772396.1		-2.406	4.161
TRINITY_DN13929_c0_g1	GB53852	paired amphipathic helix protein Sin3a	-2.088	4.420
TRINITY_DN13956_c0_g1	GB54341	RNA-binding protein 33-like	-1.984	4.515
TRINITY_DN13699_c0_g1	GB41835	sorting nexin-13-like	-2.520	3.914
TRINITY_DN13676_c0_g1	XP_016767354.1		-3.985	2.279
TRINITY_DN13889_c0_g1	GB44338	small G protein signaling modulator 3 homolog	-2.667	3.555
TRINITY_DN11861_c1_g1	GB43504	neural/ectodermal development factor IMP-L2	-2.220	3.424
TRINITY_DN14001_c0_g2	GB41033	facilitated trehalose transporter Tret1-like	-3.425	2.196
TRINITY_DN12063_c0_g1	GB49936	calcium uptake protein 1 homolog, mitochondrial-like	-1.957	3.635
TRINITY_DN13321_c0_g1	XP_016769905.1		-2.840	2.740
TRINITY_DN13931_c4_g1	GB47270	cytochrome P450 4C1	-2.250	2.887
TRINITY_DN12721_c1_g1	GB43391	transmembrane protein 8B-like	-2.179	2.823
TRINITY_DN11401_c0_g1	GB50510	uncharacterized protein LOC409674	-2.693	1.986
TRINITY_DN10695_c0_g1	XP_016773356.1		-2.119	2.488
TRINITY_DN13698_c3_g1	GB45025	mTERF domain-containing protein 1, mitochondrial-like	-1.966	2.555
TRINITY_DN13919_c0_g1	GB49593	cytosolic carboxypeptidase-like protein 5-like	-1.689	2.775
TRINITY_DN14156_c10_g1	XP_016768402.1		-2.084	2.375
TRINITY_DN13974_c0_g1	XP_016768210.1		-1.595	2.767
TRINITY_DN12596_c0_g1	GB40801	thioredoxin-like protein 4A-like	-1.708	2.337
TRINITY_DN13248_c0_g1	GB42981	beta-1,3-glucan-binding protein	-1.695	2.316
TRINITY_DN14247_c8_g2	GB52590	fatty acid synthase-like	-1.057	1.011
TRINITY_DN10832_c0_g1	GB54056	serine hydroxymethyltransferase, cytosolic	-1.253	0.361
TRINITY_DN11786_c1_g1	GB51214	troponin T, skeletal muscle	-0.409	0.847
TRINITY_DN13339_c0_g1	GB40735	fructose-bisphosphate aldolase-like	-0.171	0.399
TRINITY_DN13721_c8_g1	XP_016772716.1		-0.207	0.212
TRINITY_DN13271_c0_g1	GB40312	choline/ethanolamine kinase-like	-0.155	0.010
TRINITY_DN10322_c0_g1	XP_016769014.1		-0.113	0.029

Supplementary Table 13: List of genes showing statistically significant pheromone-induced alternative splicing in *Lasius flavus*. These genes were defined as those that have at least two isoforms that are differentially expressed following pheromone treatment (EBseq; posterior probability of differential expression $p < 0.05$), and for which one isoform increases in expression while another decreases. The last two columns show the fold changes of the most down-regulated and most up-regulated isoforms, on a Log_2 scale.

Gene	Amel ortholog	Name	Lowest FC	Highest FC
RF55_752	GB48208	protein argonaute-2	-3.293	6.447
RF55_883	XP_016770988.1		-5.907	3.599
RF55_4195	XP_016772612.1		-5.174	4.309
RF55_412	XP_016770021.1		-3.388	5.967
RF55_2523	XP_016770619.1		-4.765	4.035
RF55_3907	XP_016771611.1		-4.451	3.686
RF55_4516	GB54906	niemann-Pick C1 protein-like	-3.798	4.302
RF55_2336	GB54910	tyrosine-protein kinase Abl-like	-3.225	4.453
RF55_1192	XP_016769850.1		-1.948	5.730
XLOC_016164			-5.497	2.128
RF55_3254	XP_016773300.1		-2.731	4.891
RF55_1559	XP_016768510.1		-2.991	4.323
RF55_4335	XP_016767803.1		-3.810	3.431
RF55_6516	GB40718	thioredoxin reductase 1	-3.254	3.922
RF55_11163	GB53163	transient receptor potential channel pyrexia	-3.902	3.257
RF55_1926	XP_016766364.1		-1.790	5.315
RF55_9605	GB42484	aminopeptidase N-like isoformX1	-5.026	2.037
XLOC_003165			-0.670	6.390
RF55_4472	GB43953	cycle	-3.905	3.034
RF55_13096	XP_016767210.1		-4.835	2.091
RF55_9014	GB55507	FYVE, RhoGEF and PH domain-containing protein 4-like	-3.921	2.964
RF55_8972	XP_016771571.1		-4.889	1.983
RF55_4341	XP_392463.4		-3.232	3.610
XLOC_013280			-2.421	4.392
RF55_2163	GB55475	cullin-5	-1.950	4.847
RF55_1574	XP_016770060.1		-0.533	6.238
RF55_6406	XP_016767146.1		-2.055	4.665
RF55_1819	GB51068	arrestin homolog	-2.300	4.399
RF55_2357	XP_016769793.1		-4.971	1.713
RF55_2452	XP_016767413.1		-4.941	1.680
RF55_764	XP_001121384.4		-4.468	2.131
RF55_5343	XP_016766531.1		-3.377	3.201
RF55_2401	XP_016769193.1		-3.897	2.672
RF55_6625	XP_016770656.1		-2.071	4.477
RF55_4021	XP_016767419.1		-1.817	4.656
RF55_317	XP_016769975.1		-2.714	3.712
RF55_561	GB51542	PH-interacting protein	-3.360	2.995
RF55_2755	GB48836	uncharacterized protein LOC100577578	-4.539	1.798
RF55_357			-4.014	2.285
RF55_9274	XP_016767607.1		-4.110	2.100
RF55_1902	GB46211	zinc finger protein 665-like	-4.221	1.952
RF55_1336	XP_016767701.1		-4.216	1.932
RF55_1520	XP_016768717.1		-2.049	4.022
RF55_2217	GB47260	zinc finger protein 598-like	-1.961	4.105
RF55_6360	XP_003251881.3		-3.578	2.473
RF55_15946	XP_016767569.1		-2.320	3.724
RF55_4138	XP_016766515.1		-2.359	3.664
RF55_1328	GB41746	neogenin	-2.030	3.877
RF55_6748	GB52716	NAD kinase-like, transcript variant X11	-3.494	2.393
RF55_7315	GB55434	rab GDP dissociation inhibitor beta	-3.129	2.715
RF55_9900	XP_016767864.1		-3.551	2.280
RF55_1325	GB44695	lysophospholipase-like protein 1-like	-4.098	1.674
RF55_9036	XP_016768958.1		-3.941	1.782
RF55_742	XP_016769412.1		-2.761	2.958
RF55_9907	GB47039	dynamin	-3.990	1.713
RF55_9185	XP_016768847.1		-3.836	1.860
RF55_2976	GB42054	sodium/potassium-transporting ATPase subunit alpha	-2.057	3.604

(continued)

Gene	Amel ortholog	Name	Lowest FC	Highest FC
RF55_4649	GB43618	aconitate hydratase, mitochondrial-like	-5.041	0.572
RF55_12355	GB42664	probable ATP-dependent RNA helicase DDX43-like	-1.994	3.618
RF55_1934	GB54827	synaptotagmin 1	-5.391	0.191
RF55_360	GB46768	uncharacterized MFS-type transporter C09D4.1-like	-3.231	2.320
RF55_5123	XP_016772933.1		-2.736	2.811
RF55_3009	GB53317	dentin sialophosphoprotein-like	-3.764	1.779
RF55_5073	GB55540	zinc finger MYM-type protein 3-like	-1.862	3.663
RF55_9319	XP_016768793.1		-1.602	3.896
RF55_952	GB42014	E3 ubiquitin-protein ligase TRIP12-like	-1.664	3.631
RF55_3561	GB41659	endothelin-converting enzyme 1	-1.912	3.334
RF55_4175	GB44311	actin related protein 1	-1.441	3.773
RF55_7804			-2.544	2.630
RF55_1689	XP_016768411.1		-3.377	1.747
RF55_5052	XP_016773337.1		-2.418	2.684
RF55_457	GB40931	uncharacterized protein LOC409781	-2.351	2.695
RF55_7632	XP_016767095.1		-2.046	2.994
RF55_11518	GB41804	nardilysin	-2.281	2.751
RF55_4355	XP_016771542.1		-2.445	2.518
RF55_1518	XP_016768753.1		-3.093	1.861
RF55_5804	XP_016766957.1		-2.623	2.273
RF55_4425	GB51264	glutamine-dependent NAD(+) synthetase, transcript variant X3	-2.194	2.679
RF55_5947	XP_016768497.1		-2.036	2.699
RF55_3209	GB46684	monocarboxylate transporter 3-like	-2.250	2.446
RF55_4132	XP_006572145.2		-1.892	2.795
RF55_3822	XP_016769466.1		-2.589	2.077
RF55_8207	XP_016767693.1		-2.678	1.961
RF55_3129	GB53974	probable RNA helicase armi	-2.475	2.104
RF55_8822	GB41970	ras-like protein 2-like	-2.501	2.077
RF55_9449	GB55840	protein sidekick-1-like	-1.974	2.475
RF55_583	GB41366	protein MLP1-like	-2.515	1.925
RF55_5597	GB46705	muscle M-line assembly protein unc-89	-4.201	0.194
RF55_7779	GB40928	tripartite motif-containing protein 2-like	-1.906	2.463
RF55_1209	XP_016766933.1		-1.841	2.469
RF55_2958	XP_016771468.1		-2.247	1.945
RF55_7227	GB45211	troponin C type I	-3.495	0.696
RF55_15397	XP_016766611.1		-2.189	1.967
RF55_3792	GB42024	translation initiation factor 2	-2.347	1.770
XLOC_009000			-1.970	2.140
RF55_1494	XP_016771667.1		-1.826	2.225
RF55_2964	GB45277	multidrug resistance-associated protein 4-like	-1.941	2.099
RF55_13040	XP_016771370.1		-1.707	2.305
XLOC_009717			-1.857	2.130
RF55_6105	GB40496	aftiphilin-like	-1.929	2.041
RF55_2225	XP_016769102.1		-2.458	1.503
RF55_7166	GB51290	ultrabithorax	-1.857	2.095
RF55_2340	GB55485	DNA methyltransferase 3	-2.222	1.709
RF55_5141	GB51219	eye-specific diacylglycerol kinase	-1.577	2.334
RF55_3424	GB42681	mucin-5AC-like	-1.913	1.991
RF55_335	GB42666	serine palmitoyltransferase 2-like	-2.186	1.689
RF55_2921	GB40416	twist	-2.142	1.676
RF55_1441	GB47599	cytoplasmic dynein 1 light intermediate chain 1	-1.365	2.346
XLOC_001241			-1.913	1.776
RF55_404	GB53011	polyphosphoinositide phosphatase	-2.127	1.562
RF55_11430	XP_016769030.1		-2.092	1.569
RF55_1609	GB40975	gamma-aminobutyric acid receptor subunit beta	-1.933	1.669
RF55_5498	GB55971	palmitoyltransferase ZDHHC9-like	-1.814	1.696
RF55_13282	GB54319	synaptotagmin 20	-1.820	1.656
RF55_4134	XP_016771187.1		-1.634	1.784
RF55_3869	GB51413	C3 and PZP-like alpha-2-macroglobulin domain-containing protein 8-like	-1.835	1.564
RF55_5507	XP_016772718.1		-1.550	1.756
RF55_3824	XP_016768669.1		-1.525	1.609
XLOC_005436			-1.759	1.364

(continued)

Gene	Amel ortholog	Name	Lowest FC	Highest FC
RF55_10519	GB54446	arginine kinase	-2.965	0.146
RF55_310	100576851	ornithine decarboxylase antizyme 1-like	-2.171	0.484
RF55_3137	GB54056	serine hydroxymethyltransferase, cytosolic	-0.504	0.306
RF55_6300	XP_016767697.1		-0.194	0.398
RF55_4024	XP_016767817.1		-0.296	0.295

Supplementary Table 14: The posterior model probabilities of five competing multivariate Bayesian models of the module eigengene dataset. The best-fitting model (with posterior probability of almost 1) contains the treatment effect only (not the species effect, or the treatment-by-species interaction).

	Posterior model probability
Treatment x Species	0
Treatment + Species	0
Treatment	1
Species	0
Intercept only	0

Supplementary Table 15: Full summary of the best-fitting multivariate Bayesian model of the eigengene data for all nine modules, implemented in the programming language `Stan` via the R package `brms`. The most salient part of the output is the population-level effects (often called fixed effects), which give the coefficients for the intercept and the effect of queen pheromone treatment on the eigengenes for each module. The 9 response variables were all scaled to have mean 0 and variance 1 before running the model, meaning that the estimates can be interpreted as Cohen's d effect size. The remaining sections describe the (co)variance associated with colony (which appears to be low), and the covariance in the residuals (which illustrates how eigengenes are correlated across modules).

```
##  Family: MV(gaussian, gaussian, gaussian, gaussian, gaussian, gaussian, gaussian, gaussian, gaussian)
##   Links: mu = identity; sigma = identity
##          mu = identity; sigma = identity
##          mu = identity; sigma = identity
##          mu = identity; sigma = identity
##          mu = identity; sigma = identity
##          mu = identity; sigma = identity
##          mu = identity; sigma = identity
##          mu = identity; sigma = identity
##          mu = identity; sigma = identity
## Formula: m1 ~ Treatment + (1 | p | colony)
##          m2 ~ Treatment + (1 | p | colony)
##          m3 ~ Treatment + (1 | p | colony)
##          m4 ~ Treatment + (1 | p | colony)
##          m5 ~ Treatment + (1 | p | colony)
##          m6 ~ Treatment + (1 | p | colony)
##          m7 ~ Treatment + (1 | p | colony)
##          m8 ~ Treatment + (1 | p | colony)
##          m9 ~ Treatment + (1 | p | colony)
##    Data: eigen.data %>% mutate(Module = gsub("Module ", "m" (Number of observations: 39)
## Samples: 4 chains, each with iter = 5000; warmup = 2500; thin = 1;
##          total post-warmup samples = 10000
##
## Group-Level Effects:
## ~colony (Number of levels: 27)
##                                Estimate Est.Error l-95% CI u-95% CI Eff.Sample Rhat
## sd(m1_Intercept)                   0.08      0.06     0.00     0.21       4356 1.00
## sd(m2_Intercept)                   0.04      0.03     0.00     0.12       7092 1.00
## sd(m3_Intercept)                   0.04      0.03     0.00     0.13      10000 1.00
## sd(m4_Intercept)                   0.04      0.03     0.00     0.13       8519 1.00
## sd(m5_Intercept)                   0.05      0.03     0.00     0.12       4317 1.00
## sd(m6_Intercept)                   0.05      0.04     0.00     0.14       4690 1.00
## sd(m7_Intercept)                   0.06      0.04     0.00     0.17      10000 1.00
## sd(m8_Intercept)                   0.13      0.09     0.01     0.34       6666 1.00
## sd(m9_Intercept)                   0.04      0.03     0.00     0.12       5685 1.00
## cor(m1_Intercept,m2_Intercept)     0.02      0.32    -0.58     0.60      10000 1.00
## cor(m1_Intercept,m3_Intercept)     0.01      0.32    -0.60     0.61      10000 1.00
## cor(m2_Intercept,m3_Intercept)    -0.01      0.32    -0.62     0.60      10000 1.00
## cor(m1_Intercept,m4_Intercept)    -0.00      0.32    -0.62     0.61      10000 1.00
## cor(m2_Intercept,m4_Intercept)     0.05      0.32    -0.57     0.64      10000 1.00
## cor(m3_Intercept,m4_Intercept)    -0.02      0.32    -0.62     0.60      10000 1.00
## cor(m1_Intercept,m5_Intercept)     0.02      0.32    -0.58     0.61      10000 1.00
## cor(m2_Intercept,m5_Intercept)     0.00      0.32    -0.60     0.59      10000 1.00
## cor(m3_Intercept,m5_Intercept)    -0.02      0.32    -0.63     0.59      10000 1.00
## cor(m4_Intercept,m5_Intercept)     0.02      0.32    -0.58     0.62      10000 1.00
```

```
##  cor(m1_Intercept,m6_Intercept)     0.00      0.31    -0.59     0.60       10000 1.00
##  cor(m2_Intercept,m6_Intercept)    -0.02      0.32    -0.61     0.59       10000 1.00
##  cor(m3_Intercept,m6_Intercept)     0.05      0.32    -0.58     0.64       10000 1.00
##  cor(m4_Intercept,m6_Intercept)     0.03      0.31    -0.57     0.63       10000 1.00
##  cor(m5_Intercept,m6_Intercept)     0.03      0.32    -0.58     0.63       10000 1.00
##  cor(m1_Intercept,m7_Intercept)    -0.01      0.32    -0.61     0.59       10000 1.00
##  cor(m2_Intercept,m7_Intercept)     0.02      0.32    -0.59     0.62       10000 1.00
##  cor(m3_Intercept,m7_Intercept)     0.06      0.32    -0.57     0.65       10000 1.00
##  cor(m4_Intercept,m7_Intercept)     0.02      0.32    -0.58     0.62       10000 1.00
##  cor(m5_Intercept,m7_Intercept)    -0.01      0.32    -0.61     0.59       10000 1.00
##  cor(m6_Intercept,m7_Intercept)    -0.02      0.32    -0.63     0.59       10000 1.00
##  cor(m1_Intercept,m8_Intercept)     0.01      0.31    -0.59     0.61       10000 1.00
##  cor(m2_Intercept,m8_Intercept)    -0.01      0.31    -0.60     0.58       10000 1.00
##  cor(m3_Intercept,m8_Intercept)     0.04      0.32    -0.57     0.64       10000 1.00
##  cor(m4_Intercept,m8_Intercept)     0.02      0.31    -0.58     0.61       10000 1.00
##  cor(m5_Intercept,m8_Intercept)    -0.00      0.32    -0.60     0.60       10000 1.00
##  cor(m6_Intercept,m8_Intercept)    -0.04      0.31    -0.62     0.57        7970 1.00
##  cor(m7_Intercept,m8_Intercept)    -0.04      0.32    -0.62     0.58        6458 1.00
##  cor(m1_Intercept,m9_Intercept)    -0.01      0.32    -0.62     0.60       10000 1.00
##  cor(m2_Intercept,m9_Intercept)     0.05      0.33    -0.59     0.65       10000 1.00
##  cor(m3_Intercept,m9_Intercept)    -0.00      0.32    -0.61     0.60       10000 1.00
##  cor(m4_Intercept,m9_Intercept)    -0.05      0.32    -0.64     0.57       10000 1.00
##  cor(m5_Intercept,m9_Intercept)     0.04      0.31    -0.57     0.62       10000 1.00
##  cor(m6_Intercept,m9_Intercept)     0.02      0.31    -0.59     0.62        8255 1.00
##  cor(m7_Intercept,m9_Intercept)     0.01      0.32    -0.60     0.61        6350 1.00
##  cor(m8_Intercept,m9_Intercept)     0.03      0.32    -0.58     0.63        6231 1.00
##
##  Population-Level Effects:
##                 Estimate Est.Error l-95% CI u-95% CI Eff.Sample Rhat
##  m1_Intercept      -0.27      0.18    -0.64     0.09       4403 1.00
##  m2_Intercept      -0.23      0.19    -0.60     0.14       5015 1.00
##  m3_Intercept       0.23      0.19    -0.16     0.60       5448 1.00
##  m4_Intercept      -0.63      0.15    -0.92    -0.32       5864 1.00
##  m5_Intercept      -0.18      0.18    -0.53     0.19       4295 1.00
##  m6_Intercept       0.03      0.20    -0.36     0.43       5752 1.00
##  m7_Intercept      -0.05      0.20    -0.46     0.34       5803 1.00
##  m8_Intercept       0.13      0.21    -0.28     0.54       5686 1.00
##  m9_Intercept       0.32      0.17    -0.01     0.67       4355 1.00
##  m1_TreatmentQP     0.56      0.26     0.04     1.06       4299 1.00
##  m2_TreatmentQP     0.48      0.27    -0.06     1.00       4542 1.00
##  m3_TreatmentQP    -0.47      0.28    -1.01     0.10       5341 1.00
##  m4_TreatmentQP     1.28      0.22     0.85     1.71       5443 1.00
##  m5_TreatmentQP     0.37      0.26    -0.15     0.86       4059 1.00
##  m6_TreatmentQP    -0.07      0.29    -0.65     0.50       5358 1.00
##  m7_TreatmentQP     0.11      0.30    -0.47     0.69       5655 1.00
##  m8_TreatmentQP    -0.27      0.29    -0.85     0.32       5407 1.00
##  m9_TreatmentQP    -0.66      0.25    -1.16    -0.17       4120 1.00
##
##  Family Specific Parameters:
##           Estimate Est.Error l-95% CI u-95% CI Eff.Sample Rhat
##  sigma_m1     0.82      0.08     0.69     0.98      10000 1.00
##  sigma_m2     0.84      0.08     0.70     1.01       7447 1.00
##  sigma_m3     0.87      0.09     0.72     1.05       7227 1.00
##  sigma_m4     0.70      0.07     0.58     0.85      10000 1.00
```

```
## sigma_m5     0.82      0.07     0.69     0.97       5832 1.00
## sigma_m6     0.90      0.09     0.75     1.10       6538 1.00
## sigma_m7     0.92      0.09     0.76     1.12      10000 1.00
## sigma_m8     0.91      0.10     0.75     1.12      10000 1.00
## sigma_m9     0.79      0.07     0.66     0.94       6247 1.00
##
## Residual Correlations:
##               Estimate Est.Error l-95% CI u-95% CI Eff.Sample Rhat
## rescor(m1,m2)     0.56      0.10     0.35     0.73       6438 1.00
## rescor(m1,m3)     0.69      0.08     0.51     0.82       7120 1.00
## rescor(m2,m3)     0.25      0.12    -0.00     0.48      10000 1.00
## rescor(m1,m4)     0.53      0.10     0.31     0.71       8099 1.00
## rescor(m2,m4)     0.73      0.07     0.56     0.84      10000 1.00
## rescor(m3,m4)     0.10      0.13    -0.17     0.35      10000 1.00
## rescor(m1,m5)     0.59      0.10     0.38     0.76       5667 1.00
## rescor(m2,m5)     0.66      0.08     0.48     0.79       5941 1.00
## rescor(m3,m5)     0.42      0.11     0.19     0.61       8145 1.00
## rescor(m4,m5)     0.71      0.07     0.55     0.83       7301 1.00
## rescor(m1,m6)     0.65      0.09     0.45     0.79       6974 1.00
## rescor(m2,m6)     0.19      0.13    -0.07     0.43      10000 1.00
## rescor(m3,m6)     0.64      0.08     0.46     0.78       8022 1.00
## rescor(m4,m6)     0.32      0.12     0.06     0.54       8027 1.00
## rescor(m5,m6)     0.78      0.06     0.65     0.87      10000 1.00
## rescor(m1,m7)     0.59      0.10     0.36     0.75      10000 1.00
## rescor(m2,m7)     0.59      0.09     0.39     0.74      10000 1.00
## rescor(m3,m7)     0.71      0.07     0.54     0.83      10000 1.00
## rescor(m4,m7)     0.32      0.12     0.06     0.55      10000 1.00
## rescor(m5,m7)     0.35      0.12     0.11     0.56       8081 1.00
## rescor(m6,m7)     0.21      0.13    -0.07     0.45       8440 1.00
## rescor(m1,m8)     0.53      0.11     0.29     0.71      10000 1.00
## rescor(m2,m8)     0.53      0.10     0.30     0.71      10000 1.00
## rescor(m3,m8)     0.55      0.10     0.34     0.72      10000 1.00
## rescor(m4,m8)     0.46      0.11     0.22     0.66      10000 1.00
## rescor(m5,m8)     0.49      0.11     0.26     0.68      10000 1.00
## rescor(m6,m8)     0.39      0.12     0.13     0.60      10000 1.00
## rescor(m7,m8)     0.44      0.12     0.18     0.65      10000 1.00
## rescor(m1,m9)     0.57      0.10     0.35     0.74       5328 1.00
## rescor(m2,m9)     0.77      0.06     0.64     0.87       7273 1.00
## rescor(m3,m9)     0.54      0.10     0.33     0.70      10000 1.00
## rescor(m4,m9)     0.45      0.11     0.21     0.65      10000 1.00
## rescor(m5,m9)     0.83      0.05     0.71     0.90      10000 1.00
## rescor(m6,m9)     0.60      0.09     0.39     0.75      10000 1.00
## rescor(m7,m9)     0.57      0.10     0.35     0.73      10000 1.00
## rescor(m8,m9)     0.52      0.11     0.28     0.70      10000 1.00
##
## Samples were drawn using sampling(NUTS). For each parameter, Eff.Sample
## is a crude measure of effective sample size, and Rhat is the potential
## scale reduction factor on split chains (at convergence, Rhat = 1).
```

Supplementary Table 16: Results of Spearman's rank correlations testing for a relationship between the effect of queen pheromone on gene expression, and the connectedness of the gene. Negative values of Spearman's Rho mean that highly pheromone-sensitive genes tend to have lower connectedness.

Species	rho	p
Apis mellifera	-0.2462854	0
Bombus terrestris	-0.2988244	0
Lasius flavus	-0.4151465	0
Lasius niger	-0.3158513	0

Supplementary Table 17: A list of the six module pairs, from Morandin *et al.* 2016 and the present study, which had significantly more genes in common than expected by chance. The p-values were calculated by running hypergeometric tests on all possible pairs of modules from the two studies, and then adjusting all the p-values using the Benjamini-Hochberg procedure.

Morandin module	Holman module	n overlapping genes	Size of Morandin module	Size of Holman module	p-value	Caste bias of Morandin module
Module 32	Module 2	20	39	363	0.0000	Worker-biased
Module 31	Module 1	69	161	969	0.0003	Queen-biased
Module 26	Module 8	5	49	24	0.0005	Worker-biased
Module 32	Module 8	4	39	24	0.0033	Worker-biased
Module 13	Module 4	10	61	177	0.0155	Queen-biased
Module 10	Module 3	10	77	150	0.0232	Worker-biased

Supplementary Table 18: The results of GSEA (gene set enrichment analysis) for pheromone sensitivity in gene expression in *Apis mellifera*. The table lists GO and KEGG terms with their NES (normalized enrichment score), the associated raw and adjusted p-values (adjustment was performed using Benjamini-Hochberg correction), and the genes underlying the enrichment result.

Test_type	ID	pvalue	p.adjust	NES	Description
GO: Biological process	GO:0006368	0.001	0.032	-2.020	transcription elongation from RNA polymerase II promoter
GO: Biological process	GO:0015986	0.006	0.077	-1.738	ATP synthesis coupled proton transport
GO: Biological process	GO:0016570	0.017	0.098	-1.653	histone modification
GO: Biological process	GO:0042742	0.033	0.134	1.461	defense response to bacterium
GO: Biological process	GO:0016579	0.045	0.139	-1.457	protein deubiquitination
GO: Cellular component	GO:0005576	0.000	0.012	1.535	extracellular region
GO: Cellular component	GO:0005667	0.036	0.134	-1.536	transcription factor complex
GO: Cellular component	GO:0005743	0.038	0.134	-1.637	mitochondrial inner membrane
GO: Cellular component	GO:0005680	0.044	0.139	-1.465	anaphase-promoting complex
GO: Cellular component	GO:0005886	0.049	0.144	1.232	plasma membrane
GO: Molecular function	GO:0008137	0.001	0.032	-2.130	NADH dehydrogenase (ubiquinone) activity
GO: Molecular function	GO:0005549	0.002	0.042	1.433	odorant binding
GO: Molecular function	GO:0004984	0.005	0.069	1.460	olfactory receptor activity
GO: Molecular function	GO:0004722	0.010	0.087	-1.668	protein serine/threonine phosphatase activity
GO: Molecular function	GO:0005319	0.012	0.092	1.537	lipid transporter activity
GO: Molecular function	GO:0004252	0.020	0.113	1.365	serine-type endopeptidase activity
GO: Molecular function	GO:0046933	0.023	0.121	-1.588	proton-transporting ATP synthase activity, rotational mechanism
GO: Molecular function	GO:0016614	0.027	0.125	1.440	oxidoreductase activity, acting on CH-OH group of donors
KEGG	KEGG:04711	0.037	0.134	-1.478	Circadian rhythm - fly

Supplementary Table 19: The results of GSEA (gene set enrichment analysis) for pheromone sensitivity in gene expression in *Bombus terrestris*. The table lists GO and KEGG terms with their NES (normalized enrichment score), the associated raw and adjusted p-values (adjustment was performed using Benjamini-Hochberg correction), and the genes underlying the enrichment result.

Test_type	ID	pvalue	p.adjust	NES	Description
GO: Biological process	GO:0006030	0.001	0.032	1.684	chitin metabolic process
GO: Biological process	GO:0045087	0.012	0.092	1.589	innate immune response
GO: Biological process	GO:0000398	0.036	0.134	-1.381	mRNA splicing, via spliceosome
GO: Biological process	GO:0015991	0.042	0.137	-1.376	ATP hydrolysis coupled proton transport
GO: Biological process	GO:0042742	0.044	0.139	1.476	defense response to bacterium
GO: Cellular component	GO:0005576	0.000	0.012	1.650	extracellular region
GO: Cellular component	GO:0005886	0.003	0.045	1.545	plasma membrane
GO: Cellular component	GO:0005694	0.026	0.125	1.511	chromosome
GO: Molecular function	GO:0042302	0.000	0.025	1.737	structural constituent of cuticle
GO: Molecular function	GO:0004984	0.001	0.032	1.703	olfactory receptor activity
GO: Molecular function	GO:0005549	0.001	0.032	1.678	odorant binding
GO: Molecular function	GO:0008061	0.001	0.032	1.678	chitin binding
GO: Molecular function	GO:0102336	0.010	0.086	1.606	3-oxo-arachidoyl-CoA synthase activity
GO: Molecular function	GO:0102337	0.010	0.086	1.606	3-oxo-cerotoyl-CoA synthase activity
GO: Molecular function	GO:0102338	0.010	0.086	1.606	3-oxo-lignoceronyl-CoA synthase activity
GO: Molecular function	GO:0102756	0.010	0.086	1.606	very-long-chain 3-ketoacyl-CoA synthase activity
GO: Molecular function	GO:0016614	0.023	0.121	1.527	oxidoreductase activity, acting on CH-OH group of donors
GO: Molecular function	GO:0043565	0.030	0.134	1.305	sequence-specific DNA binding
GO: Molecular function	GO:0004252	0.045	0.139	1.395	serine-type endopeptidase activity
GO: Molecular function	GO:0004888	0.047	0.140	1.460	transmembrane signaling receptor activity
KEGG	KEGG:00900	0.003	0.046	1.667	Terpenoid backbone biosynthesis
KEGG	KEGG:00910	0.015	0.098	1.563	Nitrogen metabolism
KEGG	KEGG:03022	0.020	0.113	-1.375	Basal transcription factors
KEGG	KEGG:00360	0.033	0.134	1.499	Phenylalanine metabolism

Supplementary Table 20: The results of GSEA (gene set enrichment analysis) for pheromone sensitivity in gene expression in *Lasius flavus*. The table lists GO and KEGG terms with their NES (normalized enrichment score), the associated raw and adjusted p-values (adjustment was performed using Benjamini-Hochberg correction), and the genes underlying the enrichment result.

Test_type	ID	pvalue	p.adjust	NES	Description
GO: Biological process	GO:0006464	0.008	0.083	1.603	cellular protein modification process
GO: Biological process	GO:0006364	0.014	0.098	1.598	rRNA processing
GO: Biological process	GO:0042742	0.015	0.098	1.552	defense response to bacterium
GO: Biological process	GO:0006633	0.016	0.098	1.582	fatty acid biosynthetic process
GO: Biological process	GO:0007020	0.025	0.124	1.512	microtubule nucleation
GO: Cellular component	GO:0005730	0.002	0.042	1.786	nucleolus
GO: Cellular component	GO:0035267	0.007	0.079	1.630	NuA4 histone acetyltransferase complex
GO: Cellular component	GO:0032040	0.014	0.098	1.577	small-subunit processome
GO: Cellular component	GO:0005886	0.022	0.120	1.506	plasma membrane
GO: Cellular component	GO:0005856	0.024	0.124	1.514	cytoskeleton
GO: Cellular component	GO:0031011	0.036	0.134	1.479	Ino80 complex
GO: Molecular function	GO:0004984	0.013	0.092	1.441	olfactory receptor activity
GO: Molecular function	GO:0003779	0.016	0.098	1.543	actin binding
GO: Molecular function	GO:0005549	0.018	0.103	1.562	odorant binding
GO: Molecular function	GO:0042302	0.029	0.132	1.517	structural constituent of cuticle
GO: Molecular function	GO:0036459	0.032	0.134	-1.577	thiol-dependent ubiquitinyl hydrolase activity
GO: Molecular function	GO:0005319	0.034	0.134	1.495	lipid transporter activity
GO: Molecular function	GO:0102336	0.040	0.134	1.479	3-oxo-arachidoyl-CoA synthase activity
GO: Molecular function	GO:0102337	0.040	0.134	1.479	3-oxo-cerotoyl-CoA synthase activity
GO: Molecular function	GO:0102338	0.040	0.134	1.479	3-oxo-lignoceronyl-CoA synthase activity
GO: Molecular function	GO:0102756	0.040	0.134	1.479	very-long-chain 3-ketoacyl-CoA synthase activity
KEGG	KEGG:00130	0.006	0.077	1.633	Ubiquinone and other terpenoid-quinone biosynthesis
KEGG	KEGG:04080	0.012	0.092	1.560	Neuroactive ligand-receptor interaction
KEGG	KEGG:00061	0.037	0.134	1.479	Fatty acid biosynthesis
KEGG	KEGG:00640	0.038	0.134	1.428	Propanoate metabolism
KEGG	KEGG:00790	0.038	0.134	1.466	Folate biosynthesis
KEGG	KEGG:00630	0.050	0.144	1.413	Glyoxylate and dicarboxylate metabolism

Supplementary Table 21: The results of GSEA (gene set enrichment analysis) for pheromone sensitivity in gene expression in *Lasius niger*. The table lists GO and KEGG terms with their NES (normalized enrichment score), the associated raw and adjusted p-values (adjustment was performed using Benjamini-Hochberg correction), and the genes underlying the enrichment result.

Test_type	ID	pvalue	p.adjust	NES	Description
GO: Biological process	GO:0030163	0.002	0.042	-1.884	protein catabolic process
GO: Biological process	GO:0009058	0.040	0.134	1.449	biosynthetic process
GO: Cellular component	GO:0000139	0.003	0.050	1.691	Golgi membrane
GO: Cellular component	GO:0005576	0.041	0.137	1.321	extracellular region
GO: Cellular component	GO:0005680	0.042	0.137	-1.494	anaphase-promoting complex
GO: Molecular function	GO:0036402	0.001	0.035	-1.880	proteasome-activating ATPase activity
GO: Molecular function	GO:0008146	0.006	0.077	1.608	sulfotransferase activity
GO: Molecular function	GO:0042302	0.007	0.081	1.587	structural constituent of cuticle
GO: Molecular function	GO:0005198	0.010	0.086	1.588	structural molecule activity
GO: Molecular function	GO:0005509	0.025	0.124	1.349	calcium ion binding
GO: Molecular function	GO:0036459	0.031	0.134	-1.532	thiol-dependent ubiquitinyl hydrolase activity
GO: Molecular function	GO:0008536	0.046	0.139	-1.483	Ran GTPase binding
KEGG	KEGG:01040	0.002	0.042	1.692	Biosynthesis of unsaturated fatty acids
KEGG	KEGG:03050	0.013	0.092	1.475	Proteasome
KEGG	KEGG:04080	0.027	0.125	1.424	Neuroactive ligand-receptor interaction
KEGG	KEGG:00260	0.047	0.140	1.401	Glycine, serine and threonine metabolism

Supplementary Table 22: The Supplementary Table hows the Spearman correlation (rho) and p-value for correlations across genes in the pheromone-sensitivity of their isoform production, for each pair of species. For each gene, our metric of the sensitivity of splicing to pheromone treatment was calculated by taking the difference between the highest and lowest log fold change values for the various isoforms. Thus, genes for which one isoform strongly increased in expression and one strongly decreased following pheromone treatment score high, and those in which there is no response to pheromone – or a consistent response for all isoforms – score low. The results suggest that the pheromone sensitivity in splicing is highly conserved between orthologous bee genes, and somewhat less conserved between orthologous ant genes, and between bee and ants genes.

Species1	Species2	rho	p	p.adjust	sig
Apis mellifera	Bombus terrestris	0.185	0.000	0.000	*
Apis mellifera	Lasius flavus	0.063	0.068	0.137	
Apis mellifera	Lasius niger	0.043	0.153	0.184	
Bombus terrestris	Lasius flavus	0.089	0.010	0.030	*
Bombus terrestris	Lasius niger	0.030	0.294	0.294	
Lasius flavus	Lasius niger	0.041	0.150	0.184	

Supplementary Table 23: The results of GSEA (gene set enrichment analysis) for pheromone sensitivity in alternative splicing. The table lists statistically significant GO and KEGG terms with their NES (normalized enrichment score), the associated raw and adjusted p-values (adjustment was performed using Benjamini-Hochberg correction), and the genes underlying each enrichment result. Note that the online version of this table at https://mikheyev.github.io/queen-pheromone/ has additional columns giving the names and IDs of the enriched genes, which did not fit on this page.

Test_type	ID	pvalue	p.adjust	NES	Description	Species
GO: Cellular component	GO:0005789	0.037	0.139	1.450	endoplasmic reticulum membrane	am
GO: Cellular component	GO:0016592	0.038	0.139	1.452	mediator complex	am
GO: Molecular function	GO:0001104	0.032	0.127	1.456	RNA polymerase II transcription cofactor activity	am
KEGG	KEGG:04144	0.004	0.073	1.432	Endocytosis	am
KEGG	KEGG:04745	0.007	0.087	1.597	Phototransduction - fly	am
KEGG	KEGG:00630	0.012	0.098	1.553	Glyoxylate and dicarboxylate metabolism	am
KEGG	KEGG:04310	0.023	0.115	1.393	Wnt signaling pathway	am
KEGG	KEGG:00250	0.025	0.115	1.478	Alanine, aspartate and glutamate metabolism	am
KEGG	KEGG:00760	0.039	0.139	1.448	Nicotinate and nicotinamide metabolism	am
GO: Biological process	GO:0007034	0.021	0.115	-1.584	vacuolar transport	bt
GO: Biological process	GO:0006351	0.045	0.151	1.363	transcription, DNA-templated	bt
GO: Biological process	GO:0006412	0.048	0.151	-1.429	translation	bt
GO: Cellular component	GO:0005886	0.003	0.073	1.538	plasma membrane	bt
GO: Cellular component	GO:0005576	0.011	0.098	1.563	extracellular region	bt
GO: Molecular function	GO:0046983	0.011	0.098	1.535	protein dimerization activity	bt
GO: Molecular function	GO:0003735	0.023	0.115	-1.445	structural constituent of ribosome	bt
GO: Molecular function	GO:0004672	0.027	0.115	1.454	protein kinase activity	bt
GO: Molecular function	GO:0004252	0.047	0.151	1.397	serine-type endopeptidase activity	bt
KEGG	KEGG:00534	0.005	0.073	1.596	Glycosaminoglycan biosynthesis - heparan sulfate / heparin	bt
KEGG	KEGG:04214	0.018	0.115	1.472	Apoptosis - fly	bt
KEGG	KEGG:04350	0.021	0.115	1.489	TGF-beta signaling pathway	bt
KEGG	KEGG:03010	0.037	0.139	-1.341	Ribosome	bt
GO: Molecular function	GO:0030170	0.005	0.073	1.642	pyridoxal phosphate binding	lf
GO: Molecular function	GO:0004252	0.008	0.089	1.593	serine-type endopeptidase activity	lf
KEGG	KEGG:00190	0.002	0.073	1.682	Oxidative phosphorylation	lf
KEGG	KEGG:00860	0.003	0.073	1.682	Porphyrin and chlorophyll metabolism	lf
KEGG	KEGG:03010	0.011	0.098	-1.828	Ribosome	lf
KEGG	KEGG:04624	0.027	0.115	1.460	Toll and Imd signaling pathway	lf
KEGG	KEGG:01100	0.047	0.151	1.163	Metabolic pathways	lf
GO: Biological process	GO:0007034	0.024	0.115	-1.628	vacuolar transport	ln
GO: Biological process	GO:0005975	0.029	0.117	1.386	carbohydrate metabolic process	ln
GO: Cellular component	GO:0005794	0.023	0.115	-1.588	Golgi apparatus	ln
GO: Cellular component	GO:0005886	0.024	0.115	1.511	plasma membrane	ln
GO: Molecular function	GO:0030170	0.019	0.115	1.479	pyridoxal phosphate binding	ln
GO: Molecular function	GO:0009055	0.019	0.115	-1.590	electron transfer activity	ln
GO: Molecular function	GO:0005198	0.027	0.115	1.488	structural molecule activity	ln
GO: Molecular function	GO:0003924	0.047	0.151	1.290	GTPase activity	ln
KEGG	KEGG:01230	0.000	0.022	1.672	Biosynthesis of amino acids	ln
KEGG	KEGG:01200	0.000	0.022	1.597	Carbon metabolism	ln
KEGG	KEGG:00030	0.001	0.061	1.712	Pentose phosphate pathway	ln
KEGG	KEGG:00260	0.004	0.073	1.639	Glycine, serine and threonine metabolism	ln
KEGG	KEGG:00040	0.006	0.082	1.612	Pentose and glucuronate interconversions	ln
KEGG	KEGG:04140	0.011	0.098	1.393	Autophagy - animal	ln
KEGG	KEGG:01100	0.015	0.115	1.156	Metabolic pathways	ln
KEGG	KEGG:00051	0.019	0.115	1.520	Fructose and mannose metabolism	ln
KEGG	KEGG:00630	0.025	0.115	1.455	Glyoxylate and dicarboxylate metabolism	ln

Supplementary Table 24: List of every significant enrichment test result for each module, for all four ontologies. The latter two columns specify all the genes associated with the focal GO or KEGG term that are found in the module. The GeneRatio and BgRatio columns give the number of genes annotated with the focal term that are present in the focal module or the gene universe, respecitvely. These values were used to calculate the enrichment column, as the proportion of genes associated with the focal annotation term in the module, divided by the equivalent proportion in the gene universe. Note that the online version of this table at https://mikheyev.github.io/queen-pheromone/ has additional columns giving the names and IDs of the enriched genes, and some extra enrichment test statistics, which did not fit on this page.

Module	Test_type	ID	Description	enrichment	pvalue	p.adjust
Module 1	GO:CC	GO:0044428	nuclear part	1.518	0.000	0.000
Module 1	GO:MF	GO:0003676	nucleic acid binding	1.220	0.000	0.001
Module 1	GO:CC	GO:0043231	intracellular membrane-bounded organelle	1.205	0.000	0.001
Module 1	KEGG	KEGG:03440	Homologous recombination	2.020	0.000	0.013
Module 1	GO:BP	GO:0043170	macromolecule metabolic process	1.132	0.000	0.023
Module 1	GO:CC	GO:0043229	intracellular organelle	1.143	0.001	0.006
Module 1	GO:CC	GO:1990234	transferase complex	1.567	0.001	0.006
Module 1	GO:BP	GO:0006139	nucleobase-containing compound metabolic process	1.164	0.001	0.027
Module 1	GO:CC	GO:0005681	spliceosomal complex	2.376	0.001	0.007
Module 1	KEGG	KEGG:03460	Fanconi anemia pathway	1.994	0.001	0.026
Module 1	GO:BP	GO:0046483	heterocycle metabolic process	1.153	0.001	0.027
Module 1	GO:BP	GO:1901360	organic cyclic compound metabolic process	1.147	0.002	0.027
Module 1	GO:BP	GO:0006725	cellular aromatic compound metabolic process	1.145	0.002	0.027
Module 1	GO:BP	GO:0033554	cellular response to stress	1.538	0.003	0.027
Module 1	GO:CC	GO:0043233	organelle lumen	1.370	0.003	0.015
Module 1	GO:CC	GO:0070013	intracellular organelle lumen	1.370	0.003	0.015
Module 1	KEGG	KEGG:03420	Nucleotide excision repair	1.840	0.005	0.067
Module 1	GO:BP	GO:0006325	chromatin organization	1.602	0.005	0.043
Module 1	GO:MF	GO:0101005	ubiquitinyl hydrolase activity	1.789	0.006	0.073
Module 1	GO:MF	GO:0019899	enzyme binding	1.574	0.006	0.073
Module 1	GO:MF	GO:0008276	protein methyltransferase activity	2.236	0.008	0.073
Module 1	KEGG	KEGG:03040	Spliceosome	1.402	0.008	0.087
Module 1	GO:MF	GO:0005524	ATP binding	1.174	0.010	0.077
Module 1	GO:CC	GO:0005667	transcription factor complex	1.827	0.011	0.046
Module 1	GO:BP	GO:0022402	cell cycle process	1.659	0.012	0.091
Module 1	GO:CC	GO:0033202	DNA helicase complex	2.376	0.013	0.046
Module 1	GO:CC	GO:0044446	intracellular organelle part	1.161	0.013	0.046
Module 1	KEGG	KEGG:03430	Mismatch repair	1.903	0.020	0.167
Module 1	GO:MF	GO:0060589	nucleoside-triphosphatase regulator activity	1.640	0.024	0.145
Module 1	GO:MF	GO:0005543	phospholipid binding	1.579	0.028	0.147
Module 1	GO:CC	GO:0044427	chromosomal part	1.544	0.032	0.101
Module 1	GO:MF	GO:0016772	transferase activity, transferring phosphorus-containing groups	1.204	0.033	0.152
Module 1	GO:BP	GO:0006996	organelle organization	1.214	0.033	0.208
Module 1	GO:BP	GO:0000278	mitotic cell cycle	1.595	0.034	0.208
Module 1	GO:CC	GO:0044798	nuclear transcription factor complex	1.728	0.040	0.116
Module 1	GO:MF	GO:0016810	hydrolase activity, acting on carbon-nitrogen (but not peptide) bonds	1.597	0.040	0.164
Module 1	GO:BP	GO:1903047	mitotic cell cycle process	1.659	0.042	0.236
Module 1	GO:MF	GO:0008047	enzyme activator activity	1.538	0.045	0.168
Module 1	GO:CC	GO:0030880	RNA polymerase complex	1.584	0.048	0.128
Module 1	KEGG	KEGG:00310	Lysine degradation	1.554	0.049	0.333
Module 2	KEGG	KEGG:03010	Ribosome	3.646	0.000	0.000
Module 2	GO:CC	GO:0005840	ribosome	3.669	0.000	0.000
Module 2	GO:CC	GO:0005737	cytoplasm	1.648	0.000	0.000
Module 2	GO:BP	GO:0019538	protein metabolic process	1.745	0.000	0.000
Module 2	GO:CC	GO:0043232	intracellular non-membrane-bounded organelle	2.059	0.000	0.000
Module 2	GO:CC	GO:0044444	cytoplasmic part	1.679	0.000	0.000

(continued)

Module	Test_type	ID	Description	enrichment	pvalue	p.adjust
Module 2	GO:BP	GO:1901564	organonitrogen compound metabolic process	1.495	0.000	0.000
Module 2	GO:CC	GO:0044391	ribosomal subunit	3.447	0.000	0.001
Module 2	GO:BP	GO:0044260	cellular macromolecule metabolic process	1.321	0.000	0.005
Module 2	GO:CC	GO:0005839	proteasome core complex	3.575	0.002	0.011
Module 2	GO:CC	GO:1905368	peptidase complex	2.523	0.007	0.032
Module 2	KEGG	KEGG:00590	Arachidonic acid metabolism	3.913	0.007	0.117
Module 2	GO:CC	GO:0000502	proteasome complex	2.925	0.008	0.034
Module 2	KEGG	KEGG:03050	Proteasome	2.069	0.009	0.117
Module 2	GO:BP	GO:1901576	organic substance biosynthetic process	1.231	0.011	0.111
Module 2	GO:BP	GO:0044249	cellular biosynthetic process	1.232	0.012	0.111
Module 2	GO:MF	GO:0051082	unfolded protein binding	2.662	0.014	0.167
Module 2	KEGG	KEGG:00051	Fructose and mannose metabolism	3.261	0.018	0.185
Module 2	GO:CC	GO:0043229	intracellular organelle	1.152	0.029	0.111
Module 2	KEGG	KEGG:04150	mTOR signaling pathway	1.726	0.030	0.245
Module 2	GO:BP	GO:0043170	macromolecule metabolic process	1.142	0.030	0.210
Module 2	GO:BP	GO:0044248	cellular catabolic process	1.484	0.032	0.210
Module 2	GO:CC	GO:0030117	membrane coat	2.437	0.038	0.116
Module 2	GO:CC	GO:0005615	extracellular space	2.681	0.044	0.116
Module 2	GO:CC	GO:0012506	vesicle membrane	3.217	0.048	0.116
Module 2	GO:CC	GO:0030660	Golgi-associated vesicle membrane	3.217	0.048	0.116
Module 2	GO:CC	GO:0030662	coated vesicle membrane	3.217	0.048	0.116
Module 3	GO:MF	GO:0038023	signaling receptor activity	5.009	0.000	0.000
Module 3	GO:MF	GO:0022803	passive transmembrane transporter activity	7.557	0.000	0.000
Module 3	GO:CC	GO:0098794	postsynapse	10.144	0.000	0.000
Module 3	GO:CC	GO:0071944	cell periphery	4.254	0.000	0.000
Module 3	GO:CC	GO:0005886	plasma membrane	4.463	0.000	0.000
Module 3	GO:CC	GO:0045211	postsynaptic membrane	10.144	0.000	0.000
Module 3	GO:CC	GO:0097060	synaptic membrane	10.144	0.000	0.000
Module 3	GO:CC	GO:0016021	integral component of membrane	1.474	0.000	0.000
Module 3	GO:CC	GO:0031224	intrinsic component of membrane	1.474	0.000	0.000
Module 3	GO:BP	GO:0007165	signal transduction	2.717	0.000	0.001
Module 3	GO:CC	GO:0044459	plasma membrane part	5.917	0.000	0.000
Module 3	KEGG	KEGG:04080	Neuroactive ligand-receptor interaction	7.988	0.000	0.002
Module 3	GO:CC	GO:0098590	plasma membrane region	8.453	0.000	0.000
Module 3	GO:MF	GO:0015318	inorganic molecular entity transmembrane transporter activity	3.382	0.000	0.000
Module 3	GO:BP	GO:0007186	G-protein coupled receptor signaling pathway	8.889	0.000	0.002
Module 3	GO:BP	GO:0050794	regulation of cellular process	1.895	0.000	0.002
Module 3	GO:MF	GO:0015075	ion transmembrane transporter activity	3.055	0.000	0.002
Module 3	GO:BP	GO:0012501	programmed cell death	9.600	0.002	0.024
Module 3	KEGG	KEGG:04214	Apoptosis - fly	4.905	0.002	0.054
Module 3	KEGG	KEGG:00020	Citrate cycle (TCA cycle)	4.385	0.011	0.154
Module 3	KEGG	KEGG:01200	Carbon metabolism	2.796	0.017	0.186
Module 3	GO:BP	GO:0006099	tricarboxylic acid cycle	4.800	0.020	0.187
Module 3	KEGG	KEGG:04310	Wnt signaling pathway	3.550	0.023	0.198
Module 3	GO:BP	GO:0006887	exocytosis	4.364	0.027	0.205
Module 3	KEGG	KEGG:00350	Tyrosine metabolism	6.213	0.037	0.271
Module 3	GO:BP	GO:0035556	intracellular signal transduction	2.074	0.045	0.296
Module 4	KEGG	KEGG:04141	Protein processing in endoplasmic reticulum	3.106	0.000	0.000
Module 4	GO:BP	GO:0006082	organic acid metabolic process	3.261	0.000	0.000
Module 4	GO:BP	GO:0044283	small molecule biosynthetic process	3.652	0.000	0.000
Module 4	KEGG	KEGG:01200	Carbon metabolism	3.071	0.000	0.001
Module 4	KEGG	KEGG:01100	Metabolic pathways	1.505	0.000	0.001
Module 4	GO:CC	GO:0005783	endoplasmic reticulum	3.831	0.000	0.005
Module 4	KEGG	KEGG:01212	Fatty acid metabolism	3.685	0.000	0.003
Module 4	GO:BP	GO:0051186	cofactor metabolic process	3.366	0.000	0.005
Module 4	KEGG	KEGG:01230	Biosynthesis of amino acids	3.276	0.000	0.004

(continued)

Module	Test_type	ID	Description	enrichment	pvalue	p.adjust
Module 4	GO:BP	GO:0006520	cellular amino acid metabolic process	3.102	0.000	0.005
Module 4	GO:BP	GO:0005975	carbohydrate metabolic process	3.021	0.000	0.005
Module 4	GO:MF	GO:0019842	vitamin binding	4.253	0.001	0.017
Module 4	GO:BP	GO:0006629	lipid metabolic process	3.069	0.001	0.007
Module 4	GO:MF	GO:0016903	oxidoreductase activity, acting on the aldehyde or oxo group of donors	5.401	0.001	0.017
Module 4	KEGG	KEGG:00030	Pentose phosphate pathway	4.095	0.001	0.014
Module 4	KEGG	KEGG:01040	Biosynthesis of unsaturated fatty acids	5.460	0.003	0.022
Module 4	GO:MF	GO:0050662	coenzyme binding	2.431	0.004	0.037
Module 4	GO:MF	GO:0016746	transferase activity, transferring acyl groups	2.881	0.004	0.037
Module 4	KEGG	KEGG:00061	Fatty acid biosynthesis	4.680	0.006	0.036
Module 4	KEGG	KEGG:04512	ECM-receptor interaction	4.680	0.006	0.036
Module 4	GO:MF	GO:0070279	vitamin B6 binding	3.739	0.007	0.049
Module 4	GO:BP	GO:0055086	nucleobase-containing small molecule metabolic process	2.207	0.007	0.057
Module 4	KEGG	KEGG:03050	Proteasome	2.520	0.009	0.053
Module 4	KEGG	KEGG:00500	Starch and sucrose metabolism	4.095	0.010	0.053
Module 4	GO:BP	GO:0005996	monosaccharide metabolic process	4.174	0.011	0.070
Module 4	GO:CC	GO:0005789	endoplasmic reticulum membrane	3.264	0.013	0.069
Module 4	GO:CC	GO:0044432	endoplasmic reticulum part	3.264	0.013	0.069
Module 4	GO:BP	GO:0044262	cellular carbohydrate metabolic process	5.217	0.014	0.081
Module 4	KEGG	KEGG:00260	Glycine, serine and threonine metabolism	3.150	0.014	0.066
Module 4	KEGG	KEGG:00565	Ether lipid metabolism	4.914	0.015	0.066
Module 4	GO:MF	GO:0017171	serine hydrolase activity	2.778	0.016	0.091
Module 4	GO:BP	GO:1901576	organic substance biosynthetic process	1.350	0.016	0.085
Module 4	KEGG	KEGG:03060	Protein export	3.640	0.016	0.068
Module 4	GO:CC	GO:0042175	nuclear outer membrane-endoplasmic reticulum membrane network	3.060	0.018	0.069
Module 4	GO:MF	GO:0016614	oxidoreductase activity, acting on CH-OH group of donors	3.038	0.018	0.092
Module 4	GO:BP	GO:0006793	phosphorus metabolic process	1.833	0.018	0.085
Module 4	GO:CC	GO:0016021	integral component of membrane	1.232	0.019	0.069
Module 4	GO:CC	GO:0031224	intrinsic component of membrane	1.232	0.019	0.069
Module 4	GO:CC	GO:0000502	proteasome complex	3.560	0.019	0.069
Module 4	KEGG	KEGG:00520	Amino sugar and nucleotide sugar metabolism	2.586	0.021	0.081
Module 4	GO:BP	GO:0019637	organophosphate metabolic process	1.913	0.022	0.085
Module 4	GO:BP	GO:0006575	cellular modified amino acid metabolic process	4.472	0.022	0.085
Module 4	GO:BP	GO:0044249	cellular biosynthetic process	1.332	0.022	0.085
Module 4	GO:CC	GO:1905368	peptidase complex	2.880	0.023	0.072
Module 4	GO:BP	GO:0044255	cellular lipid metabolic process	2.504	0.026	0.092
Module 4	KEGG	KEGG:00280	Valine, leucine and isoleucine degradation	2.340	0.034	0.120
Module 4	KEGG	KEGG:00510	N-Glycan biosynthesis	2.978	0.035	0.120
Module 4	GO:MF	GO:0016875	ligase activity, forming carbon-oxygen bonds	2.991	0.037	0.160
Module 4	KEGG	KEGG:00062	Fatty acid elongation	3.510	0.043	0.139
Module 4	GO:CC	GO:0012505	endomembrane system	1.710	0.048	0.133
Module 5	GO:BP	GO:0046907	intracellular transport	2.909	0.004	0.046
Module 5	KEGG	KEGG:04120	Ubiquitin mediated proteolysis	3.220	0.004	0.148
Module 5	GO:BP	GO:0070727	cellular macromolecule localization	3.117	0.005	0.046
Module 5	GO:BP	GO:0051649	establishment of localization in cell	2.815	0.005	0.046
Module 5	KEGG	KEGG:04142	Lysosome	3.861	0.008	0.148
Module 5	GO:BP	GO:0032535	regulation of cellular component size	6.545	0.009	0.046
Module 5	GO:BP	GO:0032970	regulation of actin filament-based process	6.545	0.009	0.046
Module 5	GO:BP	GO:0043254	regulation of protein complex assembly	6.545	0.009	0.046
Module 5	GO:BP	GO:0044087	regulation of cellular component biogenesis	6.545	0.009	0.046
Module 5	GO:BP	GO:0090066	regulation of anatomical structure size	6.545	0.009	0.046

(continued)

Module	Test_type	ID	Description	enrichment	pvalue	p.adjust
Module 5	GO:MF	GO:0004721	phosphoprotein phosphatase activity	4.534	0.010	0.151
Module 5	GO:BP	GO:0045184	establishment of protein localization	2.530	0.010	0.046
Module 5	GO:BP	GO:0042592	homeostatic process	4.364	0.011	0.046
Module 5	GO:BP	GO:0008104	protein localization	2.494	0.011	0.046
Module 5	GO:BP	GO:0006810	transport	1.904	0.012	0.046
Module 5	KEGG	KEGG:04933	AGE-RAGE signaling pathway in diabetic complications	5.405	0.015	0.198
Module 5	GO:BP	GO:0097435	supramolecular fiber organization	5.035	0.019	0.059
Module 5	GO:BP	GO:0022411	cellular component disassembly	8.727	0.019	0.059
Module 5	GO:BP	GO:0051129	negative regulation of cellular component organization	8.727	0.019	0.059
Module 5	GO:MF	GO:0070279	vitamin B6 binding	4.970	0.020	0.151
Module 5	GO:BP	GO:0030036	actin cytoskeleton organization	4.675	0.023	0.067
Module 5	GO:BP	GO:0045454	cell redox homeostasis	4.364	0.028	0.073
Module 5	GO:BP	GO:0051128	regulation of cellular component organization	4.364	0.028	0.073
Module 5	GO:MF	GO:0016830	carbon-carbon lyase activity	7.179	0.028	0.151
Module 5	GO:MF	GO:0008092	cytoskeletal protein binding	3.314	0.029	0.151
Module 5	GO:MF	GO:0016788	hydrolase activity, acting on ester bonds	2.485	0.030	0.151
Module 5	KEGG	KEGG:04144	Endocytosis	2.402	0.034	0.331
Module 5	GO:MF	GO:0019842	vitamin binding	4.038	0.035	0.151
Module 5	GO:CC	GO:0016021	integral component of membrane	1.278	0.038	0.432
Module 5	GO:CC	GO:0031224	intrinsic component of membrane	1.278	0.038	0.432
Module 5	GO:MF	GO:0019787	ubiquitin-like protein transferase activity	3.801	0.041	0.151
Module 5	GO:MF	GO:0001882	nucleoside binding	2.267	0.045	0.151
Module 5	KEGG	KEGG:00250	Alanine, aspartate and glutamate metabolism	5.405	0.049	0.383
Module 6	GO:MF	GO:0001882	nucleoside binding	3.878	0.000	0.008
Module 6	GO:MF	GO:0016817	hydrolase activity, acting on acid anhydrides	2.295	0.009	0.118
Module 6	KEGG	KEGG:03410	Base excision repair	7.279	0.028	0.460
Module 6	GO:BP	GO:0007275	multicellular organism development	7.059	0.030	0.533
Module 6	KEGG	KEGG:04140	Autophagy - animal	3.276	0.030	0.460
Module 6	GO:MF	GO:0004540	ribonuclease activity	6.140	0.039	0.271
Module 6	GO:MF	GO:0003676	nucleic acid binding	1.463	0.042	0.271
Module 6	KEGG	KEGG:04350	TGF-beta signaling pathway	5.956	0.042	0.460
Module 7	GO:BP	GO:0035556	intracellular signal transduction	4.638	0.001	0.038
Module 7	KEGG	KEGG:04068	FoxO signaling pathway	5.883	0.004	0.069
Module 7	KEGG	KEGG:04320	Dorso-ventral axis formation	8.423	0.004	0.069
Module 7	KEGG	KEGG:04080	Neuroactive ligand-receptor interaction	6.618	0.009	0.077
Module 7	KEGG	KEGG:00511	Other glycan degradation	12.354	0.010	0.077
Module 7	KEGG	KEGG:04070	Phosphatidylinositol signaling system	4.877	0.021	0.130
Module 7	KEGG	KEGG:00562	Inositol phosphate metabolism	4.633	0.024	0.130
Module 7	GO:BP	GO:0007165	signal transduction	2.363	0.033	0.546
Module 8	KEGG	KEGG:00190	Oxidative phosphorylation	20.621	0.000	0.000
Module 8	KEGG	KEGG:01100	Metabolic pathways	3.135	0.000	0.000
Module 8	GO:BP	GO:0017144	drug metabolic process	16.783	0.000	0.000
Module 8	GO:CC	GO:0044429	mitochondrial part	12.346	0.000	0.000
Module 8	GO:BP	GO:0055086	nucleobase-containing small molecule metabolic process	12.781	0.000	0.000
Module 8	GO:CC	GO:0070469	respiratory chain	35.188	0.000	0.000
Module 8	GO:BP	GO:0019637	organophosphate metabolic process	11.077	0.000	0.000
Module 8	GO:CC	GO:0031967	organelle envelope	12.064	0.000	0.000
Module 8	GO:CC	GO:0031975	envelope	12.064	0.000	0.000
Module 8	GO:BP	GO:1901135	carbohydrate derivative metabolic process	9.920	0.000	0.000
Module 8	GO:BP	GO:0022900	electron transport chain	41.026	0.000	0.000
Module 8	GO:CC	GO:0031966	mitochondrial membrane	13.405	0.000	0.000
Module 8	GO:MF	GO:0009055	electron transfer activity	39.773	0.000	0.000
Module 8	GO:BP	GO:0006793	phosphorus metabolic process	8.981	0.000	0.000
Module 8	GO:CC	GO:0098803	respiratory chain complex	33.512	0.000	0.000

(continued)

Module	Test_type	ID	Description	enrichment	pvalue	p.adjust
Module 8	GO:BP	GO:0006091	generation of precursor metabolites and energy	21.099	0.000	0.000
Module 8	GO:CC	GO:0098796	membrane protein complex	9.179	0.000	0.000
Module 8	GO:MF	GO:0016651	oxidoreductase activity, acting on NAD(P)H	31.818	0.000	0.000
Module 8	GO:MF	GO:0015318	inorganic molecular entity transmembrane transporter activity	11.067	0.000	0.000
Module 8	GO:MF	GO:0015075	ion transmembrane transporter activity	10.832	0.000	0.000
Module 8	GO:CC	GO:0019866	organelle inner membrane	13.684	0.000	0.000
Module 8	GO:CC	GO:0045259	proton-transporting ATP synthase complex	37.533	0.000	0.000
Module 8	GO:BP	GO:0015980	energy derivation by oxidation of organic compounds	21.719	0.000	0.000
Module 8	GO:CC	GO:0005746	mitochondrial respiratory chain	31.278	0.000	0.000
Module 8	GO:CC	GO:0033178	proton-transporting two-sector ATPase complex, catalytic domain	26.810	0.000	0.000
Module 8	GO:CC	GO:0098800	inner mitochondrial membrane protein complex	26.810	0.000	0.000
Module 8	GO:MF	GO:0022804	active transmembrane transporter activity	16.746	0.000	0.000
Module 8	GO:CC	GO:0031090	organelle membrane	6.824	0.000	0.000
Module 8	GO:CC	GO:1990204	oxidoreductase complex	23.458	0.000	0.000
Module 8	GO:CC	GO:0016469	proton-transporting two-sector ATPase complex	15.639	0.000	0.000
Module 8	GO:CC	GO:0044455	mitochondrial membrane part	15.639	0.000	0.000
Module 8	GO:BP	GO:1901564	organonitrogen compound metabolic process	2.725	0.000	0.001
Module 8	GO:CC	GO:0044444	cytoplasmic part	2.412	0.002	0.003
Module 8	GO:BP	GO:0006139	nucleobase-containing compound metabolic process	2.332	0.004	0.008
Module 8	GO:BP	GO:0046483	heterocycle metabolic process	2.253	0.005	0.010
Module 8	GO:BP	GO:0006725	cellular aromatic compound metabolic process	2.238	0.005	0.010
Module 8	GO:BP	GO:1901360	organic cyclic compound metabolic process	2.215	0.005	0.010
Module 8	GO:MF	GO:0020037	heme binding	14.141	0.008	0.019
Module 8	GO:MF	GO:0046906	tetrapyrrole binding	14.141	0.008	0.019
Module 8	GO:BP	GO:0006810	transport	2.974	0.008	0.015
Module 8	GO:CC	GO:0044446	intracellular organelle part	2.085	0.012	0.016
Module 8	GO:CC	GO:0005737	cytoplasm	1.822	0.015	0.019
Module 8	GO:MF	GO:0016817	hydrolase activity, acting on acid anhydrides	2.974	0.022	0.046
Module 8	GO:BP	GO:0034641	cellular nitrogen compound metabolic process	1.806	0.023	0.037
Module 9	GO:MF	GO:0020037	heme binding	18.301	0.005	0.052
Module 9	GO:MF	GO:0046906	tetrapyrrole binding	18.301	0.005	0.052
Module 9	GO:BP	GO:0035556	intracellular signal transduction	4.444	0.026	0.624
Module 9	GO:MF	GO:0004672	protein kinase activity	4.334	0.029	0.177
Module 9	GO:MF	GO:0016772	transferase activity, transferring phosphorus-containing groups	3.167	0.032	0.177
Module 9	KEGG	KEGG:00230	Purine metabolism	6.177	0.039	0.153

Supplementary Table 25: List of all the genes in Module 0, ranked by their within-module connectivity, k. The latter four columns give the Log_2 fold-change in expression in response to queen pheromone in each of the four species.

Gene	Name	k	am_fc	bt_fc	lf_fc	ln_fc
GB40730	odorant receptor 2	0.657	0.543	-0.105	1.086	-0.276
GB52877	uncharacterized protein LOC726699 isoform X2	0.585	-0.596	-0.112	0.245	-0.452
GB55921	esterase FE4-like	0.526	-0.072	-0.235	0.847	-0.150
GB42205	uncharacterized protein LOC724413	0.510	0.457	0.093	0.259	-0.125
GB55743	WD repeat-containing protein 78-like isoform X2	0.476	-0.120	1.333	1.646	-0.336
GB43902	hexaprenyldihydroxybenzoate methyltransferase, mitochondrial-like	0.407	-0.066	-0.005	7.514	-0.368
GB54281	cAMP-dependent protein kinase type II regulatory subunit isoform X2	0.398	0.016	0.017	0.171	0.157
GB43306	uncharacterized protein LOC100188904	0.391	-0.793	-0.228	0.921	-0.243
GB49904	bone morphogenetic protein 5-like	0.380	-0.772	0.197	0.683	-0.437
GB45633	uncharacterized protein LOC726990 isoform X2	0.349	0.790	0.158	0.320	0.064
GB48685	endothelin-converting enzyme-like 1-like isoform X1	0.348	0.314	0.100	0.862	0.066
GB45358	nuclear cap-binding protein subunit 2-like	0.347	0.132	0.062	-0.102	-0.036
GB49297	protein eyes shut-like isoform X2	0.334	-0.229	-0.316	1.535	0.055
GB51851	diphthamide biosynthesis protein 7-like isoform X1	0.315	-0.392	-0.114	-0.330	-0.066
GB48100	forkhead box protein K2-like	0.308	0.050	-0.287	0.382	-0.106
GB53410	nicotinamide riboside kinase	0.307	-0.025	0.021	1.512	-0.035
GB52361	odorant receptor 2a	0.307	-2.275	0.354	0.651	-0.469
GB49771	probable U3 small nucleolar RNA-associated protein 11-like isoform X1	0.306	0.085	-0.023	3.629	0.030
GB54350	spermatogenesis-associated protein 6-like isoform X2	0.302	-0.229	0.134	0.223	0.334
GB52326	chemosensory protein 4 precursor	0.277	0.503	0.183	0.313	0.052
100576247	frizzled-2-like, transcript variant X8	0.271	0.159	-0.049	-0.493	0.149
GB41772	heterochromatin protein 1-binding protein 3-like	0.269	-0.107	-0.437	0.639	-0.215
GB46956	homeobox protein B-H2-like	0.267	0.017	0.097	0.970	-0.353
GB44120	venom serine protease 34 isoform X2	0.258	0.792	0.228	0.271	0.115
GB47515	homeobox protein unplugged-like	0.253	-1.151	1.322	1.113	0.393
GB52687	GATA zinc finger domain-containing protein 4-like	0.248	0.085	-0.035	0.425	-0.070
GB54180	segmentation protein paired	0.246	-0.322	-0.005	0.391	-0.061
GB17991	tyramine receptor	0.238	-0.428	0.172	0.118	0.301
GB43643	hepatic leukemia factor isoform X5	0.230	-0.089	-0.181	0.262	-0.495
GB45986	scavenger receptor class B member 1	0.228	0.737	0.218	0.318	0.455
100578193	uncharacterized protein LOC100578193	0.227	-0.376	0.379	0.192	-0.454
726803	uncharacterized protein LOC726803	0.222	0.675	-0.323	-1.698	0.240
GB52620	paired box pox-meso protein isoform X1	0.218	0.346	0.583	0.410	0.085
GB49410	calmodulin-like	0.214	-0.028	-0.008	-1.489	0.317
GB51295	homeotic protein Sex combs reduced	0.199	-0.405	0.076	0.708	-0.237
GB49332	brain-specific homeobox protein homolog	0.199	0.783	0.074	-0.005	-0.302
GB40967	tyrosine hydroxylase	0.194	0.212	0.083	0.013	-0.112
GB53374	connectin isoform X2	0.192	-0.494	0.060	-0.340	-0.648
GB49973	tachykinin-like peptides receptor 99D-like isoformX1	0.190	0.068	-0.354	0.494	-0.030
GB42531	zinc finger protein 470-like	0.184	-0.127	0.093	0.251	0.835
GB52394	odorant receptor 35	0.184	0.674	-0.888	1.733	0.259
GB40567	serine protease nudel	0.179	-0.181	0.132	-0.354	-1.014
GB47274	UNC93-like protein-like	0.176	0.098	0.047	0.278	0.026
100216325	extra macrochaetae	0.172	0.538	-0.212	0.590	0.053
GB40377	uncharacterized protein LOC551717	0.171	-0.439	0.321	0.518	-0.556
100576903	leucine-rich repeat-containing protein 15-like isoform 1	0.168	-0.164	-0.287	0.211	-0.534
GB50585	alpha-2 adrenergic receptor-like	0.164	-1.339	0.107	0.453	0.382
GB44017	uncharacterized protein LOC411209 isoform X2	0.163	0.147	0.157	0.704	0.179
GB40864	titin-like	0.159	-0.271	0.167	0.352	-0.557
GB51441	chromatin modification-related protein eaf-1-like isoform X1	0.157	0.162	-0.038	0.203	-0.381
GB55297	group XV phospholipase A2-like isoform X2	0.157	-0.254	0.042	0.194	0.430
GB42892	uncharacterized protein LOC100578699	0.156	-0.687	0.414	-0.110	0.072
102654715	general transcriptional corepressor trfA-like isoform X2	0.156	0.008	0.148	1.073	-0.040
GB55712	oocyte zinc finger protein XlCOF6-like isoform 2	0.148	0.095	0.322	-0.037	0.025
GB50761	chymotrypsin-1	0.146	1.053	0.300	-0.139	-0.372
GB54775	atrial natriuretic peptide-converting enzyme isoform X2	0.146	0.374	-0.157	-0.146	-0.533

(continued)

Gene	Name	k	am__fc	bt__fc	lf__fc	ln__fc
102655422	uncharacterized protein LOC102655422	0.139	-1.295	0.071	1.006	-0.490
GB54401	elongation of very long chain fatty acids protein AAEL008004-like isoform X2	0.134	2.090	-0.419	0.471	0.403
GB51583	kynurenine/alpha-aminoadipate aminotransferase, mitochondrial-like	0.129	-0.366	-0.612	1.167	-0.378
726761	paired box protein Pax-2a-like isoform X2	0.119	0.095	0.104	0.580	-0.370
GB54748	proteasome activator complex subunit 3-like	0.118	0.750	-0.032	0.763	0.035
GB47018	uncharacterized protein LOC724886	0.116	-0.016	-0.102	0.381	-0.425
410557	ATP synthase subunit d, mitochondrial	0.116	-0.280	-0.145	-0.101	0.117
GB52755	SET and MYND domain-containing protein 4-like isoform X1	0.114	-0.461	-0.071	0.193	0.210
551397	28S ribosomal protein S18a, mitochondrial isoform 2	0.111	0.089	-0.035	5.044	0.065
GB54292	carbohydrate sulfotransferase 11-like	0.108	0.602	-0.181	0.001	-0.709
GB54802	N-acetyllactosaminide beta-1,3-N-acetylglucosaminyltransferase-like isoform X4	0.101	-0.095	-0.020	0.254	-0.112
GB45977	U11/U12 small nuclear ribonucleoprotein 25 kDa protein-like	0.097	-0.407	0.188	0.295	-0.027
GB44425	coiled-coil domain-containing protein 104-like	0.095	1.001	-0.655	0.322	-0.011
GB42296	peroxidase	0.094	0.260	0.055	0.434	-0.088
GB49261	uncharacterized protein LOC100576662 isoform X2	0.094	1.315	-1.902	0.224	0.493
GB40112	uncharacterized protein LOC410462	0.094	0.132	-0.110	1.218	0.006
102655945	uncharacterized protein LOC102655945	0.085	1.061	-0.056	0.057	0.418
GB51846	intraflagellar transport protein 46 homolog	0.085	0.303	0.069	0.369	-0.203
GB41946	cuticular protein analogous to peritrophins 3-D precursor	0.084	-0.432	-0.150	-0.132	-0.364
GB52186	trypsin-1	0.083	0.310	-0.067	0.900	-0.562
725247	probable inactive tRNA-specific adenosine deaminase-like protein 3-like	0.082	0.052	-0.012	-0.175	-0.085
GB43690	uncharacterized protein LOC727344	0.077	0.925	0.699	-0.125	0.733
GB51582	aristaless-related homeobox protein isoform X1	0.077	-0.772	0.114	0.048	-0.586
GB50650	flocculation protein FLO11 isoform X3	0.075	-0.451	0.317	0.294	-0.384
GB53126	polypeptide N-acetylgalactosaminyltransferase 2-like isoform X1	0.073	-0.187	-0.076	0.012	0.253
GB52465	vitellogenin-2-like	0.071	-0.364	-0.183	0.253	0.064
102656830	uncharacterized protein LOC102656830	0.069	-0.580	0.562	-0.771	0.529
GB55389	spondin-1 isoform X1	0.069	-0.107	0.092	-0.298	-0.032
100579019	probable salivary secreted peptide-like	0.068	1.317	0.381	0.966	0.725
GB48935	protein Star-like	0.066	0.533	0.226	-0.029	0.098
GB45062	protein apterous isoform X1	0.064	-0.004	0.067	0.433	-0.219
GB51622	heterogeneous nuclear ribonucleoprotein L isoform X1	0.062	-0.356	-0.025	0.052	0.156
GB48039	etoposide-induced protein 2.4-like isoform X2	0.059	0.148	0.042	0.308	0.048
GB45218	uncharacterized protein LOC408317 isoform 1	0.059	-0.070	0.203	0.140	0.013
GB55286	homeobox protein SIX2	0.056	0.543	0.219	-0.100	0.112
GB41714	uncharacterized protein LOC727150 isoform X2	0.054	-0.383	-0.295	-0.683	0.180
GB48943	isocitrate dehydrogenase [NAD] subunit beta, mitochondrial-like	0.053	-0.263	-0.324	-1.008	0.153
GB42736	TM2 domain-containing protein CG10795-like	0.048	0.588	0.014	0.369	0.350
GB46213	histone RNA hairpin-binding protein	0.045	0.063	0.099	0.008	0.300
GB42548	protein kinase C-binding protein NELL1-like isoform X1	0.043	0.474	0.194	0.226	-0.272
GB46050	protein king tubby-like	0.042	-0.063	-0.091	0.062	-0.034
GB47259	transcription initiation factor TFIID subunit 12 isoform X1	0.038	0.292	0.060	0.518	0.151
GB50014	coiled-coil domain-containing protein 111-like isoform X1	0.038	-0.372	0.063	-0.053	0.156
725329	UPF0489 protein C5orf22 homolog isoform X3	0.037	0.315	-0.061	0.093	0.137
725238	histone H1B-like	0.031	0.275	0.307	0.125	-0.002
GB46620	uncharacterized protein C1orf112 homolog	0.028	0.139	0.483	0.270	0.128
GB43867	uncharacterized protein PFB0765w-like	0.027	0.468	0.326	-0.087	0.028
724536	uncharacterized protein LOC724536	0.021	0.396	0.162	0.905	-0.269
GB52644	ATP synthase-coupling factor 6, mitochondrial	0.019	-0.152	-0.223	-0.052	0.205
GB55014	ras-related protein Rab-2-like	0.013	0.371	-0.046	0.544	0.065
GB41419	zinc finger protein 813-like	0.009	-0.489	0.053	0.313	0.092

Supplementary Table 26: List of all the genes in Module 1, ranked by their within-module connectivity, k. The latter four columns give the Log_2 fold-change in expression in response to queen pheromone in each of the four species.

Gene	Name	k	am_fc	bt_fc	lf_fc	ln_fc
GB55823	tRNA-splicing ligase RtcB homolog	288.606	-0.028	0.161	0.039	-0.010
GB44900	cullin-3 isoform X1	285.798	-0.038	-0.005	-0.009	-0.017
GB54956	autophagy 1	283.901	0.048	-0.011	-0.004	0.029
GB43113	serrate RNA effector molecule homolog isoform X1	274.934	-0.183	0.030	0.046	0.007
GB45829	vacuolar protein sorting-associated protein 4B isoformX1	272.201	0.063	0.026	0.041	0.074
GB47683	LOW QUALITY PROTEIN: vacuolar protein sorting-associated protein 8 homolog	271.823	-0.281	0.064	0.020	0.062
GB41448	rho GTPase-activating protein 26-like isoform X5	269.986	-0.156	-0.033	0.059	0.076
725575	axoneme-associated protein mst101(2)-like	266.565	-0.208	0.094	-0.036	0.090
GB55512	acidic fibroblast growth factor intracellular-binding protein isoform X1	264.662	-0.010	0.025	0.011	-0.433
GB43200	tRNA (uracil-5-)-methyltransferase homolog A-like isoform X2	262.811	-0.180	-0.073	0.000	0.079
GB43952	nitric oxide synthase-interacting protein homolog	259.869	-0.111	0.151	-0.225	0.100
GB45823	pre-mRNA-processing-splicing factor 8-like	256.623	-0.028	0.025	0.023	-0.042
GB44359	leucine-rich repeat-containing protein 16A-like isoform X4	251.887	-0.215	0.020	0.043	0.079
GB53699	survival of motor neuron-related-splicing factor 30-like isoform 2	250.815	-0.053	0.011	-0.037	0.049
GB44682	catalase isoform 1	247.562	-0.212	-0.020	0.051	-0.020
GB52979	dentin sialophosphoprotein-like isoform X1	242.910	-0.130	-0.058	0.029	-0.040
GB47599	cytoplasmic dynein 1 light intermediate chain 1 isoform X2	242.471	-0.376	0.013	0.058	0.035
GB44573	ubiquitin carboxyl-terminal hydrolase 5-like	241.462	0.145	0.067	-0.086	0.060
GB50370	histone-lysine N-methyltransferase SETD1B-like isoform 1	240.883	-0.133	0.102	0.691	0.057
GB41911	procollagen-lysine,2-oxoglutarate 5-dioxygenase 3-like isoform X1	240.264	-0.111	0.099	0.029	0.495
GB44594	tyrosine-protein kinase hopscotch isoform X2	240.018	-0.151	0.028	-0.076	0.020
GB49111	neuropathy target esterase sws isoform X3	239.648	-0.076	-0.005	-0.010	0.298
GB52662	probable actin-related protein 2/3 complex subunit 2 isoform 2	237.852	-0.091	0.039	0.022	0.051
GB48925	superkiller viralicidic activity 2-like 2-like isoform X2	234.511	-0.125	0.027	0.037	-0.007
GB50757	KH domain-containing, RNA-binding, signal transduction-associated protein 3-like isoformX2	232.041	-0.146	-0.073	0.041	0.040
724928	tubulin-specific chaperone C-like isoform 1	232.013	-0.098	0.056	-0.024	0.107
GB56004	ATPase family AAA domain-containing protein 1-A-like	229.972	0.049	0.029	0.058	0.044
GB55941	hemK methyltransferase family member 1-like isoform X2	229.967	-0.064	0.134	-0.059	0.023
GB53725	splicing factor 3B subunit 1-like isoform X2	227.261	-0.355	-0.084	0.082	-0.016
GB49918	optineurin isoform X2	226.533	-0.360	0.037	0.048	0.062
GB42141	probable medium-chain specific acyl-CoA dehydrogenase, mitochondrial-like	225.660	0.661	-0.149	-0.068	0.190
GB49425	ATP-dependent zinc metalloprotease YME1 homolog isoform X3	225.136	-0.450	0.126	0.035	0.100
GB44423	vacuolar fusion protein MON1 homolog A-like	224.900	-0.003	-0.018	0.042	0.012
GB51133	uncharacterized protein LOC725950 isoform X7	224.345	-0.295	-0.025	0.295	-0.058
GB49943	eukaryotic translation initiation factor 3 subunit D	223.696	0.154	0.055	0.032	-0.025
GB43707	smallminded	223.354	-0.511	0.090	0.031	0.036
GB43172	cyclin-dependent kinase 11B isoform X1	222.968	-0.021	-0.056	-0.064	-0.051
GB47089	BRCA1-A complex subunit Abraxas-like isoform X2	222.947	-0.066	0.206	0.037	0.074
GB47208	mitogen-activated protein kinase kinase kinase 10 isoform X4	221.513	-0.155	0.018	0.032	-0.044
GB48347	WASH complex subunit strumpellin-like isoform X3	220.709	-0.369	0.105	-0.008	0.024
GB44556	uncharacterized protein LOC411962 isoform X2	220.167	-0.113	0.155	0.003	0.034
GB50854	AP-1 complex subunit beta-1	219.382	-0.135	-0.021	-0.027	-0.027
GB55540	zinc finger MYM-type protein 3-like	219.303	-0.012	-0.020	0.077	0.108
GB46636	probable exonuclease mut-7 homolog isoform X2	219.014	-0.284	-0.119	0.007	0.025
GB40507	nucleolar protein 10	218.410	-0.260	0.145	-0.055	0.122
GB54228	SUMO-activating enzyme subunit 2 isoform X1	217.331	0.057	0.184	0.108	-0.015
GB54389	copper-transporting ATPase 1 isoform X2	217.116	0.136	-0.024	0.035	-0.076
GB44516	exostasin 2, transcript variant X2	217.072	-0.134	-0.105	0.058	0.059
GB41488	ankyrin repeat and LEM domain-containing protein 2-like isoform X4	216.318	-0.398	0.042	0.036	0.105
GB50042	vesicular integral-membrane protein VIP36	215.918	0.293	-0.070	0.513	0.077

(continued)

Gene	Name	k	am_fc	bt_fc	lf_fc	ln_fc
GB47110	methylcrotonoyl-CoA carboxylase subunit alpha, mitochondrial-like	215.492	-0.037	-0.068	-0.055	-0.732
GB49428	tuberin isoform X2	215.211	-0.197	-0.043	0.065	0.084
GB51360	integrator complex subunit 1-like, transcript variant X2	214.639	-0.001	-0.102	-0.034	0.096
GB45873	ubiquitin conjugation factor E4 A-like isoform X2	214.480	-0.130	-0.067	-0.019	0.091
GB41024	U3 small nucleolar RNA-associated protein 6 homolog isoform X2	214.220	-0.075	0.293	-0.047	-0.013
GB40711	RING finger protein 10-like	214.183	-0.226	-0.061	0.030	0.020
GB50872	general transcription factor IIF subunit 2 isoformX1	213.920	-0.153	0.131	0.066	0.055
GB42977	DNA polymerase delta catalytic subunit isoform X2	212.907	-0.149	0.087	-0.089	0.021
GB54054	LOW QUALITY PROTEIN: ubiquitin carboxyl-terminal hydrolase 7	210.778	0.095	0.019	0.065	-0.248
GB44421	replication protein A 70 kDa DNA-binding subunit isoform X2	210.012	-0.283	0.030	-0.033	0.147
GB46511	protein bric-a-brac 2 isoform X1	209.843	-0.273	0.227	0.101	0.194
GB46065	Hermansky-Pudlak syndrome 5 protein homolog isoform X1	208.516	-0.334	-0.069	-0.018	-0.012
GB49749	uncharacterized protein C12orf4 homolog	208.432	0.108	0.113	0.051	0.033
GB42159	uncharacterized exonuclease C637.09-like isoform X3	208.007	-0.229	0.139	0.041	0.017
GB46431	eukaryotic translation initiation factor 3 subunit A	207.752	-0.048	0.074	0.036	-0.080
GB42058	helicase SKI2W	207.739	-0.339	0.017	0.015	-0.001
GB55476	oxysterol-binding protein-related protein 9-like isoform X4	207.601	-0.121	0.076	0.082	-0.234
GB49296	zinc finger CCCH domain-containing protein 13-like	207.297	-0.395	0.059	0.011	-0.009
GB42448	mediator of RNA polymerase II transcription subunit 15-like isoform X2	206.688	-0.455	0.018	0.283	0.188
410869	vacuolar-sorting protein SNF8 isoform X1	206.502	-0.111	0.043	0.085	0.113
GB50847	SWI/SNF-related matrix-associated actin-dependent regulator of chromatin subfamily E member 1-like	206.231	-0.054	0.119	0.044	0.007
GB50287	RAD50-interacting protein 1-like isoform X1	205.396	-0.257	-0.004	0.178	-0.025
GB50276	dual specificity mitogen-activated protein kinase kinase 4	205.102	0.195	0.049	0.052	0.033
GB54194	charged multivesicular body protein 4b isoform X1	205.028	0.012	0.019	-0.004	0.085
GB45008	angio-associated migratory cell protein-like isoform X2	204.582	-0.069	0.085	-0.182	0.032
GB55459	general transcription factor IIH subunit 1 isoform X1	203.928	-0.056	0.135	0.079	0.034
GB45683	WD repeat-containing protein 3-like	203.881	-0.225	0.041	0.019	0.061
GB41025	chromobox protein homolog 5-like	203.640	-0.358	-0.061	0.056	-0.296
GB51246	mediator of RNA polymerase II transcription subunit 23 isoform X2	203.426	-0.053	-0.044	0.042	-0.006
GB54087	zinc finger FYVE domain-containing protein 19-like	203.271	-0.162	-0.045	0.089	0.097
GB54425	cullin-associated NEDD8-dissociated protein 1-like isoform X2	202.732	0.201	0.085	-0.022	0.033
GB41258	BTB/POZ domain-containing adapter for CUL3-mediated RhoA degradation protein 3-like	202.640	-0.124	0.060	-0.038	0.010
GB51495	u4/U6.U5 tri-snRNP-associated protein 1-like, transcript variant X2	202.473	-0.207	0.062	0.045	0.114
GB47575	tyrosine-protein phosphatase corkscrew isoform X4	202.143	-0.052	-0.069	0.045	0.041
GB41293	histone acetyltransferase KAT8 isoform X1	201.807	0.073	-0.028	0.484	0.024
GB54589	YTH domain family protein 3-like isoform X1	201.617	-0.277	0.048	0.116	0.010
GB45114	HEAT repeat-containing protein 1 isoform X2	201.613	-0.038	0.086	-0.306	0.049
GB44690	serine/threonine-protein phosphatase 4 regulatory subunit 3 isoform X2	200.880	-0.068	0.209	0.048	0.163
GB50409	splicing factor 3A subunit 1 isoformX1	200.840	-0.256	0.167	-0.003	-0.055
GB43192	transcription initiation factor TFIID subunit 6 isoform X2	200.834	-0.004	-0.081	-0.006	-0.050
GB53829	CD2 antigen cytoplasmic tail-binding protein 2 homolog	200.789	-0.290	0.026	-0.038	-0.095
GB44912	lethal(3)malignant brain tumor-like protein 3-like isoform X6	200.712	-0.322	0.096	0.070	0.188
GB47102	general vesicular transport factor p115	200.226	-0.249	0.026	-0.039	0.352
GB52559	LMBR1 domain-containing protein 2 homolog	199.815	0.089	-0.068	0.042	0.013
GB43122	probable RNA-binding protein 19-like isoform X2	199.503	0.104	0.025	0.023	0.058
GB46972	vacuolar protein sorting-associated protein 52 homolog isoform 1	199.017	-0.352	-0.041	-0.054	-0.001
GB40578	sodium/hydrogen exchanger 7 isoform X1	197.993	0.160	-0.060	0.130	-0.042
GB55413	zinc transporter 9-like	197.332	-0.117	0.092	0.033	0.091
GB51717	WASH complex subunit 7-like	196.822	0.098	0.087	0.049	-0.136
GB51954	sec1 family domain-containing protein 2-like isoform X2	196.778	-0.116	-0.037	0.030	0.081

(continued)

Gene	Name	k	am_fc	bt_fc	lf_fc	ln_fc
GB47680	TATA-binding protein-associated factor 172-like isoform X2	196.597	-0.251	0.056	0.448	-0.293
GB52641	glyoxalase domain-containing protein 4-like	196.564	-0.406	0.078	0.001	0.057
GB48101	importin-4-like	196.494	0.324	0.131	0.606	0.065
GB49047	26S protease regulatory subunit 6A	196.445	0.187	-0.077	-0.069	0.012
GB54045	ras-related protein Rab-40C-like isoform 2	196.441	-0.071	-0.039	-0.024	0.078
GB40893	trafficking protein particle complex subunit 8-like isoform X3	196.405	-0.438	0.015	-0.015	-0.219
100578159	zinc finger matrin-type protein CG9776-like isoform X4	196.303	-0.279	-0.007	-0.179	0.673
GB47093	activator of basal transcription 1-like	196.009	-0.259	0.058	-0.021	0.094
GB44486	tyrosine-protein phosphatase non-receptor type 21 isoform X2	195.678	-0.042	-0.028	0.060	0.052
GB49468	RRP12-like protein-like isoform X2	195.375	-0.318	0.054	0.075	-0.037
GB43565	OTU domain-containing protein 5-A-like	195.025	0.116	0.135	0.056	0.042
GB53614	RNA-binding protein 26 isoform X2	195.007	-0.037	0.091	0.042	0.045
GB54328	GDP-Man:Man(3)GlcNAc(2)-PP-Dol alpha-1,2-mannosyltransferase-like	194.815	-0.290	-0.015	0.056	0.036
GB41239	trichohyalin	194.803	-0.497	0.108	0.042	-0.065
GB41795	vacuolar protein sorting-associated protein 33B isoform X2	194.645	0.018	-0.076	-0.009	0.072
GB54526	mitotic spindle assembly checkpoint protein MAD1	194.425	-0.097	0.159	0.056	0.143
GB45184	2-hydroxyacyl-CoA lyase 1-like	194.014	0.240	-0.134	-0.380	-0.091
GB40361	sister chromatid cohesion protein PDS5 homolog B-B-like isoform X1	193.122	-0.278	0.042	-0.004	-0.069
GB54995	Rho-associated, coiled-coil containing protein kinase 2, transcript variant X2	193.003	-0.253	-0.053	0.077	-0.031
GB50952	E3 ubiquitin-protein ligase RNF13-like isoform X2	192.533	-0.043	-0.051	0.063	0.114
GB10936	U4/U6 small nuclear ribonucleoprotein Prp3	192.477	-0.083	0.106	0.039	0.049
GB47660	alpha-1,3-mannosyl-glycoprotein 4-beta-N-acetylglucosaminyltransferase B-like isoform X3	192.160	-0.068	0.063	0.042	0.181
GB40880	DDB1- and CUL4-associated factor-like 1-like isoform X1	191.996	-0.185	0.027	0.087	0.034
GB49654	zinc finger protein 830-like	191.792	-0.538	0.051	0.042	0.117
GB45214	zinc finger FYVE domain-containing protein 16 isoform X5	191.541	-0.217	0.041	0.079	0.165
GB49409	tetratricopeptide repeat protein 7B-like, transcript variant X5	191.376	-0.237	-0.009	0.005	0.017
GB43540	uncharacterized protein LOC552071	191.181	-0.323	0.040	0.052	0.087
GB47904	26S protease regulatory subunit 7	191.168	0.193	0.026	-0.088	-0.022
GB43124	uncharacterized protein C19orf47 homolog	190.700	-0.127	0.009	-0.111	0.114
GB47888	bifunctional protein NCOAT-like isoform X2	190.612	-0.179	0.012	-0.121	0.055
GB55056	spermatogenesis-associated protein 20 isoform X2	189.608	-0.420	-0.134	0.293	0.076
GB55644	uncharacterized LOC409221, transcript variant X3	189.462	0.018	0.038	0.023	0.007
410886	E3 ubiquitin-protein ligase UBR2-like	189.454	-0.066	-0.113	0.133	0.055
GB54718	DNA repair protein REV1	188.920	-0.159	0.014	0.102	0.102
GB46344	ubiquitin carboxyl-terminal hydrolase 8-like isoform X2	188.908	0.134	0.013	0.067	0.268
GB49490	actin-like protein 87C-like	188.715	0.274	0.001	0.057	0.122
GB40741	PAX-interacting protein 1-like	187.839	-0.149	0.094	-0.160	0.086
GB45436	coiled-coil and C2 domain-containing protein 1-like isoform X2	187.646	-0.233	-0.004	-0.315	0.173
GB40948	uncharacterized protein LOC412397 isoform X2	187.602	-0.120	-0.199	0.033	-0.012
GB42841	very long-chain specific acyl-CoA dehydrogenase, mitochondrial-like	187.450	0.103	-0.056	0.190	-0.203
726952	ER degradation-enhancing alpha-mannosidase-like 1	187.413	-0.262	0.064	-0.016	-0.030
GB46070	protein SMG5-like	187.278	-0.123	-0.093	0.306	-0.032
GB45452	AP-3 complex subunit mu-1-like isoform X1	187.048	0.066	0.026	-0.031	-0.011
GB50607	grpE protein homolog 1, mitochondrial	187.038	0.010	-0.045	-0.025	0.085
GB43637	protein asunder homolog	186.757	0.248	0.179	-0.044	-0.042
GB50231	protein RMD5 homolog A-like	186.629	0.306	-0.021	0.033	0.062
GB40744	squamous cell carcinoma antigen recognized by T-cells 3	186.590	0.003	0.143	0.077	0.004
GB50945	dentin sialophosphoprotein-like	186.476	-0.240	0.217	-0.089	-0.012
GB50067	charged multivesicular body protein 3	186.297	0.037	-0.039	-0.047	0.091
GB51588	actin-related protein 2-like isoform X5	186.025	-0.121	-0.014	0.084	0.092
GB47440	dynamin related protein 1	185.715	0.339	0.122	0.027	0.041
GB42750	LOW QUALITY PROTEIN: PHD finger protein 14-like	185.705	-0.090	0.156	0.022	0.121
GB42223	elongation factor G, mitochondrial-like	185.662	-0.288	0.011	-0.095	0.721
GB42739	xanthine dehydrogenase isoform X4	185.648	-0.141	-0.103	-0.062	0.063

(continued)

Gene	Name	k	am_fc	bt_fc	lf_fc	ln_fc
GB48532	protein zer-1 homolog isoform X2	185.282	-0.031	0.016	0.101	0.069
GB55550	ras GTPase-activating protein 1-like, transcript variant X3	185.050	0.044	0.164	0.082	0.031
GB48631	probable complex I intermediate-associated protein 30, mitochondrial-like	184.959	0.057	0.018	0.087	0.077
GB50083	pre-mRNA-splicing factor SPF27 isoform X1	184.875	0.021	0.074	0.027	0.024
GB47333	probable glutamate–tRNA ligase, mitochondrial-like	184.870	-0.353	0.025	-0.007	0.275
GB46028	E3 ubiquitin-protein ligase RNF14-like	184.647	-0.469	0.038	1.186	0.209
GB43178	WD repeat-containing protein mio-B isoform X2	184.577	0.029	0.053	0.068	0.033
GB41080	ubiquitin carboxyl-terminal hydrolase 14-like isoform 2	184.341	0.123	-0.094	0.036	-0.022
GB45113	mitochondrial inner membrane protein OXA1L-like	182.835	-0.400	-0.053	-0.062	-0.065
GB46026	mitochondrial import receptor subunit TOM70	182.748	0.101	0.027	-0.144	0.069
GB42452	mediator of RNA polymerase II transcription subunit 27	182.523	-0.273	0.105	0.100	0.101
GB51699	WD repeat-containing protein 43-like	182.248	0.267	0.150	0.117	0.053
GB45739	2-oxoisovalerate dehydrogenase subunit alpha, mitochondrial-like isoform 1	182.211	0.117	0.024	-0.038	-0.197
GB47420	nuclear RNA export factor 1-like isoform 2	182.079	-0.306	0.047	0.072	0.134
GB46118	cerebellar degeneration-related protein 2-like isoform X1	181.506	-0.181	0.141	0.146	-0.001
GB40466	ATP-binding cassette sub-family F member 2-like isoform X1	181.493	-0.293	0.102	0.014	0.068
GB45345	zinc finger MYND domain-containing protein 11-like isoform X2	181.475	-0.266	-0.010	0.032	-0.002
GB54780	protein brunelleschi-like isoform X2	181.363	-0.092	0.045	0.009	-0.002
GB52883	FAM203 family protein GA19338-like	180.657	-0.379	-0.072	0.020	0.082
GB52978	TBC1 domain family member 15	180.579	-0.070	-0.021	0.046	0.117
GB48457	peptidylprolyl isomerase domain and WD repeat-containing protein 1 isoform X3	180.397	-0.266	0.033	0.150	0.098
GB44048	endoplasmic reticulum lectin 1-like isoform X4	179.737	0.213	0.034	-0.049	0.038
GB43448	calpain-B	179.650	-0.150	-0.189	0.095	-0.055
GB49027	sulfide:quinone oxidoreductase, mitochondrial-like isoform X2	179.615	-0.241	0.062	0.028	0.015
GB41713	TATA element modulatory factor-like isoform X2	179.524	-0.594	-0.133	0.082	0.034
GB52536	LOW QUALITY PROTEIN: proteasome-associated protein ECM29 homolog	179.513	-0.371	-0.105	0.025	0.036
GB47250	THO complex subunit 1-like isoform X2	179.502	-0.461	-0.036	-0.011	0.107
GB46034	syntaxin-12	179.272	-0.564	0.105	0.060	-0.009
GB46121	ubiquitin fusion degradation protein 1 homolog isoform X2	179.208	0.163	-0.069	-0.060	0.062
GB45831	beta-parvin-like	179.062	0.098	-0.072	0.041	0.065
GB55931	double-strand-break repair protein rad21 homolog isoform X1	178.517	0.093	-0.006	0.128	0.017
GB51496	uncharacterized protein C17orf85 homolog	178.481	-0.443	0.021	0.086	0.021
GB52628	heat shock factor protein isoform X3	178.385	-0.403	-0.107	0.128	-0.012
GB46594	striatin-interacting proteins 2-like isoform X1	178.316	-0.052	-0.078	-0.081	0.145
GB46074	survival motor neuron protein-like	178.233	0.032	-0.105	-0.125	-0.002
GB47329	WD and tetratricopeptide repeats protein 1-like isoform X2	178.168	-0.178	-0.016	0.001	0.173
GB42204	rho guanine nucleotide exchange factor 3-like	177.960	-0.399	-0.016	-0.060	0.021
GB45868	F-box/WD repeat-containing protein 7 isoform X2	177.795	-0.233	0.075	0.080	-0.150
GB48544	coiled-coil domain-containing protein 43-like	177.549	-0.002	0.032	0.214	0.094
GB50345	probable phosphorylase b kinase regulatory subunit alpha-like isoform X4	177.041	-0.197	-0.137	0.132	-0.020
GB49040	KAT8 regulatory NSL complex subunit 2 isoform X5	176.859	0.063	0.002	0.095	0.101
GB50587	uncharacterized protein LOC410622	176.823	-0.129	-0.049	0.030	0.021
GB43464	lysosomal Pro-X carboxypeptidase-like	176.813	-0.032	-0.141	-0.033	-0.373
GB49651	exocyst complex component 3	176.785	0.344	0.034	0.078	0.056
GB47161	pre-mRNA-splicing factor 18-like	176.685	-0.316	-0.041	0.078	0.050
GB53617	sorting and assembly machinery component 50 homolog	176.588	0.247	0.040	0.060	0.000
GB44439	eukaryotic initiation factor 4A-III-like isoform 1	176.366	0.245	0.037	0.022	0.128
GB55333	N-alpha-acetyltransferase 35, NatC auxiliary subunit isoform X2	176.327	-0.217	-0.021	0.162	0.029
GB46061	RNA-binding protein 28-like	176.308	-0.530	0.038	-0.060	0.118
GB41244	inositol polyphosphate 5-phosphatase K-like isoformX1	176.006	0.425	-0.133	-0.380	0.076
GB52848	titin-like	175.912	-0.713	-0.008	0.830	0.328
GB46036	periodic tryptophan protein 2 homolog	175.769	-0.564	0.132	-0.043	0.085
GB50145	ubiquitin domain-containing protein 2-like	175.671	-0.096	-0.017	-0.067	0.100

(continued)

Gene	Name	k	am_fc	bt_fc	lf_fc	ln_fc
GB46927	F-box only protein 21-like	175.665	-0.531	-0.024	0.690	0.850
GB51464	rho GTPase-activating protein 44-like isoform X2	175.508	-0.036	0.075	-0.016	0.112
GB44172	protein CASC3-like isoform X2	175.365	-0.278	0.057	0.105	0.071
GB46655	splicing factor U2AF 50 kDa subunit isoform X1	175.346	0.241	0.063	-0.016	-0.018
GB49596	NEDD8-activating enzyme E1 catalytic subunit-like	175.331	0.002	0.073	-0.037	0.022
GB49974	3-hydroxyisobutyryl-CoA hydrolase, mitochondrial-like isoform X1	175.257	-0.363	0.026	0.096	-0.002
GB46211	zinc finger protein 665-like isoform X3	174.898	-0.037	0.092	0.039	0.088
GB42525	zinc finger protein ZPR1	174.773	-0.176	0.080	-0.139	0.089
GB49423	probable queuine tRNA-ribosyltransferase	174.770	-0.069	-0.047	0.084	0.135
GB51806	coiled-coil domain-containing protein 93 isoform X2	174.661	-0.074	-0.078	-0.065	0.281
GB45534	exocyst complex component 7	174.532	-0.169	0.086	0.109	0.038
GB47192	exosome complex component MTR3-like isoform X2	174.451	0.089	0.028	0.010	0.147
GB54583	H/ACA ribonucleoprotein complex non-core subunit NAF1-like	174.213	-0.595	0.031	-0.072	-0.048
GB40858	probable ATP-dependent RNA helicase DDX47-like isoform 1	174.212	-0.234	0.056	-1.075	0.022
GB44010	zinc finger protein 23-like isoform 1	174.099	-0.475	0.243	-0.475	0.055
GB49244	charged multivesicular body protein 5-like	174.097	0.253	0.067	0.095	0.035
GB46494	Golgi reassembly-stacking protein 2-like	174.007	0.104	0.078	0.596	-0.072
GB40267	probable 39S ribosomal protein L45, mitochondrial	173.613	-0.311	0.081	0.117	0.045
GB41968	protein max isoform 2	173.479	0.140	-0.019	0.103	0.041
GB48170	ran GTPase-activating protein 1-like isoform X1	173.431	-0.100	0.188	-0.020	0.130
GB52673	gamma-tubulin complex component 6-like isoform X1	173.260	-0.317	0.105	0.036	0.147
GB49776	retinoblastoma-binding protein 5-like isoformX1	173.230	-0.130	0.066	-0.042	0.073
GB41902	pre-rRNA-processing protein TSR1 homolog	173.175	-0.250	0.101	0.029	0.004
GB40559	CDK5 regulatory subunit-associated protein 3-like	173.018	-0.183	0.013	-0.085	-0.095
100576610	mitochondrial ribonuclease P protein 3-like	172.912	-0.459	0.064	0.024	-0.001
GB40901	probable cleavage and polyadenylation specificity factor subunit 2 isoform X1	172.492	-0.063	0.136	1.290	-0.033
GB44940	exosome complex exonuclease RRP44-like isoform X1	172.448	-0.123	0.018	-0.018	0.061
GB50421	prosaposin isoformX1	172.352	-0.146	-0.105	0.045	-0.194
GB50747	trafficking protein particle complex subunit 13-like	172.300	-0.302	-0.023	0.002	0.117
GB43820	PHD finger and CXXC domain-containing protein CG17446-like isoform 1	172.086	-0.410	-0.004	0.013	0.090
GB50846	vacuolar protein-sorting-associated protein 36	172.061	-0.193	-0.059	0.032	0.034
GB54227	phosducin-like protein-like isoform 1	171.960	-0.100	-0.122	0.058	0.170
GB48450	bobby sox, transcript variant X3	171.925	-0.212	-0.097	0.089	0.038
GB45905	thyroid receptor-interacting protein 11 isoform X3	171.893	-0.420	-0.162	0.042	-0.069
GB51462	methyltransferase-like protein 23-like isoform X2	171.635	-0.683	0.028	0.431	0.077
GB43305	ubiquitin specific protease-like	171.466	-0.082	-0.047	-0.029	0.064
GB43888	parafibromin	171.400	0.014	0.175	-0.010	0.036
GB52977	UBX domain-containing protein 1-A-like	171.159	-0.300	0.060	-0.072	0.282
GB54938	ATPase WRNIP1-like isoform X6	171.130	-0.337	0.003	0.024	-0.112
GB42479	T-complex protein 1 subunit eta	171.027	0.193	0.140	-0.016	-0.015
GB42447	WW domain-binding protein 11-like	170.595	-0.067	0.035	0.010	0.058
GB41300	bystin isoform 1	170.473	-0.085	0.087	-0.052	-0.035
GB44865	rab GTPase-binding effector protein 1-like isoform X2	170.166	0.238	0.114	0.056	0.080
GB45370	DNA-directed RNA polymerase I subunit RPA2 isoform X2	170.030	0.021	-0.043	-0.412	-0.085
GB44414	alpha-mannosidase 2 isoform X1	169.963	-0.125	-0.081	0.059	-0.046
GB40463	ethanolaminephosphotransferase 1-like	169.767	-0.037	0.037	-0.052	0.122
GB54300	probable U2 small nuclear ribonucleoprotein A' isoform X1	169.481	-0.060	0.032	-0.046	0.100
GB42451	serine/threonine-protein kinase TBK1	169.370	-0.321	0.138	0.005	0.142
GB50972	gastrulation defective protein 1 homolog	169.318	-0.197	0.066	0.190	-0.003
GB51706	histone-lysine N-methyltransferase SETD2-like isoform X1	169.310	-0.448	-0.016	0.094	0.118
GB54377	male-specific lethal 1 homolog	169.302	-0.011	0.079	0.079	-0.348
GB52165	probable ATP-dependent RNA helicase YTHDC2 isoform X1	169.149	-0.217	-0.149	-0.050	0.104
GB40320	protein tumorous imaginal discs, mitochondrial-like isoform X1	169.053	-0.162	0.004	0.038	-0.042
GB51655	nucleoporin GLE1-like	168.835	-0.085	-0.064	-0.039	0.014
GB42997	uncharacterized protein LOC100578201	168.760	-0.380	0.169	0.111	0.002
GB40563	threonine–tRNA ligase, cytoplasmic-like isoform X1	168.650	0.189	0.008	-0.030	-0.044

(continued)

Gene	Name	k	am_fc	bt_fc	lf_fc	ln_fc
GB43815	probable 39S ribosomal protein L23, mitochondrial-like isoform X1	168.326	-0.159	-0.003	-0.021	0.087
GB42645	kinesin B	168.289	-0.537	0.274	0.178	-0.070
GB42933	serine/threonine-protein kinase TAO1 isoform X2	168.168	-0.118	-0.014	-0.103	-0.067
GB53934	rho GTPase-activating protein 24-like	168.018	-0.324	0.055	-0.038	0.104
GB52990	BRISC and BRCA1-A complex member 1-like	167.991	-0.325	0.117	0.011	0.000
GB42381	E3 ubiquitin-protein ligase RNF126-like isoform X5	167.958	-0.066	0.005	0.055	0.081
GB55397	vesicle-associated membrane protein 7	167.887	0.142	-0.087	-0.037	0.052
GB53172	ADAM 17-like protease-like isoform 2	167.549	0.075	-0.045	0.024	0.102
GB44189	glutamic acid-rich protein-like	167.435	0.088	0.053	-0.048	-0.065
GB44397	regulator of microtubule dynamics protein 1-like isoform X1	167.408	-0.199	-0.023	-0.012	0.001
GB49996	regulator of G-protein signaling loco isoform X4	167.176	-0.009	-0.024	0.066	0.114
GB50589	DDRGK domain-containing protein 1-like	166.950	-0.102	-0.095	-0.048	0.064
GB53147	cell division cycle protein 16 homolog isoform X2	166.635	-0.130	0.183	0.153	0.023
GB47744	sorting nexin-25-like	166.613	-0.049	-0.073	0.154	0.141
GB47826	alkylated DNA repair protein alkB homolog 8-like	166.429	-0.575	-0.065	-0.083	0.046
GB44104	V-type proton ATPase subunit G	166.402	0.017	-0.017	0.083	0.071
GB40780	zinc finger protein 330 homolog	166.146	-0.406	0.077	0.068	0.091
GB50183	cylicin-2-like	166.137	0.166	0.017	0.218	-0.164
GB51867	ubiquitin carboxyl-terminal hydrolase isozyme L5	166.115	-0.013	0.025	-0.045	0.126
GB53132	trifunctional enzyme subunit beta, mitochondrial-like	166.078	0.253	-0.029	-0.140	0.185
GB41755	vacuolar protein sorting-associated protein 37A	165.533	0.186	0.071	0.075	-0.027
GB48266	Hermansky-Pudlak syndrome 3 protein homolog	165.228	-0.146	-0.091	0.131	0.124
GB51701	CUE domain-containing protein 1	165.138	-0.284	-0.202	-0.083	0.144
GB49608	protein angel-like isoform X1	164.997	-0.475	-0.150	0.010	-0.391
GB53228	vacuolar protein sorting-associated protein 37B-like	164.960	-0.100	0.032	0.052	0.067
GB45119	elongation factor Ts, mitochondrial-like	164.901	0.416	-0.040	0.031	-0.035
GB45360	exportin-2	164.633	0.331	0.115	-0.053	0.039
GB49226	tetratricopeptide repeat protein 1-like	164.561	-0.131	0.065	-0.004	0.119
GB50920	39S ribosomal protein L37, mitochondrial	164.535	-0.063	0.082	-0.022	0.044
GB54957	anaphase-promoting complex subunit 7 isoform X1	164.376	0.108	-0.077	0.029	-0.015
GB55017	putative GTP-binding protein 6-like isoform X1	164.245	-0.428	0.098	0.202	0.065
GB42207	eukaryotic translation initiation factor 2D-like isoform X2	164.231	-0.440	-0.188	0.085	0.084
GB48145	tyrosine-protein phosphatase non-receptor type 61F-like	164.163	-0.300	0.313	-0.022	0.362
GB44883	protein bunched, class 2/F/G isoform isoform X2	164.085	0.142	0.043	0.110	0.082
GB54809	cell wall protein IFF6-like isoform X1	164.074	-0.279	0.094	0.019	-0.049
GB46339	heat shock protein 75 kDa, mitochondrial isoform 1	163.973	0.223	0.086	0.083	0.110
102654871	DCN1-like protein 3-like	163.739	-0.545	-0.115	-0.053	-0.027
GB44262	spectrin beta chain, non-erythrocytic 5 isoform X4	163.638	-0.035	-0.099	-0.101	-0.092
GB54321	RuvB-like 2 isoform X1	163.574	0.269	0.089	0.108	-0.020
GB13213	eukaryotic translation initiation factor 4 gamma	163.516	-0.340	0.013	-0.050	0.075
GB52916	phosphatidylinositide phosphatase SAC2-like isoform X2	163.515	-0.204	0.003	0.158	0.187
GB51426	asparagine synthetase domain-containing protein 1 isoform X6	163.373	-0.107	-0.152	0.082	0.073
GB44028	vacuolar protein sorting-associated protein 35 isoform X2	163.272	-0.058	-0.346	0.038	-0.049
GB45341	polypeptide N-acetylgalactosaminyltransferase 35A-like	163.094	-0.417	0.088	1.666	-0.012
GB54701	suppressor of G2 allele of SKP1 homolog isoformX1	162.668	-0.177	0.120	-0.009	1.085
GB42667	trafficking protein particle complex subunit 4-like	162.088	-0.220	-0.175	0.039	-0.006
GB41333	DNA-directed RNA polymerase III subunit RPC1-like isoform X1	161.616	-0.158	0.168	-0.165	-0.375
GB54198	liprin-alpha-2-like isoform X13	161.551	-0.154	-0.035	0.400	0.001
GB40399	synaptobrevin homolog YKT6 isoformX1	161.403	-0.045	0.048	0.021	-0.005
GB44449	putative 28S ribosomal protein S5, mitochondrial isoform X1	161.331	0.166	-0.028	0.031	0.006
GB44293	lisH domain and HEAT repeat-containing protein KIAA1468 homolog isoform X1	161.004	-0.392	-0.026	0.059	-0.077
GB55595	probable 39S ribosomal protein L24, mitochondrial	160.670	-0.237	-0.024	0.049	0.045
GB49250	heme oxygenase isoform X1	160.548	-0.706	-0.186	0.037	0.045
GB47236	micronuclear linker histone polyprotein isoform X1	160.442	-0.047	0.038	0.064	0.062
GB49158	dynactin subunit 2, transcript variant X2	159.937	-0.077	0.072	-0.209	0.130
GB47884	TELO2-interacting protein 2-like isoform X1	159.816	-0.270	0.302	0.113	0.112
GB55913	integrator complex subunit 2	159.763	-0.370	0.086	0.042	-0.048
GB46147	uncharacterized protein LOC408724	159.238	0.187	-0.004	-0.018	0.033

(continued)

Gene	Name	k	am__fc	bt__fc	lf__fc	ln__fc
GB51413	C3 and PZP-like alpha-2-macroglobulin domain-containing protein 8-like	159.115	0.104	0.024	0.154	0.054
GB55847	ubiquitin activating enzyme 1 isoform 1	159.073	-0.098	-0.008	-0.010	-0.296
GB44597	pleiotropic regulator 1	159.035	0.112	0.078	0.144	0.205
GB40881	transcription initiation factor IIA subunit 1 isoform 1	159.019	-0.359	0.015	-0.072	-0.022
GB41600	high mobility group protein 20A-like isoform X1	158.977	-0.711	0.154	-0.076	0.054
GB45297	zinc finger FYVE domain-containing protein 1-like	158.813	0.114	0.040	0.083	0.022
GB51400	probable phenylalanine–tRNA ligase alpha subunit-like isoform X3	158.773	0.115	0.031	0.012	0.165
GB49652	cell division cycle 5-like protein-like	158.664	-0.243	0.099	0.095	1.240
GB48349	zinc finger CCCH domain-containing protein 18-like isoform X3	158.531	-0.538	0.056	0.069	0.055
GB44901	conserved oligomeric Golgi complex subunit 2-like isoform X2	158.426	-0.218	-0.031	0.065	0.144
GB47434	F-box only protein 6-like isoform 1	158.212	-0.018	-0.052	0.083	0.071
GB45822	corepressor interacting with RBPJ 1-like	158.192	-0.296	0.102	0.107	0.109
GB48534	probable protein phosphatase 2C T23F11.1-like isoform X1	158.183	-0.174	0.034	0.030	0.001
GB40760	syntaxin-5-like	158.158	0.069	0.011	-0.053	0.023
GB43848	glucose-induced degradation protein 8 homolog isoform X2	157.961	0.276	0.117	0.141	0.053
GB54244	drebrin-like protein-like	157.855	-0.182	0.094	0.173	-0.036
GB52462	THUMP domain-containing protein 3-like isoform X1	157.790	-0.504	0.022	-0.213	0.167
GB40672	type II inositol 1,4,5-trisphosphate 5-phosphatase-like isoform X1	157.693	-0.151	-0.045	0.089	0.193
GB49112	probable uridine-cytidine kinase-like isoformX1	157.377	0.194	0.044	0.142	-0.020
GB52060	septin-1-like	157.246	-0.321	0.060	0.078	0.069
GB49020	DNA-directed RNA polymerase III subunit RPC5	157.229	-0.374	0.300	-0.608	0.154
GB42771	F-box/WD repeat-containing protein 5-like	157.095	-0.695	0.308	-0.055	0.184
GB43266	C-terminal-binding protein isoform X3	157.011	-0.396	-0.183	-0.065	-0.061
GB40833	UPF0396 protein CG6066-like isoform X3	156.972	-0.261	-0.016	-0.030	0.024
GB54570	golgin subfamily A member 2-like isoform X2	156.967	-0.363	0.049	0.026	0.107
725816	homocysteine S-methyltransferase 2-like	156.846	-0.277	-0.223	-0.049	-0.009
GB49537	probable DNA mismatch repair protein Msh6	156.722	-0.091	-0.006	-0.087	-0.026
GB42564	serine/threonine-protein phosphatase 2A 56 kDa regulatory subunit gamma isoform-like isoform X2	156.492	-0.098	0.056	0.159	0.113
GB45761	T-complex protein 1 subunit gamma	156.211	0.420	0.083	0.326	0.453
GB46115	U4/U6 small nuclear ribonucleoprotein Prp31-like isoform X2	156.196	0.255	0.069	0.059	-0.051
GB55994	dual specificity protein phosphatase 12 isoform X2	155.992	-0.167	-0.026	0.007	-0.007
GB46248	sorting nexin lst-4-like isoform 1	155.920	0.055	-0.030	0.166	-0.119
GB53317	dentin sialophosphoprotein-like	155.904	-0.652	0.096	0.043	0.066
GB51762	THO complex subunit 5 homolog isoform X1	155.638	0.052	0.176	0.031	0.005
GB48817	nuclear protein localization protein 4 homolog isoform X3	155.634	0.351	-0.033	0.129	0.219
GB43502	E3 UFM1-protein ligase 1 homolog	155.525	-0.355	0.151	-0.028	0.044
GB44289	ataxin-3-like isoform X4	155.443	-0.132	-0.040	0.808	0.041
GB40308	hsp70-binding protein 1-like	155.243	0.330	0.052	0.687	0.024
GB45397	MATH and LRR domain-containing protein PFE0570w-like	155.167	-0.716	0.135	0.012	0.103
GB46459	islet cell autoantigen 1-like isoform X2	155.086	-0.073	0.141	-0.012	0.175
GB41802	uncharacterized protein LOC552003 isoform X4	154.883	0.077	0.064	-0.017	-0.004
GB48821	structural maintenance of chromosomes protein 6-like isoform X4	154.721	-0.248	0.085	0.022	0.086
GB47184	SCY1-like protein 2-like	154.647	-0.236	-0.086	0.738	0.012
GB50997	structural maintenance of chromosomes protein 3	154.643	0.042	-0.054	-0.092	0.067
GB42274	regulation of nuclear pre-mRNA domain-containing protein 1B-like isoform X2	154.545	0.225	0.059	0.085	0.012
GB51564	prefoldin subunit 3-like	154.533	0.203	-0.008	-0.036	0.057
GB43955	protein PFF0380w-like	154.491	0.042	-0.119	0.143	0.010
GB40388	exocyst complex component 8	154.447	0.093	0.092	0.069	0.006
GB51586	26S protease regulatory subunit 10B	154.429	0.064	-0.047	-0.187	0.038
GB41759	N-acetylglucosaminyl-phosphatidylinositol biosynthetic protein-like isoform X2	154.294	-0.430	-0.111	-0.029	-0.012
GB51603	peptidyl-alpha-hydroxyglycine alpha-amidating lyase 1-like isoform X5	154.199	-0.638	0.043	-0.021	0.132
GB50511	DNA mismatch repair protein Mlh1 isoform X2	154.043	-0.042	0.001	-0.019	0.148

(continued)

Gene	Name	k	am_fc	bt_fc	lf_fc	ln_fc
GB50998	pre-mRNA 3'-end-processing factor FIP1-like	153.795	0.352	0.067	0.009	0.020
GB40364	conserved oligomeric Golgi complex subunit 8-like	153.743	-0.389	0.038	0.002	0.070
GB41852	centromere-associated protein E isoform X8	153.348	-0.097	0.066	0.621	0.078
GB45544	uncharacterized protein LOC552484	153.299	-0.198	-0.078	-0.177	0.147
GB55753	dehydrogenase/reductase SDR family protein 7-like isoform 1	153.268	-0.176	-0.016	-0.035	0.108
GB45693	DNA topoisomerase 3-beta-1-like isoform 2	153.200	0.101	0.106	0.122	0.026
GB51599	collagen type IV alpha-3-binding protein-like isoformX1	153.003	0.028	0.117	-0.071	0.002
GB48596	anaphase-promoting complex subunit 4-like isoformX2	152.946	-0.183	0.007	0.161	0.040
GB53191	arf-GAP domain and FG repeat-containing protein 1 isoform X1	152.926	-0.117	-0.127	-0.048	-0.028
GB44757	actin-interacting protein 1 isoform X2	152.868	0.062	-0.084	0.093	-0.018
GB49773	sequestosome-1	152.779	-0.096	-0.269	0.168	0.370
GB51334	methionine aminopeptidase 2	152.762	0.168	0.026	0.080	0.027
GB51968	conserved oligomeric Golgi complex subunit 3	152.691	-0.252	0.032	0.122	0.177
GB50357	clathrin heavy chain-like isoform 1	152.659	-0.357	-0.035	-0.029	-0.425
GB41668	serine/threonine-protein kinase/endoribonuclease IRE1 isoform X2	152.628	-0.261	-0.039	0.117	0.130
GB48955	microfibrillar-associated protein 1	152.621	-0.003	0.098	-0.024	0.001
GB47180	TATA-box-binding protein-like isoform 1	152.572	-0.556	0.014	-0.156	0.141
GB50280	iron-sulfur cluster assembly 2 homolog, mitochondrial-like	152.514	-0.177	-0.205	-0.022	0.069
102656136	prefoldin subunit 4-like	152.482	-0.693	0.110	-0.074	0.199
GB42874	transforming acidic coiled-coil-containing protein 3 isoform X1	152.267	-0.246	0.122	0.171	0.133
GB50104	KAT8 regulatory NSL complex subunit 3 isoform X2	152.231	-0.333	0.126	0.588	0.192
GB48855	uncharacterized protein YJR142W-like	152.199	-0.186	0.010	-0.088	-0.062
GB44338	small G protein signaling modulator 3 homolog	151.689	0.125	0.010	0.050	0.188
GB54685	uncharacterized protein C3orf18 homolog isoform X1	151.666	-0.209	-0.160	0.159	0.011
GB46457	GPI ethanolamine phosphate transferase 3-like isoform X2	151.461	-0.079	-0.072	0.115	0.056
411727	bromodomain-containing protein 8	151.384	0.053	0.155	-0.077	-0.024
GB54643	putative mitochondrial inner membrane protein-like isoform X7	151.202	-0.228	-0.024	0.062	-0.031
GB49025	zinc transporter foi-like isoform X4	151.022	-0.107	-0.127	-0.047	0.006
GB51103	COP9 signalosome complex subunit 6-like isoform X2	150.822	0.167	0.086	0.000	0.070
GB42880	protein FAM8A1-like isoform X2	150.730	0.125	-0.176	0.133	0.091
GB46641	E3 ubiquitin-protein ligase LRSAM1-like isoform X3	150.537	-0.310	-0.074	-0.062	0.002
GB41835	sorting nexin-13-like isoform X2	150.327	-0.209	-0.007	0.121	0.080
GB45156	WD repeat-containing protein 59-like isoform X2	150.246	0.084	-0.072	0.194	-0.009
GB43439	UV radiation resistance-associated gene protein-like	150.151	-0.012	-0.308	1.472	-0.005
GB43470	regulator of nonsense transcripts 2	150.085	-0.313	-0.047	-0.055	0.001
GB43215	actin-related protein 6-like isoform X2	149.654	0.306	0.078	0.043	0.062
GB51005	N-alpha-acetyltransferase 20-like isoform X2	149.618	0.015	0.043	0.171	0.099
GB50360	zinc finger CCCH domain-containing protein 11A-like	149.264	-0.542	-0.065	0.122	-0.111
GB41340	uncharacterized protein LOC100579034 isoform X2	149.233	-0.295	0.138	-0.034	-0.017
GB55701	putative aldehyde dehydrogenase family 7 member A1 homolog isoform 2	149.106	0.525	-0.091	0.039	-0.108
GB51539	28S ribosomal protein S17, mitochondrial isoform X2	149.100	-0.119	-0.063	-0.034	0.068
GB50658	DNA primase large subunit	148.856	-0.007	0.197	-0.085	0.243
GB53251	N-alpha-acetyltransferase 25, NatB auxiliary subunit isoform X2	148.837	0.002	0.154	-0.978	0.062
GB43300	RNA-binding protein squid-like isoform X6	148.821	-0.128	0.090	0.069	0.098
GB50482	uncharacterized protein LOC724971 isoform X2	148.549	-0.044	-0.240	0.002	0.086
GB54657	Rad54 protein isoform X2	148.453	-0.208	-0.178	0.101	0.168
GB52541	probable cation-transporting ATPase 13A1-like	148.285	-0.210	0.139	-0.027	-0.011
GB46262	IQ motif and SEC7 domain-containing protein 2 isoform X5	148.155	-0.576	0.000	0.069	0.010
GB53005	gem-associated protein 8-like	147.970	0.045	-0.012	-0.057	0.073
GB50935	mitochondrial chaperone BCS1-like	147.842	-0.133	-0.045	-0.033	0.128
GB51271	protein Peter pan-like	147.719	-0.277	0.094	0.194	0.141
GB52980	uncharacterized protein LOC552428	147.645	-0.029	-0.069	0.051	-0.011
GB45566	protein EFR3 homolog cmp44E isoformX1	147.605	0.033	0.049	0.146	0.239
GB44347	TBC1 domain family member 16 isoform X2	147.584	-0.126	-0.103	0.251	0.111
GB49949	WD repeat domain phosphoinositide-interacting protein 2-like isoform X2	147.456	-0.013	0.094	0.134	-0.040

(continued)

Gene	Name	k	am_fc	bt_fc	lf_fc	ln_fc
GB51634	glucose 1,6-bisphosphate synthase isoform X2	147.312	0.197	-0.064	-0.859	0.125
GB50078	ATP-dependent RNA helicase abstrakt	147.275	0.000	0.143	0.054	0.064
GB54909	dynamin-binding protein-like isoform X2	147.189	-0.063	-0.013	0.105	0.036
GB40113	UBX domain-containing protein 4-like isoform 1	147.057	-0.284	-0.030	-0.100	0.037
GB43858	INO80 complex subunit E-like	147.047	-0.107	0.033	0.018	-0.003
GB47748	two pore calcium channel protein 1	146.924	-0.335	-0.153	0.000	0.080
GB53915	dimethyladenosine transferase 1, mitochondrial	146.920	-0.504	-0.074	0.074	0.032
GB43625	RNA methyltransferase-like protein 1-like	146.919	-0.249	0.139	-0.114	0.033
GB49161	probable aminoacyl tRNA synthase complex-interacting multifunctional protein 2 isoform X2	146.895	-0.550	-0.034	0.053	0.060
GB47812	LOW QUALITY PROTEIN: regulatory-associated protein of mTOR	146.889	-0.163	0.018	0.064	-0.118
GB46894	pre-mRNA-splicing factor SYF1-like isoformX1	146.864	-0.029	0.244	-0.045	0.176
GB43553	5'-AMP-activated protein kinase catalytic subunit alpha-2 isoform X3	146.695	-0.160	-0.007	0.123	0.082
GB49789	28S ribosomal protein S29, mitochondrial isoformX1	146.636	-0.621	-0.020	0.340	0.190
GB49570	centaurin-gamma-1A	146.577	-0.192	-0.051	0.127	0.143
GB53650	glycerol kinase-like isoform X2	146.470	-0.518	-0.203	-0.050	-0.026
GB44176	integrator complex subunit 8	146.406	0.208	-0.047	0.118	0.097
GB44991	RNA-binding protein cabeza-like isoform X1	146.402	-0.063	-0.038	0.098	-0.004
GB45122	mitochondrial assembly of ribosomal large subunit protein 1-like	146.396	-0.415	0.019	0.491	-0.004
GB52910	octopamine receptor	146.272	-0.773	-0.024	-0.880	0.160
GB50740	ubiquitin-conjugating enzyme E2 S-like isoform X2	146.187	0.264	0.118	-0.531	0.159
GB42216	hepatoma-derived growth factor-related protein 2-like isoform 2	146.166	0.006	0.059	-0.040	0.048
GB47964	zinc finger protein 543-like isoform X1	146.089	0.201	0.014	0.131	0.013
GB43074	phosphatidylinositol 4,5-bisphosphate 3-kinase catalytic subunit delta isoform-like isoform X1	146.087	-0.084	-0.016	0.132	0.134
GB53359	alkylated DNA repair protein alkB homolog 1	145.953	-0.022	0.031	-0.041	-0.004
GB54342	DEAD-box helicase Dbp80 isoform X2	145.760	0.205	0.038	-0.107	0.130
GB47417	mediator of RNA polymerase II transcription subunit 15 isoform X2	145.743	-0.140	-0.020	0.132	0.016
GB49634	trafficking protein particle complex subunit 12-like	145.631	-0.008	0.077	0.214	0.023
GB43163	nuclear factor related to kappa-B-binding protein	145.400	-0.284	0.020	-1.020	-0.049
412933	geranylgeranyl transferase type-1 subunit beta isoform X1	145.150	0.369	-0.075	-0.012	-0.072
GB48982	uncharacterized protein LOC413055	145.140	-0.293	0.008	3.650	-0.019
GB40301	aldose 1-epimerase-like isoform X2	145.036	-0.115	-0.011	0.071	0.043
GB18555	tether containing UBX domain for GLUT4	144.695	-0.384	0.051	-0.053	0.027
GB54831	nuclear transcription factor Y subunit gamma-like isoform X1	144.336	-0.120	-0.093	0.025	-0.098
GB45689	mediator of RNA polymerase II transcription subunit 25 isoform X3	144.074	0.018	0.109	-0.034	0.116
GB48625	probable ATP-dependent RNA helicase spindle-E isoform X2	144.018	0.222	0.161	-0.111	-0.143
GB52677	forkhead box protein K1-like isoform X2	143.982	0.035	0.150	0.051	0.062
GB50699	protein SET-like isoform X1	143.935	0.487	0.040	0.147	0.187
GB45039	probable RNA polymerase II nuclear localization protein SLC7A6OS-like	143.908	-0.015	0.258	0.096	0.098
GB54951	ubiquitin carboxyl-terminal hydrolase 46-like isoform X1	143.785	-0.194	0.129	-0.032	0.073
GB42918	protein NipSnap	143.609	0.389	-0.058	0.048	0.012
GB50069	vacuolar protein sorting-associated protein 51 homolog	143.558	-0.077	0.033	0.026	0.175
GB40556	WD repeat-containing protein 61-like	143.504	-0.197	-0.056	0.084	0.194
GB40798	serine/arginine-rich splicing factor 7 isoform X1	143.484	0.105	0.064	0.680	0.068
GB52737	vacuolar protein sorting-associated protein 41 homolog isoform X2	143.398	0.094	-0.051	0.116	-0.080
GB45491	G patch domain and ankyrin repeat-containing protein 1 homolog	143.392	-0.239	-0.061	0.094	0.090
GB42352	elongation factor Tu GTP-binding domain-containing protein 1-like, transcript variant X4	143.328	0.037	0.059	0.160	-0.069
GB50688	26S proteasome non-ATPase regulatory subunit 7-like	143.312	0.165	0.046	-0.080	0.049
GB52904	nuclear hormone receptor HR96 isoform X2	143.160	-0.254	-0.024	0.061	0.028
GB40891	RING finger protein 157-like isoform X3	143.094	-0.013	0.090	-0.049	-0.003
GB44930	ell-associated factor Eaf-like isoform X1	143.043	0.055	0.143	-0.108	0.005

(continued)

Gene	Name	k	am_fc	bt_fc	lf_fc	ln_fc
102656131	biogenesis of lysosome-related organelles complex 1 subunit 2-like	142.988	-0.018	-0.213	0.152	-0.164
GB44543	DNA polymerase alpha subunit B isoform X2	142.941	0.137	-0.092	0.046	0.112
GB40452	brefeldin A-inhibited guanine nucleotide-exchange protein 1	142.897	-0.260	0.062	0.123	-0.043
GB43856	dnaJ homolog subfamily C member 11-like	142.832	0.136	0.106	-0.086	0.074
GB42681	mucin-5AC-like isoform X3	142.483	-0.259	0.094	0.929	0.097
GB51143	protein downstream neighbor of son homolog	142.459	-0.228	0.045	-0.090	-0.023
GB45656	AKT-interacting protein-like	142.298	0.124	-0.101	0.097	0.111
GB55943	protein FAM114A2-like	142.218	-0.563	-0.060	-0.208	0.030
GB41485	WD repeat-containing protein 55 homolog	142.125	-0.475	-0.007	-0.073	0.188
GB55659	tudor domain-containing protein 3-like isoform X1	142.028	-0.252	0.175	0.065	0.130
GB55811	protein MTO1 homolog, mitochondrial-like isoform 1	141.867	-0.186	-0.008	0.083	0.091
GB54571	FACT complex subunit Ssrp1	141.755	-0.303	0.089	-0.069	0.027
GB48157	G patch domain-containing protein 1 homolog	141.737	-0.170	0.233	0.165	-0.084
GB46247	eukaryotic translation initiation factor 3 subunit H	141.615	0.074	0.121	-0.052	-0.045
GB53825	transcription initiation factor TFIID subunit 2	141.574	-0.718	-0.010	0.112	0.084
GB51716	60S ribosomal protein L23a	141.375	0.165	0.055	0.118	-0.086
726768	probable ATP-dependent RNA helicase DDX17-like	141.344	-0.338	-0.040	0.145	-0.008
GB53852	paired amphipathic helix protein Sin3a isoform X3	141.337	-0.307	-0.105	0.200	0.011
GB52306	dnaJ homolog subfamily C member 13	141.074	0.061	0.005	-0.008	0.129
GB46520	vacuolar protein sorting-associated protein 26B	140.985	-0.108	-0.098	-0.132	-0.058
GB48169	hamartin isoform X3	140.911	-0.343	0.003	0.109	-0.019
GB41781	pontin protein isoform 1	140.590	0.324	0.172	0.027	0.006
GB42016	intron-binding protein aquarius	140.511	-0.069	0.074	-0.096	-0.148
GB43219	uncharacterized protein LOC724290	140.457	-0.858	0.184	0.096	0.129
GB50141	erythroid differentiation-related factor 1-like isoform X1	140.411	0.053	0.066	0.087	0.004
GB46363	15-hydroxyprostaglandin dehydrogenase [NAD(+)]-like isoform X2	140.408	-0.210	0.185	0.124	-0.072
GB46707	vacuolar protein-sorting-associated protein 25	140.385	0.004	-0.039	-0.060	0.161
GB41257	probable protein phosphatase CG10417-like	140.191	0.199	0.037	-0.080	0.061
GB46963	probable ATP-dependent RNA helicase DHX36-like isoform X2	140.063	-0.071	0.069	0.150	-0.007
GB43894	histone deacetylase 3 isoform 1	139.863	-0.262	0.115	0.073	-0.121
GB49666	dnaJ homolog subfamily C member 8-like	139.618	-0.559	0.052	0.117	0.100
GB53001	uncharacterized protein LOC100577272	139.583	-0.060	-0.023	0.087	0.172
GB47260	zinc finger protein 598-like isoform X2	139.535	-0.602	0.059	-0.125	0.012
GB42230	protein IWS1 homolog isoformX2	139.492	-0.098	0.188	-0.067	0.062
GB51525	synergin gamma-like	139.422	-0.275	-0.072	-0.051	0.087
GB48115	major facilitator superfamily domain-containing protein 6	139.377	0.182	0.009	0.074	0.030
GB52726	ras association domain-containing protein 8-like isoform X2	139.261	0.067	-0.078	0.032	-0.036
GB49089	integrator complex subunit 4-like	139.213	0.007	0.200	-0.082	-0.036
GB53333	V-type proton ATPase catalytic subunit A-like isoform X3	139.135	0.063	-0.058	0.035	0.042
GB51071	putative deoxyribose-phosphate aldolase-like isoform X2	139.083	-0.152	-0.056	-0.036	-0.099
GB47882	abl interactor 2	139.082	0.183	0.055	0.191	-0.045
GB53177	GPN-loop GTPase 1-like	138.834	-0.393	-0.037	0.008	0.063
GB43186	symplekin	138.787	0.034	0.095	0.020	-0.042
GB48845	probable rRNA-processing protein EBP2 homolog	138.660	-0.482	0.131	-0.079	0.115
GB53239	HEAT repeat-containing protein 6-like	138.658	-0.173	-0.041	-0.130	0.331
GB45173	autism susceptibility gene 2 protein-like isoform X2	138.640	-0.236	-0.002	0.099	0.024
GB52774	glycylpeptide N-tetradecanoyltransferase 1	138.581	0.396	-0.013	0.070	0.027
GB49612	protein real-time-like isoform X1	138.473	-0.073	-0.056	0.037	-0.049
GB44524	peptidyl-prolyl cis-trans isomerase-like 2-like	138.429	-0.171	0.129	0.017	0.002
GB47813	malonyl-CoA decarboxylase, mitochondrial-like	138.416	-0.540	-0.122	0.033	0.051
GB51822	Sip1/TFIP11 interacting protein	138.412	0.082	0.114	0.000	0.150
GB50347	LOW QUALITY PROTEIN: polymerase delta-interacting protein 2-like	138.219	-0.051	0.063	0.118	0.118
GB54684	ADIPOR-like receptor CG5315-like isoform X2	138.166	-0.013	-0.117	-0.101	0.808
GB44103	protein LTV1 homolog	137.904	-0.782	0.120	-0.056	0.169
GB41865	DNA-binding protein Ets97D homolog	137.821	0.139	0.129	-0.063	0.139
GB43486	uncharacterized protein LOC552788	137.742	-0.205	-0.058	0.179	0.153
GB53328	DNA/RNA-binding protein KIN17	137.725	-0.245	-0.290	0.071	0.081
GB42234	cat eye syndrome critical region protein 5-like isoform X2	137.716	-0.431	-0.067	-0.088	0.071

(continued)

Gene	Name	k	am__fc	bt__fc	lf__fc	ln__fc
GB40801	thioredoxin-like protein 4A-like isoform X1	137.667	0.048	0.046	0.176	0.142
GB40473	cytoplasmic phosphatidylinositol transfer protein 1	137.659	0.071	-0.061	0.305	0.048
102655259	5-methylcytosine rRNA methyltransferase NSUN4-like isoform X1	137.648	-0.667	-0.043	-0.637	0.114
GB48928	MATH and LRR domain-containing protein PFE0570w-like	137.616	0.055	0.155	-0.573	0.079
GB40745	synaptosomal-associated protein 29	137.441	-0.348	0.073	0.083	0.038
GB43288	ribonuclease P protein subunit p21-like	137.401	0.191	-0.004	0.810	0.167
GB54916	protein Mo25-like isoform X3	137.362	0.255	-0.027	0.165	-0.340
GB46909	basic proline-rich protein-like isoform X2	137.237	-0.212	0.106	0.072	-0.065
GB49643	probable enoyl-CoA hydratase, mitochondrial-like	137.233	0.248	0.017	-0.087	-0.080
GB54220	nuclear distribution protein nudE-like 1-A-like isoform X1	137.197	-0.231	-0.003	0.164	0.098
GB46016	transmembrane protein 131-like	136.872	0.110	-0.005	0.126	0.039
GB46911	programmed cell death protein 10-like	136.857	0.081	0.005	0.012	0.117
GB52852	zinc finger protein 879-like isoform X2	136.815	-0.168	0.009	0.034	0.060
GB52080	probable GDP-L-fucose synthase isoform X2	136.788	0.008	0.079	0.100	0.076
GB44642	F-box/WD repeat-containing protein 9 isoform 1	136.760	0.055	-0.116	-0.023	0.054
GB41769	actin-related protein 8 isoform 1	136.721	0.312	0.072	-0.006	0.092
GB45357	protein EMSY-like isoform X2	136.706	-0.506	0.139	0.080	0.154
725378	nucleolar GTP-binding protein 2	136.550	-0.387	0.139	-0.143	0.033
GB41444	N-acetyltransferase 10-like	136.506	-0.626	0.033	0.067	0.038
GB51692	mediator of RNA polymerase II transcription subunit 14 isoform X2	136.458	0.013	-0.078	0.078	0.518
GB48402	zinc finger C4H2 domain-containing protein-like isoform X3	136.345	0.272	-0.013	0.022	0.099
GB49491	anaphase-promoting complex subunit 1	136.277	-0.421	-0.014	0.155	0.241
GB53777	beta-1,4-galactosyltransferase 7 isoform X2	136.140	0.006	-0.141	0.113	0.151
GB53859	arf-GAP with coiled-coil, ANK repeat and PH domain-containing protein 2 isoform X1	136.121	-0.180	0.059	0.081	-0.015
GB41711	uncharacterized protein LOC408493	136.083	-0.251	0.078	0.118	0.049
GB48781	nuclear nucleic acid-binding protein C1D-like isoformX2	136.022	-0.244	0.095	0.074	0.039
GB44345	non-histone protein 10-like	135.776	-0.204	0.323	-0.013	-0.019
GB51123	UPF0047 protein C4A8.02c-like	135.667	0.051	0.003	0.068	-0.007
GB53236	retrograde Golgi transport protein RGP1 homolog isoform X1	135.639	-0.205	0.007	-0.015	0.061
GB41909	bluestreak isoform X1	135.629	-0.318	-0.126	0.111	0.032
GB52352	WD repeat and HMG-box DNA-binding protein 1-like isoform X1	135.598	-0.755	0.071	-0.043	0.086
GB42770	male-specific lethal 3 homolog isoform X1	135.588	-0.576	0.212	0.002	0.205
GB50801	serine/threonine-protein kinase VRK1-like	135.579	-0.117	0.015	0.047	0.150
GB46615	rho GTPase-activating protein 1-like isoformX2	135.553	-0.226	0.203	0.122	-0.068
GB46606	peptidyl-prolyl cis-trans isomerase FKBP8-like isoform X3	135.420	-0.233	-0.175	-0.166	0.095
GB50281	transducin (beta)-like 3 isoform X1	135.397	-0.284	0.016	0.118	0.149
GB45280	mitochondrial import inner membrane translocase subunit TIM44-like isoform 1	135.337	-0.450	0.141	-0.017	0.179
GB41884	calcineurin-binding protein cabin-1-like isoform X2	135.223	-0.120	0.126	-0.102	-0.109
GB51658	ribosome maturation protein SBDS-like	135.221	0.129	0.109	0.042	0.167
GB42846	cullin-4B-like	135.218	-0.526	0.057	0.131	-0.642
GB51910	zinc finger CCCH-type with G patch domain-containing protein-like	135.176	-0.819	0.143	0.058	0.001
GB53979	SEC23-interacting protein-like isoformX1	135.058	-0.050	0.027	0.095	-0.026
GB42420	coiled-coil domain-containing protein 102A-like	135.048	-0.643	0.140	0.014	0.114
102655679	DNA-directed RNA polymerase III subunit RPC7-like	135.039	-0.026	0.238	0.183	0.076
GB55281	TBC1 domain family member 20-like isoform X2	134.964	0.067	-0.143	0.080	0.066
GB47428	synaptojanin-1	134.963	-0.127	0.217	0.172	-0.036
GB41469	septin-2 isoform X1	134.757	0.102	-0.063	0.047	0.097
GB43626	uncharacterized protein LOC409331 isoform X3	134.686	0.062	0.280	-0.070	-0.009
GB54680	LOW QUALITY PROTEIN: vinculin	134.668	-0.257	-0.112	0.193	0.135
GB55854	ubiquitin-protein ligase E3B-like isoform X3	134.594	-0.501	-0.067	0.077	-0.029
GB49124	myotubularin-related protein 2 isoform X3	134.536	0.086	0.122	-0.057	-0.041
GB46645	ras-related protein Rab-21	134.381	-0.148	-0.019	0.072	-0.011
GB53931	laminin subunit alpha-1-like isoform X3	134.352	-0.440	0.013	0.713	-0.640
GB49344	serine/threonine-protein kinase pelle isoform X1	134.236	-0.220	-0.014	0.181	0.091
GB52757	uncharacterized protein LOC408473 isoform 1	134.221	-0.125	-0.421	0.131	0.029

(continued)

Gene	Name	k	am__fc	bt__fc	lf__fc	ln__fc
GB45126	DNA topoisomerase 1 isoform 2	134.183	-0.246	0.133	-0.162	0.086
GB49961	WD repeat-containing protein 44-like isoform X1	134.072	-0.088	-0.044	0.179	0.161
409254	protein RTF2 homolog	134.048	-0.252	-0.298	0.051	0.224
GB49520	BRCA1-A complex subunit BRE-like	134.037	-0.161	0.107	0.109	0.151
GB44327	protein SERAC1-like isoform X2	134.020	0.068	0.076	0.120	0.034
GB44429	DNA damage-binding protein 1-like	133.865	-0.013	0.118	0.072	0.076
GB52421	protein lines isoform 1	133.783	0.015	-0.019	0.074	-0.047
GB47594	golgin-45-like	133.763	-0.223	0.057	-0.039	-0.050
GB41775	integrator complex subunit 5-like isoform X2	133.678	-0.017	0.013	-0.070	-0.170
GB52253	protein PRRC2C-like isoform X2	133.587	-0.542	-0.077	4.658	0.078
GB53133	ral GTPase-activating protein subunit beta-like isoform X7	133.429	-0.104	-0.075	0.175	-0.213
GB46654	ribosomal L1 domain-containing protein CG13096-like	133.307	-0.240	-0.010	-0.026	0.037
GB55591	microtubule-associated protein futsch-like isoform X1	133.307	-0.463	-0.261	-1.150	-0.357
GB49315	protein lin-54 homolog isoform X2	133.250	-0.285	0.162	0.045	0.466
GB51597	DNA repair protein complementing XP-A cells homolog	133.233	-0.222	0.086	-0.137	0.097
GB47436	acetyl-CoA acetyltransferase, mitochondrial-like isoform X1	133.185	0.042	-0.076	0.025	-0.162
GB49033	glutamyl-tRNA(Gln) amidotransferase subunit A, mitochondrial isoform X1	133.079	0.262	-0.012	-0.032	-0.089
GB53439	putative sodium-coupled neutral amino acid transporter 10-like isoform X2	133.067	-0.642	-0.067	-0.028	0.670
GB46881	COMM domain-containing protein 5-like isoform X2	133.046	-0.189	0.203	-0.025	0.165
GB47238	UPF0396 protein CG6066-like isoform 1	133.023	-0.172	0.138	-0.024	0.121
GB50269	very-long-chain (3R)-3-hydroxyacyl-[acyl-carrier protein] dehydratase 3 isoform X2	132.947	-0.314	0.107	0.113	0.041
GB46627	paraplegin-like	132.923	-0.645	-0.009	0.022	0.126
GB41640	zucchini	132.809	-0.080	0.177	-0.053	0.077
GB46691	histone-lysine N-methyltransferase, H3 lysine-79 specific-like isoform X2	132.779	-0.140	0.129	0.070	-0.079
GB53707	density-regulated protein-like isoform X1	132.617	-0.053	0.099	-0.024	0.010
GB40634	upstream stimulatory factor 1-like	132.535	-0.068	0.001	0.168	0.136
GB45279	RING finger protein PFF0165c-like	132.383	-0.560	0.044	-0.081	0.173
GB46216	protein RFT1 homolog isoform X1	132.315	-0.094	-0.026	-0.028	0.057
GB48370	ATP-binding cassette sub-family B member 7, mitochondrial isoform X1	132.291	-0.591	-0.163	0.059	0.140
GB53960	spliceosome-associated protein CWC15 homolog isoform 1	132.275	0.212	0.132	-0.034	-0.028
GB49680	LIM domain kinase 1 isoform X2	132.272	-0.053	0.155	0.182	0.105
GB53882	IST1 homolog isoform X4	132.106	-0.274	-0.010	-0.003	0.188
GB49762	nuclear pore complex protein Nup50 isoform X1	131.998	-0.293	0.064	-0.060	-0.003
GB55439	probable ATP-dependent RNA helicase DDX49-like	131.929	-0.552	-0.011	0.173	0.090
GB53068	cysteine and histidine-rich domain-containing protein	131.789	0.053	0.132	0.102	0.170
GB53618	uncharacterized protein LOC724843 isoform X1	131.720	-0.722	-0.157	0.018	-0.079
GB55183	ankyrin repeat domain-containing protein SOWAHB-like isoform X5	131.493	-0.724	0.032	0.265	-0.207
GB54672	39S ribosomal protein L21, mitochondrial-like	131.490	0.014	0.126	-0.053	0.210
GB47596	general transcription factor 3C polypeptide 1-like	131.455	-0.679	-0.044	0.198	-0.047
GB40909	sorting nexin-29-like isoform X4	131.453	-0.133	-0.216	0.162	-0.053
GB49032	CDP-diacylglycerol–glycerol-3-phosphate 3-phosphatidyltransferase, mitochondrial-like isoform X2	131.394	0.408	-0.103	-0.014	0.010
GB47576	NADH dehydrogenase [ubiquinone] 1 alpha subcomplex assembly factor 5-like isoform 2	131.384	-0.147	-0.062	-0.106	0.205
GB44062	tyrosine-protein kinase Fps85D-like isoform X4	131.346	-0.095	-0.052	0.160	0.126
GB52645	pinin	130.952	-0.454	0.116	0.141	0.096
GB55626	peptidyl-prolyl cis-trans isomerase D-like isoform X1	130.909	-0.307	0.111	-0.008	0.088
GB46257	myotubularin-related protein 14 isoform 1	130.821	-0.075	0.263	0.122	0.127
GB45457	vacuolar protein sorting-associated protein 53 homolog	130.762	-0.268	0.130	0.167	-0.012
GB53939	mitotic checkpoint protein BUB3	130.527	-0.119	0.120	-0.020	0.091
GB54592	tudor domain-containing protein 7-like isoform X1	130.491	0.063	0.036	0.143	0.126
GB46736	probable ubiquitin carboxyl-terminal hydrolase FAF-X isoform X6	130.420	-0.447	0.110	-0.812	-0.010
GB41770	28S ribosomal protein S22, mitochondrial	130.376	0.197	0.125	0.080	-0.013
GB51134	serine/threonine-protein kinase mig-15 isoform X13	130.269	-0.300	-0.041	0.104	0.000
GB46048	transmembrane protein 62-like, transcript variant X2	130.196	-0.546	0.007	0.051	0.404

(continued)

Gene	Name	k	am_fc	bt_fc	lf_fc	ln_fc
GB47991	biogenesis of lysosome-related organelles complex 1 subunit 1-like	130.149	0.052	0.040	0.134	0.070
GB53820	gametogenetin-binding protein 2-like isoform X1	130.140	0.036	-0.041	-0.079	0.131
GB51395	rabenosyn-5	130.110	-0.187	0.142	0.054	0.074
GB45545	NADH dehydrogenase [ubiquinone] 1 beta subcomplex subunit 9-like	130.031	-0.304	-0.174	0.040	0.168
102655877	beta-1,3-galactosyltransferase 6-like	129.924	-0.349	0.071	0.243	0.033
GB50897	glycoprotein 150 isoform X1	129.776	-0.244	-0.142	0.016	-0.029
GB51542	PH-interacting protein isoform X1	129.754	-0.160	-0.139	0.168	0.129
GB46740	N-acetylgalactosaminyltransferase 6-like isoform 1	129.692	-0.218	0.004	0.023	-0.084
GB43873	sin3 histone deacetylase corepressor complex component SDS3-like isoform 1	129.581	0.120	0.109	-0.039	0.254
GB47747	60S ribosomal protein L6	129.557	-0.004	-0.018	0.197	0.161
GB55492	importin-9 isoform X2	129.237	0.101	0.160	-0.226	0.021
GB42721	aspartate–tRNA ligase, mitochondrial-like	129.197	-0.063	-0.009	0.300	0.101
GB50764	coiled-coil domain-containing protein 124-like	129.117	-0.130	0.110	-0.179	0.113
GB51067	akirin-2 isoform 2	128.889	0.369	0.110	-0.027	-0.104
GB44890	regulator of nonsense transcripts 1 isoform X1	128.823	-0.204	0.014	0.018	-0.104
GB49402	erlin-1-like	128.712	-0.389	0.108	0.015	0.220
GB51884	calcyclin-binding protein-like	128.465	-0.063	0.149	0.075	0.028
GB45342	transcriptional adapter 1-like isoform 1	128.454	-0.203	0.163	-0.017	0.049
GB41304	probable glutamine–tRNA ligase	128.430	-0.078	-0.007	-0.110	-0.132
GB47846	WD repeat-containing protein 48-like isoform X2	128.270	0.105	0.045	0.076	0.094
GB41693	DNA topoisomerase 3-alpha-like	127.956	-0.065	0.021	-0.060	0.185
GB43868	dnaJ homolog subfamily C member 1-like isoform X1	127.950	-0.394	0.152	-0.062	-0.115
GB40091	LIM domain-containing protein jub-like	127.854	0.071	0.009	0.048	0.094
GB47960	moesin/ezrin/radixin homolog 1 isoform X15	127.810	0.144	-0.059	0.048	0.021
GB49354	uncharacterized protein PF11_0213-like isoform X1	127.780	-0.246	0.233	-0.019	0.151
GB42071	GATA zinc finger domain-containing protein 7-like	127.757	-0.221	-0.008	0.025	0.046
GB46378	galactosylgalactosylxylosylprotein 3-beta-glucuronosyltransferase I	127.692	0.211	-0.007	0.832	0.048
GB55422	maspardin-like isoform X3	127.634	0.003	0.138	0.103	0.065
GB51427	bromodomain-containing protein DDB_G0280777	127.634	-0.496	0.215	-0.102	0.158
GB55570	transmembrane protein 53-like isoform X3	127.590	-0.362	-0.164	0.437	0.106
GB43260	macrophage erythroblast attacher-like isoform 1	127.361	0.045	0.189	0.032	0.035
GB40098	cyclin-H	127.309	-0.042	0.119	0.528	0.234
GB45037	beta-lactamase-like protein 2-like isoform X2	127.289	-0.572	0.003	-0.356	-0.445
102654890	activating signal cointegrator 1-like isoform X2	127.198	-0.228	0.024	-0.195	0.114
GB40491	translation initiation factor eIF-2B subunit gamma isoform X1	127.157	-0.025	-0.077	0.077	0.063
GB44069	syntaxin-16 isoform X1	127.067	-0.786	0.214	0.423	0.150
102656215	NADH dehydrogenase [ubiquinone] 1 alpha subcomplex assembly factor 3-like	127.031	-0.616	0.112	0.103	0.069
GB50515	ankycorbin-like isoform X1	126.984	-0.479	0.064	0.169	0.006
GB43301	ras GTPase-activating protein-binding protein 2 isoform X1	126.964	0.058	0.011	0.078	0.161
GB49500	zinc finger protein 808-like, transcript variant X2	126.938	-0.610	0.180	-0.049	0.074
GB53184	replication protein A 32 kDa subunit	126.826	-0.040	0.180	0.123	0.074
GB44660	probable ribonuclease P/MRP protein subunit POP5-like isoform 2	126.749	-0.165	0.025	0.043	0.039
GB52658	general transcription factor 3C polypeptide 5-like	126.566	-0.327	0.099	0.171	0.024
GB51763	UPF0505 protein C16orf62 homolog isoform X1	126.450	-0.191	-0.067	0.109	-0.032
GB52994	transcription elongation factor B polypeptide 1 isoform X5	126.420	-0.013	0.115	0.150	0.170
GB46268	plasminogen activator inhibitor 1 RNA-binding protein-like isoform X1	126.399	0.216	0.109	-0.015	-0.008
GB46042	TBC1 domain family member 23-like	126.389	0.166	0.047	-0.015	0.145
GB47271	adenylate cyclase type 9-like isoform X2	126.322	-0.138	-0.020	-0.026	0.100
GB53836	protein spinster-like isoformX1	126.246	0.045	-0.150	0.006	0.015
GB40467	ubiquitin-conjugating enzyme E2 J2-like	126.188	-0.020	0.075	-0.066	0.023
726689	prefoldin subunit 1-like isoform X1	126.172	0.061	-0.039	0.001	0.288
GB47539	acidic mammalian chitinase isoform X2	125.996	0.127	0.013	0.203	0.159
GB40872	josephin-like protein-like isoform X1	125.853	-0.406	0.007	0.037	0.370
GB54340	GTP-binding protein 10 homolog	125.807	-0.180	0.101	0.056	0.085

(continued)

Gene	Name	k	am_fc	bt_fc	lf_fc	ln_fc
GB54928	tRNA (guanine(10)-N2)-methyltransferase homolog	125.781	-0.169	0.045	-0.012	0.271
GB46915	serine/threonine-protein phosphatase 2A activator isoform X2	125.748	0.076	0.084	0.149	0.081
725981	protein TIPIN homolog	125.745	0.064	-0.048	-0.032	0.107
GB48417	probable E3 ubiquitin-protein ligase RNF144A-like isoform X1	125.697	-0.377	-0.020	0.046	0.107
GB53196	uncharacterized protein LOC412107	125.674	-0.351	-0.143	-0.011	0.165
GB48351	succinate-semialdehyde dehydrogenase, mitochondrial	125.532	0.214	0.063	-0.084	-0.023
GB51037	CTD small phosphatase-like protein 2-like isoform X1	125.445	0.020	-0.041	-0.020	0.102
GB49221	phosphomevalonate kinase-like	125.423	0.511	-0.223	-0.135	0.008
GB53663	GTPase Era, mitochondrial-like isoform X1	125.412	-0.408	-0.062	0.093	0.053
GB52918	serine protease HTRA2, mitochondrial	125.253	0.027	0.031	-0.006	-0.059
GB44144	probable phenylalanine–tRNA ligase, mitochondrial	125.240	-0.702	0.047	0.134	0.244
GB47272	nucleoporin Nup37-like isoform X2	125.219	0.456	0.459	-0.125	0.175
GB51721	zinc finger protein 800-like isoform X2	125.145	-0.896	0.157	0.120	0.198
GB47746	AP-3 complex subunit beta-2-like isoform X3	125.135	-0.334	0.100	-0.002	0.098
GB47577	SAP domain-containing ribonucleoprotein-like isoform X3	124.963	-0.089	0.209	-0.049	0.129
GB48122	MAU2 chromatid cohesion factor homolog isoform X1	124.957	-0.203	0.056	0.033	0.035
GB44830	cleavage stimulation factor subunit 2 isoform X2	124.921	0.285	0.173	0.106	-0.156
GB40017	enhancer of mRNA-decapping protein 4	124.835	-0.141	0.152	-0.105	0.023
GB46274	arginine/serine-rich coiled-coil protein 2-like isoform X2	124.650	-0.327	-0.060	0.188	-0.264
GB49777	seipin-like isoform 1	124.648	0.013	0.023	-0.060	0.141
GB55506	histidine–tRNA ligase, cytoplasmic-like isoform X5	124.446	0.171	0.041	0.062	0.021
GB49556	DNA replication licensing factor Mcm2-like isoform X1	124.403	-0.642	0.029	0.029	0.029
GB42786	microtubule-associated protein RP/EB family member 1-like isoform X4	124.385	0.372	0.108	0.080	0.123
GB54354	uncharacterized protein DDB_G0274915-like isoform X2	124.308	0.108	0.036	-0.502	0.045
GB54976	ER membrane protein complex subunit 8/9 homolog	124.294	0.135	-0.059	-0.012	0.051
GB47007	actin-related protein 5	124.267	-0.100	-0.048	0.022	0.218
GB41252	cell cycle checkpoint protein RAD17-like isoform X2	123.974	0.096	0.058	-0.041	0.040
GB53677	clavesin-2-like isoform X3	123.879	-0.558	0.160	0.184	-0.099
100577180	uncharacterized protein LOC100577180	123.803	0.009	0.177	0.039	0.100
GB53659	ribosome biogenesis protein WDR12 homolog isoform X1	123.796	0.198	0.214	-1.357	0.047
GB48223	peroxisomal targeting signal 1 receptor-like isoform X1	123.778	-0.157	-0.006	-0.004	0.038
GB55003	protein phosphatase 1 regulatory subunit 16A-like isoform X7	123.775	-0.107	0.073	0.104	0.132
GB50727	prefoldin subunit 2-like	123.681	0.257	0.013	0.076	0.051
GB44904	serine/threonine-protein phosphatase 4 regulatory subunit 2	123.618	-0.944	0.077	-0.024	-0.037
GB46751	nuclear export mediator factor NEMF homolog isoform X2	123.577	0.001	0.095	0.040	-0.029
GB45272	neuroligin-4, Y-linked isoform X2	123.572	-0.501	0.003	-0.039	-0.026
GB46204	protein cereblon-like	123.521	0.139	-0.076	-0.013	0.143
GB41850	hemocytin isoform X3	123.443	-0.006	0.021	-0.404	0.205
GB45716	WD repeat and FYVE domain-containing protein 2-like	123.293	-0.453	-0.121	0.060	0.134
GB54287	protein virilizer	123.226	-0.022	0.068	0.005	-0.062
GB41213	intraflagellar transport protein 20 homolog isoform X1	123.182	-0.120	0.006	0.008	-0.014
GB40955	glutamate–cysteine ligase regulatory subunit isoform X2	123.091	0.128	-0.032	-0.022	-0.087
102654121	protein YIPF5-like	122.983	0.254	0.136	0.005	-0.126
GB49593	cytosolic carboxypeptidase-like protein 5-like isoform X1	122.944	-0.590	-0.279	0.101	0.236
GB51087	pyridoxal kinase-like	122.915	-0.579	-0.032	0.047	0.096
GB53726	protein CNPPD1-like	122.884	-0.081	0.155	0.043	0.119
GB54579	regulator of nonsense transcripts 3B isoform 1	122.849	-0.518	0.041	0.183	-0.004
GB51158	uncharacterized protein LOC726321	122.826	-0.169	-0.055	0.076	0.166
GB47633	serine/threonine-protein kinase D3 isoformX2	122.805	-0.417	0.038	0.054	0.030
GB51508	probable elongator complex protein 3-like isoform X3	122.744	0.086	0.222	0.722	0.082
GB48204	CD109 antigen isoform X2	122.704	-0.190	-0.087	-0.016	-0.181
GB44906	ubiquitin conjugation factor E4 B isoform X1	122.692	-0.374	-0.011	-0.129	0.040
GB49538	inhibitor of growth protein 1-like	122.582	-0.557	0.219	-0.088	0.098
GB45568	uncharacterized protein C2orf42 homolog	122.281	-0.609	0.154	0.075	0.114
GB42079	protein arginine N-methyltransferase 8	122.245	0.573	-0.033	0.014	-0.008
GB48010	protein ENL-like isoform X2	122.190	-0.176	0.102	0.092	-0.018
GB47844	AP-3 complex subunit delta-1	121.983	-0.477	-0.061	-0.014	0.087
GB42778	U6 snRNA-associated Sm-like protein LSm4-like	121.959	0.240	0.126	0.038	-0.025

(continued)

Gene	Name	k	am_fc	bt_fc	lf_fc	ln_fc
GB43622	endoplasmic reticulum mannosyl-oligosaccharide 1,2-alpha-mannosidase-like isoform X1	121.931	-0.229	-0.018	-0.071	0.118
GB41969	mediator of RNA polymerase II transcription subunit 26 isoform X3	121.892	-0.209	0.141	0.215	-0.049
725795	INO80 complex subunit D-like	121.872	0.076	-0.068	0.036	0.117
GB55012	transcription factor Dp-1	121.848	0.062	0.240	0.110	0.901
GB47554	apoptosis inhibitor 5-like	121.695	-0.187	0.064	0.198	0.085
GB54968	phosphorylated adapter RNA export protein	121.579	-0.195	0.068	0.104	0.136
GB46962	SH3 domain-binding glutamic acid-rich protein homolog isoform X1	121.528	0.293	0.067	0.177	0.121
GB46773	cysteine-rich protein 2-binding protein-like	121.394	-0.404	0.112	0.012	0.125
GB51589	nuclear pore membrane glycoprotein 210-like	121.357	0.410	0.047	-0.445	-0.047
GB53717	uncharacterized protein LOC726365 isoform X2	121.206	-0.444	0.059	0.144	-0.078
GB50032	prefoldin subunit 6-like isoform X1	121.070	0.247	-0.216	0.059	0.122
GB41754	protein DDI1 homolog 2-like isoform X2	121.016	0.093	-0.075	0.019	0.156
102655440	uncharacterized protein LOC102655440	120.995	-0.649	0.044	0.110	0.118
GB54019	myosin-1-like isoform X2	120.967	-0.024	0.149	0.117	0.025
GB49883	uncharacterized protein CG7065-like	120.635	0.000	-0.041	0.035	0.040
GB44049	spermatogenesis-associated protein 5-like isoform X1	120.599	0.087	-0.098	0.104	0.107
GB47427	protein ariadne-2-like	120.572	-0.066	0.065	-0.051	0.037
551815	uncharacterized protein C9orf114 homolog	120.334	0.100	0.134	0.094	-0.237
GB41455	cysteine–tRNA ligase, cytoplasmic-like	120.333	0.039	-0.031	0.052	0.235
GB52801	phosphatidylinositol 5-phosphate 4-kinase type-2 beta-like isoformX1	120.313	-0.141	-0.121	0.255	0.059
GB50555	thioredoxin peroxidase 3 isoform 2	120.293	0.161	0.049	0.632	0.080
GB45107	peroxisome biogenesis factor 1-like isoform X1	120.239	0.156	0.295	0.064	-0.071
GB42751	LOW QUALITY PROTEIN: DNA repair protein RAD50	120.170	-0.043	-0.160	0.179	0.161
GB48467	methyltransferase-like protein 13-like isoform X2	120.134	-0.112	0.057	0.275	-0.008
GB42744	uncharacterized protein LOC100577988	120.095	-0.265	0.004	0.468	0.060
GB48247	uncharacterized protein LOC410423 isoform X1	119.819	-0.337	0.205	0.001	-0.037
GB41140	UPF0609 protein CG1218-like isoform X1	119.800	-0.752	0.112	0.341	0.139
GB45709	broad-complex core protein isoforms 1/2/3/4/5 isoform X6	119.690	-0.225	-0.004	0.476	0.052
GB53250	PHD finger protein 12-like isoform X1	119.640	-0.343	0.071	0.008	0.084
GB54385	cation transport regulator-like protein 2-like	119.557	-0.276	-0.054	0.054	-0.287
GB53832	lipoyltransferase 1, mitochondrial-like isoformX2	119.492	-0.231	-0.212	-0.081	-0.007
GB45695	protein cramped-like isoform X2	119.449	-0.285	-0.197	0.064	0.107
GB41002	protein timeless homolog isoform X3	119.409	-0.116	0.249	0.065	-0.133
GB45837	transcription factor 25-like isoform X2	119.399	-0.409	0.040	0.022	0.105
GB53398	zinc finger protein 622-like isoform 1	119.291	-0.022	-0.006	-0.059	0.077
GB42014	E3 ubiquitin-protein ligase TRIP12-like isoform X3	119.173	-0.055	-0.014	0.101	0.004
GB45014	nuclear pore complex protein Nup93-like	119.093	0.181	0.219	0.079	-0.147
GB54297	programmed cell death protein 2	119.064	-0.889	0.018	0.306	0.111
GB41489	nuclear fragile X mental retardation-interacting protein 1-like isoform X1	119.047	0.016	0.116	0.049	0.019
724372	nucleolar protein 12-like isoform X1	119.045	-0.913	0.096	-0.260	-0.069
GB54209	prostatic acid phosphatase-like isoform X1	119.014	-0.695	0.146	0.135	-0.405
GB42200	uncharacterized protein LOC408577	118.930	-0.052	-0.077	-0.159	-0.367
GB43213	muskelin	118.839	-0.163	0.186	-0.090	0.085
GB50704	spindle assembly abnormal protein 6 homolog	118.836	0.050	-0.019	0.007	0.031
GB53538	nischarin	118.787	0.127	-0.198	0.194	0.050
GB45507	cell division cycle protein 23 homolog	118.749	-0.239	-0.055	-0.012	0.022
GB48789	disks large homolog 5-like isoform X2	118.663	-1.320	-0.013	-0.083	0.017
GB46194	uncharacterized protein LOC725889	118.517	-0.106	0.158	0.689	0.084
GB47827	exosome complex component RRP45-like isoform X2	118.503	-0.400	0.163	0.032	0.190
GB54530	dedicator of cytokinesis protein 7-like	118.398	-0.141	-0.257	0.596	0.108
GB41603	PTB domain-containing adapter protein ced-6 isoform X2	118.280	-0.228	0.015	-0.474	-0.114
102656134	transcriptional adapter 2B-like isoform X2	118.219	0.092	-0.014	0.509	0.058
GB54433	60S ribosomal protein L19	118.126	0.338	0.044	-0.042	-0.005
411879	WD repeat-containing protein 82-like	118.107	0.275	0.000	0.139	0.112
GB47016	LOW QUALITY PROTEIN: kynurenine–oxoglutarate transaminase 3-like	118.106	0.138	-0.132	-0.006	0.020
GB42714	splicing factor 45-like	118.023	0.163	0.094	-0.056	-0.001

(continued)

Gene	Name	k	am_fc	bt_fc	lf_fc	ln_fc
GB49155	lysine–tRNA ligase isoform X1	118.018	0.195	0.001	-0.044	0.002
GB47965	MKI67 FHA domain-interacting nucleolar phosphoprotein-like	117.973	-0.203	0.007	0.043	0.037
GB53199	N-acetylgalactosaminyltransferase 7	117.879	0.038	0.128	-0.067	-0.004
GB40886	CCA tRNA nucleotidyltransferase 1, mitochondrial-like isoform X2	117.789	-0.709	-0.026	-0.097	-0.031
GB45666	protein arginine N-methyltransferase 3	117.739	-0.449	0.130	-0.250	-0.042
GB47299	bile salt-activated lipase-like isoform X4	117.717	0.171	-0.226	-0.165	-0.165
GB49828	zinc finger CCHC-type and RNA-binding motif-containing protein 1-like	117.602	-0.192	0.094	0.081	-0.141
GB48857	quinone oxidoreductase-like protein 2-like	117.595	0.374	0.097	0.141	0.089
GB47589	cell division cycle and apoptosis regulator protein 1	117.484	-0.603	0.057	-0.006	0.119
GB53223	rhomboid-related protein 3-like isoform X1	117.479	0.097	-0.159	0.042	0.039
GB50130	nucleolar complex protein 3 homolog	117.470	-0.161	0.228	0.098	0.105
GB49457	hydroxymethylglutaryl-CoA lyase, mitochondrial-like isoform X1	117.467	-0.296	0.058	-0.011	-0.123
GB54049	eukaryotic translation initiation factor 3 subunit L-like	117.463	0.100	0.057	-0.016	-0.106
GB55804	kelch domain-containing protein 10 homolog isoform 2	117.320	0.227	-0.094	0.194	0.102
GB44140	WW domain-binding protein 4-like	117.312	0.027	-0.031	0.101	-0.058
GB46889	SWI/SNF-related matrix-associated actin-dependent regulator of chromatin subfamily A-like protein 1-like	117.030	-0.718	-0.005	0.133	0.255
GB48682	protein FAM50 homolog	117.004	0.059	0.292	0.112	0.300
GB48103	syntaxin-12 isoform X1	116.981	-0.181	-0.043	-0.016	0.118
102656501	putative uncharacterized zinc finger protein 814-like	116.970	0.082	-0.078	0.106	-0.033
GB41231	ubiquitin-conjugating enzyme E2 G1	116.939	0.115	0.119	-0.061	0.037
GB45449	protein LZIC-like	116.912	-0.016	-0.011	-0.170	0.171
GB51505	sphingomyelin phosphodiesterase 4-like	116.892	0.131	0.034	0.079	0.118
GB55517	uncharacterized protein LOC410000 isoform X1	116.847	-0.357	0.044	-0.610	0.125
GB55477	39S ribosomal protein L19, mitochondrial	116.844	-0.314	0.176	-0.887	0.096
GB44758	eukaryotic translation initiation factor 2-alpha kinase isoform X3	116.583	-0.224	-0.086	-0.133	0.247
GB46898	transcription initiation factor TFIID subunit 7 isoform X3	116.529	-0.017	0.123	0.036	0.180
GB44082	engulfment and cell motility protein 1	116.524	0.317	-0.065	0.180	-0.065
102654955	ATP synthase subunit b, mitochondrial-like	116.516	-0.478	-0.090	0.306	0.410
GB52790	gamma-aminobutyric acid receptor-associated protein	116.475	0.126	-0.036	-0.164	-0.007
GB53421	Golgi resident protein GCP60-like	116.467	-0.006	0.009	0.145	0.007
GB47409	transmembrane protein 145-like isoform X1	116.363	-0.435	-0.231	0.467	0.069
GB41822	acid phosphatase-like protein 2-like	116.279	-0.413	0.254	-0.038	0.027
GB54702	S1 RNA-binding domain-containing protein 1-like isoform X1	116.246	-0.391	0.106	0.147	0.073
GB47306	sulfhydryl oxidase 1-like	116.127	-0.288	-0.031	0.305	0.008
102654029	uncharacterized protein LOC102654029 isoform X1	116.023	-0.437	0.057	0.112	0.961
726043	leucine-rich repeat-containing protein 57-like	115.986	-0.221	0.080	0.056	-0.014
GB50030	SET and MYND domain-containing protein 5	115.903	0.086	-0.123	-0.025	-0.027
GB40765	ankyrin repeat domain-containing protein 12-like isoform X3	115.863	-0.691	-0.106	0.026	-0.047
GB46752	microtubule-associated serine/threonine-protein kinase-like, transcript variant X3	115.613	0.366	0.229	0.002	0.112
GB49621	alpha-L-fucosidase	115.488	-0.194	-0.056	-0.032	0.120
GB41677	adenosine kinase 1-like isoformX1	115.447	-0.059	0.007	-0.019	-0.023
GB41510	probable deoxyhypusine synthase-like isoform X1	115.401	-0.070	-0.023	0.091	0.021
GB44969	lipoamide acyltransferase component of branched-chain alpha-keto acid dehydrogenase complex, mitochondrial-like	115.371	-0.025	-0.063	-0.071	-0.221
GB52803	28 kDa heat- and acid-stable phosphoprotein-like	115.371	0.327	0.020	0.107	0.093
GB48059	ecdysone receptor isoform B1	115.212	-0.421	0.155	-0.053	0.111
GB40654	nuclear factor NF-kappa-B p110 subunit isoform X3	115.182	-0.438	-0.063	0.383	0.048
GB40032	rRNA-processing protein UTP23 homolog	115.167	-1.084	0.201	0.048	0.266
GB47502	uncharacterized protein LOC409690 isoform X2	115.131	-0.299	-0.056	0.151	-0.036
GB44598	threonine aspartase 1-like isoform X2	115.088	-0.134	0.100	-0.035	0.260
GB40431	beta-ureidopropionase-like isoform 1	114.929	0.434	0.047	-0.080	-0.095
GB42693	la protein homolog	114.846	0.074	0.148	-0.107	0.060
GB41545	MD-2-related lipid-recognition protein-like	114.830	0.191	-0.015	0.120	-0.073
GB48876	probable methyltransferase-like protein 15 homolog isoform X1	114.681	-0.678	-0.069	0.209	0.135

(continued)

Gene	Name	k	am_fc	bt_fc	lf_fc	ln_fc
GB49908	cGMP-dependent protein kinase foraging	114.554	0.196	0.060	0.098	-0.009
GB51526	uncharacterized protein LOC410093 isoform X1	114.448	-0.116	0.236	-0.398	0.013
GB55156	THUMP domain-containing protein 1 homolog	114.359	-0.587	0.064	0.053	0.120
GB51963	mitochondrial ribonuclease P protein 1 homolog	114.335	-0.491	-0.200	-0.014	0.139
GB40859	uncharacterized protein LOC100576321	114.233	-0.669	0.094	-0.675	0.117
GB45133	zinc finger protein 341-like isoform X2	114.079	0.096	0.337	0.124	-0.114
GB53683	integrator complex subunit 10-like isoform X3	114.074	0.191	-0.144	0.171	0.059
102655284	serine–tRNA ligase, mitochondrial-like	114.039	0.117	0.025	-0.057	-0.091
GB41117	LOW QUALITY PROTEIN: serine/threonine-protein kinase 11-interacting protein-like	114.011	-0.340	-0.061	0.169	-0.173
GB52746	zinc finger protein 845-like isoform X1	113.979	0.211	-0.076	0.153	0.183
GB46790	solute carrier family 35 member F5-like isoform X2	113.969	-0.097	0.013	0.115	0.159
GB47820	zinc finger protein 91-like isoform X2	113.945	-0.473	0.050	0.059	0.054
GB54568	uncharacterized protein LOC726916	113.944	-0.193	0.252	1.787	0.042
GB48846	organic cation transporter protein-like	113.876	-0.821	0.149	0.012	-0.203
GB45150	adenylate cyclase 3 isoform X2	113.840	-0.119	0.182	-0.038	-0.227
GB54314	nucleostemin 1	113.830	-0.548	0.139	-0.224	0.066
GB53824	neuferricin-like	113.767	-0.459	-0.044	1.421	-0.019
GB41449	cytoplasmic FMR1-interacting protein isoform 1	113.677	-0.099	-0.002	0.052	-0.017
GB49497	V-type proton ATPase subunit B-like	113.561	0.114	-0.071	0.170	-0.018
GB48018	uncharacterized protein LOC100578752	113.513	0.162	0.075	0.083	0.131
GB47418	uncharacterized LOC409282, transcript variant X3	113.428	-0.184	0.000	0.180	0.087
GB45318	uncharacterized protein LOC725813	113.413	0.027	-0.125	-0.010	0.099
GB42083	uncharacterized protein LOC100578864	113.324	-1.018	-0.075	0.714	-0.077
GB45041	metallophosphoesterase 1 homolog	113.220	-0.203	0.040	2.105	0.122
GB41378	probable helicase with zinc finger domain-like	113.179	-0.308	0.291	0.024	-0.056
GB41259	INO80 complex subunit B-like isoform X1	113.156	-0.310	0.070	0.053	0.101
GB49235	WD repeat-containing protein 24-like	113.039	-0.049	0.090	0.102	0.035
GB46590	interaptin-like	113.000	-0.268	0.090	0.047	0.123
GB52697	origin recognition complex subunit 4 isoform X2	112.949	-0.800	-0.174	0.028	-0.054
GB46267	uncharacterized protein LOC727260	112.866	0.374	0.131	-0.053	0.199
GB54729	ATP-dependent DNA helicase PIF1-like	112.837	-0.808	0.237	-0.060	-0.401
GB54256	uncharacterized protein LOC100577343 isoform X2	112.820	-0.232	-0.297	0.246	0.015
GB40075	importin subunit beta-1 isoform X2	112.814	0.010	0.247	-0.250	-0.102
GB41671	uncharacterized protein LOC724294	112.797	-0.465	-0.030	-0.028	0.020
GB55187	conserved oligomeric Golgi complex subunit 6-like	112.733	0.005	0.054	0.099	0.058
GB49427	protein RRNAD1-like	112.700	-0.256	0.033	-2.053	0.167
GB40296	kanadaptin-like	112.644	-0.547	0.037	0.050	0.094
GB42785	spermatogenesis-defective protein 39 homolog	112.604	-0.129	0.019	-0.150	0.189
GB41291	heparan-alpha-glucosaminide N-acetyltransferase-like isoform X2	112.563	0.015	-0.133	0.152	0.094
GB53296	pipsqueak	112.535	-0.123	0.178	0.095	0.036
GB55601	protein YIPF1-like	112.490	0.103	-0.058	0.137	0.110
GB46714	nucleoporin SEH1 isoform X1	112.479	0.461	0.022	0.128	0.069
GB45179	mRNA-capping enzyme	112.424	0.208	-0.009	0.005	-0.088
GB42697	SPRY domain-containing protein 7-like isoform 1	112.333	0.362	-0.156	0.127	0.242
GB55112	histone H4 transcription factor-like isoform X1	111.951	-0.540	0.030	-0.313	-0.133
GB52804	lysophospholipid acyltransferase 7-like	111.894	-0.337	0.093	0.690	-0.069
GB50024	origin recognition complex subunit 1	111.891	-0.183	0.354	0.140	0.188
100578218	mimitin, mitochondrial-like	111.869	-0.271	0.042	0.085	0.090
GB45372	kynurenine formamidase-like isoform X4	111.850	-0.170	-0.011	0.012	-0.029
GB42814	calcium-transporting ATPase type 2C member 1-like isoform X2	111.664	0.150	0.040	-0.287	-0.070
727332	protein BUD31 homolog isoform X1	111.661	0.406	-0.061	0.121	0.011
GB52455	protein CASC4-like isoform X2	111.641	0.347	-0.065	-0.042	-0.104
GB45740	39S ribosomal protein L40, mitochondrial	111.623	-0.145	0.020	-0.175	0.071
GB54326	apoptosis regulatory protein Siva-like	111.417	-0.417	0.099	0.040	-0.063
GB52701	chloride channel protein 2-like isoform X3	111.309	-0.221	-0.146	0.293	-1.415
GB42664	probable ATP-dependent RNA helicase DDX43-like	111.288	-0.201	0.126	-0.128	0.104
GB55010	spermine synthase-like isoformX1	111.222	0.110	0.002	-0.751	0.034
410849	protein sly1 homolog	111.161	-0.119	0.038	-0.104	-0.241
GB42820	N-alpha-acetyltransferase 30-like	111.125	-0.408	0.077	0.068	0.160

(continued)

Gene	Name	k	am_fc	bt_fc	lf_fc	ln_fc
GB45185	FAS-associated factor 2	110.998	0.120	-0.073	0.314	-0.118
GB50146	xaa-Pro aminopeptidase 1-like isoform X1	110.993	-0.049	0.165	0.023	-0.052
GB53794	melanotransferrin	110.858	0.300	-0.025	0.058	-0.102
GB45749	sorting nexin-4-like isoform X1	110.822	0.278	-0.178	0.094	0.081
GB43376	low molecular weight phosphotyrosine protein phosphatase-like isoform X2	110.783	-0.133	0.028	-0.020	0.266
GB52982	magnesium transporter NIPA2	110.718	-0.115	-0.048	0.155	0.117
GB47735	endonuclease III-like protein 1-like	110.664	-0.185	0.152	2.441	0.230
GB53846	structural maintenance of chromosomes protein 5-like	110.625	-0.105	-0.155	-0.017	0.009
GB47879	phosphatidylinositol 4-kinase alpha-like	110.583	0.140	-0.053	0.563	0.048
GB40030	adenylyltransferase and sulfurtransferase MOCS3-like	110.324	-0.311	-0.044	0.070	0.170
GB44745	zinc finger protein 267-like	110.270	-0.594	0.131	-0.047	-0.127
GB49894	probable cytochrome P450 6a14 isoform X1	110.209	-0.423	0.224	0.368	0.175
GB49171	protein sidekick isoformX2	110.189	-0.113	0.159	-0.741	0.344
GB42707	mortality factor 4-like protein 1-like isoform X1	110.154	-0.114	0.015	-0.019	0.149
GB53681	cytochrome b5 reductase 4-like isoform X4	110.081	-0.211	-0.113	0.030	-0.019
GB40673	lambda crystallin-like protein	110.046	0.035	-0.027	-0.218	0.031
GB53376	uncharacterized protein C18orf8-like	109.921	0.185	0.082	0.051	0.244
GB41161	probable DNA-directed RNA polymerases I and III subunit RPAC2-like	109.880	-0.053	-0.019	0.006	-0.001
GB41335	N(6)-adenine-specific DNA methyltransferase 2-like	109.879	0.025	-0.017	0.108	0.123
410675	protein BTG3-like isoform X2	109.722	0.214	-0.167	0.088	0.004
GB46477	LOW QUALITY PROTEIN: putative inositol monophosphatase 3-like	109.700	0.084	-0.112	0.082	0.141
GB48017	transforming growth factor beta regulator 1-like	109.685	-0.002	0.028	-0.078	0.229
GB51160	ralBP1-associated Eps domain-containing protein 2-like isoform X1	109.639	0.007	-0.103	-0.020	0.093
102656137	coiled-coil domain-containing protein 174-like	109.589	-0.594	0.014	0.118	0.156
GB41654	deformed epidermal autoregulatory factor 1	109.497	0.225	0.138	0.052	-0.079
GB54659	transcription initiation factor TFIID subunit 8-like	109.440	-0.069	0.111	-0.051	0.192
GB50993	DNA-directed RNA polymerase III subunit RPC4-like	109.440	0.122	0.118	0.062	0.211
GB55164	lethal(2) giant larvae protein homolog 1-like isoform X9	109.417	0.117	-0.014	0.059	0.501
GB55463	caprin homolog	109.280	0.131	0.155	0.004	-0.400
GB52975	uncharacterized protein LOC409658	109.212	-0.027	0.107	0.002	-0.127
GB47162	ADP-ribosylation factor-like protein 13B-like isoform X2	109.136	-0.281	-0.009	0.087	-0.049
GB51675	uncharacterized protein R102.4-like isoform X2	108.945	-0.282	0.054	-0.042	0.071
GB41982	polycomb protein Scm isoform X2	108.788	0.217	-0.001	0.066	-0.173
GB48031	vacuolar protein sorting-associated protein 54	108.698	-0.202	-0.165	0.048	-0.040
GB40400	tyrosine-protein kinase PR2-like	108.546	-0.217	-0.428	-0.462	0.558
GB47295	KH domain-containing protein C56G2.1-like isoform X3	108.464	-0.615	0.143	-0.075	-0.095
GB44558	probable malonyl-CoA-acyl carrier protein transacylase, mitochondrial-like	108.445	-0.423	-0.082	-0.037	-0.062
GB47310	NECAP-like protein CG9132-like isoform X1	108.364	-0.326	-0.019	-0.042	-0.214
GB40489	NADH dehydrogenase [ubiquinone] 1 alpha subcomplex subunit 10, mitochondrial	108.340	-0.574	-0.070	0.122	0.171
GB53256	39S ribosomal protein L3, mitochondrial	108.332	-0.017	-0.035	-0.008	0.212
GB51743	protein decapentaplegic	108.317	-0.139	0.010	0.294	-0.053
102656258	nuclear RNA export factor 2-like	108.138	0.224	-0.059	-0.082	0.034
GB42779	alpha-1,3/1,6-mannosyltransferase ALG2-like	108.136	-0.362	0.065	-0.183	0.094
GB45515	uncharacterized protein LOC411721	108.099	-0.493	0.040	0.159	0.140
GB43270	MATH and LRR domain-containing protein PFE0570w-like isoform X2	108.057	-0.864	-0.022	0.190	-0.437
GB44877	probable 39S ribosomal protein L49, mitochondrial-like	108.022	0.115	0.088	0.071	0.095
GB53606	histone-lysine N-methyltransferase eggless	107.890	0.285	-0.017	0.215	-0.066
GB47885	probable cytochrome P450 304a1	107.804	0.040	-0.105	-0.224	0.470
102656433	DNA ligase 1-like	107.748	-0.370	0.129	0.072	0.033
GB53021	PR domain zinc finger protein 10-like isoform X2	107.729	-0.228	0.026	0.223	-0.086
GB49067	zinc finger protein GLI1-like	107.635	-0.113	-0.085	0.087	-0.039
GB48158	uncharacterized protein LOC408905	107.593	0.046	0.214	-0.517	0.151
GB43353	uncharacterized protein LOC727578	107.586	-0.226	-0.060	0.314	0.075
GB43586	uncharacterized protein LOC100576934	107.502	-0.026	-0.122	0.173	-0.003
GB49139	autophagy protein 5	107.497	0.063	0.000	1.216	0.095

(continued)

Gene	Name	k	am_fc	bt_fc	lf_fc	ln_fc
GB54253	uncharacterized protein LOC727266	107.460	0.097	-0.049	0.137	-0.052
GB55071	F-box only protein 9-like	107.385	-0.197	-0.136	0.184	0.096
GB42139	E3 ubiquitin-protein ligase RFWD3	107.112	-0.187	0.055	-0.081	0.161
GB52058	chromobox protein homolog 1-like isoform X1	107.045	0.079	0.128	0.209	0.124
GB52888	uncharacterized protein C9orf85 homolog	106.980	-0.444	-0.064	0.078	0.152
102655203	EKC/KEOPS complex subunit TPRKB-like	106.920	-0.457	-0.273	1.280	0.166
GB40949	homologous-pairing protein 2 homolog	106.918	-0.164	-0.049	-0.032	0.203
GB47461	protein inturned-like	106.872	-0.417	0.053	-0.038	0.209
GB50796	probable N-acetyltransferase CML3-like	106.870	-0.010	-0.094	0.101	-0.090
GB49050	zinc finger protein 484-like isoform X2	106.821	-0.208	0.096	-0.146	0.001
GB41176	polyglutamine-binding protein 1-like	106.738	-0.347	0.135	1.182	0.011
GB41889	aminoacylase-1-like	106.691	0.163	0.044	-0.052	-0.229
GB52266	furin-like protease 2-like	106.591	-0.927	-0.074	0.011	0.044
GB52642	probable serine/threonine-protein kinase tsuA-like	106.370	-0.045	-0.160	-0.066	-0.003
GB54808	protein maelstrom homolog isoform X3	106.350	-0.117	0.176	0.051	-0.013
GB48255	isochorismatase domain-containing protein 2, mitochondrial-like isoform X3	106.248	0.092	0.004	-0.147	0.002
GB46792	inosine-5'-monophosphate dehydrogenase isoform 1	106.198	0.491	-0.011	0.049	-0.191
GB55076	signal recognition particle 19 kDa protein	106.102	0.203	-0.075	0.035	-0.076
GB48414	trafficking protein particle complex subunit 3	106.014	-0.017	0.137	0.078	0.059
GB54387	probable ribosome biogenesis protein RLP24-like	106.012	0.237	0.104	-0.070	0.131
GB50996	DNA-directed RNA polymerase II subunit RPB3-like	105.925	0.041	0.219	0.151	0.009
GB46476	ubiquitin-conjugating enzyme E2 W-like	105.843	0.143	0.031	0.033	0.049
GB50298	putative transferase CAF17 homolog, mitochondrial-like	105.803	-0.132	0.014	-0.040	-0.054
GB47389	LETM1 and EF-hand domain-containing protein anon-60Da, mitochondrial-like	105.799	-0.024	0.079	-0.150	0.191
GB50103	crossover junction endonuclease EME1-like	105.726	-0.298	-0.053	0.268	-0.016
GB44705	mediator of RNA polymerase II transcription subunit 6	105.621	0.005	0.037	0.205	0.011
GB40843	leucine-rich repeat-containing protein 47-like	105.459	-0.041	-0.092	0.052	0.055
GB42384	serine/threonine-protein kinase RIO2-like	105.243	-0.235	0.148	0.327	0.031
GB41171	uncharacterized protein LOC551860	105.190	-0.105	0.313	-0.148	0.370
GB51627	dehydrodolichyl diphosphate synthase-like isoform X4	105.121	-0.993	0.040	-0.078	0.214
GB43495	tRNA dimethylallyltransferase, mitochondrial-like isoform X2	105.117	-0.241	0.121	0.074	-0.006
GB40355	coiled-coil domain-containing protein 94-like isoform X2	105.112	-0.571	0.266	-0.009	-0.018
413881	AP-3 complex subunit sigma-2-like, transcript variant X2	105.074	0.156	0.027	0.182	0.022
GB46697	carbonic anhydrase 2	105.025	-0.220	-0.030	-0.106	-0.008
GB47961	mitogen-activated protein kinase kinase kinase 4	105.014	0.123	-0.048	0.111	-0.311
GB47454	putative inhibitor of apoptosis	104.968	-0.433	-0.027	-0.386	0.093
GB45389	E3 ubiquitin-protein ligase RNF123-like isoform X1	104.882	-0.475	-0.106	0.026	-0.215
GB49451	mediator of RNA polymerase II transcription subunit 28-like	104.793	0.221	-0.015	0.086	0.064
GB55518	uncharacterized protein C17orf59 homolog isoform X3	104.791	0.000	0.005	0.212	0.197
GB40515	myotubularin-related protein 4-like isoform X1	104.540	0.129	0.056	0.103	-0.013
551412	polycomb protein EED-like	104.503	0.103	0.272	-0.003	0.004
GB40862	UPF0545 protein C22orf39 homolog	104.447	-0.267	-0.106	0.096	0.228
GB44358	leucine-rich repeat-containing protein 48-like isoform X1	104.425	-1.155	-0.597	0.021	0.343
GB45083	protein FRG1 homolog	104.391	-0.222	0.067	0.126	0.141
GB47834	histone deacetylase complex subunit SAP30 homolog isoform X1	104.355	-0.185	-0.016	-0.977	-0.176
GB45193	glutaryl-CoA dehydrogenase, mitochondrial	104.324	0.119	-0.010	0.061	-0.182
GB49553	stromal membrane-associated protein 1-like	104.276	0.084	0.039	0.284	0.082
GB45020	uncharacterized protein LOC408364 isoform X1	104.160	-0.018	-0.003	0.174	0.114
GB43627	zinc finger protein GLI2-like isoform X4	103.992	-0.525	0.050	0.183	0.056
GB45025	mTERF domain-containing protein 1, mitochondrial-like	103.958	-0.723	-0.126	0.407	0.273
GB42089	transmembrane protein 256 homolog	103.935	0.079	-0.039	-0.112	0.028
GB41569	UPF0364 protein C6orf211 homolog isoform X1	103.890	-0.351	-0.232	0.002	0.155
GB47443	mesoderm induction early response protein 1-like isoform X6	103.824	-0.156	0.097	0.270	-0.040
GB50809	sprT-like domain-containing protein Spartan-like isoform X3	103.722	-0.095	0.035	-0.001	-0.033
GB42805	heparan-sulfate 6-O-sulfotransferase 2	103.644	0.047	-0.091	0.020	0.047
GB50167	probable 28S ribosomal protein S26, mitochondrial	103.634	-0.838	0.528	0.085	0.175
725620	28S ribosomal protein S33, mitochondrial	103.501	-0.200	-0.032	0.075	0.138
GB53672	failed axon connections isoform X2	103.411	0.227	0.157	0.024	-2.471

(continued)

Gene	Name	k	am_fc	bt_fc	lf_fc	ln_fc
GB50694	F-box/LRR-repeat protein 20-like isoform X2	103.399	-0.113	-0.092	0.151	0.005
GB43126	transmembrane protein 184B-like isoform X1	103.381	-0.008	0.011	0.170	-0.058
GB48126	putative uncharacterized protein DDB_G0282133-like	103.293	-0.101	0.147	0.027	-0.261
GB51465	NAD-dependent protein deacetylase Sirt7	103.146	-0.218	0.300	-0.078	0.051
GB53430	reticulon-4-interacting protein 1, mitochondrial-like isoform 1	103.121	0.062	0.009	0.052	0.232
GB49837	WD repeat domain phosphoinositide-interacting protein 3-like isoform 1	103.087	0.396	0.076	0.098	0.026
GB41157	RPII140-upstream gene protein-like	102.925	-0.481	-0.023	1.273	0.149
102654398	DNA repair protein xrcc4-like	102.917	-1.082	0.023	0.327	0.240
GB55902	cystathionine-beta-synthase	102.905	-0.018	0.064	-0.098	-0.063
GB41030	UV excision repair protein RAD23 homolog B-like isoformX2	102.873	0.172	-0.065	-0.112	0.052
GB52091	protein suppressor of forked-like isoform X3	102.864	0.162	-0.020	0.165	-0.061
102655971	5'-nucleotidase domain-containing protein 1-like	102.770	0.145	-0.068	0.095	0.165
GB44695	lysophospholipase-like protein 1-like isoform X1	102.717	-0.072	-0.061	-0.022	0.128
GB43434	enolase-phosphatase E1-like isoform 2	102.710	-0.049	0.137	0.066	0.280
724133	peptide chain release factor 1-like, mitochondrial-like	102.705	-0.238	0.172	0.130	0.042
102656263	protein farnesyltransferase subunit beta-like	102.683	0.002	-0.046	0.033	-0.123
GB49242	apoptosis-inducing factor 3 isoform X1	102.666	0.178	-0.174	0.127	0.043
GB53201	39S ribosomal protein L44, mitochondrial	102.662	-0.432	0.001	-0.075	0.241
GB41441	ubiquitin-like protein 3-like isoform X3	102.654	-0.316	0.091	-0.176	0.220
GB51756	zinc finger protein 69-like isoform X1	102.569	0.343	-0.107	0.044	-0.140
GB40146	GTP-binding protein ypt7	102.549	0.370	-0.119	0.120	0.078
GB55283	uncharacterized protein LOC552036 isoform X2	102.508	0.174	-0.012	0.181	0.118
GB44540	DNA repair protein RAD51 homolog 1	102.497	0.105	0.210	-0.253	0.027
GB51281	uncharacterized protein LOC409514 isoform 1	102.487	-0.188	-0.236	0.154	-0.908
GB51080	uncharacterized protein C16orf52 homolog A isoform X4	102.465	0.410	0.072	0.123	0.083
408979	eukaryotic translation initiation factor 5 isoform X2	102.329	0.115	0.147	-0.014	0.165
GB52473	DNA polymerase eta	102.113	0.015	0.188	0.074	0.192
GB53916	eukaryotic translation initiation factor 3 subunit E-like isoform X2	102.080	0.070	0.088	-0.037	-0.574
GB46495	tetratricopeptide repeat protein 4	101.988	-0.265	0.046	-0.028	0.141
GB54981	cysteine-rich hydrophobic domain 2 protein-like	101.935	-0.075	0.023	0.024	-0.005
GB40648	disks large 1 tumor suppressor protein-like, transcript variant X2	101.917	-0.324	-0.190	0.309	0.104
GB55452	apolipophorin-III-like protein precursor	101.760	-0.192	-0.005	-0.130	-0.164
GB42941	uridine-cytidine kinase-like 1-like isoform 1	101.730	0.098	-0.035	0.166	0.064
102654889	zinc finger protein 235-like	101.705	-0.725	0.164	0.373	-0.187
GB45281	E3 ubiquitin-protein ligase hyd isoform X2	101.695	0.032	0.088	0.071	-0.199
GB46986	39S ribosomal protein L46, mitochondrial	101.685	-0.254	0.035	-0.051	0.319
GB45665	fidgetin-like protein 1-like	101.628	-0.068	0.289	-0.025	-0.043
GB46067	putative N-acetylglucosamine-6-phosphate deacetylase	101.487	0.396	-0.009	0.110	0.269
GB47719	protein KRI1 homolog	101.435	-0.308	0.075	-0.014	0.025
GB43552	leucine-rich repeat-containing protein 15-like isoform X1	101.279	-0.238	-0.141	-0.026	0.086
GB45618	E3 ubiquitin-protein ligase mind-bomb isoform X3	101.181	-0.413	0.066	0.076	-0.096
GB53790	sorting nexin-27-like isoform X3	101.169	-0.524	0.057	0.027	-0.018
GB44734	checkpoint protein HUS1-like isoform X1	101.105	-0.947	0.371	-0.035	0.428
GB49349	CTL-like protein 1-like	101.087	-0.026	-0.235	0.052	0.034
GB41153	peroxisomal membrane protein PEX16-like	100.928	-0.317	0.208	-0.007	0.013
GB50197	splicing factor 3A subunit 3 isoform X1	100.811	0.051	0.117	0.206	0.178
GB49172	nucleosome-remodeling factor subunit NURF301-like isoform X2	100.805	-0.413	-0.085	0.015	-0.194
GB46681	cyclin-K	100.731	0.170	-0.002	0.014	-0.062
GB51000	alpha-(1,6)-fucosyltransferase	100.680	-0.019	0.075	-0.005	0.163
100578779	uncharacterized protein LOC100578779	100.662	-0.856	-0.125	0.219	0.037
GB44113	5'-AMP-activated protein kinase subunit beta-1 isoform X2	100.538	0.172	-0.022	0.005	0.065
GB44611	digestive organ expansion factor homolog	100.485	0.114	0.033	0.024	-0.050
GB51117	rho GTPase-activating protein 18-like isoform X2	100.437	-0.480	0.056	0.058	-0.017
GB46132	glycosaminoglycan xylosylkinase-like	100.402	0.670	0.007	0.224	0.043
GB53415	WW domain-binding protein 2-like isoform X1	100.370	0.432	-0.018	-0.457	-0.326
GB51060	probable serine/threonine-protein kinase DDB_G0282963 isoform X4	100.349	-1.729	0.045	-0.272	0.568

(continued)

Gene	Name	k	am_fc	bt_fc	lf_fc	ln_fc
GB46877	kinase suppressor of Ras 2	100.346	0.150	0.132	0.102	0.118
GB41215	rotatin-like isoform X1	100.309	-0.112	-0.185	0.760	0.155
GB17746	putative lipoyltransferase 2, mitochondrial	100.238	-0.437	-0.010	-0.632	0.118
102656846	G2/mitotic-specific cyclin-A-like	100.179	0.103	0.326	-0.134	0.175
GB44776	lanC-like protein 2-like isoform X5	100.107	0.057	0.012	0.192	-0.027
GB53029	pre-mRNA-processing factor 17 isoform X1	100.091	-0.027	0.217	-0.119	-0.127
GB49012	glycerol kinase isoform X1	99.985	0.273	0.240	0.073	0.031
GB41794	sorting nexin-30-like	99.887	0.007	-0.090	0.047	0.176
102655841	uncharacterized protein LOC102655841	99.844	0.192	-0.043	0.055	0.111
GB48963	chromosome transmission fidelity protein 18 homolog isoform X2	99.750	-1.359	0.064	1.160	0.201
GB55864	UDP-glucuronosyltransferase 1-8-like	99.644	-0.500	0.081	-0.050	0.047
GB53710	breakpoint cluster region protein-like isoform X3	99.607	-0.007	-0.131	0.005	-0.115
GB42383	uncharacterized protein MAL13P1.304-like	99.586	-1.050	0.060	-0.139	0.213
GB45228	chondroitin sulfate synthase 2-like	99.570	-0.571	0.056	0.099	0.026
GB49709	coiled-coil domain-containing protein 86-like	99.499	-0.083	0.182	-0.047	-0.025
GB42650	GPI mannosyltransferase 4-like	99.404	-0.536	0.058	0.101	0.098
GB42844	guanine nucleotide exchange factor DBS-like isoform X3	99.399	-0.390	-0.258	0.189	-0.004
GB47802	signal recognition particle receptor subunit beta	99.385	0.292	0.039	0.165	0.120
GB53403	uncharacterized LOC100577196, transcript variant X4	99.305	0.148	0.113	-0.006	0.245
GB48022	protein henna-like isoform X3	99.274	-0.362	-0.326	-0.063	0.109
GB54698	DNA-directed RNA polymerase I subunit RPA49-like isoform X3	99.037	-0.544	0.044	-0.159	0.078
GB52456	LOW QUALITY PROTEIN: protein DENND6A-like	98.901	0.242	0.217	0.152	0.035
GB42356	arginine-glutamic acid dipeptide repeats protein-like	98.809	-0.675	-0.096	0.189	0.042
GB55020	plexin-A4 isoform X3	98.802	-0.043	-0.090	0.074	-0.263
GB48708	sphingomyelin synthase-related 1	98.752	-0.100	-0.226	0.001	-0.035
727539	lysocardiolipin acyltransferase 1-like	98.713	0.163	0.022	-0.051	-0.038
GB54927	BRCA1-associated protein-like isoform X2	98.691	-0.172	-0.014	0.131	0.079
GB41029	cell cycle control protein 50A-like isoform X5	98.595	-0.130	-0.030	0.246	0.056
GB44149	proclotting enzyme isoform X1	98.592	-0.173	-0.090	1.551	-0.075
GB45947	pterin-4-alpha-carbinolamine dehydratase	98.521	0.166	-0.052	-0.060	-0.028
GB46112	replication factor C subunit 2	98.409	0.173	-0.566	-0.176	0.217
GB55662	mitochondrial fission process protein 1-like	98.299	0.152	-0.165	0.018	0.192
GB46775	bifunctional arginine demethylase and lysyl-hydroxylase PSR isoform X1	98.241	0.293	0.136	0.774	0.069
GB52799	methionine aminopeptidase 1D, mitochondrial-like	98.231	-0.152	0.073	0.168	0.231
GB52911	exosome complex component CSL4-like	98.127	0.348	0.041	-0.154	0.198
GB55264	probable serine/threonine-protein kinase DDB_G0283337-like	98.123	-0.186	-0.157	0.399	-0.016
GB45819	uncharacterized protein LOC725150	98.099	-0.445	-0.160	0.565	0.037
GB41392	heterogeneous nuclear ribonucleoprotein A1, A2/B1 homolog isoform X3	98.050	0.007	0.115	0.156	-0.047
GB51243	glutathione S-transferase omega-1	97.963	0.125	-0.038	0.077	-0.121
GB45684	protein spire-like isoform X4	97.717	-0.406	0.021	0.243	-0.182
GB53647	WD repeat-containing protein 75-like	97.642	0.281	0.047	-0.013	0.059
GB54752	breast cancer metastasis-suppressor 1-like protein-like isoform 1	97.583	0.055	0.141	-0.020	0.064
GB53821	telomerase-binding protein EST1A-like isoform X4	97.571	-0.924	-0.020	-0.513	-0.320
GB53131	acylglycerol kinase, mitochondrial-like	97.530	-0.352	0.123	-0.138	0.251
GB50772	uncharacterized protein LOC100578420 isoform X2	97.478	-0.531	0.114	-0.106	-0.044
GB50272	trans-1,2-dihydrobenzene-1,2-diol dehydrogenase-like isoform X5	97.445	0.046	-0.041	-0.134	-0.237
411763	DNA polymerase subunit gamma-1, mitochondrial	97.406	-0.739	-0.122	0.059	-0.082
GB55091	translation initiation factor eIF-2B subunit beta	97.358	0.200	0.223	-0.241	0.024
GB44689	E3 ubiquitin-protein ligase synoviolin A-like isoform X3	97.322	0.060	0.079	0.106	-0.005
100576966	reticulocyte-binding protein 2 homolog a-like isoform X2	97.303	-0.461	-0.108	0.095	0.138
GB48312	pre-mRNA-splicing factor RBM22-like	97.300	0.316	-0.014	0.094	-0.103
GB53208	uncharacterized LOC408620, transcript variant X2	97.152	0.152	0.089	0.184	-0.060
GB46689	kinesin 4A isoformX1	97.132	-0.458	-0.083	-0.060	-0.090
GB46653	GPI inositol-deacylase-like isoform X4	97.011	-0.339	-0.019	-0.035	-0.023
GB40590	metallo-beta-lactamase domain-containing protein 1-like isoform X2	96.922	0.250	0.134	0.236	0.152

(continued)

Gene	Name	k	am_fc	bt_fc	lf_fc	ln_fc
GB18213	meteorin precursor	96.885	0.174	0.185	0.171	0.108
GB40712	malectin-like	96.880	0.043	0.011	0.097	-0.215
GB55223	transcription initiation factor TFIID subunit 1 isoform X1	96.867	0.291	0.198	0.069	-0.036
GB46273	uncharacterized protein LOC409083	96.801	0.286	0.044	-0.285	0.078
GB41472	DNA-directed RNA polymerase III subunit RPC3-like	96.772	-0.019	-0.020	0.197	0.156
GB50322	WD repeat domain phosphoinositide-interacting protein 4-like isoform X2	96.746	0.046	-0.097	0.015	0.011
GB40415	protein brambleberry-like	96.619	-0.243	0.135	-0.193	0.082
GB49330	nicastrin isoform X1	96.608	0.154	-0.037	-0.106	0.035
724533	small nuclear ribonucleoprotein Sm D3 isoform X1	96.606	0.236	0.102	-0.160	0.017
GB55538	uncharacterized protein LOC409034	96.603	-0.454	0.081	0.842	-0.030
GB51129	cyclin-dependent kinase 20-like isoform 1	96.557	-0.645	0.029	0.136	-0.135
GB55824	probable serine hydrolase-like isoform X3	96.442	0.199	-0.153	-0.038	0.036
GB52029	eukaryotic translation initiation factor 3 subunit G-like	96.340	0.588	0.006	-0.259	0.069
GB47235	protein UBASH3A homolog	96.324	-0.085	-0.071	0.405	0.016
GB50552	oxysterol-binding protein-related protein 11-like	96.310	0.327	0.078	0.320	-0.006
GB45045	uncharacterized protein LOC552753 isoform X3	96.267	-0.460	0.106	0.117	0.026
GB55247	UDP-N-acetylglucosamine–peptide N-acetylglucosaminyltransferase 110 kDa subunit isoform 2	96.252	-0.172	-0.080	0.260	-0.185
GB54259	NAD-dependent protein deacetylase Sirt2 isoform X4	96.187	0.254	-0.032	-0.125	0.006
GB43990	peptidyl-tRNA hydrolase ICT1, mitochondrial-like	96.151	-0.088	0.080	0.031	-0.032
GB47304	5-formyltetrahydrofolate cyclo-ligase-like	96.146	-0.108	0.054	-0.024	-0.036
GB42066	kinesin 3D isoform X2	96.094	-0.118	0.116	-0.023	0.088
GB47191	uncharacterized protein LOC100576348 isoform X2	96.093	-0.648	0.349	-0.385	0.205
GB47105	nucleolar MIF4G domain-containing protein 1 homolog isoform X1	95.945	-1.041	0.143	0.055	-0.089
GB53240	E3 ubiquitin-protein ligase RNF25-like isoformX1	95.753	-0.276	0.082	-0.231	-0.058
GB45009	katanin p80 WD40 repeat-containing subunit B1 isoform X2	95.627	-0.464	0.098	-0.237	0.084
551467	syntaxin-18 isoform X2	95.610	-0.482	-0.099	-0.074	0.150
GB44492	isopentenyl-diphosphate Delta-isomerase 1-like isoformX1	95.609	-0.429	-1.284	-0.100	0.041
GB50012	uncharacterized protein LOC726323 isoform X1	95.602	0.327	-0.058	0.152	0.474
GB46218	transferrin isoform X4	95.581	0.062	-0.064	0.014	-0.206
GB52796	translin	95.465	0.172	0.086	-0.067	0.175
GB52198	LOW QUALITY PROTEIN: actin-binding protein anillin	95.443	0.146	0.101	0.210	-0.082
GB54837	protein MAK16 homolog A-like	95.367	-0.138	0.434	-0.160	-0.231
GB54388	RNA 3'-terminal phosphate cyclase-like isoform X1	95.281	0.303	0.065	-0.093	0.199
GB47315	mitochondrial thiamine pyrophosphate carrier-like isoform X2	95.181	-0.159	-0.087	-0.007	0.945
GB50365	ski oncogene	95.176	-0.230	-0.046	0.181	0.023
GB50962	POU domain protein CF1A-like	95.165	-1.125	-0.123	-0.099	-0.152
552237	transmembrane protein 242-like	95.084	-0.179	-0.072	0.028	-0.127
GB55485	DNA methyltransferase 3	95.081	-0.587	-0.080	-0.084	0.024
GB52744	poly(A) RNA polymerase, mitochondrial-like isoform X2	95.039	-0.531	0.036	-0.030	0.178
GB40446	unconventional prefoldin RPB5 interactor-like	95.038	-1.483	-0.003	0.258	0.160
GB53302	mini-chromosome maintenance complex-binding protein isoform 2	94.970	-0.295	0.008	0.408	0.076
GB42263	RNA-binding protein 40-like	94.942	-0.495	0.160	-0.166	0.310
GB44999	chascon-like	94.939	-0.633	-0.324	0.280	-0.211
GB53246	protein unc-119 homolog B	94.868	-0.035	0.072	0.120	0.053
GB46980	probable tRNA N6-adenosine threonylcarbamoyltransferase	94.811	0.132	-0.012	0.140	-0.060
GB51396	hydroxysteroid dehydrogenase-like protein 2-like isoform X2	94.773	-0.237	0.092	-0.096	0.025
GB41295	nogo-B receptor-like	94.756	0.096	0.038	0.066	-0.036
GB53289	uncharacterized protein LOC552029 isoform X1	94.539	0.507	-0.019	-0.535	-0.007
GB45378	thrombospondin-3 isoform X2	94.419	-0.077	-0.251	-0.260	0.017
GB49574	uncharacterized protein LOC552171 isoform X1	94.378	-0.425	-0.044	0.045	-0.152
GB45295	importin subunit alpha-2-like isoform X1	94.343	0.055	0.179	0.193	0.014
GB53676	protein smoothened isoform X2	94.307	-0.345	0.031	-0.516	0.262
GB53802	dipeptidyl peptidase 9-like	94.263	0.343	0.188	0.164	-0.041
GB42690	FAD-linked sulfhydryl oxidase ALR isoformX1	94.223	0.269	-0.004	0.012	0.124
GB40106	EGF domain-specific O-linked N-acetylglucosamine transferase-like isoform X5	94.159	0.115	0.110	0.020	-0.099
GB42206	uncharacterized protein LOC100578913 isoform X1	94.060	-0.726	0.270	-0.051	0.024

(continued)

Gene	Name	k	am_fc	bt_fc	lf_fc	ln_fc
GB41179	zinc finger protein 569-like	93.987	-0.394	-0.445	0.139	0.017
GB43121	uncharacterized protein F21D5.5-like isoform X2	93.949	-0.107	0.088	0.231	0.476
GB53418	HD domain-containing protein 2-like isoform X1	93.949	-0.081	-0.241	0.076	0.247
GB49100	probable tRNA pseudouridine synthase 2-like	93.894	-0.025	0.021	0.073	0.186
GB51541	protein Hook homolog 3-like isoform 1	93.827	-0.280	0.068	0.004	-0.147
GB40336	neutral ceramidase isoform X1	93.825	-0.217	-0.021	1.244	-0.173
GB50886	mediator of RNA polymerase II transcription subunit 30 isoform 1	93.761	0.281	0.038	-0.131	0.231
GB51900	surfeit locus protein 6 homolog	93.758	-0.643	-0.107	0.425	0.336
GB41517	uncharacterized protein LOC100578546 isoform X1	93.744	-0.707	-0.163	-0.060	0.012
GB49415	ATP synthase mitochondrial F1 complex assembly factor 2-like	93.694	-0.031	-0.104	-0.015	-0.096
GB51394	poly(ADP-ribose) glycohydrolase ARH3-like	93.569	0.060	0.085	0.020	0.096
GB45680	UPF0536 protein C12orf66 homolog isoform X2	93.560	-0.105	0.016	0.277	0.089
GB45492	protein misato-like isoform X1	93.515	0.007	-0.107	0.069	0.148
GB54242	ribonucleases P/MRP protein subunit POP1-like	93.487	-0.197	0.162	0.136	0.046
GB55560	SH2B adapter protein 1-like isoform X1	93.437	0.443	0.060	0.172	-0.049
GB52503	integrator complex subunit 7-like isoformX1	93.345	0.215	0.061	0.045	-0.078
GB55837	MLX-interacting protein isoform X2	93.186	-0.254	-0.155	-0.055	-0.045
102654737	peptide deformylase, mitochondrial-like	93.129	-0.255	0.385	0.015	0.189
GB51537	28S ribosomal protein S11, mitochondrial isoform X1	93.061	-0.247	0.050	0.055	0.133
GB53695	fatty-acid amide hydrolase 2-like, transcript variant X3	93.036	0.636	-0.227	-0.193	0.039
GB44799	uncharacterized protein LOC552039 isoform X1	92.778	-0.888	0.007	-0.014	-0.042
GB53716	estrogen sulfotransferase-like	92.751	-0.035	0.344	0.037	0.126
GB44659	uncharacterized protein LOC100577941 isoform X2	92.658	-0.320	0.115	0.101	0.134
GB51943	short coiled-coil protein homolog	92.653	0.241	-0.010	0.162	0.249
GB44886	glycine-rich cell wall structural protein 1.8-like isoform X1	92.649	0.210	0.160	0.121	0.243
GB46501	tRNA:m(4)X modification enzyme TRM13 homolog	92.540	-0.059	-0.113	0.082	-0.209
GB53387	GTP-binding protein Di-Ras2-like	92.496	-0.155	-0.054	0.509	-0.338
GB46920	iron-sulfur cluster assembly enzyme ISCU, mitochondrial	92.435	0.107	-1.031	0.115	-0.099
GB53861	mitochondrial tRNA-specific 2-thiouridylase 1-like	92.346	-0.408	-0.116	0.235	0.151
GB49516	calcium and integrin-binding protein 1-like isoform X2	91.973	0.156	0.034	0.231	0.132
GB49953	vacuolar protein sorting-associated protein 45 isoform 1	91.766	-0.480	-0.079	0.028	0.054
GB53532	serine/threonine-protein kinase STE20-like	91.607	0.397	-0.090	-0.063	-0.267
102656291	coiled-coil domain-containing protein 115-like	91.603	-0.080	-0.192	0.033	-0.161
GB40983	methylosome protein 50 isoform X2	91.343	0.382	0.155	0.012	-0.050
GB50063	DDB1- and CUL4-associated factor 10-like	91.312	0.033	0.131	-0.006	0.042
GB47651	dnaJ homolog subfamily B member 12-like	91.285	-0.178	-0.033	-0.169	0.060
GB42015	tubulin polyglutamylase TTLL7-like	91.238	-0.224	0.020	0.289	0.178
100578654	uncharacterized protein LOC100578654 isoform X4	91.119	-0.554	0.139	0.230	0.211
GB46452	DNA replication factor Cdt1 isoform X1	91.035	-0.135	0.284	-0.047	0.155
GB49629	ubiquitin carboxyl-terminal hydrolase isoform X2	90.931	0.419	-0.014	-0.075	-0.042
GB54293	uncharacterized protein LOC724457	90.747	-0.329	0.214	0.354	0.114
GB55801	uncharacterized protein LOC413738 isoform X2	90.637	-0.519	0.105	-0.200	-0.132
GB45688	synaptic vesicle membrane protein VAT-1 homolog-like	90.598	-0.303	0.126	0.180	-0.063
GB51635	centrin-1	90.588	-0.532	-0.118	0.076	0.008
GB48944	vesicle transport protein SEC20-like isoform X1	90.567	0.337	0.055	-0.003	0.256
GB46960	polypeptide N-acetylgalactosaminyltransferase 5-like isoform X1	90.557	-0.095	-0.001	0.679	0.033
GB50941	phosphatidate phosphatase LPIN2-like isoform X1	90.554	0.121	-0.110	0.071	-0.126
GB48677	protein O-mannosyl-transferase 2-like isoform X2	90.532	0.906	0.125	0.177	0.022
GB53015	protein FAM151A-like isoform X3	90.385	-0.685	0.200	0.063	0.010
GB42321	protein SGT1 homolog ecdysoneless isoform X1	90.345	-0.205	-0.039	-0.007	-0.010
GB41452	serine/threonine-protein phosphatase PGAM5, mitochondrial-like	90.279	0.090	0.312	-0.065	0.193
GB49540	dynactin subunit 4	90.246	0.165	0.082	0.135	-0.024
GB53847	abhydrolase domain-containing protein 2	90.213	0.169	0.012	0.031	0.041
GB50359	UHRF1-binding protein 1-like isoform X5	90.185	-0.040	-0.058	0.055	-0.341
GB49352	transmembrane protein 59-like	90.082	0.312	0.070	0.138	0.051
GB41792	putative uncharacterized protein DDB_G0282133-like isoform X2	90.022	-0.054	0.294	0.422	0.013
GB50004	protein preli-like isoform 1	90.014	-0.390	0.025	-0.042	0.062

(continued)

Gene	Name	k	am_fc	bt_fc	lf_fc	ln_fc
GB46748	39S ribosomal protein L54, mitochondrial	90.005	-0.184	0.084	-0.234	0.211
GB44210	contactin	89.791	-0.133	-0.091	0.116	-0.197
GB42265	nuclear envelope phosphatase-regulatory subunit 1-like isoform X2	89.750	0.235	0.084	0.206	0.030
GB46222	odorant binding protein 13 precursor	89.715	0.554	-3.469	0.121	-0.027
GB40717	B-cell CLL/lymphoma 7 protein family member B-like isoform X1	89.686	-0.090	0.274	0.063	0.082
GB43564	uncharacterized protein LOC100578443	89.671	-0.674	0.365	0.051	0.105
GB51518	probable serine/threonine-protein kinase dyrk2-like	89.378	0.000	0.012	0.144	0.234
GB51015	acylamino-acid-releasing enzyme-like isoform X2	89.371	0.020	0.137	0.133	0.090
GB52105	putative uncharacterized protein DDB_G0271606-like isoform X2	89.301	-0.441	0.555	-0.132	0.290
GB43246	uncharacterized protein KIAA0513-like isoform X2	89.294	-0.850	0.092	0.144	0.112
GB45456	flocculation protein FLO11-like isoform X2	89.264	-0.142	-0.053	0.422	-0.137
GB48061	uncharacterized protein LOC413052	89.264	-0.146	0.213	-0.228	0.026
GB52998	radial spoke head protein 9 homolog	89.197	-0.397	-0.690	0.227	-0.124
GB49284	mucolipin-3 isoform 2	89.132	0.125	0.034	0.075	0.068
GB55092	suppressor of fused homolog	89.127	-0.433	0.104	0.146	-0.162
GB41260	uncharacterized protein LOC414002	89.043	0.178	0.001	-0.383	0.921
GB40269	nucleolar protein 14 homolog isoform X1	89.003	-0.074	-0.088	-0.123	0.337
GB44800	f-box only protein 33-like, transcript variant X3	88.978	-0.117	0.054	0.047	0.033
GB42086	single-strand selective monofunctional uracil DNA glycosylase-like	88.965	0.199	-0.059	0.205	0.216
102655420	uncharacterized protein LOC102655420	88.838	-0.265	0.105	0.304	0.773
GB46741	growth hormone-regulated TBC protein 1-A isoform X1	88.753	-0.183	0.124	0.130	0.303
GB52652	uncharacterized protein LOC724152	88.676	0.056	0.296	0.110	0.297
GB40796	phospholipase DDHD1-like isoform X3	88.640	-0.026	-0.076	0.017	-0.234
GB50173	TATA box-binding protein-associated factor RNA polymerase I subunit B-like	88.635	-0.937	-0.151	0.036	0.154
GB40067	dual serine/threonine and tyrosine protein kinase isoform X1	88.601	-0.119	0.098	0.050	-0.071
GB47768	rac GTPase-activating protein 1-like	88.576	-0.006	0.245	0.440	0.345
GB43480	cleavage and polyadenylation specificity factor 160, transcript variant X2	88.499	0.205	0.146	-0.083	-0.065
GB51106	mRNA turnover protein 4 homolog	88.489	0.329	0.105	-0.143	0.204
GB55371	probable asparagine–tRNA ligase, mitochondrial-like isoform X1	88.468	-0.101	0.018	0.039	0.212
GB41829	ubiquitin-like modifier-activating enzyme atg7-like isoform X3	88.330	-0.035	-0.078	0.150	0.044
GB48636	exosome complex component RRP46	88.276	0.267	-0.035	0.087	0.044
GB40941	ornithine decarboxylase-like isoform X1	88.201	0.375	1.875	0.091	0.940
GB43551	uncharacterized protein LOC409563	88.158	-0.119	0.094	-0.067	-0.060
GB44054	DNA replication complex GINS protein PSF1-like	88.127	0.251	0.091	-0.072	0.247
GB45830	kinesin 8	87.881	-1.662	0.280	0.581	0.056
GB50352	glutathione synthetase isoform X1	87.640	-0.508	0.041	2.133	0.086
GB47992	splicing factor 3B subunit 4	87.546	-0.634	0.164	-0.034	-0.005
GB41979	UTP–glucose-1-phosphate uridylyltransferase isoform X3	87.538	-0.323	-0.028	0.063	0.066
GB51421	CTP synthase, transcript variant X4	87.447	-0.357	0.058	-0.069	0.139
GB49449	uncharacterized protein LOC409179 isoform 1	87.431	-0.438	-0.840	0.367	-0.062
GB46928	lys-63-specific deubiquitinase BRCC36-like isoform X2	87.410	0.148	0.160	0.196	0.189
GB49123	uncharacterized protein LOC100577638 isoform X1	87.389	0.017	0.163	-0.332	-0.079
GB44058	UDP-glucuronosyltransferase 1-3-like	87.277	-0.083	0.061	0.271	0.243
GB45674	protein aurora borealis	87.210	-0.633	0.197	0.164	0.596
GB53826	transcription factor E2F4 isoform X1	87.000	0.476	-0.028	0.527	-0.017
GB55002	uncharacterized protein LOC100578883	86.998	-0.263	0.035	0.257	0.084
GB40022	hydroxylysine kinase-like isoform X1	86.982	0.558	0.174	-0.043	0.085
GB44434	probable glucosamine 6-phosphate N-acetyltransferase-like	86.981	-0.106	0.008	0.127	0.081
GB52653	farnesyl pyrophosphate synthase-like	86.956	-0.509	-1.359	0.056	0.132
GB55007	serine protease snake isoform X3	86.762	0.010	-0.081	-0.055	1.064
GB49384	uncharacterized protein LOC725157	86.382	-0.016	0.306	0.967	-0.202
GB55527	uncharacterized protein LOC408649 isoform X2	86.335	-0.148	-0.133	-0.343	-0.306
GB49539	toys are us	86.264	0.001	0.056	0.246	0.046
GB41894	uncharacterized protein LOC411277 isoform X28	85.976	-0.536	0.234	0.095	-0.205

(continued)

Gene	Name	k	am_fc	bt_fc	lf_fc	ln_fc
GB44703	proteasome activator complex subunit 4-like	85.829	-0.282	-0.159	-0.415	-0.124
GB54462	protein LLP homolog	85.703	-0.307	0.041	-0.257	-0.005
GB46617	rhythmically expressed gene 2 protein-like isoform X1	85.658	0.012	-0.061	0.133	-0.061
102655268	WD repeat-containing protein 63-like	85.602	-0.170	-0.073	-0.082	0.095
GB40251	uncharacterized protein LOC552030 isoform X1	85.189	0.291	-0.007	-0.390	0.070
GB49343	transcriptional repressor protein YY1-like isoform 1	85.138	0.331	0.031	0.009	-0.013
GB55317	protein lunapark-B-like, transcript variant X2	85.094	-0.050	0.141	-0.235	0.008
GB47319	zinc finger protein Elbow-like isoform X1	84.907	-0.012	0.109	-0.036	-0.138
GB47460	cell division cycle protein 20 homolog isoform X1	84.834	-0.318	0.166	-0.345	0.133
GB40480	serine/threonine-protein kinase OSR1-like isoform X5	84.824	-0.257	0.027	0.190	0.083
GB45040	Krueppel-like factor 10-like isoform X1	84.472	-0.500	-0.116	0.131	-0.112
GB51863	flotillin-2 isoform X2	84.406	-0.328	-0.205	0.021	-0.010
GB45609	flavin-containing monooxygenase FMO GS-OX-like 4-like	84.048	-0.805	-0.208	-0.402	-0.057
GB42054	sodium/potassium-transporting ATPase subunit alpha isoform X5	83.979	-0.289	-0.056	0.178	0.093
GB50877	tyrosine-protein kinase Dnt isoform X1	83.942	-0.519	-0.156	0.204	0.094
GB48118	negative elongation factor E	83.789	-0.071	0.130	0.151	0.048
GB45703	uncharacterized aarF domain-containing protein kinase 1-like isoform 2	83.675	-0.044	-0.097	-0.318	-0.145
GB53953	mitochondrial coenzyme A transporter SLC25A42-like isoformX1	83.475	-0.688	-0.241	0.131	0.131
GB45913	protein lethal(2)essential for life-like	83.415	0.284	0.457	-0.049	-0.244
GB47106	NADH-ubiquinone oxidoreductase 75 kDa subunit, mitochondrial	83.401	-0.252	0.000	0.275	-0.035
GB53257	muscle, skeletal receptor tyrosine protein kinase-like isoform X1	83.267	0.157	-0.200	-0.043	0.011
GB51487	proton-coupled amino acid transporter 4-like	83.253	-0.269	-0.009	0.088	-0.019
GB48234	tyrosine-protein phosphatase Lar-like	83.239	-0.236	-0.128	-0.907	0.093
GB45046	cell division control protein 6 homolog	83.213	-0.143	0.077	-0.540	0.019
GB51937	hiiragi, transcript variant X4	83.149	0.327	-0.233	-0.019	0.097
GB54689	hippocampus abundant transcript 1 protein-like isoform X6	83.131	0.090	0.008	0.108	-0.402
GB54282	kinetochore protein NDC80 homolog	83.120	-0.190	0.071	-0.185	0.285
GB54319	synaptotagmin 20 isoform X5	83.080	-0.013	-0.097	0.104	-0.067
GB47468	integrin alpha-8 isoform X1	83.071	-0.251	-0.225	0.065	-0.156
102656381	ceramide phosphoethanolamine synthase-like	83.056	-0.581	-0.263	0.018	0.055
GB40535	39S ribosomal protein L48, mitochondrial isoform X4	83.044	-0.486	-0.078	-0.019	0.016
GB49071	uncharacterized protein LOC725791	82.956	-0.282	0.191	-0.138	0.951
551892	cytoplasmic protein NCK1 isoform X3	82.845	0.112	0.027	0.158	-0.059
GB43095	BMP-binding endothelial regulator protein isoform X1	82.817	-0.641	-0.158	0.111	0.139
102656425	CDGSH iron-sulfur domain-containing protein 3, mitochondrial-like	82.778	0.035	-0.051	0.021	0.164
GB46580	protein wntless-like isoform X1	82.769	0.370	0.100	-0.036	0.194
GB53185	GPN-loop GTPase 3-like	82.731	0.064	0.011	0.229	0.103
GB46630	uncharacterized protein LOC100577504 isoform X1	82.691	-0.564	-0.046	0.630	0.001
GB40904	uncharacterized protein LOC725211	82.666	-0.446	0.177	0.045	0.117
GB45195	protein scribble homolog	82.556	0.288	-0.008	0.162	-0.194
GB48483	chaoptin-like isoform X2	82.501	0.144	0.192	0.077	0.198
GB40963	putative transcription factor SOX-15 isoform X3	82.433	0.120	-0.197	-0.010	-0.491
GB51348	rootletin-like isoform X3	82.400	-0.538	-0.118	0.816	-0.121
GB42808	neuroligin 5	82.328	-1.955	0.416	-0.380	-0.284
GB42555	uncharacterized protein LOC100577221	82.255	-0.216	0.265	0.036	0.083
GB44044	intraflagellar transport protein 57 homolog isoform X3	82.252	0.430	-0.038	1.478	-0.053
GB48776	coiled-coil domain-containing protein 6-like	82.242	0.064	0.143	-0.061	-0.657
GB42110	slowpoke-binding protein isoform X6	82.180	0.252	0.062	0.398	0.259
552443	N-alpha-acetyltransferase 60-like	82.157	0.133	0.186	0.212	-0.044
GB50784	zinc finger matrin-type protein 5-like	82.103	0.385	-0.039	0.123	0.049
GB51513	UPF0183 protein CG7083-like	82.054	0.021	0.110	-0.368	0.173
GB47815	haloacid dehalogenase-like hydrolase domain-containing protein 2-like	82.048	0.234	0.217	0.587	0.150
GB46467	uncharacterized protein LOC726694 isoform X2	82.023	0.466	0.114	0.000	0.161
GB48380	major facilitator superfamily domain-containing protein 8-like isoform X2	81.985	0.085	0.126	0.104	-0.170

(continued)

Gene	Name	k	am_fc	bt_fc	lf_fc	ln_fc
GB48321	vanin-like protein 1-like	81.909	0.236	-0.094	0.163	0.060
GB42192	metallophosphoesterase domain-containing protein 1-like isoform 1	81.821	0.144	0.020	0.266	0.391
GB47740	leucine-rich repeat-containing G-protein coupled receptor 4-like	81.667	-0.530	-0.276	-0.215	0.389
GB40704	dynein intermediate chain 2, ciliary-like isoform X5	81.595	-0.772	-0.042	0.305	0.168
GB42183	uncharacterized protein LOC100578600	81.248	-0.241	0.005	0.375	-0.230
GB45851	dnaJ homolog subfamily C member 22-like	81.145	0.072	-0.004	-0.035	-0.256
GB53539	thyroid transcription factor 1-associated protein 26 homolog	81.067	0.078	0.136	0.023	0.066
GB43729	rac guanine nucleotide exchange factor JJ	81.053	-0.047	0.497	0.117	0.326
GB54190	uncharacterized aarF domain-containing protein kinase 4 isoform X4	80.865	-0.146	-0.058	0.070	0.115
GB54421	uncharacterized protein DDB_G0287625-like	80.684	0.256	-0.022	0.023	0.034
GB44117	roundabout homolog 2 isoform X3	80.623	-0.244	-0.158	0.553	-0.275
724756	uncharacterized protein LOC724756	80.557	0.025	-0.725	0.060	0.043
GB54653	U1 small nuclear ribonucleoprotein A	80.553	0.454	-0.056	0.001	0.169
GB55296	pyroglutamyl-peptidase 1-like	80.518	0.580	0.074	0.191	-0.083
GB43159	NF-X1-type zinc finger protein NFXL1-like	80.362	-0.148	0.121	-0.243	-0.037
GB45139	uncharacterized protein LOC726903	80.312	-1.337	0.560	0.135	0.027
GB52666	putative uncharacterized protein DDB_G0282133-like	79.969	-1.060	-0.421	0.512	-0.155
GB52601	AT-rich interactive domain-containing protein 5B-like isoform X3	79.819	0.063	0.167	0.069	0.029
GB42231	beta-1,4-N-acetylgalactosaminyltransferase bre-4 isoform X3	79.701	-0.373	0.009	-0.123	0.254
GB54758	WD repeat, SAM and U-box domain-containing protein 1-like isoform X1	79.656	0.042	-0.282	-0.167	-0.009
GB44387	B-cell lymphoma/leukemia 11B-like isoform X3	79.548	-0.545	0.142	0.433	-0.341
GB55755	protein outspread	79.451	-0.086	0.101	0.173	0.061
GB42526	malate dehydrogenase, mitochondrial-like isoform 1	79.380	-0.247	-0.234	0.027	0.080
GB40770	dehydrogenase/reductase SDR family member 11-like isoform X2	79.183	0.033	0.354	0.387	0.167
GB43220	transcription termination factor 2 isoform X1	79.103	0.264	0.276	0.054	-0.143
GB53378	zinc finger protein 28 homolog	79.024	-0.579	-0.155	0.011	-0.158
GB52797	enhancer of polycomb homolog 1	78.900	0.178	0.048	-0.019	0.123
GB45255	aurora kinase B isoform X1	78.875	0.169	0.148	-0.154	0.179
GB47938	uncharacterized protein LOC412825 isoform X1	78.849	-0.421	-0.004	0.758	-0.248
GB44203	arrestin domain-containing protein 3	78.790	0.250	0.209	0.426	0.078
GB45714	transglutaminase	78.609	-0.397	-0.018	0.096	-0.030
GB50669	uncharacterized protein LOC410428	78.547	-0.037	-0.722	0.111	-0.218
GB51714	stimulator of interferon genes protein-like	78.445	0.019	-0.023	0.020	-0.026
GB47159	caspase-1-like	78.366	-0.297	0.066	0.095	-0.189
GB52077	period circadian protein	78.261	-0.128	0.116	-1.166	0.648
GB43261	ubiquitin thioesterase trabid isoform X1	78.205	-0.448	0.092	-0.007	0.135
GB44781	exocyst complex component 4-like	78.176	0.421	0.023	0.199	0.272
GB41417	leucine-rich repeats and immunoglobulin-like domains protein 1-like	78.123	-0.280	0.022	0.168	-0.300
GB49988	SRR1-like protein-like isoform X2	78.056	-0.800	0.006	0.083	0.119
GB44443	transmembrane protein 179-like isoform X1	78.041	-0.032	0.017	0.058	0.006
GB47331	programmed cell death protein 5-like	78.040	0.198	0.052	0.264	0.104
GB54851	zinc transporter 1-like isoform X7	77.948	0.095	0.233	0.432	0.125
GB45058	MIP18 family protein CG7949-like	77.881	0.135	0.272	0.135	0.307
GB40750	putative ATP-dependent RNA helicase me31b-like isoform 1	77.618	-0.170	0.165	0.085	-0.077
GB54158	intraflagellar transport protein 140 homolog	77.564	0.183	0.075	0.141	-0.221
GB45420	graves disease carrier protein homolog	77.457	-0.136	0.142	0.121	0.004
GB40312	choline/ethanolamine kinase-like isoform X4	77.275	-0.464	-0.239	0.030	-0.129
GB51242	uncharacterized protein LOC727370 isoform X3	77.253	-0.136	-0.028	0.331	-0.279
GB49870	long-chain-fatty-acid–CoA ligase 6-like isoform X3	77.099	0.181	-0.075	0.041	-0.218
GB46145	sodium/potassium-transporting ATPase subunit beta-2-like	77.043	0.270	-0.183	0.172	-0.041
GB53531	A disintegrin and metalloproteinase with thrombospondin motifs 7-like isoform X9	76.937	-0.880	0.376	0.034	-0.094
GB42500	peptidoglycan-recognition protein LC isoform X2	76.795	0.337	-0.110	0.481	0.037
GB52154	protein prenyltransferase alpha subunit repeat-containing protein 1-like isoform X1	76.786	0.392	0.019	-0.324	0.119

(continued)

Gene	Name	k	am_fc	bt_fc	lf_fc	ln_fc
GB45052	LOW QUALITY PROTEIN: ral guanine nucleotide dissociation stimulator	76.777	0.001	0.121	0.121	-0.145
GB46038	elongation of very long chain fatty acids protein 4-like isoform X2	76.743	-0.137	0.327	0.250	-0.120
GB43831	ATP-binding cassette sub-family D member 3-like	76.668	0.078	-0.341	-0.157	0.066
GB41921	adenylate cyclase type 5-like	76.269	-0.070	-0.125	0.238	0.037
GB54100	ragulator complex protein LAMTOR4 homolog isoform X2	76.132	0.300	0.013	0.155	0.059
GB50016	cell division cycle-associated protein 7-like	76.130	-0.144	0.268	-0.080	0.222
GB44328	dynein light chain 1, axonemal-like isoform X2	76.108	0.098	-1.100	0.040	0.026
GB52882	calcium-independent phospholipase A2-gamma-like isoform X1	76.028	-0.625	-0.240	0.087	0.077
GB48310	zinc transporter ZIP1-like isoform X2	75.837	-0.284	-0.217	0.107	-0.033
GB42276	uncharacterized protein LOC550958	75.798	-0.392	0.049	0.283	0.053
GB43427	homer protein homolog 2-like isoform X2	75.655	-0.132	0.023	0.059	0.409
GB45106	run domain Beclin-1 interacting and cysteine-rich containing protein-like isoform X1	75.610	-0.249	0.033	0.106	-0.256
GB46056	heart- and neural crest derivatives-expressed protein 1-like isoform X1	75.470	-0.289	-0.058	0.300	-0.042
GB50933	GATA-binding factor A	75.273	0.470	0.171	0.186	0.188
GB46490	rho guanine nucleotide exchange factor 10-like isoform X3	75.258	-0.154	-0.020	-0.054	0.105
GB49469	lymphocyte cytosolic protein 2-like isoform X1	75.092	-0.620	0.073	-0.050	0.170
GB46245	integrator complex subunit 12 isoform X5	75.068	-0.180	0.019	-0.211	0.151
GB55192	sorting nexin-16-like	75.064	0.327	-0.092	0.094	0.309
GB53518	28S ribosomal protein S30, mitochondrial	75.012	-0.310	1.494	-0.276	0.234
GB50282	glycine receptor subunit alpha-2 isoform X8	74.986	-0.092	-0.123	0.254	0.147
GB53662	protein HID1-like isoformX1	74.843	0.306	0.135	-0.219	0.189
GB55604	uncharacterized protein LOC725144 isoform X4	74.636	0.154	-0.307	0.139	0.485
GB44440	FERM domain-containing protein 8 isoform 1	74.577	0.394	-0.073	0.188	0.025
GB44337	DNA polymerase delta small subunit isoform X2	74.552	-0.209	0.006	-0.281	0.150
413046	sarcolemmal membrane-associated protein-like	74.474	-0.257	0.033	0.027	-0.390
GB52073	probable citrate synthase 1, mitochondrial-like	74.380	-0.402	-0.159	-0.063	0.189
GB48829	blood vessel epicardial substance-like, transcript variant X4	74.359	0.190	-0.071	0.056	0.023
GB42719	E3 ubiquitin-protein ligase MARCH8-like isoform X1	74.118	0.349	-0.092	0.251	0.061
GB52025	neprilysin 2 isoform X4	74.074	0.374	-0.138	0.252	0.058
GB46395	40S ribosomal protein S12, mitochondrial	74.035	0.337	-0.054	0.172	0.060
GB42478	CD2-associated protein	73.863	-0.001	0.070	0.174	-0.083
GB50290	uncharacterized protein LOC724917 isoform X2	73.841	0.226	0.350	0.270	-0.035
GB49084	dual specificity protein kinase TTK-like	73.756	0.439	0.177	-0.323	0.030
GB49769	putative tRNA (cytidine(32)/guanosine(34)-2'-O)-methyltransferase-like isoform 1	73.579	0.351	-0.023	-0.881	0.379
724643	borealin-like	73.268	0.084	-0.069	-0.054	-0.057
GB51504	solute carrier family 23 member 1-like	73.116	0.084	0.375	0.092	-0.147
GB51118	28S ribosomal protein S28, mitochondrial	73.098	0.030	0.082	-0.543	0.002
GB51739	alpha-ketoglutarate-dependent dioxygenase alkB homolog 4-like	73.036	0.274	0.147	0.055	-0.225
GB55746	myosin-IB isoform X2	72.930	0.013	-0.052	-0.027	-0.294
GB42156	enhancer of filamentation 1	72.907	-0.529	0.137	-0.201	-0.097
GB41773	proteoglycan 4-like isoform X1	72.903	0.252	-0.691	0.192	0.182
GB50978	general transcription factor IIE subunit 1	72.884	0.551	-0.001	0.066	0.130
GB50435	zinc finger and BTB domain-containing protein 20-like	72.488	-0.679	0.073	0.398	0.228
GB46368	15-hydroxyprostaglandin dehydrogenase [NAD(+)]-like	72.350	0.030	-0.441	-0.235	-0.312
725196	putative peptidyl-tRNA hydrolase PTRHD1-like isoformX2	72.052	0.398	-0.002	0.303	0.050
GB51558	dentin sialophosphoprotein-like isoform X2	71.895	-0.397	-0.114	-0.220	0.064
GB48836	uncharacterized protein LOC100577578 isoform X1	71.835	0.129	0.104	0.072	-0.016
GB50460	ras association domain-containing protein 2	71.786	0.459	0.502	0.047	0.120
GB55840	protein sidekick-1-like isoform X5	71.586	0.185	0.007	-0.058	-0.042
GB41486	putative neutral sphingomyelinase-like isoform X1	71.456	-0.003	-0.053	0.098	0.088
GB50407	uncharacterized protein LOC100578579 isoform X2	71.069	-0.744	0.189	1.007	0.261
GB51409	homeobox protein Nkx-6.1-like	70.823	-0.911	-0.956	0.292	0.214
GB54525	ras-like protein family member 11B-like	70.737	0.048	-0.121	0.101	-0.174
GB46537	cell death-related nuclease 6 isoform X1	70.643	0.085	-0.077	0.048	0.212

(continued)

Gene	Name	k	am_fc	bt_fc	lf_fc	ln_fc
GB55909	uncharacterized protein LOC725992 isoform X1	70.565	-0.315	-0.117	0.884	-0.046
102656840	probable cyclin-dependent serine/threonine-protein kinase DDB_G0292550-like	70.505	-0.400	0.225	0.154	0.044
GB42872	G1/S-specific cyclin-E	70.253	0.191	0.303	-0.024	0.212
GB53943	tRNA methyltransferase 112 homolog	70.189	0.037	0.189	-0.233	0.158
GB45525	kin of IRRE-like protein 3-like isoform X2	70.042	-0.964	-0.142	0.162	0.094
100577213	tumor necrosis factor receptor superfamily member 10B-like	70.011	-1.145	-0.169	0.511	-0.178
GB50990	protein FAM76A-like isoform X3	69.283	0.323	0.002	0.149	0.078
GB45815	SCY1-like protein 2-like isoform X2	69.109	-0.067	-0.060	-0.440	0.422
GB40781	fibroblast growth factor receptor substrate 2-like	68.905	-0.317	0.233	0.755	0.057
GB47057	protein tramtrack, alpha isoform-like isoform X7	68.792	-0.261	0.027	0.067	0.400
GB51817	glucose dehydrogenase [FAD, quinone]	68.699	-2.564	0.738	0.323	0.156
GB43825	lysosomal aspartic protease	68.480	0.602	0.094	0.862	1.896
GB40871	protein bric-a-brac 1-like	68.319	-0.467	0.025	1.044	0.089
GB55299	serine/threonine-protein kinase PAK mbt isoform X3	68.100	-0.157	0.137	0.100	0.041
GB41414	titin-like isoform X3	67.979	0.223	-0.313	0.767	0.402
GB47470	cadherin-related tumor suppressor	67.920	-1.283	0.060	0.609	-0.621
GB45841	ras guanine nucleotide exchange factor P-like isoform X2	67.567	0.277	-0.006	0.062	-0.065
GB54522	Bardet-Biedl syndrome 2 protein homolog isoform X2	67.193	0.188	-0.124	0.369	-0.046
GB51515	uncharacterized LOC100577174, transcript variant X4	66.925	0.049	0.124	0.096	-0.093
GB44462	cytochrome c oxidase subunit 5A, mitochondrial	66.921	-0.379	-0.216	0.690	0.179
GB52039	hairy/enhancer-of-split related with YRPW motif protein 1-like isoform X4	66.839	-0.709	-0.103	0.204	-0.067
GB50050	ADP-ribosylation factor-like protein 3-like	66.792	-0.076	-0.182	0.023	0.131
GB52504	lachesin-like isoform X4	66.460	-0.402	0.470	0.081	-0.381
GB49115	probable low affinity copper uptake protein 2-like isoform X1	66.205	-0.271	-0.118	-0.349	0.159
GB53788	tyrosine-protein phosphatase non-receptor type 9-like isoform 1	66.099	0.067	0.112	0.441	0.229
GB47804	peptidoglycan-recognition protein LB isoform X2	65.957	0.010	-0.001	0.170	0.010
GB55486	histone lysine demethylase PHF8-like isoform X3	65.601	0.142	-0.035	0.166	0.046
GB51014	EF-hand domain-containing protein CG10641-like isoform X2	65.290	0.361	0.172	-0.038	-0.010
GB51418	uncharacterized protein LOC725844	65.263	0.152	-0.160	0.862	0.035
GB54359	uncharacterized protein LOC726251 isoform X1	65.245	0.850	-0.142	-0.026	-0.057
GB42894	disheveled-associated activator of morphogenesis 1 isoform X1	65.089	0.039	-0.119	0.458	0.149
GB42330	protein DPCD-like	64.814	0.287	0.149	-0.071	0.109
GB54886	LOW QUALITY PROTEIN: protein GDAP2 homolog	64.724	0.235	-0.047	0.084	-0.431
102655356	thyrotropin-releasing hormone receptor-like	64.716	0.471	0.358	0.293	0.306
102656439	adenosine deaminase CECR1-like	64.651	-0.150	-0.046	1.283	-0.241
GB18327	pyrokinin-like receptor 2	64.498	-0.712	-0.304	0.039	-0.127
GB52279	neurexin 1 precursor	64.304	-0.945	0.220	0.476	0.420
GB45547	uncharacterized protein LOC726958	64.298	0.293	-0.047	0.218	0.208
GB45870	uncharacterized protein LOC551818	63.897	-0.190	0.053	0.361	0.182
GB55531	rab11 family-interacting protein 1 isoform X2	63.845	0.210	-0.009	0.095	-0.031
102655904	DNA-directed RNA polymerases I, II, and III subunit RPABC3-like isoform X6	63.815	0.388	-0.073	0.224	-0.080
GB50181	RCC1 domain-containing protein DDB_G0279253-like	63.567	0.153	-0.039	0.095	-0.196
GB50525	serine/arginine repetitive matrix protein 2-like isoform X3	63.490	-0.232	-0.134	0.199	-0.147
GB47185	uncharacterized protein LOC551433	63.480	-0.990	-0.924	0.281	0.070
GB47838	flocculation protein FLO11-like	63.330	-0.646	-0.309	1.013	-0.339
GB44072	E3 ubiquitin-protein ligase TRIM9 isoform X2	63.248	-0.711	0.120	0.334	-0.163
GB42062	uncharacterized protein LOC724563	63.019	-0.016	-0.280	0.209	0.210
GB40876	pleckstrin homology domain-containing family J member 1-like	62.950	-0.329	0.130	-0.036	0.194
GB44045	protein croquemort isoform X6	62.793	-0.216	0.016	-0.299	0.033
GB41975	cyclin-Y	62.619	-0.242	0.036	-0.061	0.139
GB45875	G-protein coupled receptor Mth2-like isoform X2	62.434	0.382	-0.151	0.821	0.313
724832	innexin inx2	61.707	0.152	0.193	-0.319	0.147
GB46541	B(0,+)-type amino acid transporter 1-like isoform X2	61.507	0.068	-0.023	0.251	-0.160
GB49738	photoreceptor-specific nuclear receptor isoform X1	60.929	0.241	-0.289	0.162	-0.257
GB52417	spatacsin, transcript variant X2	60.764	-0.216	-0.069	1.143	0.078

(continued)

Gene	Name	k	am_fc	bt_fc	lf_fc	ln_fc
GB49655	uncharacterized protein LOC726100 isoform X1	60.685	-0.040	-0.021	-0.007	0.209
GB43909	plasma membrane calcium-transporting ATPase 3 isoform X1	60.423	-0.072	-0.068	0.044	-0.041
GB42412	tubulin delta chain-like	59.984	-0.813	-0.110	0.022	0.038
GB49807	cell cycle checkpoint protein RAD1	59.592	-0.179	0.065	0.192	0.220
GB50620	peptidyl-prolyl cis-trans isomerase-like	59.190	0.016	0.035	-0.120	0.220
GB43130	single Ig IL-1-related receptor-like	58.933	0.170	0.219	-0.019	-0.293
GB50648	serine proteinase stubble isoform X1	58.740	-0.925	-0.089	0.323	0.409
GB52947	protein mesh-like isoform X2	58.654	0.616	0.171	0.136	0.281
GB41734	reversion-inducing-cysteine-rich protein with kazal motifs isoform X2	58.587	-0.510	0.054	0.216	0.106
102656594	uncharacterized protein LOC102656594	58.469	0.101	0.006	0.321	0.009
GB46060	UPF0501 protein KIAA1430 homolog	58.307	0.025	0.128	0.215	-0.136
GB41591	putative glucose-6-phosphate 1-epimerase-like	57.829	0.273	0.173	0.107	0.213
GB46376	protein LSM14 homolog A isoform X1	57.778	-0.036	0.251	0.058	0.292
GB51736	tweedle motif cuticular protein 2	57.413	-0.171	0.332	0.182	0.485
GB42758	forkhead box protein D3-like	57.213	-0.235	-0.011	0.092	-0.188
GB40799	protein HOS4-like isoform X1	57.076	-0.093	0.065	0.453	0.159
GB54778	CAD protein isoform 1	56.949	-0.132	0.164	0.884	-0.336
GB44464	ubiquinone biosynthesis protein COQ7	56.739	0.263	0.048	0.179	-0.117
GB52756	apyrase precursor	56.414	-0.083	-0.109	0.058	0.060
GB53625	uncharacterized protein LOC411622	56.379	0.140	-0.290	0.156	0.032
GB45073	fibrillin-2-like	55.388	-0.264	-0.345	0.776	-0.677
GB51613	uncharacterized protein LOC408570 isoform X1	55.337	0.555	-0.155	1.091	0.585
GB54268	nicotinate phosphoribosyltransferase-like isoform X3	55.131	0.015	-0.026	-0.058	0.020
GB49416	protein msta, isoform A-like isoform X3	54.018	0.016	-0.187	0.566	-0.593
GB46398	thyrotroph embryonic factor isoformX1	53.627	-0.931	0.238	-0.098	-0.010
GB55516	bone morphogenetic protein 2-B isoform X2	53.338	-0.006	0.040	0.219	0.501
GB51331	tektin-4-like	53.263	0.240	-0.145	-0.011	0.174
GB45403	innexin inx1-like	53.227	0.006	0.170	0.255	0.087
GB47970	alpha-aminoadipic semialdehyde synthase, mitochondrial	53.226	0.004	-0.096	0.014	0.034
GB52700	uncharacterized protein LOC410520	53.181	-1.560	-0.046	-0.076	-1.077
GB54499	molybdenum cofactor sulfurase-like isoform X3	53.069	0.029	-0.072	0.070	0.112
GB51938	leucine rich repeat G protein coupled receptor	53.017	-0.404	0.505	0.189	0.050
GB49862	uncharacterized protein LOC724773 isoform X1	52.859	-0.524	-0.254	0.161	-0.232
GB50257	uncharacterized protein LOC408508 isoform X1	52.807	-0.036	0.181	-0.408	0.242
GB50129	tektin-3-like	52.584	-1.076	-0.346	1.345	0.072
102656337	mitotic spindle assembly checkpoint protein MAD2A-like isoform X1	52.345	0.279	0.172	-0.056	0.361
102653588	cleavage and polyadenylation specificity factor 73-like	51.696	-0.139	-0.053	1.960	-0.104
GB43158	nuclear pore complex protein Nup205	51.156	0.053	0.150	0.801	0.062
GB51560	histone-lysine N-methyltransferase 2D-like isoform X1	51.141	0.587	1.130	0.593	-0.085
GB54295	beta-1-syntrophin isoform X3	51.071	-0.596	0.203	-0.878	-0.499
GB50745	sphingosine kinase 2-like isoform X3	51.028	0.036	-0.173	0.209	-0.185
GB47849	pyrroline-5-carboxylate reductase 2-like isoform X2	50.912	-0.008	-0.243	0.025	0.611
GB40437	adenomatous polyposis coli protein-like	50.524	-0.457	0.083	-0.038	-0.194
GB55505	open rectifier potassium channel protein 1-like	50.459	-0.180	-0.183	0.102	-0.051
GB52791	ammonium transporter 1-like	50.269	-0.651	-0.071	0.231	0.282
100578205	uncharacterized protein LOC100578205	50.239	-1.000	0.168	-0.274	0.506
GB48271	broad-complex isoform X10	50.199	-0.326	-0.912	0.366	0.368
GB42812	uncharacterized SDCCAG3 family protein-like	49.484	-0.054	-0.103	-1.076	0.042
GB52614	REST corepressor 3 isoformX2	49.114	0.404	0.153	0.128	0.136
GB43877	aquaporin AQPcic-like isoform X2	48.978	0.274	0.126	0.363	-0.008
GB48543	uncharacterized protein LOC100577936 isoform X5	48.832	-0.490	-0.212	-0.101	-0.260
GB41027	BAG family molecular chaperone regulator 2-like	48.747	0.252	0.039	-0.397	0.092
GB41583	cysteine-rich PDZ-binding protein	48.721	0.159	0.046	0.189	0.302
GB41296	uncharacterized protein LOC100578542 precursor	48.665	0.146	-0.359	1.752	0.217
GB54239	zinc finger protein 853 isoform X6	48.402	0.300	-0.069	-0.006	0.046
100577273	uncharacterized protein LOC100577273	48.191	0.128	0.748	0.151	-0.234
GB44163	transmembrane and TPR repeat-containing protein CG4341-like isoform X1	47.994	0.189	0.092	0.152	0.180
GB41760	lipase 3-like	47.861	0.199	0.492	0.368	0.325

(continued)

Gene	Name	k	am__fc	bt__fc	lf__fc	ln__fc
GB48086	uncharacterized protein LOC551512	47.617	0.145	0.010	0.068	0.042
GB49149	neurogenic locus Notch protein isoform X3	47.488	-0.222	0.214	-0.001	-0.120
GB41115	Kv channel-interacting protein 1-like isoform X5	47.397	-1.062	0.183	0.352	-0.409
102655819	extensin-like	47.279	-0.451	-0.916	0.948	-0.287
GB44043	uncharacterized protein LOC724216	47.147	0.517	-1.371	0.693	-0.533
GB44829	bifunctional purine biosynthesis protein PURH-like isoform X2	46.740	-0.001	-0.050	0.403	0.086
GB48446	interferon regulatory factor 2-binding protein-like B-like	46.686	-0.067	0.052	0.202	-0.914
GB44170	intraflagellar transport protein 43 homolog isoform X4	45.900	0.117	0.018	0.544	-0.171
GB55834	uncharacterized protein LOC726282	45.592	0.136	0.448	0.758	-0.076
GB41182	proton-associated sugar transporter A-like isoform 1	45.270	0.466	-0.009	0.062	-0.275
GB50113	mitogen-activated protein kinase kinase kinase 15-like isoform X3	43.841	0.609	-0.068	0.169	0.229
102656841	WD repeat-containing protein WRAP73-like	43.107	-0.408	0.039	-0.529	0.054
GB50094	MMS19 nucleotide excision repair protein homolog	42.874	-0.070	-0.203	0.224	-0.322
102655306	uncharacterized protein LOC102655306	41.899	-0.836	0.435	0.405	-0.012
GB46663	uncharacterized protein LOC410375 isoform X2	41.853	0.185	-0.053	0.026	0.066
GB50402	uncharacterized protein LOC412801	41.663	-0.306	-0.285	0.506	-0.387
GB46296	slit homolog 1 protein-like	41.112	-0.034	0.004	-0.165	0.042
GB48694	probable inactive protein kinase DDB__G0270444-like isoform X2	41.007	-0.987	0.199	0.281	0.280
GB44577	solute carrier family 35 member G1-like	40.877	-0.300	-0.002	0.884	NA
GB47635	suppressor of variegation 3-9 isoform X2	40.782	0.147	0.066	0.267	0.035
GB47199	bifunctional methylenetetrahydrofolate dehydrogenase/cyclohydrolase, mitochondrial isoform X2	40.668	-0.041	0.028	0.173	-0.228
GB49794	uncharacterized protein LOC100577530 isoform X3	39.723	-0.221	0.091	0.255	-0.068
GB54817	muscle-specific protein 20	39.531	0.505	-0.102	0.343	0.089
GB47579	vesicular glutamate transporter 3 isoform X2	39.006	-0.079	-0.261	0.105	-0.094
GB52517	oxysterol-binding protein-related protein 1-like isoformX1	38.947	-0.065	0.033	0.306	0.010
GB54765	cytochrome P450 18a1	35.960	-1.059	0.059	0.043	-0.577
GB44783	putative GTP cyclohydrolase 1 type 2 Nif3l1-like	35.900	0.331	0.018	0.014	1.081
GB50763	arrestin domain-containing protein 2	35.767	-0.129	0.040	0.155	-0.756
GB19642	troponin C type IIa	35.629	0.425	-0.599	0.286	0.169
GB43860	protein ELYS-like isoform X3	35.552	-0.352	0.063	0.499	-0.115
GB51013	T-related protein-like isoform X2	35.369	0.266	-0.305	0.323	-0.706
GB45344	probable phosphatase phospho2-like	35.315	0.554	-0.015	0.199	-0.120
GB13325	chemosensory protein 6 precursor	34.928	0.839	-0.008	0.184	0.769
GB49079	estradiol 17-beta-dehydrogenase 8-like	33.906	0.577	-0.085	-0.422	0.010
GB50845	uncharacterized protein LOC725891 isoform X5	33.631	1.165	-0.377	0.431	0.274
GB46437	histone chaperone asf1	33.555	-0.251	0.210	0.204	0.078
GB46286	zinc carboxypeptidase A 1-like isoform X1	33.386	0.464	0.388	0.454	-0.231
GB50062	sulfotransferase 1C4-like	33.349	-0.498	-0.736	0.121	1.202
GB45076	transcription factor Sox-21-B-like	32.981	-1.436	0.875	0.697	-0.556
GB48028	circadian locomoter output cycles protein kaput	32.814	0.100	-0.121	-0.251	0.125
GB42300	uncharacterized protein LOC100577920	32.667	-0.628	0.052	0.290	0.246
GB48270	uncharacterized protein LOC100577045 isoform X1	31.620	0.630	-0.040	0.335	0.178
GB44913	fringe glycosyltransferase isoform X1	31.448	0.697	0.033	0.126	0.236
GB49541	zinc finger SWIM domain-containing protein 8-like isoform X3	31.361	-0.086	0.148	-0.369	0.082
GB47805	peptidoglycan-recognition protein S2 isoform X1	30.439	0.513	0.010	-0.054	0.383
GB41695	transcription factor Sox-10-like isoform X1	30.159	-0.162	-0.205	0.088	-0.483
GB51292	homeobox protein H90	29.960	-1.572	0.261	-0.224	0.048
GB52040	popeye domain-containing protein 3-like	28.477	-0.650	-0.279	0.268	0.039
GB53025	sister chromatid cohesion protein DCC1-like	24.610	-0.255	0.281	-0.145	0.335
102655272	NHP2-like protein 1-like	23.818	0.763	0.149	-1.619	0.068

Supplementary Table 27: List of all the genes in Module 2, ranked by their within-module connectivity, *k*. The latter four columns give the Log_2 fold-change in expression in response to queen pheromone in each of the four species.

Gene	Name	k	am_fc	bt_fc	lf_fc	ln_fc
GB43105	casein kinase II subunit alpha isoform X6	82.780	0.390	0.079	0.009	0.017
GB40946	serine/threonine-protein phosphatase 2A 65 kDa regulatory subunit A alpha isoform-like isoform X1	80.539	0.409	0.072	0.045	0.018
GB45257	ubiquitin-conjugating enzyme E2 L3-like isoform 2	80.094	0.362	-0.030	-0.006	0.127
GB53180	importin subunit alpha-3	79.075	0.568	0.036	-0.021	-0.023
GB53723	cytoplasmic tRNA 2-thiolation protein 1-like	78.715	0.051	0.045	0.029	0.039
GB43750	prefoldin subunit 5-like	77.479	0.556	0.115	0.000	0.034
GB43742	thioredoxin 1-like 1 isoform 1	77.174	0.421	-0.021	0.247	-0.069
GB51414	mRNA export factor-like	76.223	0.405	0.089	-0.108	0.058
GB40429	ras-related protein Rab-11A isoform X1	75.955	0.247	-0.061	-0.309	0.056
GB46322	electron transfer flavoprotein-ubiquinone oxidoreductase, mitochondrial isoform X2	74.439	0.364	-0.133	-0.135	0.123
GB45181	probable Bax inhibitor 1	74.401	0.447	-0.067	-0.167	-0.271
GB53311	neuralized-like protein 2-like	72.979	0.756	-0.023	0.579	0.014
GB55232	3-hydroxyacyl-CoA dehydrogenase type-2-like	72.680	0.677	0.035	-0.121	0.216
GB45127	transmembrane 9 superfamily member 4-like isoform 1	72.448	0.232	-0.081	-0.106	-0.004
GB51710	eukaryotic initiation factor 4A-like isoformX2	72.194	0.331	-0.006	0.260	0.307
GB50369	ankyrin repeat and FYVE domain-containing protein 1-like isoform X3	71.980	0.271	0.036	-0.705	0.047
GB56034	nuclear migration protein nudC-like	71.171	0.501	0.067	0.023	0.077
GB45752	ubiquitin-conjugating enzyme E2 N	69.697	0.424	0.142	0.002	0.539
GB49192	ribosomal RNA small subunit methyltransferase NEP1-like	69.454	0.310	0.014	-0.100	0.000
GB55434	rab GDP dissociation inhibitor beta	68.379	0.353	0.051	-0.164	-0.319
GB50080	calmodulin-like protein 4-like	68.211	0.636	0.085	-0.099	-0.055
GB48487	xylosyltransferase oxt	68.192	0.310	-0.014	-0.043	0.014
552579	proteasome subunit beta type-3-like	67.351	0.419	-0.072	-0.104	0.032
GB44693	complement component 1 Q subcomponent-binding protein, mitochondrial-like	67.275	0.063	0.118	-0.028	0.088
GB51009	T-complex protein 1 subunit delta-like isoform 1	66.898	0.649	0.022	0.073	-0.058
GB44312	hydroxyacylglutathione hydrolase, mitochondrial-like isoform X2	66.474	0.428	-0.035	-0.045	0.053
GB48369	lysM and putative peptidoglycan-binding domain-containing protein 1-like isoform X2	66.443	0.361	-0.093	-0.008	0.109
GB53382	cytosolic Fe-S cluster assembly factor NUBP1 homolog isoform X1	66.353	0.456	-0.062	-0.018	0.043
GB47478	glutathione peroxidase-like 1	65.945	0.203	-0.012	-0.260	-0.373
GB51333	coatomer subunit gamma isoform X3	65.732	0.460	0.098	-0.074	-0.028
GB50731	ragulator complex protein LAMTOR1-like	65.449	0.539	0.108	0.816	-0.041
GB51065	40S ribosomal protein S10-like isoform 1	65.323	0.601	0.063	-0.229	-0.009
GB45285	eukaryotic translation initiation factor 3 subunit F-like	65.166	0.584	0.035	0.043	-0.035
GB53349	proteasome subunit alpha type-3	64.905	0.309	-0.074	-0.190	-0.182
GB49597	eukaryotic translation initiation factor 4B-like	64.753	0.418	0.040	-0.073	0.101
GB55568	short-chain specific acyl-CoA dehydrogenase, mitochondrial isoform X1	64.412	0.490	-0.035	0.007	-0.100
GB55572	S-phase kinase-associated protein 1 isoform 1	64.297	0.150	0.010	-0.326	0.040
GB45047	proteasome subunit beta type-7-like	64.215	0.378	-0.068	0.402	-0.077
GB45354	V-type proton ATPase subunit d isoform X1	63.727	0.332	-0.032	0.022	-0.003
GB44576	ester hydrolase C11orf54 homolog	63.638	0.189	-0.001	0.281	0.278
GB48072	eukaryotic translation initiation factor 3 subunit M	63.521	0.307	0.089	0.021	0.095
GB50598	aldose reductase-like isoform 1	63.496	0.540	0.224	-0.038	-0.202
GB51072	40S ribosomal protein S4-like isoform 1	62.458	0.669	0.076	-0.156	-0.144
GB48916	charged multivesicular body protein 6-like	62.203	0.254	0.043	-0.115	-0.047
GB48669	neutral and basic amino acid transport protein rBAT isoform X3	62.108	0.233	-0.029	0.073	0.086
GB41358	elongation factor 1-alpha	61.731	0.815	0.070	-0.210	NA
GB51497	DNA-directed RNA polymerase II subunit RPB11	61.629	0.252	-0.042	0.014	0.100
GB41664	group XIIA secretory phospholipase A2-like isoform X1	61.328	0.266	-0.030	0.056	0.100
GB44755	eukaryotic translation initiation factor 4E type 3-A-like	61.145	0.104	0.124	0.037	-0.672
GB55625	NEDD4 family-interacting protein 1-like	60.731	0.565	-0.013	-0.004	0.009
GB43147	proteasome subunit beta type-2-like isoform 1	60.665	0.308	-0.135	-0.046	0.059

(continued)

Gene	Name	k	am_fc	bt_fc	lf_fc	ln_fc
GB40653	60S ribosomal protein L24	60.472	0.538	0.043	-0.180	0.199
GB54784	ATP-dependent RNA helicase DDX42-like isoform X2	60.316	0.241	0.085	-0.025	0.013
GB47655	protein AAR2 homolog	60.035	0.331	0.176	-0.019	-0.066
GB55420	programmed cell death protein 6-like isoform 3	59.941	0.583	-0.082	0.020	0.821
GB43852	ubiquitin-like protein 4A-like isoform 2	59.611	0.305	0.039	-0.024	0.114
GB40539	40S ribosomal protein S20	59.557	0.518	0.072	-0.128	-0.045
GB55639	40S ribosomal protein S3	58.751	0.666	0.014	0.136	-0.081
GB50057	dihydroorotate dehydrogenase (quinone), mitochondrial-like	58.648	0.193	-0.053	0.111	0.151
GB41363	26S protease regulatory subunit 6B isoform 1	58.459	0.541	-0.014	-0.027	0.020
GB47998	AP-2 complex subunit mu isoform 1	58.347	0.330	-0.034	-0.005	-0.514
GB42560	14-3-3 protein epsilon isoform X2	58.339	0.504	0.037	0.170	-0.078
GB53626	myotrophin-like isoform 2	58.009	0.507	0.161	-0.060	0.138
GB54379	cleavage stimulation factor subunit 1-like	57.944	0.596	0.189	0.029	0.011
GB44704	flavin reductase (NADPH)-like	57.917	0.211	0.087	0.024	-0.013
GB46420	eukaryotic translation initiation factor 3 subunit I isoform X1	57.072	0.563	0.127	-0.057	0.032
GB45018	prolactin regulatory element-binding protein-like	56.995	0.339	0.027	-0.019	-0.072
GB45880	ubiquinone biosynthesis protein COQ4 homolog, mitochondrial-like isoform X3	56.947	0.226	-0.065	0.014	0.013
GB50333	40S ribosomal protein S6-like	56.908	0.380	0.292	-0.017	0.047
410306	ADP-ribosylation factor 2-like	56.597	0.360	0.012	-0.069	-0.012
GB54211	protein DJ-1-like	56.345	0.314	0.001	0.770	0.017
GB40576	60S acidic ribosomal protein P0 isoform X2	56.091	0.742	0.104	-0.019	0.119
GB41211	ATP-binding cassette sub-family E member 1	55.950	0.787	0.100	-0.059	-0.008
GB40414	protein SMG8-like	55.864	0.131	0.026	0.327	-0.016
GB48810	60S ribosomal protein L8	55.820	0.537	0.086	0.059	0.089
GB18750	T-cell immunomodulatory protein isoform X1	55.698	0.409	0.015	-0.039	-0.096
GB42679	40S ribosomal protein S8	55.513	0.299	0.073	-0.149	0.107
GB52728	prohibitin-2-like	55.365	0.262	0.097	0.047	0.032
GB54797	brahma-associated protein of 60 kDa-like isoform X2	55.360	0.361	0.025	-0.138	0.043
GB46540	cytochrome b5-like isoform X2	55.297	0.456	0.126	-0.141	0.071
GB45171	SUMO-conjugating enzyme UBC9 isoform X2	55.127	0.578	-0.007	-0.095	0.074
GB47103	elongation factor 1-beta'	55.076	0.310	0.095	-0.023	0.262
GB52116	60S ribosomal protein L36 isoform X2	54.840	0.561	0.063	0.003	-0.040
GB42537	40S ribosomal protein S15	54.821	0.734	0.068	0.000	0.034
GB54747	26S proteasome non-ATPase regulatory subunit 11-like	54.723	0.383	-0.031	-0.132	-0.019
GB54973	selT-like protein-like isoform 1	54.677	0.693	-0.082	0.029	-0.023
GB43392	guanine deaminase-like	54.429	0.260	-0.056	0.238	-0.619
GB54814	60S ribosomal protein L31 isoform 1	54.370	0.721	0.082	-0.070	-0.152
GB40718	thioredoxin reductase 1 isoform X1	54.111	0.398	-0.092	0.191	0.014
GB46888	alpha-methylacyl-CoA racemase-like	53.923	0.439	-0.183	-0.612	-0.112
GB55901	ribosome biogenesis protein NSA2 homolog isoform X1	53.877	0.488	-0.004	-0.042	-0.079
GB53402	calcineurin B homologous protein 1	53.642	0.378	-0.153	-0.148	-0.118
GB45856	protein GPR107-like isoform X4	53.485	0.401	-0.050	-0.178	0.016
GB53849	segment polarity protein dishevelled homolog DVL-3 isoform X2	53.479	0.499	0.099	0.001	0.815
GB47925	coatomer subunit beta	53.403	0.552	0.111	-0.067	0.332
GB52694	proteasome subunit beta type-4-like	53.270	0.581	-0.064	-0.112	-0.014
GB49377	40S ribosomal protein S3a	53.268	0.501	0.027	0.285	0.002
GB44997	palmitoyltransferase ZDHHC5-like isoform X1	52.977	0.105	0.161	0.013	0.038
GB50652	T-complex protein 1 subunit epsilon	52.915	0.773	0.122	-0.487	0.017
GB53360	V-type proton ATPase subunit D 1-like isoform 1	52.783	0.192	-0.021	0.035	0.613
GB51033	E3 ubiquitin-protein ligase parkin-like isoform 1	52.691	0.271	-0.037	-0.036	0.019
GB51683	annexin-B9-like isoform X1	52.638	0.544	-0.038	0.069	-0.033
GB50158	60S ribosomal protein L4 isoform 1	52.600	0.748	0.103	0.167	0.113
GB55011	eukaryotic translation initiation factor 3 subunit K-like	52.505	0.352	0.033	-0.060	0.027
GB44749	60S ribosomal protein L9	52.408	0.618	0.058	-0.193	0.171
GB42036	protein SEC13 homolog isoform X2	52.388	0.485	0.051	0.018	0.857
GB45261	mannose-1-phosphate guanyltransferase beta-like	52.325	0.496	0.060	0.006	-0.007
GB47880	superoxide dismutase 1	52.265	0.467	-0.346	-0.019	-0.324
GB40341	uncharacterized protein LOC413618	52.060	0.173	0.083	-0.088	0.038
GB54183	DNA-directed RNA polymerases I, II, and III subunit RPABC2-like	51.823	0.240	0.046	-0.144	0.138

(continued)

Gene	Name	k	am_fc	bt_fc	lf_fc	ln_fc
GB44160	protein dpy-30 homolog	51.691	0.633	0.120	-0.179	-0.047
GB45369	receptor of activated protein kinase C 1, transcript variant X3	51.675	0.606	0.099	0.010	-0.174
GB50870	39S ribosomal protein L41, mitochondrial	51.531	0.088	0.021	-0.040	0.051
GB43559	60S ribosomal protein L3	51.530	0.601	0.114	0.002	0.140
GB50519	transmembrane emp24 domain-containing protein eca-like	51.317	0.710	0.016	0.386	-0.862
GB48536	T-complex protein 1 subunit beta-like isoform 1	51.251	0.746	0.097	0.028	-0.021
GB51889	vesicle-trafficking protein SEC22b-B-like isoform X1	51.132	0.430	-0.044	-0.072	0.066
GB53974	probable RNA helicase armi	51.116	0.142	0.244	0.511	0.104
GB54854	proteasome maturation protein-like	51.069	0.523	-0.080	-0.048	-0.008
GB50455	ubiquitin-conjugating enzyme E2-17 kDa-like	51.034	0.676	0.052	0.053	0.293
GB53799	proteasome subunit alpha type-2	50.980	0.636	-0.096	-0.148	-0.018
GB42152	puromycin-sensitive aminopeptidase isoform X3	50.955	0.157	0.044	-0.029	-0.088
GB42810	uncharacterized protein C7orf26 homolog	50.806	0.420	-0.029	-0.054	0.029
GB50917	60S acidic ribosomal protein P1	50.680	0.627	0.068	0.033	0.142
GB43466	retinol dehydrogenase 11-like	50.634	0.327	-0.044	-0.036	0.118
GB51973	methionine aminopeptidase 1-like	50.381	0.262	-0.038	0.082	0.084
GB54779	syntenin-1-like isoform X1	50.290	0.407	-0.032	0.016	-0.057
GB47638	ER membrane protein complex subunit 3-like	50.176	0.705	-0.010	-0.047	0.048
GB46750	40S ribosomal protein S16	50.099	0.620	0.090	0.551	-0.424
GB45737	target of rapamycin complex subunit lst8-like	50.032	0.402	0.118	0.038	0.199
GB52512	60S ribosomal protein L28	50.020	0.714	0.064	0.396	-0.049
GB48150	actin-related protein 2/3 complex subunit 1A	49.843	0.525	0.038	-0.046	-0.050
GB55748	exosome complex component RRP40	49.785	0.322	0.168	-0.128	0.047
GB53219	40S ribosomal protein S17	49.705	0.742	0.097	0.116	-0.250
409728	40S ribosomal protein S5 isoform X1	49.450	0.917	0.069	0.174	0.120
GB51201	40S ribosomal protein S12 isoform X1	49.398	0.724	0.067	-0.012	0.028
GB44039	malate dehydrogenase, cytoplasmic-like isoform 1	49.342	0.513	-0.139	-0.007	0.175
GB55077	ras suppressor protein 1 isoform X2	49.164	0.280	-0.109	0.062	0.091
GB46562	40S ribosomal protein S24-like isoform X2	49.143	0.321	0.031	0.110	0.051
410017	protein OPI10 homolog	49.121	0.564	0.074	-0.268	0.011
GB46776	40S ribosomal protein S11 isoform X1	49.040	0.624	0.088	-0.058	-0.245
GB48309	uncharacterized protein LOC552534	48.953	0.201	0.030	0.046	0.050
GB51038	60S ribosomal protein L23	48.907	0.739	0.081	-0.185	-0.027
GB40232	peroxiredoxin 1	48.841	0.790	0.104	0.467	0.275
GB41604	chloride intracellular channel exc-4	48.700	0.223	-0.021	0.229	0.257
GB50356	60S acidic ribosomal protein P2	48.671	0.837	0.100	-0.028	-0.052
GB53321	transmembrane protein adipocyte-associated 1 homolog isoform X1	48.633	0.102	-0.026	0.057	1.013
GB50303	unc-112-related protein-like isoform 1	48.557	0.015	-0.028	-0.032	-0.020
GB48630	JNK1/MAPK8-associated membrane protein-like	48.447	0.187	0.118	-0.052	0.050
GB54184	bridging integrator 3 homolog	48.280	0.247	0.042	0.090	0.089
GB47881	signal peptidase complex catalytic subunit SEC11A	48.133	0.526	0.073	-0.070	0.641
GB51264	glutamine-dependent NAD(+) synthetase, transcript variant X3	48.095	0.014	-0.053	0.000	-0.049
GB41631	60S ribosomal protein L34 isoform X2	48.041	0.518	0.106	-0.169	-0.198
GB52789	60S ribosomal protein L22 isoform 1	48.019	0.507	-0.009	0.013	0.425
GB41198	CDGSH iron-sulfur domain-containing protein 2 homolog isoform X1	47.967	0.308	-0.067	-0.012	0.045
GB52256	60S ribosomal protein L5	47.585	0.370	0.048	-0.007	0.017
GB47689	deoxyhypusine hydroxylase-like	47.480	0.236	0.022	0.967	0.202
GB44646	uncharacterized protein LOC100577295	47.302	0.330	0.179	0.091	0.027
GB51031	uncharacterized protein LOC727650 isoform X1	47.280	0.505	0.114	0.095	-0.066
GB55528	26S proteasome non-ATPase regulatory subunit 6-like	47.152	0.346	-0.055	-0.210	0.055
GB55891	diphosphoinositol polyphosphate phosphohydrolase 1	47.113	0.424	0.030	0.047	0.025
GB54243	LOW QUALITY PROTEIN: carbonyl reductase [NADPH] 1-like	46.880	0.819	-0.092	0.512	0.055
GB52946	CCR4-NOT transcription complex subunit 7-like isoform X2	46.803	0.434	0.132	0.049	-0.007
GB48172	GDP-mannose 4,6 dehydratase-like isoform X3	46.741	0.311	0.167	-0.232	-0.116
GB44147	60S ribosomal protein L15	46.714	0.498	0.050	0.243	-0.020
GB49750	leucine-rich repeat neuronal protein 1-like isoform X2	46.627	0.697	0.193	-0.183	-0.055
GB40882	40S ribosomal protein S13 isoform X1	46.598	0.445	0.107	-0.056	-0.049

(continued)

Gene	Name	k	am_fc	bt_fc	lf_fc	ln_fc
GB44803	glutathione S-transferase omega-1 isoform X1	46.505	0.720	0.134	-0.021	0.115
GB54165	signal recognition particle subunit SRP68	46.495	0.313	0.022	-0.124	-0.201
GB49364	splicing factor U2af 38 kDa subunit	46.473	0.505	0.018	0.626	0.110
GB51359	60S ribosomal protein L27a isoform X1	46.464	0.710	-0.002	-0.191	-0.209
GB46774	dnaJ protein homolog 1-like	46.338	0.148	0.121	-0.047	-0.104
GB51727	activator of 90 kDa heat shock protein ATPase homolog 1-like isoform 2	46.274	0.437	0.096	-0.036	0.013
GB48886	protein YIF1B-like	46.028	0.561	-0.197	0.654	0.023
GB47030	serine/threonine-protein phosphatase 6 catalytic subunit isoform 1	45.990	0.434	0.076	-0.050	0.142
GB44927	DNA excision repair protein haywire isoform X2	45.980	0.152	0.042	0.021	0.138
GB48261	116 kDa U5 small nuclear ribonucleoprotein component-like isoform 1	45.925	0.282	0.165	0.797	-0.055
GB53247	transmembrane emp24 domain-containing protein-like	45.868	0.541	-0.041	-0.070	-0.074
GB41362	H/ACA ribonucleoprotein complex subunit 1-like	45.854	0.408	0.177	-0.127	-0.094
GB40395	transmembrane emp24 domain-containing protein bai	45.718	0.378	0.004	-0.001	-0.091
GB44520	uncharacterized protein LOC552106 isoform X1	45.566	0.560	0.110	0.249	-0.078
GB48750	F-box-like/WD repeat-containing protein ebi isoform 1	45.555	0.567	0.174	-0.046	0.149
GB50513	dihydropteridine reductase isoform X1	45.520	0.470	-0.081	-0.139	-0.048
GB44905	serine/threonine-protein kinase mTOR	45.440	0.140	-0.080	0.036	-0.094
GB54343	10 kDa heat shock protein, mitochondrial-like isoform X1	45.414	1.367	0.306	-0.139	-0.103
GB54131	uncharacterized protein LOC726184	45.096	0.258	-0.032	0.006	-0.190
GB55288	O-acetyl-ADP-ribose deacetylase MACROD2-like isoform X3	45.010	-0.070	0.050	-0.015	0.067
GB40513	GPI transamidase component PIG-S-like isoform X1	44.900	0.252	-0.003	0.057	-0.022
GB41525	3-ketodihydrosphingosine reductase-like isoform 1	44.782	0.332	-0.121	-0.085	-0.019
GB46375	ubiquitin-conjugating enzyme E2 variant 2-like isoform 1	44.762	0.537	0.116	0.084	0.044
GB45624	diphthine synthase	44.599	0.666	0.169	-0.119	0.146
GB43379	membrane-bound transcription factor site-2 protease-like	44.340	0.609	-0.093	-0.035	0.198
GB47590	40S ribosomal protein S7	44.263	0.527	0.016	-0.009	0.081
GB44841	methylthioribose-1-phosphate isomerase-like isoform X4	44.168	0.641	0.223	-0.193	0.031
GB46462	6-phosphogluconolactonase-like	44.117	0.465	0.037	-0.084	-0.823
GB44870	zinc finger protein 706-like isoform X3	43.891	0.355	0.108	0.045	0.068
GB43115	U6 snRNA-associated Sm-like protein LSm7 isoform X3	43.467	0.478	-0.014	-0.048	-0.176
GB43999	peroxiredoxin-5, mitochondrial	43.349	0.073	-0.338	-0.367	-0.276
GB42809	translationally-controlled tumor protein homolog isoform 1	43.010	0.406	0.070	0.090	0.063
GB45374	40S ribosomal protein S23-like	42.728	0.576	0.161	-0.027	0.280
GB49552	venom protease	42.579	0.168	1.465	-0.022	-0.032
GB50228	uncharacterized protein LOC726353 isoform X1	42.577	0.121	-0.128	0.379	-0.082
GB44678	hairless	42.238	0.292	0.107	0.056	-0.009
GB44936	histone-arginine methyltransferase CARMER-like isoform 1	42.203	0.390	-0.044	0.005	0.285
GB53070	phosphatidylcholine:ceramide cholinephosphotransferase 1-like isoform X1	42.186	0.253	0.000	0.047	0.173
GB42705	protein archease-like	42.064	1.184	0.032	-0.022	0.078
GB50929	mitochondrial import receptor subunit TOM40 homolog 1-like isoform 1	42.006	0.601	0.112	-0.255	-0.034
GB43634	congested-like trachea protein-like isoform X2	41.916	0.400	0.072	-0.072	0.116
GB45690	phosphoribosylformylglycinamidine synthase	41.774	0.316	-0.028	-0.057	0.047
GB50873	60S ribosomal protein L30 isoform 1	41.747	0.540	0.132	0.138	0.557
GB42516	Rab escort protein	41.739	0.277	0.222	0.037	0.021
GB42189	15 kDa selenoprotein-like isoform X1	41.640	0.322	0.009	-0.023	0.120
GB48215	endoplasmic reticulum resident protein 44 isoform X2	41.598	0.229	0.049	-0.119	0.104
GB55220	L-xylulose reductase	41.366	0.481	0.188	-0.028	0.030
GB40073	COP9 signalosome complex subunit 8-like	41.235	0.373	0.047	0.095	-0.166
GB55989	AN1-type zinc finger protein 2B-like isoform X1	41.195	0.281	-0.158	-0.315	0.174
GB42780	CCHC-type zinc finger protein CG3800-like isoform X3	40.990	0.409	0.039	-0.012	0.525
GB44206	death-associated protein 1-like	40.988	0.519	-0.118	-0.167	0.189
GB43844	ARL14 effector protein-like	40.833	0.597	0.004	0.218	0.057
GB44735	2-hydroxyacylsphingosine 1-beta-galactosyltransferase-like isoform X2	40.833	0.220	0.073	-0.048	0.172
GB49312	glyoxylate reductase/hydroxypyruvate reductase-like	40.827	0.350	-0.023	-0.032	-0.085
GB49628	cytosolic non-specific dipeptidase-like isoform 1	40.778	0.528	0.064	-0.110	-0.071
GB40866	heat shock protein cognate 4	40.767	0.496	0.076	-0.369	-0.432

(continued)

Gene	Name	k	am_fc	bt_fc	lf_fc	ln_fc
GB42829	juvenile hormone epoxide hydrolase 1	40.757	0.491	-0.003	-0.042	-0.120
GB55186	B(0,+)-type amino acid transporter 1-like	40.583	0.453	0.074	0.028	0.083
GB53027	mediator of RNA polymerase II transcription subunit 4	40.497	0.848	0.089	0.089	0.032
GB46334	uncharacterized protein LOC724516	40.373	0.535	0.079	0.059	-0.032
GB51188	lysophospholipid acyltransferase 2-like	40.363	0.843	-0.138	-0.183	-0.098
GB47514	dynactin subunit 6	40.146	0.505	-0.103	0.060	0.014
GB52102	40S ribosomal protein S26	40.132	0.398	0.007	0.111	-0.023
726117	triosephosphate isomerase	40.091	0.140	-0.145	0.141	-0.178
GB47553	electron transfer flavoprotein subunit alpha, mitochondrial-like isoform 1	39.917	0.650	0.029	-0.095	0.473
GB41522	methyl-CpG-binding domain protein 2 isoformX3	39.873	0.559	0.144	-0.132	-0.020
GB54263	integrator complex subunit 6-like isoform X2	39.854	0.109	0.222	-0.054	-0.054
GB54166	uridine 5'-monophosphate synthase isoform X1	39.790	0.536	0.008	-0.113	-0.012
GB51062	exostosin-1 isoform X2	39.767	0.670	-0.165	-0.067	-0.007
GB50981	enoyl-CoA delta isomerase 2, mitochondrial-like	39.665	0.485	0.014	-0.145	0.104
GB53138	inorganic pyrophosphatase-like	39.495	0.437	0.080	0.629	0.072
GB51533	pleckstrin homology domain-containing family F member 2-like isoform 2	39.482	0.338	0.004	0.015	-0.067
GB55053	soluble calcium-activated nucleotidase 1-like	39.369	0.668	-0.106	-0.074	0.052
GB55081	TM2 domain-containing protein CG11103-like	39.261	0.269	0.156	-0.053	0.003
GB54693	fumarylacetoacetate hydrolase domain-containing protein 2A-like isoformX2	39.260	0.467	-0.034	-0.061	-0.141
GB52500	rRNA 2'-O-methyltransferase fibrillarin	39.250	0.705	0.302	0.075	-0.054
GB47469	rRNA-processing protein FCF1 homolog	39.226	0.378	0.077	-0.174	0.006
GB40887	V-type proton ATPase subunit E isoform 3	39.170	0.266	-0.054	-0.219	0.063
GB50637	SUZ domain-containing protein 1-like isoform X2	39.158	0.337	0.153	-0.032	0.120
413799	mitochondrial import inner membrane translocase subunit Tim17-A isoform 2	39.088	0.628	-0.012	0.095	0.024
GB40208	WD40 repeat-containing protein SMU1-like isoform 1	38.990	0.349	-0.076	-0.002	0.082
GB41342	DNA replication licensing factor Mcm7	38.907	0.522	0.066	-0.205	0.103
GB54192	60S ribosomal protein L13 isoform 1	38.870	0.396	0.058	0.028	0.185
GB49159	probable nuclear transport factor 2-like isoform 3	38.578	0.717	0.187	-0.075	0.031
GB47441	V-type proton ATPase 21 kDa proteolipid subunit-like	38.388	0.549	0.030	-0.016	0.001
GB47399	muscle segmentation homeobox-like isoform X1	38.281	0.773	-0.256	-0.493	-0.118
GB52698	synaptobrevin-like isoformX1	38.200	0.605	0.070	-0.006	0.766
GB41598	brahma associated protein 55kd	38.136	0.522	0.182	-0.087	0.077
GB51590	protein MEF2BNB homolog	37.971	0.035	0.573	-0.110	-0.019
GB50299	tubulin–tyrosine ligase-like protein 12-like	37.864	0.439	0.020	0.020	0.093
GB42649	putative deoxyribonuclease TATDN1-like isoform X1	37.768	0.614	-0.133	-0.001	-0.046
GB55013	NTF2-related export protein isoform X5	37.738	0.296	0.072	-0.124	0.291
GB52120	peroxiredoxin-6	37.724	0.382	-0.055	-0.015	0.109
412837	ribonuclease H2 subunit A-like	37.638	0.614	0.211	0.023	-0.034
GB48905	glutathione S-transferase S1	37.553	0.615	0.074	0.044	0.173
GB45435	LOW QUALITY PROTEIN: probable tRNA(His) guanylyltransferase-like	37.547	0.528	0.030	0.055	-0.094
GB43228	uncharacterized protein LOC408327 isoform X1	37.498	0.357	0.067	-0.189	0.089
GB53737	zinc finger protein Xfin-like	37.344	0.634	-0.010	-0.077	0.111
GB51043	arginase-1-like isoform X2	37.282	0.195	-0.082	-0.072	-0.293
GB51543	60S ribosomal protein L13a isoform 2	37.211	0.594	0.036	0.356	-0.133
GB45012	adenosylhomocysteinase-like	37.172	0.583	0.156	-0.009	0.248
GB43697	mediator of RNA polymerase II transcription subunit 16 isoform X3	37.167	0.457	0.056	0.995	-0.098
GB47617	peptidyl-prolyl cis-trans isomerase-like	37.130	0.569	0.057	0.018	0.544
GB45147	clavesin-2-like	36.835	1.275	-0.339	0.021	-0.106
GB42239	senecionine N-oxygenase-like isoform X3	36.751	0.916	0.297	-0.213	-0.068
GB45640	adenosine monophosphate-protein transferase FICD homolog isoform 1	36.668	0.507	0.129	-0.205	0.102
GB48811	ATP-dependent RNA helicase bel	36.570	0.378	0.046	-0.138	0.057
GB49264	protein phosphatase methylesterase 1-like isoform 1	36.437	0.272	-0.028	0.122	0.008
GB50928	39S ribosomal protein L14, mitochondrial	36.373	0.554	-0.020	0.002	0.111
GB45433	small ribonucleoprotein particle protein B	36.335	0.638	0.239	0.058	-0.118
GB42039	ribosomal RNA processing protein 36 homolog	36.173	0.178	-0.014	1.315	0.173

(continued)

Gene	Name	k	am_fc	bt_fc	lf_fc	ln_fc
GB55282	von Willebrand factor A domain-containing protein 9-like	36.035	0.139	0.067	-0.076	0.158
GB50304	zinc transporter 7-like	35.996	0.658	0.082	-0.104	-0.066
GB49129	uncharacterized protein LOC408761 isoform 2	35.770	0.211	0.110	-0.541	-0.014
GB47973	integrin-linked protein kinase-like	35.679	0.376	0.001	0.019	-0.035
GB52033	uncharacterized protein LOC100578121 isoform X2	35.583	0.354	-0.401	0.383	-0.172
GB46059	THO complex subunit 3-like	35.530	0.319	0.072	-0.012	-0.078
GB53712	putative phospholipase B-like lamina ancestor-like isoform X1	35.415	0.617	-0.124	-0.033	-0.122
GB50198	serine/threonine-protein kinase grp isoform X3	35.256	0.428	0.100	-0.168	-0.078
726972	uncharacterized protein LOC726972 isoform X2	35.211	0.648	-0.144	-0.091	-0.019
GB44292	cyclin-dependent kinase 14-like isoform X3	35.055	0.271	0.071	-0.069	-0.023
GB48364	inactive hydroxysteroid dehydrogenase-like protein 1-like isoform X3	35.037	0.674	-0.012	-0.042	-0.028
GB53628	serine/threonine-protein kinase STK11 isoform X1	34.892	1.226	0.182	0.281	0.047
GB44130	nucleolar protein 9-like	34.810	0.067	-0.037	-0.017	0.051
GB52433	BET1 homolog isoform X2	34.796	0.505	-0.104	0.085	0.182
GB45490	regulator of chromosome condensation 1, transcript variant X9	34.692	0.290	0.240	0.096	0.019
724802	protein Asterix-like	34.625	0.996	-0.077	-0.092	0.010
GB51698	hexamerin 70a precursor	34.611	4.071	0.179	-0.218	-0.256
GB45527	TM2 domain-containing protein almondex	34.595	0.426	0.044	0.127	0.027
GB55515	inositol oxygenase-like	34.544	0.418	-0.069	-0.137	-0.252
GB53823	3-oxoacyl-[acyl-carrier-protein] synthase, mitochondrial-like	34.399	0.724	-0.022	0.005	0.075
GB50265	glutathione S-transferase D1 isoform X4	34.396	0.539	-0.084	0.637	0.013
GB51977	PEST proteolytic signal-containing nuclear protein-like	34.370	0.250	0.132	0.067	0.192
GB43709	cytochrome P450 9e2-like isoform X3	34.366	0.031	-0.082	0.040	-0.127
412189	phosphopantothenoylcysteine decarboxylase-like isoform X2	34.310	0.218	-0.024	0.050	-0.101
GB44133	tubulin beta-1 chain	34.216	0.143	0.158	-0.110	-0.107
GB40596	ubiquitin-like protein 7-like isoform X2	34.192	0.424	0.071	-0.148	-0.041
GB46844	uracil phosphoribosyltransferase homolog isoformX2	34.091	0.503	0.166	-0.030	0.066
GB50902	glyceraldehyde-3-phosphate dehydrogenase 2 isoform 1	34.081	0.170	-0.056	0.004	-0.001
GB53341	sortilin-related receptor	34.026	0.166	0.067	-0.091	-0.150
GB41142	probable dolichyl pyrophosphate Glc1Man9GlcNAc2 alpha-1,3-glucosyltransferase-like isoform X2	34.022	0.712	0.004	0.046	0.138
GB43471	alpha/beta hydrolase domain-containing protein 17B-like isoformX1	34.019	0.298	0.094	-0.149	0.129
GB51283	retinal dehydrogenase 1-like isoformX1	34.006	0.428	0.229	0.066	-0.046
GB40976	heat shock protein 90	34.000	0.284	0.127	-0.504	0.037
GB52648	Golgi SNAP receptor complex member 2	33.919	0.297	0.144	-0.118	0.043
GB52893	protein sel-1 homolog 1-like	33.913	0.116	0.081	-0.203	-0.054
GB53540	AP-2 complex subunit alpha isoformX1	33.837	1.221	0.067	-0.031	0.029
GB44804	uncharacterized protein LOC100578631 isoform X1	33.816	0.389	0.178	0.014	-0.003
GB53656	mitochondrial ubiquitin ligase activator of nfkb 1-like	33.731	0.362	0.146	0.357	0.277
GB42244	uncharacterized protein LOC100576169	33.594	0.226	-0.134	-0.076	-0.118
102654594	WD repeat-containing protein 18-like	33.593	0.852	-0.016	-0.275	-0.066
GB48111	proteasome subunit beta type-1	33.589	0.552	-0.096	-0.039	0.382
GB42747	la-related protein 4-like isoform X2	33.487	0.297	-0.072	-0.311	-0.064
GB53500	transcriptional regulator Myc-B-like	33.404	0.523	0.268	-0.297	0.053
GB53427	nicotinic acetylcholine receptor alpha9 subunit precursor	33.353	0.755	-0.006	0.004	0.225
GB44960	actin-related protein 2/3 complex subunit 4	33.333	0.533	0.098	-0.065	0.022
GB46167	polycomb group RING finger protein 3-like	33.309	0.957	0.101	0.060	0.414
GB51656	zinc finger HIT domain-containing protein 3-like	33.267	0.404	-0.095	-0.306	0.082
GB55139	nucleoside diphosphate kinase	33.260	0.392	-0.041	-0.031	-0.038
GB44571	PQ-loop repeat-containing protein 1-like	33.255	0.369	0.104	0.201	0.075
GB49649	2-oxoisovalerate dehydrogenase subunit beta, mitochondrial-like	33.238	0.660	-0.024	-0.039	0.236
GB55529	PRA1 family protein 3-like isoform 1	33.229	0.235	0.009	0.119	0.070
GB41150	40S ribosomal protein S2 isoform 2	33.225	0.628	-0.090	0.066	-0.111
GB41465	N-alpha-acetyltransferase 15, NatA auxiliary subunit-like isoform 1	32.994	0.279	-0.070	-0.433	0.599
725416	U1 small nuclear ribonucleoprotein C	32.957	0.447	0.318	0.122	-0.218
GB48225	ras-related protein M-Ras-like isoform X1	32.671	0.335	-0.053	0.078	0.019

(continued)

Gene	Name	k	am_fc	bt_fc	lf_fc	ln_fc
GB53674	peflin-like isoform X1	32.435	0.396	-0.033	0.127	0.161
GB52780	retinal rod rhodopsin-sensitive cGMP 3',5'-cyclic phosphodiesterase subunit delta isoform X1	32.260	0.724	0.085	-0.038	0.258
GB53440	mitochondrial enolase superfamily member 1-like	32.152	0.178	-0.132	1.540	0.108
GB55532	dnaJ homolog subfamily C member 2-like isoform X1	32.068	0.241	0.170	0.019	-0.041
GB45871	protein halfway-like isoform X2	32.064	0.342	-0.034	-0.856	0.009
GB42465	DTW domain-containing protein 2-like isoform 1	31.931	0.622	0.238	-0.247	0.107
GB43302	SPARC isoformX2	31.879	0.262	0.026	0.150	0.352
GB51257	eukaryotic translation initiation factor 5B	31.875	0.104	0.073	-0.037	-0.007
102656905	nuclear receptor-binding factor 2-like	31.762	0.109	-0.080	0.147	0.038
GB54865	TATA-box-binding protein-like	31.750	0.866	-0.017	-0.098	-0.082
GB42252	armadillo repeat-containing protein 6 homolog isoform X1	31.703	1.271	0.185	-0.137	-0.068
GB50100	copper homeostasis protein cutC homolog	31.532	1.025	0.018	0.029	0.234
GB48435	phosphotriesterase-related protein-like isoform X2	31.411	0.546	0.145	0.075	0.222
GB52251	multifunctional protein ADE2, transcript variant X2	31.396	1.088	0.025	0.016	-0.169
GB46916	cyclin-related protein FAM58A-like isoform 1	31.382	0.423	0.111	-0.176	0.118
GB41827	ATP-binding cassette sub-family G member 5-like isoform X1	31.285	0.453	-0.143	-0.065	0.010
GB40362	flap endonuclease 1	31.277	0.830	0.045	-0.132	0.031
102656183	histidine triad nucleotide-binding protein 1-like	31.237	0.569	0.039	0.236	-0.055
GB48638	autophagy-specific gene 6 isoform X1	31.232	0.568	-0.027	0.054	0.058
GB40259	sel1 repeat-containing protein 1 homolog isoform 2	31.212	0.138	-0.294	0.056	0.071
GB50603	ribose-5-phosphate isomerase	31.170	0.829	-0.134	0.024	0.042
GB54634	uncharacterized protein LOC725260 isoform X1	31.137	0.878	-0.066	-0.370	-0.097
GB41841	glucoside xylosyltransferase 1-like	30.811	0.177	0.287	-0.109	-0.313
GB44918	transcription initiation factor TFIID subunit 5 isoform X1	30.760	0.425	0.142	0.038	0.065
GB44731	tafazzin homolog isoformX2	30.738	0.411	0.011	-0.119	-0.216
GB41806	calcyphosin-like protein-like isoform X1	30.707	0.585	-0.066	0.078	-0.254
GB51079	probable UDP-glucose 4-epimerase-like	30.650	0.558	0.307	0.170	0.033
GB46254	NAD-dependent protein deacetylase Sirt4 isoform 2	30.410	0.127	-0.170	0.123	0.101
GB53419	nicotinamidase-like isoform X3	30.372	0.089	-0.154	-0.141	-0.221
GB50527	putative aminopeptidase W07G4.4-like	30.181	0.436	-0.036	0.099	-0.195
GB46544	DNA-directed RNA polymerase II subunit RPB7-like	30.055	0.110	0.169	0.078	0.141
GB49521	NAD(P)H-hydrate epimerase-like isoform 1	29.929	0.574	-0.008	0.021	0.127
GB53965	uncharacterized protein LOC100578606 isoform X5	29.893	0.398	0.033	0.018	0.066
GB40831	serine protease gd isoform X2	29.878	0.051	0.093	-0.104	-0.170
GB49839	sorting nexin-8-like isoform X2	29.787	0.206	0.065	0.106	-0.294
GB51600	6-pyruvoyl tetrahydrobiopterin synthase-like	29.669	0.305	0.290	-1.933	0.058
GB46347	uncharacterized protein LOC100577486	29.665	0.662	0.031	-0.066	-0.249
GB55816	GTP-binding protein Rhes-like	29.643	0.499	0.234	0.003	0.211
GB48765	uncharacterized protein LOC410179	29.570	0.477	0.166	0.329	0.034
GB49808	uncharacterized protein LOC100578801	29.568	0.727	0.235	-0.019	0.072
GB54174	E3 ubiquitin-protein ligase RING1 isoform 1	29.511	0.643	0.044	0.788	-0.069
GB41901	protein phosphatase PTC7 homolog	29.428	0.209	0.083	0.094	0.030
GB51964	F-box/LRR-repeat protein 4 isoform X1	29.415	0.193	0.125	0.152	0.164
GB51088	isovaleryl-CoA dehydrogenase, mitochondrial-like	29.408	0.224	0.148	-0.070	-0.124
GB48566	BTB/POZ domain-containing protein 2-like isoform X2	29.307	0.661	0.014	-0.099	0.081
GB53925	uncharacterized protein LOC724993	29.104	0.509	-0.022	0.014	-0.059
GB48983	RING finger protein 121-like isoform X3	28.981	0.541	0.016	-0.188	0.055
GB50427	nucleoporin NDC1-like	28.974	0.684	0.395	-0.082	-0.114
GB50087	solute carrier family 25 member 44-like isoform X1	28.889	0.106	-0.114	-0.034	-0.062
GB48250	putative gamma-glutamylcyclotransferase CG2811-like isoform X4	28.883	0.436	-0.020	-0.031	0.114
GB46249	non-structural maintenance of chromosomes element 1 homolog isoform X1	28.852	0.209	-0.089	0.018	0.130
GB55496	pyruvate dehydrogenase E1 component subunit beta, mitochondrial	28.837	0.011	-0.165	-0.096	0.009
GB54611	antithrombin-III	28.818	0.756	-0.338	-0.366	0.745
GB45316	tetratricopeptide repeat protein 8-like isoformX1	28.810	0.731	-0.427	0.206	0.031
GB55984	protein RCC2 homolog	28.339	0.313	0.353	-0.009	-0.259
GB51210	lateral signaling target protein 2 homolog	28.270	0.003	-0.067	-0.088	-0.076
GB42924	zinc transporter ZIP1-like	28.158	0.422	0.002	-0.100	-0.090

(continued)

Gene	Name	k	am_fc	bt_fc	lf_fc	ln_fc
GB49080	nose resistant to fluoxetine protein 6-like isoform X1	27.955	0.797	-0.248	0.647	-0.259
GB45850	clavesin-2 isoform X1	27.918	0.346	0.179	-0.040	0.057
GB40906	myb-like protein P-like isoform X2	27.822	0.254	-0.374	-0.237	-0.055
GB54485	UDP-glucuronosyltransferase 1-3-like	27.810	0.228	0.479	0.104	0.076
GB43256	ATP-binding cassette sub-family D member 1-like	27.776	0.792	0.011	0.095	-0.146
GB44109	peptidylglycine alpha-hydroxylating monooxygenase	27.772	0.297	0.114	0.042	-0.050
GB49013	RNA-binding protein 8A	27.750	0.752	-0.025	1.331	0.035
GB47955	S-adenosylmethionine synthase-like isoform X1	27.740	0.015	0.070	0.049	-0.826
GB44798	uncharacterized protein LOC410725 isoform X2	27.687	0.144	-0.071	0.471	-0.029
GB46017	uncharacterized protein C4orf29 homolog isoform X3	27.642	0.510	0.138	0.060	0.028
726289	transcription factor AP-1	27.305	0.449	-0.031	0.169	-0.054
GB49331	leucine-rich repeat neuronal protein 1-like	27.177	0.717	0.119	0.125	-0.197
GB44871	glycine N-methyltransferase-like	27.121	0.157	-0.172	0.347	-0.341
GB43816	probable tubulin polyglutamylase TTLL1-like isoform X1	27.090	0.625	-0.213	0.221	0.176
726860	cytochrome b5-like isoform 1	27.055	0.778	-0.094	0.044	0.096
GB41823	pachytene checkpoint protein 2 homolog	26.979	-0.004	0.215	-0.214	0.272
GB45700	serine protease easter	26.878	0.145	-0.226	-0.013	-0.060
411552	ceramide glucosyltransferase	26.819	0.187	-0.084	-0.142	-0.143
GB54511	probable ATP-dependent RNA helicase DDX17-like	26.806	0.171	0.029	-0.404	-0.018
GB50678	2-oxoglutarate and iron-dependent oxygenase domain-containing protein 1-like isoform X1	26.787	0.554	0.177	0.016	-0.006
GB46297	cuticular protein 14 precursor	26.754	0.478	-0.510	0.042	-0.386
GB54541	leukocyte elastase inhibitor-like isoform X1	26.716	0.304	-0.419	-0.107	-0.020
GB40490	inhibitor of growth protein 5-like	26.561	0.494	0.079	0.010	0.078
GB49403	ragulator complex protein LAMTOR3-A-like	26.511	0.599	-0.099	0.096	0.123
GB40931	uncharacterized protein LOC409781 isoform X2	26.463	0.481	-0.131	0.154	-0.199
GB44344	uncharacterized protein LOC100576497 isoform X1	26.403	0.722	-0.157	0.477	0.044
GB55973	dentin sialophosphoprotein-like isoform X1	26.350	0.404	-0.567	0.238	-0.146
GB48335	40S ribosomal protein S19a-like	26.256	1.931	0.114	0.139	-0.030
GB51095	cryptochrome 2 isoform X4	26.234	0.038	-0.001	-0.359	0.177
GB50867	cell differentiation protein RCD1 homolog isoform X2	26.207	0.794	0.206	-0.022	0.097
GB41224	mediator of RNA polymerase II transcription subunit 18 isoform X1	26.167	0.764	0.032	-0.007	0.042
GB40157	uncharacterized protein LOC408421 isoformX2	26.082	0.237	-0.120	0.223	-0.211
GB46767	UPF0553 protein C9orf64 homolog	26.007	0.596	0.128	-0.114	-0.268
GB44559	probable small nuclear ribonucleoprotein Sm D1-like	25.975	0.806	0.067	0.062	0.073
GB50824	protein trapped in endoderm-1-like isoform X3	25.861	1.344	0.011	-0.018	0.068
GB50677	lipoma HMGIC fusion partner-like 2 protein-like isoform X2	25.830	0.335	0.250	-0.206	-0.228
GB46215	ras-related protein Rab-24-like isoform X2	25.811	0.359	0.077	0.108	0.263
GB46684	monocarboxylate transporter 3-like	25.621	0.190	-0.104	-0.037	0.067
GB48452	protein Smaug homolog 1-like	25.585	0.069	0.183	-0.068	0.142
GB47833	transmembrane protein 165-like	25.502	0.424	0.251	0.713	0.126
GB41720	uncharacterized protein LOC727121 isoform X1	25.224	0.265	0.164	0.027	-0.142
GB48884	egl nine homolog 1-like	25.153	0.287	0.164	0.105	0.057
GB55629	capa receptor-like GPCR	25.008	0.653	0.237	0.075	-0.072
GB44868	uncharacterized protein LOC409307	24.919	0.430	0.389	-0.205	0.125
GB48419	carbohydrate sulfotransferase 11-like isoform X1	24.862	0.092	-0.116	-0.005	-0.204
GB49026	ataxin-2 homolog isoform X4	24.857	0.190	0.024	-0.097	0.051
GB41222	G-protein coupled receptor Mth2-like	24.830	0.402	0.013	-0.018	0.133
GB51611	latrophilin Cirl-like isoform X9	24.677	0.223	-0.084	0.124	-0.071
GB50893	insulin-like growth factor-binding protein complex acid labile subunit-like	24.613	0.092	0.111	0.072	-0.233
GB44424	lipoma HMGIC fusion partner-like 3 protein-like isoform X2	24.500	0.469	-0.409	0.014	-0.068
GB52511	probable methyltransferase BTM2 homolog	24.493	0.286	-0.152	-0.130	0.268
GB47043	succinate dehydrogenase [ubiquinone] flavoprotein subunit, mitochondrial isoform X2	24.477	0.131	0.001	1.062	0.299
GB55096	NADP-dependent malic enzyme isoform X3	24.441	1.483	-0.139	0.040	-0.067
GB50509	nimrod C2 isoform X3	24.441	0.414	0.075	-0.092	-0.247
GB50218	ornithine aminotransferase, mitochondrial	24.438	1.000	0.193	0.148	0.114
GB47823	CD81 antigen isoform X1	24.195	0.183	0.151	0.260	-0.196
GB45950	organic cation transporter protein-like isoform X5	24.118	0.385	-0.266	-0.064	-1.051
GB56027	prothrombin	24.075	0.644	-0.038	0.200	-0.022

(continued)

Gene	Name	k	am__fc	bt__fc	lf__fc	ln__fc
GB52258	peptidyl-prolyl cis-trans isomerase-like 3-like	23.962	0.483	-0.115	0.131	0.101
GB41159	bifunctional dihydrofolate reductase-thymidylate synthase	23.960	0.665	0.168	0.163	-0.204
GB41897	GTP-binding protein 128up-like isoform 1	23.748	0.431	0.208	-0.111	-0.052
GB53381	cytochrome c oxidase assembly protein COX11, mitochondrial-like isoformX2	23.639	0.277	-0.081	0.010	0.072
GB41881	U3 small nucleolar ribonucleoprotein protein IMP4-like	23.437	0.278	-0.040	-0.291	-0.179
GB46762	cyclin-dependent kinases regulatory subunit-like	23.410	0.699	0.303	-0.081	0.146
GB55944	trafficking protein particle complex subunit 6B-like	23.291	0.195	0.080	0.889	0.196
GB43738	phenoloxidase subunit A3	23.274	0.818	-0.002	-0.111	0.126
GB40085	carboxypeptidase B-like	23.163	-0.088	0.550	-0.470	-0.671
GB10293	aubergine	22.990	0.360	0.234	3.125	0.022
GB50891	solute carrier organic anion transporter family member 5A1-like	22.951	0.021	-0.035	0.048	-0.140
GB53136	chromatin assembly factor 1 subunit B	22.892	0.449	0.176	-0.008	0.257
GB45250	uncharacterized protein LOC409595	22.836	0.162	-0.134	4.811	0.017
726409	peptidyl-prolyl cis-trans isomerase H-like	22.720	0.324	0.205	0.148	0.186
GB40778	UDP-galactose translocator	22.705	0.335	-0.104	0.048	-0.013
GB41376	J domain-containing protein-like isoform 2	22.701	0.280	-0.391	-0.169	-0.772
GB46832	lactosylceramide 4-alpha-galactosyltransferase-like isoform X2	22.686	1.237	0.279	0.074	-0.110
726804	protein BTG2-like	22.535	0.201	0.004	0.025	0.055
GB44994	coiled-coil-helix-coiled-coil-helix domain-containing protein 10, mitochondrial-like	22.519	0.284	0.071	0.132	0.018
GB48474	probable chitinase 3-like	22.510	0.317	-1.089	-0.137	-0.297
GB41912	trans-1,2-dihydrobenzene-1,2-diol dehydrogenase-like	22.461	2.084	0.440	-0.213	-0.286
GB50226	transferrin 1 precursor	22.313	1.234	-0.434	-0.194	-0.141
GB41670	sialin-like isoform X2	22.226	0.454	0.087	0.066	-0.232
GB53750	UPF0454 protein C12orf49 homolog isoform X2	22.213	0.682	-0.146	0.255	0.021
GB42038	WD repeat-containing protein 92-like	22.041	0.333	-0.043	-0.152	0.328
GB42614	LIX1-like protein-like isoformX2	21.905	0.426	0.226	0.115	0.063
GB48251	[Pyruvate dehydrogenase (acetyl-transferring)] kinase, mitochondrial isoform X4	21.796	0.287	-0.017	0.094	0.092
GB54097	protein Malvolio isoform X3	21.778	0.912	-0.631	-0.002	0.059
GB55576	uncharacterized LOC408661, transcript variant X2	21.274	0.392	0.058	-0.481	-0.081
GB50819	microtubule-associated protein futsch-like isoform X2	21.207	0.170	0.234	0.020	-0.001
GB50239	short/branched chain specific acyl-CoA dehydrogenase, mitochondrial-like	21.158	0.795	-0.005	-0.284	0.003
GB42822	chitooligosaccharidolytic beta-N-acetylglucosaminidase-like	20.977	0.098	0.179	0.157	-0.122
GB42306	ATP-dependent RNA helicase vasa	20.808	0.629	0.309	-0.044	0.031
GB41083	ras-related protein Rab-23 isoformX2	20.362	0.142	0.117	0.013	0.123
GB55388	arylsulfatase J-like	20.361	0.422	0.132	0.013	0.245
GB49919	MATH and LRR domain-containing protein PFE0570w-like isoform X2	20.271	0.652	0.299	0.530	0.197
GB55082	protein PBDC1-like	20.152	0.726	0.279	0.003	0.489
GB44079	menin-like	19.990	0.633	0.134	0.206	-0.046
GB46686	uncharacterized protein LOC411065 isoform X2	19.985	0.048	0.067	-0.157	-0.151
GB53956	uncharacterized protein C17orf104-like	19.933	0.259	0.135	-0.553	0.163
GB55615	cell wall protein IFF6-like	19.754	0.595	-0.244	0.170	-0.378
GB43789	tubulin polyglutamylase TTLL4-like isoform X6	19.630	0.240	0.165	-0.085	-0.020
GB43963	tyrosine kinase receptor Cad96Ca-like	19.602	0.139	0.046	-0.109	0.353
GB44064	uncharacterized protein LOC725703	19.596	0.175	-0.498	0.652	-0.303
GB50661	inosine triphosphate pyrophosphatase-like isoform X3	19.565	0.609	0.182	0.856	-0.082
GB46206	LOW QUALITY PROTEIN: origin recognition complex subunit 2-like	19.550	0.066	0.055	-0.149	0.303
GB42190	protein arginine N-methyltransferase 5 isoform X2	19.040	0.206	0.172	0.088	-0.102
GB40837	alpha-tocopherol transfer protein-like isoform X1	19.037	2.049	0.211	0.314	0.097
GB53835	zinc finger protein 704-like isoform X4	19.026	0.149	-0.073	0.488	-0.133
GB43202	uncharacterized protein LOC725540 isoform X2	18.995	0.491	-0.006	0.223	0.428
GB48790	monocarboxylate transporter 9-like isoform X4	18.821	0.014	-0.031	0.002	0.004
GB54735	uncharacterized protein LOC100578100	18.449	0.616	0.069	-0.354	-0.479
GB40523	uncharacterized protein C20orf112 homolog isoform X3	18.401	0.356	-0.278	0.012	-0.335
GB55628	probable RNA-binding protein EIF1AD-like isoform X1	18.257	0.603	0.321	0.105	0.082

(continued)

Gene	Name	k	am_fc	bt_fc	lf_fc	ln_fc
GB55203	yellow-e3 precursor	18.121	0.520	0.400	-0.036	0.011
GB43581	uncharacterized protein LOC100577641	18.077	0.469	0.007	0.178	-0.109
GB48936	facilitated trehalose transporter Tret1-1-like	17.946	-0.055	0.021	0.900	-0.195
102654371	pro-resilin-like	17.868	1.464	1.004	0.301	0.014
GB42981	beta-1,3-glucan-binding protein	17.585	0.226	-0.265	-0.327	-0.195
GB45015	SHC SH2 domain-binding protein 1 homolog B-like isoform X1	17.428	0.204	0.107	-0.298	0.048
GB48937	facilitated trehalose transporter Tret1-like	17.413	0.395	-0.016	0.027	-0.314
GB46635	phytanoyl-CoA dioxygenase domain-containing protein 1 homolog	17.402	1.184	0.015	0.142	0.168
GB48300	twin protein isoform X1	17.309	0.825	0.249	-0.206	0.030
GB42318	uncharacterized protein LOC727116	16.896	0.266	0.212	0.082	0.020
GB46589	SAGA-associated factor 29 homolog	16.792	0.286	0.157	-0.403	0.018
GB51200	DNA-binding protein D-ETS-6-like	16.707	1.482	1.211	0.176	0.127
GB51697	hexamerin 70b precursor	16.651	0.925	1.592	-0.143	-0.325
GB50693	neuropeptide Y-like	16.565	0.636	0.188	0.122	0.068
GB41669	baculoviral IAP repeat-containing protein 5 isoform X2	16.330	0.405	0.186	-0.036	-0.001
GB51075	putative odorant receptor 13a-like	16.297	1.962	0.638	-0.579	0.247
551223	probable cytochrome P450 305a1	16.282	0.788	0.155	-0.461	0.452
GB43786	calsyntenin-1-like	16.096	0.382	-0.089	0.200	-0.435
GB41207	26S proteasome non-ATPase regulatory subunit 14	15.814	0.549	-0.017	-0.599	0.035
GB43778	enhancer of split mgamma protein-like	15.716	0.475	0.113	0.190	-0.102
GB54885	transcriptional activator cubitus interruptus isoform X1	15.600	0.249	-0.258	0.259	-0.121
GB49755	uncharacterized protein LOC410867	15.314	0.258	0.016	0.192	0.004
102654312	KRR1 small subunit processome component homolog	15.079	0.304	0.177	-0.310	-0.057
GB42865	solute carrier organic anion transporter family member 5A1-like isoform X2	15.007	0.350	-0.281	0.025	-0.162
GB51494	phosphoenolpyruvate carboxykinase [GTP] isoformX1	14.954	0.427	0.066	-0.182	-0.019
GB55423	major facilitator superfamily domain-containing protein 12-like	14.832	0.067	0.155	0.112	0.308
GB48999	helix-loop-helix protein 11	14.691	1.203	0.190	0.044	0.440
GB43508	inositol polyphosphate 1-phosphatase-like isoform X2	14.582	0.900	-0.173	0.179	-0.007
GB54549	alpha-glucosidase precursor	13.838	-0.103	0.368	0.091	-0.649
GB55482	Na(+)/H(+) exchange regulatory cofactor NHE-RF1-like isoform X2	13.271	0.544	0.085	-0.543	-0.413
GB51528	DUOXA-like protein C06E1.3-like	12.347	0.364	-0.176	0.211	-0.358
GB55729	major royal jelly protein 1	12.059	1.266	0.054	0.053	0.086
GB41230	TWiK family of potassium channels protein 18-like isoform X1	11.965	0.291	-0.243	0.880	0.337
GB50469	pituitary homeobox homolog Ptx1-like isoform X5	11.637	0.686	-0.265	-0.132	0.021
725344	histone H2B-like	11.545	0.829	0.288	0.055	0.382
GB49170	40S ribosomal protein S15Aa-like isoform 1	10.727	0.758	0.049	0.219	-0.048
GB42794	circadian clock-controlled protein-like isoform 1	10.677	0.340	-0.157	-0.043	-0.034
GB42580	short-chain dehydrogenase/reductase family 9C member 7-like	10.166	0.488	-0.371	-0.021	-0.470
GB41967	uncharacterized protein LOC100576746 isoform X1	9.972	1.432	0.977	0.379	NA
GB50477	uncharacterized protein LOC100577527	9.690	1.621	-0.020	-0.011	-0.080
GB45499	sodium-coupled monocarboxylate transporter 2-like isoform X1	6.618	0.351	-0.315	0.410	0.402

Supplementary Table 28: List of all the genes in Module 3, ranked by their within-module connectivity, k. The latter four columns give the Log_2 fold-change in expression in response to queen pheromone in each of the four species.

Gene	Name	k	am_fc	bt_fc	lf_fc	ln_fc
GB40980	glucose transporter type 1 isoform X7	39.155	0.052	-0.091	1.216	0.083
GB53562	uncharacterized protein LOC408874 isoform X9	38.524	-0.005	-0.077	0.541	-0.211
GB42840	leukocyte receptor cluster member 8 homolog isoform X4	37.431	-0.455	-0.108	0.387	0.056
GB42142	nuclear hormone receptor FTZ-F1 isoform X2	36.815	-0.202	0.041	0.170	-0.321
GB54291	YTH domain-containing protein 1-like	36.717	-0.244	0.041	0.302	0.074
GB41401	RUN and FYVE domain-containing protein 2-like isoform X4	36.491	-0.081	-0.037	0.261	0.132
GB44662	A-kinase anchor protein 10, mitochondrial-like isoform X1	36.432	-0.241	0.006	0.250	0.058
GB45265	uncharacterized protein LOC409634 isoform X2	36.140	-0.408	-0.063	0.260	0.148
GB43636	transmembrane and TPR repeat-containing protein CG4050-like isoform 1	35.659	0.185	0.147	0.677	0.190
GB52643	poly(U)-specific endoribonuclease homolog	35.336	0.006	0.067	0.355	-0.326
GB46539	protein vav-like isoform X3	35.217	-0.066	-0.191	0.164	-0.090
GB53721	receptor-type tyrosine-protein phosphatase N2-like isoform X2	34.717	-0.185	0.096	0.283	0.200
GB52588	conserved oligomeric Golgi complex subunit 7	34.710	-0.051	-0.047	-0.037	0.082
GB50853	myotubularin-related protein 13 isoform X4	33.869	0.051	0.033	0.151	0.071
GB41811	filaggrin-like isoform X3	33.098	-0.534	0.032	0.211	0.073
GB43218	microtubule organizer protein 1-like isoform X4	33.029	-0.577	0.051	0.130	0.210
GB46243	zinc finger Ran-binding domain-containing protein 2-like isoform X2	32.773	-0.522	-0.029	0.260	0.179
GB52247	serine/arginine repetitive matrix protein 1-like isoform X2	32.447	-0.486	0.050	0.062	-0.081
GB42651	mitochondrial intermediate peptidase-like	32.432	-0.394	-0.065	0.066	0.180
GB51503	mitogen-activated protein kinase 1	32.148	-0.015	0.128	0.204	0.180
GB46102	vesicle-fusing ATPase 1-like	31.644	0.302	-0.073	0.215	0.274
GB45702	ubiquinone biosynthesis monooxygenase COQ6-like isoform X2	31.609	-0.258	-0.028	0.192	0.248
GB45145	MAGUK p55 subfamily member 6 isoform X4	31.521	-0.098	-0.144	0.176	0.126
GB45555	putative RNA-binding protein 15B isoform X1	31.345	-0.402	0.013	0.114	-0.070
GB47779	uncharacterized protein LOC410467	31.131	0.005	-0.010	0.193	0.127
GB48526	protein fem-1 homolog CG6966-like isoform X1	30.915	-0.058	0.133	0.067	0.155
GB54715	protein kintoun-like isoform X1	30.634	-0.274	0.070	0.119	0.070
GB55514	LOW QUALITY PROTEIN: protein suppressor of white apricot	30.563	-0.357	0.136	0.091	0.026
GB53944	exocyst complex component 1	30.154	-0.467	0.116	0.156	0.147
GB51596	neuroblastoma-amplified sequence	29.978	-0.652	-0.047	-1.039	0.224
GB44483	PRKC apoptosis WT1 regulator protein-like isoform X4	29.863	0.099	0.032	0.181	0.197
411347	peripheral plasma membrane protein CASK-like isoform X12	29.847	-0.214	0.158	0.279	-0.315
GB55146	uncharacterized protein LOC409323	29.786	0.273	-0.140	0.515	0.086
GB44208	WD repeat-containing protein 37-like isoform X4	29.592	-0.017	-0.004	0.083	0.407
GB53700	solute carrier family 12 member 8-like	29.551	0.073	0.032	0.169	0.043
GB49488	O-phosphoseryl-tRNA(Sec) selenium transferase-like isoform X3	28.734	-0.247	0.235	0.272	0.167
GB50679	UPF0430 protein CG31712-like isoform X3	28.597	-0.604	-0.035	0.214	0.119
GB55507	FYVE, RhoGEF and PH domain-containing protein 4-like isoform X2	28.573	0.146	0.129	0.167	0.067
GB55285	uncharacterized protein LOC724761	28.419	-0.232	-0.191	0.283	0.248
GB41146	synembryn isoform X3	28.266	0.066	0.010	0.143	0.100
GB41079	guanine nucleotide-binding protein subunit beta-5 isoform X2	28.079	-0.015	0.066	0.176	0.050
GB49896	kinesin 2A	27.926	0.138	0.095	0.234	0.090
GB51002	fasciculation and elongation protein zeta-2 isoform X4	27.833	0.175	0.099	0.415	0.135
GB44031	dorsal protein isoform B	27.827	-0.126	-0.097	0.223	-0.121
GB44721	cleavage and polyadenylation specificity factor subunit 5-like isoform 2	27.583	0.054	0.068	0.089	0.278
GB55350	amyloid beta A4 precursor protein-binding family B member 2-like isoform X7	27.524	-0.003	-0.201	0.302	0.360
GB47963	probable E3 ubiquitin-protein ligase HERC4-like isoform X3	27.279	-0.107	0.009	0.302	0.158
GB44077	kxDL motif-containing protein CG10681-like	27.274	-0.081	0.181	0.148	0.137
102654127	neurochondrin homolog	27.164	-0.423	-0.157	0.427	0.070
GB44296	JNK-interacting protein 1 isoform X3	26.898	-0.112	-0.154	0.543	0.233

(continued)

Gene	Name	k	am_fc	bt_fc	lf_fc	ln_fc
GB50144	galactoside 2-alpha-L-fucosyltransferase 2-like	26.798	-0.695	0.064	0.427	0.244
GB45227	transmembrane protein 64-like isoform 1	26.751	-0.198	-0.063	0.179	0.159
GB45535	phosphoribosyl pyrophosphate synthase-associated protein 2-like isoform X1	26.578	0.065	-0.033	0.201	0.183
GB54201	uncharacterized protein LOC100577050 isoform X4	26.193	-0.263	0.038	0.316	0.530
GB49397	insulin-like growth factor-binding protein complex acid labile subunit-like isoform X6	26.106	0.030	-0.296	0.304	-0.001
GB42675	adenylate cyclase type 2-like	25.972	-0.430	-0.116	0.394	0.090
GB52759	brefeldin A-inhibited guanine nucleotide-exchange protein 3-like isoform X2	25.955	-0.254	-0.040	-0.033	0.214
GB52464	uncharacterized protein LOC726793	25.802	0.283	0.097	0.066	0.257
GB55441	uncharacterized protein LOC409164 isoform X2	25.574	0.155	-0.046	0.194	0.031
GB42917	transmembrane protein 53-like	25.515	-0.009	0.201	0.312	0.096
GB45413	GTPase activating Rap/RanGAP domain-like 3 isoform X2	25.513	-0.675	0.081	0.185	0.004
GB55635	cAMP-responsive element-binding protein-like 2-like	25.490	-0.114	-0.012	0.133	0.043
GB46914	unzipped precursor	25.406	0.069	-0.208	0.324	0.081
GB40973	glutamate receptor 1-like isoform X2	25.189	0.034	-0.178	0.182	0.178
GB52600	uncharacterized protein LOC413002 isoform X2	25.018	-0.229	0.138	0.266	0.348
GB45036	breast cancer anti-estrogen resistance protein 3-like isoform X1	24.895	-0.474	-0.159	0.280	0.147
GB40305	NADH dehydrogenase [ubiquinone] iron-sulfur protein 2, mitochondrial isoform 1	24.824	-0.244	-0.193	0.147	0.040
GB54057	uncharacterized protein LOC551162 isoform X3	24.795	0.206	0.169	0.233	0.093
GB47039	dynamin isoform X11	24.655	-0.451	-0.126	0.201	0.129
GB55539	uncharacterized protein LOC100577742	24.584	-0.249	0.239	0.109	0.035
GB53582	serine/threonine-protein kinase tricorner isoform X6	24.419	-0.397	0.030	0.093	0.113
408663	soma ferritin	24.386	-0.379	0.735	0.345	0.094
GB45617	protein eyes shut isoform X1	24.338	-0.416	-0.103	0.381	0.080
GB40343	uncharacterized protein LOC413620 isoform X2	24.325	-0.201	0.033	0.080	-0.926
GB40356	spondin-1-like isoform X9	24.094	0.239	0.017	0.380	0.136
GB45140	junctophilin-1-like isoform X3	23.905	-0.318	0.055	0.312	0.270
GB44523	NACHT and WD repeat domain-containing protein 1-like isoform X2	23.892	-0.408	0.004	0.268	-0.066
GB49480	cGMP-specific 3',5'-cyclic phosphodiesterase-like isoform X4	23.856	0.255	-0.086	-0.095	0.276
GB48162	anoctamin-8-like isoformX1	23.848	-0.133	-0.246	0.283	0.360
GB51722	tetraspanin-1 isoformX1	23.814	0.341	0.157	0.299	0.175
GB40118	glutamate decarboxylase-like isoform X2	23.789	-0.164	-0.083	0.384	0.250
GB40358	RILP-like protein homolog isoform X2	23.608	-0.060	0.053	0.315	0.216
GB51489	proton-coupled amino acid transporter 1-like isoform X2	23.433	-0.220	-0.158	0.421	0.315
GB44746	uncharacterized protein LOC412112 isoform X1	23.351	0.182	-0.087	0.238	0.185
GB44482	solute carrier family 12 member 7-like isoform X5	23.302	-0.087	-0.166	-1.179	0.275
GB50415	diacylglycerol kinase theta-like isoform X7	23.214	0.066	-0.206	0.559	0.172
GB41746	neogenin isoform X2	23.159	-0.345	-0.137	0.141	0.037
GB52028	elongation factor 1-alpha	23.107	-0.132	0.112	0.424	0.039
GB43116	sarcoplasmic calcium-binding protein 1 isoformX2	23.066	0.408	-0.047	0.173	0.326
GB50516	TPPP family protein CG4893-like isoform X1	22.733	0.259	-0.002	0.217	0.125
GB43052	paramyosin, long form-like	22.730	-0.175	-0.173	0.088	0.109
GB45013	repressor of RNA polymerase III transcription MAF1 homolog	22.589	-0.047	-0.055	0.239	0.104
GB50911	probable G-protein coupled receptor CG31760-like isoform X5	22.578	-0.605	-0.349	0.277	0.268
GB43546	uncharacterized protein LOC410487	22.424	-0.469	0.053	0.112	0.060
GB51838	uncharacterized protein LOC552650	22.273	0.081	-0.411	0.185	0.227
GB48539	calcium-dependent protein kinase 4-like	22.182	-0.376	-0.042	0.164	-0.694
GB52723	uncharacterized protein LOC726322 isoform X2	22.154	-0.329	-0.086	0.313	0.228
GB51689	muscarinic acetylcholine receptor DM1-like	22.102	0.082	0.050	-0.172	0.214
GB40923	nicotinic acetylcholine receptor alpha8 subunit	22.070	0.505	-0.095	0.318	0.187
GB47352	zinc finger protein Noc-like	21.818	-0.249	0.041	0.359	0.097
GB49396	sodium- and chloride-dependent GABA transporter 1 isoform X2	21.814	-1.358	-0.165	0.819	0.034
GB44810	LOW QUALITY PROTEIN: carboxyl-terminal PDZ ligand of neuronal nitric oxide synthase protein-like	21.786	-0.317	0.061	0.311	0.160

(continued)

Gene	Name	k	am_fc	bt_fc	lf_fc	ln_fc
GB46705	muscle M-line assembly protein unc-89 isoform X5	21.660	-0.526	-0.175	0.213	-0.411
GB46673	two pore potassium channel protein sup-9-like isoform X1	21.626	-0.122	0.101	0.141	0.184
GB45263	prohormone-4	21.607	0.154	-0.032	0.362	0.377
GB48632	synaptotagmin 14, transcript variant X3	21.363	0.073	-0.307	0.416	0.027
GB52321	agglutinin-like protein 1-like isoform X4	21.241	-0.352	-0.082	0.356	0.228
GB53345	uncharacterized protein LOC100578770	21.092	-0.670	0.048	0.363	0.224
GB40701	uncharacterized protein LOC551765	21.056	-0.366	-0.189	0.155	0.062
GB50651	prohormone-3	21.047	0.137	-0.068	0.338	0.217
GB46720	regulator of G-protein signaling 17-like isoform X1	20.924	-0.082	0.368	0.280	0.133
GB53030	transmembrane protein 181-like isoform X2	20.919	0.317	0.041	0.389	0.326
GB44369	suppressor APC domain-containing protein 2-like isoform X2	20.761	0.051	0.154	0.156	0.132
GB40303	broad-complex core protein isoforms 1/2/3/4/5-like isoform X2	20.630	0.074	-0.069	0.396	0.179
GB49174	uncharacterized protein LOC412265 isoform X2	20.587	-0.932	0.106	0.191	-0.050
GB52953	guanylate cyclase, soluble, beta 1	20.444	0.607	0.151	0.169	0.206
GB45235	uncharacterized protein LOC724460	20.277	-0.284	0.909	0.258	0.313
GB40794	synaptotagmin-4 isoform 2	20.256	-0.609	-0.136	0.268	0.174
GB44213	filamin-like	20.215	-0.430	-0.054	0.426	-0.012
GB47203	uncharacterized protein LOC552612	20.155	0.107	0.024	0.210	-0.035
GB53340	spectrin beta chain isoform X1	20.116	0.274	-0.096	0.338	0.088
GB43814	potassium channel subfamily K member 18-like isoform X2	20.014	-0.572	-0.168	0.246	0.020
102656283	uncharacterized protein LOC102656283	19.767	-0.579	-0.058	0.821	-0.025
GB49268	glutamate receptor ionotropic, kainate 2-like isoform X1	19.756	-0.296	-0.082	0.386	0.222
GB42529	signal peptide peptidase-like 3-like isoform X2	19.664	0.132	-0.149	0.222	0.162
GB48187	mitogen-activated protein kinase kinase kinase 7 isoform X2	19.660	-0.044	0.109	0.347	-0.109
GB44988	prohormone-2 precursor	19.653	-0.055	-0.256	0.308	-0.768
GB46886	NMDA receptor 1 isoform X2	19.640	0.362	-0.221	0.361	0.277
GB40599	fukutin-related protein-like	19.589	-0.162	0.092	0.143	0.012
GB49732	uncharacterized protein R02F2.2-like isoform X2	19.589	-0.501	-0.198	0.199	0.020
GB42659	cAMP-dependent protein kinase type I regulatory subunit isoform X5	19.557	0.095	-0.188	0.699	0.098
GB43293	high-affinity choline transporter 1-like	19.555	0.446	-0.094	0.524	-0.236
GB53652	coenzyme Q-binding protein COQ10 homolog B, mitochondrial-like	19.533	-0.387	-0.207	0.094	0.086
GB45733	kinesin 13 isoform X2	19.513	-0.469	0.112	0.030	0.141
GB44536	uncharacterized protein LOC100576683	19.450	-0.581	0.063	0.229	0.131
GB54724	cationic amino acid transporter 4-like	19.417	0.311	0.136	0.270	-0.184
GB46165	fibroblast growth factor receptor homolog 1 isoform X2	19.399	-0.559	-0.327	0.336	0.182
GB48892	LOW QUALITY PROTEIN: synaptogyrin-2	19.361	-0.632	0.168	0.063	0.272
GB54818	muscle LIM protein Mlp84B-like isoform X2	19.346	-0.283	-0.065	0.239	0.202
GB48489	dipeptidyl aminopeptidase-like protein 6-like isoform X1	19.304	-0.078	-0.139	-0.928	0.119
GB55504	zinc finger protein 143-like isoform X2	19.176	-0.140	-0.034	0.077	0.093
GB52763	uncharacterized protein LOC100578776 isoform X2	19.168	-0.047	-0.013	0.485	0.190
GB41659	endothelin-converting enzyme 1 isoform X4	19.101	-0.277	0.036	0.268	0.246
GB55237	disco-interacting protein 2 isoform X1	19.094	0.189	0.090	1.408	-0.833
GB45393	surfeit locus protein 1	19.047	-0.301	-0.041	0.166	0.153
GB47678	protein FAM69C-like isoform X2	18.982	-0.122	-0.136	0.352	0.226
GB52679	uncharacterized protein LOC409139 isoform X2	18.976	0.148	0.101	0.136	0.189
GB41866	uncharacterized LOC552431, transcript variant X4	18.956	0.411	-0.013	0.264	0.300
725415	BTB/POZ domain-containing protein KCTD16-like	18.951	0.941	-0.651	0.296	0.346
GB47990	tropomyosin-1-like	18.938	0.150	-0.346	0.116	0.393
GB55520	PDZ domain-containing RING finger protein 4	18.829	-0.560	-0.027	0.327	0.044
GB43818	trimeric intracellular cation channel type B-like	18.770	-0.054	-0.453	0.300	0.386
GB49969	tubby-related protein 4-like isoform X3	18.752	-0.140	0.272	0.234	0.106
GB43231	uncharacterized protein LOC408329	18.748	-0.372	0.267	0.264	0.027
GB40625	synapsin isoform X2	18.745	-0.001	0.264	0.496	0.295
GB42692	ultraspiracle isoform X7	18.741	0.002	-0.002	0.227	-0.290
GB48362	protein kinase DC2 isoform X1	18.697	0.014	-0.086	0.448	0.196
GB54423	uncharacterized protein LOC551958	18.658	-0.018	-0.122	0.511	-0.108
GB50142	uncharacterized protein LOC726068	18.626	-0.183	0.296	0.221	0.057
GB55559	D-beta-hydroxybutyrate dehydrogenase, mitochondrial-like isoform X2	18.616	0.010	-0.388	0.347	0.456

(continued)

Gene	Name	k	am_fc	bt_fc	lf_fc	ln_fc
GB44430	dihydrolipoyllysine-residue succinyltransferase component of 2-oxoglutarate dehydrogenase complex, mitochondrial-like isoform X1	18.611	-0.593	-0.067	0.189	-0.248
GB54446	arginine kinase isoform X2	18.602	0.231	-0.129	0.441	0.141
GB52013	isocitrate dehydrogenase [NAD] subunit gamma 1, mitochondrial-like isoform 2	18.534	-0.603	-0.135	0.159	0.191
GB51787	myosin light chain alkali-like isoform X5	18.438	0.444	-0.279	0.372	0.350
GB47928	allatostatins precursor	18.421	-1.060	-0.088	0.304	0.282
GB40537	reticulon-4-interacting protein 1 homolog, mitochondrial-like	18.410	0.021	0.022	0.169	0.225
GB48163	probable NADH dehydrogenase [ubiquinone] iron-sulfur protein 7, mitochondrial isoform X1	18.344	0.071	0.061	0.204	0.156
GB55549	lachesin-like isoform X1	18.310	-0.165	0.018	0.289	0.009
GB42644	nicotinic acetylcholine receptor alpha2 subunit precursor	18.299	-0.691	-0.258	0.139	0.157
GB47663	uncharacterized protein LOC727019 isoform X2	18.289	-0.610	-0.259	0.556	0.315
GB54598	sodium-dependent phosphate transporter 2-like	18.284	0.447	-0.018	0.254	0.018
GB50123	myophilin-like	18.225	0.124	-0.264	0.226	0.216
GB42676	epimerase family protein SDR39U1-like	18.154	-0.300	-0.087	0.178	0.130
GB41845	dual specificity protein phosphatase Mpk3	18.147	-0.048	0.017	0.246	0.151
GB41839	uncharacterized protein LOC552552 isoform X1	18.135	-0.629	-0.129	0.083	-0.075
GB51917	1-acyl-sn-glycerol-3-phosphate acyltransferase alpha-like isoform X1	18.077	-0.788	0.146	0.442	0.340
GB41863	glutamate receptor-interacting protein 1	17.991	-0.161	-0.028	0.184	-0.038
GB41860	serine/threonine-protein kinase STE20 isoform X1	17.951	-0.709	0.516	0.417	0.374
724169	transcription factor kayak isoform X3	17.830	-0.339	0.057	0.371	0.313
GB48151	F-box/LRR-repeat protein 16	17.765	0.289	0.071	0.421	0.225
GB47728	uncharacterized protein LOC409327 isoform X2	17.741	0.045	-0.034	0.520	0.241
410562	serine/threonine-protein kinase SBK1-like isoform X1	17.709	-0.327	0.390	0.569	-0.139
GB48232	uncharacterized protein LOC552578 isoform X1	17.669	-0.636	-0.096	0.494	0.094
GB45497	histone-lysine N-methyltransferase, H3 lysine-79 specific isoform X3	17.638	-0.864	-0.204	0.304	-0.184
GB40703	cadherin-23-like	17.589	0.155	-0.149	-0.353	0.681
GB44498	uncharacterized protein LOC551934 isoform X3	17.499	0.209	-0.260	0.386	0.122
GB41241	SET and MYND domain-containing protein 4-like	17.495	0.061	-0.139	0.353	0.028
GB44970	RUN domain-containing protein 1-like	17.329	-0.932	0.012	0.208	0.059
GB45406	innexin shaking-B isoform X1	17.232	-0.513	-0.167	0.593	0.132
GB54827	synaptotagmin 1	17.230	0.403	-0.154	0.244	-0.169
GB44889	neurogenic protein big brain	17.200	-0.692	-0.004	0.370	0.535
GB41487	transcription factor SPT20 homolog isoform X3	17.170	-0.229	-0.029	0.173	0.169
GB40735	fructose-bisphosphate aldolase-like isoform X1	17.026	0.050	0.066	0.168	0.071
GB40603	uncharacterized protein LOC410369	17.025	-0.249	-0.116	0.393	-0.201
GB50150	major facilitator superfamily domain-containing protein 6-like isoform X2	17.015	0.194	-0.231	0.440	0.435
GB52350	BRO1 domain-containing protein BROX-like	16.895	0.025	-0.015	0.063	0.032
GB55976	zinc finger protein 271-like isoform X2	16.892	0.335	0.004	0.223	0.205
GB49559	intraflagellar transport protein 88 homolog isoform X2	16.889	0.267	0.051	0.271	0.109
GB44910	nuclear receptor-binding protein homolog	16.815	0.047	-0.098	0.144	0.158
GB45833	putative methyltransferase NSUN7-like isoform X1	16.699	-1.093	-0.116	0.511	-0.039
GB49688	peroxidase isoformX2	16.659	-0.101	-0.224	0.104	0.008
GB53163	transient receptor potential channel pyrexia isoform X3	16.648	-0.331	-0.341	0.295	0.283
GB51074	differentially expressed in FDCP 8 homolog isoform X2	16.564	-0.143	0.142	0.299	0.038
GB48709	uncharacterized protein LOC724300	16.559	0.102	0.059	0.440	0.327
GB50985	yorkie homolog isoform X2	16.551	-0.059	-0.027	0.262	0.180
GB48331	GTP-binding protein RAD-like isoform X2	16.457	-0.417	0.235	0.769	0.434
GB55798	cyclin-dependent kinase 5 activator 1 isoform X2	16.279	0.095	-0.066	0.369	0.136
GB42775	transmembrane protein 189-like	16.243	-0.724	-0.164	0.252	0.089
GB49967	NADP-dependent malic enzyme isoform X1	16.165	-0.404	-0.086	0.292	0.279
412927	uncharacterized protein LOC412927 isoform X4	16.110	-0.775	-0.120	0.475	0.109
GB47799	protein hairy isoform X2	16.106	-0.516	-0.104	0.333	0.016
GB56017	paired mesoderm homeobox protein 2-like isoform X2	16.098	-0.211	-0.343	0.537	0.384
GB46581	ubiquitin-conjugating enzyme E2 H	16.096	0.016	-0.015	-0.119	0.089
GB40541	vesicular inhibitory amino acid transporter-like	16.089	0.269	0.160	0.651	0.414
GB42792	uncharacterized protein LOC409805 isoform X3	15.973	0.288	-0.280	0.444	0.415

(continued)

Gene	Name	k	am_fc	bt_fc	lf_fc	ln_fc
GB45017	RNA pseudouridylate synthase domain-containing protein 2-like isoform X3	15.970	-0.317	-0.012	0.203	0.216
GB53646	polycomb group protein Pc	15.933	-0.096	-0.035	0.120	0.195
GB44011	E3 ubiquitin-protein ligase Rnf220-like isoform X5	15.909	-0.387	-0.027	0.261	-0.211
GB42757	protein disabled isoform X4	15.903	-0.586	0.060	0.237	0.041
GB50442	uncharacterized protein LOC100577562 isoform X1	15.870	0.230	0.419	0.775	0.308
GB54037	uncharacterized protein LOC727502	15.794	-0.111	-0.143	0.801	0.065
410229	slit homolog 3 protein	15.715	0.168	-0.206	0.050	-0.044
GB47148	uncharacterized protein LOC552326	15.714	0.105	0.024	1.346	0.240
GB53986	dehydrogenase/reductase SDR family member 11-like isoform X1	15.709	0.304	-0.338	0.260	0.133
GB51159	uncharacterized protein LOC413925 isoform X5	15.705	-0.474	-0.208	0.288	0.107
GB48933	methenyltetrahydrofolate synthase domain-containing protein-like	15.668	-0.876	0.034	0.573	0.176
GB42606	serotonin receptor	15.665	-0.040	-0.061	0.698	0.011
GB43365	nucleoside diphosphate kinase 7	15.647	-0.357	-0.010	0.281	0.338
411207	CCAAT/enhancer-binding protein	15.645	-0.620	-0.192	0.359	-0.052
724740	forkhead box protein G1	15.639	-0.201	0.232	0.606	0.168
GB48451	protein yippee-like 5-like isoform X1	15.605	0.225	0.083	0.117	0.090
GB46312	cuticular protein 22 precursor	15.572	0.394	-0.190	0.200	-0.543
GB40304	ankyrin repeat and SOCS box protein 16-like isoform X2	15.563	-0.370	-0.022	0.413	-0.034
GB40240	myosin regulatory light chain 2	15.537	0.520	-0.481	0.258	0.279
GB42875	ATP-binding cassette sub-family A member 2-like isoformX2	15.495	-0.202	-0.064	0.301	-0.163
GB41243	uncharacterized protein LOC408729 isoform X2	15.481	-0.096	-0.146	0.160	-0.086
GB47237	protein naked cuticle homolog 2-like	15.467	-1.459	0.205	0.316	0.036
GB49400	protein msta, isoform A-like isoform X3	15.437	0.213	0.069	0.410	0.073
726815	phosphatidylinositol-glycan biosynthesis class W protein-like	15.406	-0.621	-0.043	0.253	0.056
GB44903	calcineurin subunit B type 2-like	15.400	-0.369	-0.032	0.259	0.340
GB40496	aftiphilin-like isoform X2	15.354	0.312	-0.002	0.152	0.067
GB40159	ankyrin repeat and death domain-containing protein 1A-like isoform X3	15.339	-0.929	-0.171	0.186	-0.092
GB47229	heparan sulfate glucosamine 3-O-sulfotransferase 3A1 isoform X1	15.277	0.749	0.112	0.457	0.084
GB53055	nicotinic acetylcholine receptor beta1 subunit precursor	15.276	0.805	0.655	0.357	0.467
GB48208	protein argonaute-2 isoform X4	15.254	-0.209	-0.148	0.630	0.243
100576814	uncharacterized protein LOC100576814	15.236	0.346	-0.234	1.164	-0.163
GB41745	LOW QUALITY PROTEIN: serine/threonine-protein kinase atr-like	15.164	-0.522	0.006	-0.063	0.154
GB41981	jmjC domain-containing protein 4-like isoform 1	15.090	-0.683	0.069	0.227	0.186
GB40416	twist isoform X1	15.050	0.175	-0.195	0.335	0.037
GB40545	microprocessor complex subunit DGCR8-like isoform X4	15.014	-0.422	0.164	0.306	0.165
GB47014	wiskott-Aldrich syndrome protein family member 2-like isoform X2	14.993	-0.479	0.049	0.072	-0.370
GB42866	bruchpilot	14.984	-0.673	-0.111	0.007	0.437
GB52956	synaptotagmin-10	14.956	0.140	-0.050	0.302	0.482
GB45035	histone acetyltransferase KAT6B-like	14.951	0.456	-0.038	0.409	0.548
100578339	condensin-2 complex subunit D3-like	14.922	-0.945	0.066	0.361	0.026
GB53550	heat shock protein beta-1-like isoform X3	14.853	0.114	-0.110	-0.081	0.227
GB48698	tetraspanin-7 isoform X1	14.851	1.042	0.095	0.207	0.143
GB55663	LOW QUALITY PROTEIN: LIM/homeobox protein Lhx1-like	14.790	-0.323	0.240	0.422	-0.075
GB51063	uncharacterized protein LOC552276 isoform X1	14.736	-0.359	-0.597	0.158	0.036
GB48352	TBC1 domain family member 14	14.723	-0.439	0.052	0.339	-0.236
GB42670	WD repeat-containing protein 35-like	14.693	0.314	0.003	0.342	-0.007
GB49945	BRCA1-associated RING domain protein 1-like isoform X1	14.683	-0.055	-0.259	0.197	0.419
GB44824	corazonin receptor isoform X1	14.679	-0.301	-0.156	-0.147	-0.113
GB42576	limbic system-associated membrane protein-like	14.616	-0.659	-0.095	0.246	0.247
GB41082	tyrosine-protein kinase Src42A-like isoform X5	14.596	0.039	0.034	0.688	0.090
GB43456	18-wheeler precursor	14.596	-0.861	0.068	-0.199	0.026
GB53305	LOW QUALITY PROTEIN: protein still life, isoform SIF type 1-like	14.532	0.134	-0.144	0.630	-0.010
GB43389	8-oxo-dGDP phosphatase NUDT18-like	14.494	0.307	-0.116	0.241	-0.076

(continued)

Gene	Name	k	am_fc	bt_fc	lf_fc	ln_fc
GB49368	rho-related GTP-binding protein RhoU-like	14.452	0.199	-0.008	0.152	0.149
GB49105	ecdysteroid-regulated gene E74 isoform X10	14.440	-0.381	-0.103	2.921	0.142
GB44276	discoidin domain-containing receptor 2-like	14.363	0.656	-0.139	0.670	0.101
GB49248	tachykinins precursor	14.346	0.679	-0.024	0.264	0.301
GB49476	lysyl oxidase homolog 4 isoform X2	14.253	-0.414	-0.144	0.444	0.005
GB41265	nucleoredoxin-like isoform X3	14.208	-0.025	0.231	0.296	0.045
GB50480	mitochondrial inner membrane protein COX18-like isoform X1	14.197	-0.050	-0.148	0.340	0.440
GB50689	L-threonine 3-dehydrogenase, mitochondrial-like	14.182	0.594	-0.033	0.404	0.130
102653641	glomulin-like	14.154	-0.567	-0.201	0.256	0.111
GB54537	protein slit isoform X2	14.075	-0.013	-0.084	0.720	0.425
GB43143	tubulin-specific chaperone cofactor E-like protein-like isoform X2	14.024	0.061	-0.081	0.184	-0.094
GB51029	band 4.1-like protein 5-like isoform X1	14.002	-0.667	-0.055	0.089	-0.002
GB52719	segmentation protein Runt-like	13.760	-1.288	0.176	0.062	-0.031
GB47734	uncharacterized protein LOC100577325 isoform 1	13.722	0.224	-0.059	0.249	0.100
GB41085	tektin-B1-like isoform X3	13.701	-0.375	1.054	0.502	0.436
GB52002	agrin-like isoform X10	13.648	-0.381	0.150	0.166	0.042
GB53665	tyramine beta hydroxylase	13.555	-0.734	-0.033	1.279	0.063
550870	uncharacterized protein LOC550870	13.547	-1.865	0.320	-0.010	-0.021
GB43719	alpha-catulin-like isoform X2	13.487	-0.504	0.238	0.420	0.831
GB44254	uncharacterized protein LOC411586 isoform X1	13.355	0.155	-0.076	0.381	0.366
GB54467	probable G-protein coupled receptor 52 isoform 1	13.157	-1.060	0.124	0.453	0.316
GB52328	protein patched-like isoform X4	13.143	-0.047	0.083	0.472	-0.117
102654980	transient receptor potential channel pyrexia-like	13.088	-0.503	0.027	0.691	0.115
GB49844	mediator of RNA polymerase II transcription subunit 1 isoform X1	13.039	-0.242	-0.034	0.506	-0.067
GB51276	protocadherin-like wing polarity protein stan-like isoform X1	12.994	-0.441	-0.021	0.525	-0.077
GB43618	aconitate hydratase, mitochondrial-like isoform X1	12.878	-0.408	-0.151	0.137	0.128
GB43292	uncharacterized protein LOC551661	12.863	-0.646	0.201	0.319	0.288
GB49175	4-hydroxyphenylpyruvate dioxygenase-like	12.857	-0.346	0.354	0.565	0.181
GB47373	protein tipE-like isoform X1	12.801	-0.407	0.434	0.385	0.001
550677	protein mab-21-like isoform 1	12.777	0.348	0.213	0.303	0.023
GB47370	rho GTPase-activating protein 7 isoform X2	12.775	-0.375	-0.073	-0.449	-0.339
GB55974	calcium uniporter protein, mitochondrial-like isoform X1	12.680	0.369	0.148	0.224	0.395
GB52995	coiled-coil domain-containing protein 85C-like isoform X2	12.655	0.501	0.146	0.362	-0.179
GB53420	uncharacterized protein LOC100576355 isoformX2	12.617	-0.591	0.209	0.378	-0.016
GB40120	fas apoptotic inhibitory molecule 1-like	12.590	0.310	-0.595	0.099	0.308
GB41601	serine/threonine-protein phosphatase 6 regulatory ankyrin repeat subunit A-like isoform 2	12.553	0.239	0.032	0.254	-0.291
GB41227	cuticular protein analogous to peritrophins 3-B precursor	12.501	0.132	-0.668	0.409	0.056
GB52992	agrin-like	12.496	0.023	-0.073	0.246	-0.304
GB53351	2-hydroxyacylsphingosine 1-beta-galactosyltransferase-like isoform 1	12.390	-0.693	-0.035	1.091	0.371
GB52356	zeta-sarcoglycan isoformX2	12.330	0.222	-0.300	0.349	-0.251
GB42227	homeobox protein aristaless-like	12.322	0.337	0.045	-0.398	0.123
GB47330	uncharacterized protein LOC100578466	12.297	-0.161	0.280	2.191	-0.058
GB47902	endocuticle structural glycoprotein SgAbd-1-like	11.965	0.919	-0.329	0.255	0.410
GB44295	sodium-dependent neutral amino acid transporter B(0)AT2 isoform X1	11.870	-0.039	0.015	0.260	0.072
GB51084	radial spoke head protein 3 homolog isoform X1	11.741	0.129	-0.970	0.301	0.066
GB47967	TWiK family of potassium channels protein 7-like	11.625	0.259	0.119	0.539	0.308
GB49882	potassium voltage-gated channel protein Shaw isoform X2	11.395	-0.034	-0.144	0.275	0.098
GB45694	lutropin-choriogonadotropic hormone receptor-like isoform X4	11.315	-0.376	-0.256	1.208	0.441
100578156	uncharacterized protein LOC100578156	11.077	-1.570	0.078	0.624	0.111
GB41494	uncharacterized protein LOC100576666	10.876	0.240	0.198	1.072	0.309
409016	uncharacterized protein LOC409016 isoform X1	10.234	0.189	-0.045	0.194	0.626
GB48262	uncharacterized LOC100576671, transcript variant X5	10.198	-0.682	0.034	0.081	0.051
GB51174	uncharacterized protein DDB_G0284459-like	10.040	0.428	-0.375	0.187	0.593
GB46638	dipeptidase 1-like	9.993	-0.276	-0.736	-0.421	0.310
GB46057	PBAN-type neuropeptides precursor	9.971	0.027	-0.437	0.158	0.113

(continued)

Gene	Name	k	am__fc	bt__fc	lf__fc	ln__fc
GB55016	quinone oxidoreductase-like isoform X2	9.864	0.622	-0.080	0.465	-0.193
GB49392	actin-binding Rho-activating protein-like isoform 1	9.260	0.787	-0.188	0.332	0.185
GB48832	cuticular protein 3 precursor	9.092	1.540	-0.194	0.396	0.178
GB54396	elongation of very long chain fatty acids protein AAEL008004-like isoform X2	8.960	2.488	-0.085	0.973	0.051
GB46335	uncharacterized protein LOC100577622	8.871	0.276	0.188	1.173	0.070
GB45199	ceramide kinase-like isoform X4	8.783	0.225	0.069	0.189	-0.104
724465	enhancer of split mbeta protein-like	8.744	1.091	0.005	0.608	-0.133
GB41903	cyclin N-terminal domain-containing protein 1-like isoform X2	8.419	-0.188	0.482	0.480	0.717
724570	mpv17-like protein 2-like isoform X2	8.120	0.268	-0.018	0.168	0.125
GB44549	glucose oxidase	7.628	0.046	0.453	0.139	-0.585
GB47092	uncharacterized protein LOC724483 isoform X1	7.416	-0.525	0.725	0.485	-0.054
GB50889	RNA-binding protein 24-like isoform X3	7.323	-0.034	0.013	0.650	0.152
GB43560	insulin-like peptide 2	7.254	0.389	-0.154	0.288	0.217
GB51740	CD63 antigen	7.149	0.300	-0.111	2.628	0.093
GB49684	optomotor-blind protein-like isoform X1	7.088	0.110	0.184	0.093	-0.184
GB45542	ligand-gated chloride channel homolog 3 precursor	6.964	1.987	0.198	0.931	0.474
GB51369	opsin, ultraviolet-sensitive	6.544	0.528	0.069	0.174	0.218
GB45157	protein big brother isoform 1	5.983	-0.165	0.231	0.254	-0.045
GB13601	cuticular protein CPF1 precursor	5.919	0.521	-0.166	0.344	0.114
GB41418	uncharacterized protein PF11__0207-like isoform X2	5.728	0.673	0.073	0.169	0.761
GB47727	uncharacterized protein LOC724679 isoform 2	4.796	0.590	-0.162	0.341	0.070
102656656	carbon catabolite-derepressing protein kinase-like isoform X2	4.511	-0.279	-0.012	0.475	0.048
GB54159	uncharacterized protein LOC552735	4.176	0.329	0.154	0.367	0.326

Supplementary Table 29: List of all the genes in Module 4, ranked by their within-module connectivity, k. The latter four columns give the Log_2 fold-change in expression in response to queen pheromone in each of the four species.

Gene	Name	k	am_fc	bt_fc	lf_fc	ln_fc
GB44431	26S protease regulatory subunit 4 isoform 1	44.224	0.207	-0.042	-0.428	-0.009
GB49337	26S proteasome non-ATPase regulatory subunit 13	42.984	0.176	-0.066	-0.261	-0.561
GB50750	coatomer subunit delta isoform 2	42.890	-0.031	-0.029	-0.059	-0.049
GB45720	proteasome subunit alpha type-7-1-like	41.581	0.153	-0.095	-0.072	0.063
GB50242	26S proteasome non-ATPase regulatory subunit 4	41.384	0.221	0.063	-0.133	0.594
GB53174	programmed cell death 6-interacting protein isoform X1	41.243	-0.076	-0.016	-0.047	-0.055
GB47189	H(+)/Cl(-) exchange transporter 3-like isoform X2	41.034	0.035	-0.015	-0.102	-0.045
GB50252	GTP-binding protein SAR1b-like isoform X4	40.648	0.321	-0.067	-0.021	-0.222
102656372	tropinone reductase 2-like	39.869	0.112	0.034	0.324	0.493
GB53812	prolyl endopeptidase-like isoformX1	39.533	-0.080	-0.049	-0.093	-0.024
GB45567	E3 ubiquitin-protein ligase MARCH5 isoform X5	39.471	-0.055	0.035	0.475	0.013
GB41649	E3 ubiquitin-protein ligase MARCH6	39.279	-0.046	-0.064	-0.068	0.050
GB43819	ATPase ASNA1 homolog	39.180	0.377	-0.007	-0.143	-0.010
GB44941	eukaryotic peptide chain release factor GTP-binding subunit ERF3A	39.104	-0.059	0.072	-0.015	-0.110
GB53690	protein transport protein Sec31A	38.708	-0.007	0.031	-0.118	-0.032
GB48643	STT3, subunit of the oligosaccharyltransferase complex, homolog B	37.963	0.193	0.001	-0.160	-0.095
GB50348	bleomycin hydrolase-like isoform X2	37.859	-0.033	0.036	-0.055	0.062
GB43706	probable trans-2-enoyl-CoA reductase, mitochondrial-like	37.474	-0.011	0.005	-0.013	0.035
GB40775	apoptosis-inducing factor 1, mitochondrial	36.869	0.200	0.191	0.317	0.033
411789	protein extra bases	36.857	0.189	0.021	-0.129	0.101
GB40483	xyloside xylosyltransferase 1-like	36.853	0.030	0.006	-0.081	0.068
GB41617	methylcrotonoyl-CoA carboxylase beta chain, mitochondrial-like isoform X2	36.788	0.149	-0.169	-0.239	-0.059
GB50289	signal recognition particle 54 kDa protein-like	36.641	0.004	-0.001	-0.014	-0.020
GB50274	transitional endoplasmic reticulum ATPase TER94	36.259	0.544	-0.118	-0.306	-0.078
GB47540	putative leucine-rich repeat-containing protein DDB_G0290503 isoform X2	36.215	0.278	0.023	-0.129	0.022
GB47573	T-complex protein 1 subunit theta-like	36.082	0.156	0.051	-0.021	-0.062
GB52675	26S proteasome non-ATPase regulatory subunit 12	35.930	0.231	0.048	-0.095	0.783
GB42355	asparagine–tRNA ligase, cytoplasmic-like	35.912	0.129	0.102	-0.122	-0.043
GB42329	segmentation protein cap'n'collar-like isoform X4	35.867	-0.219	-0.101	-0.121	0.601
GB55494	probable nucleolar GTP-binding protein 1-like isoform 1	35.759	0.045	0.096	3.822	-0.122
GB42773	alanine–tRNA ligase, cytoplasmic-like isoform X1	35.229	-0.035	0.042	-0.087	0.012
GB50730	97 kDa heat shock protein isoformX1	35.064	-0.158	-0.029	-0.211	-0.161
GB48313	transmembrane 9 superfamily member 3	34.685	0.253	-0.014	-0.031	-0.035
GB50459	WD repeat-containing protein 36-like	34.579	-0.148	-0.043	-0.243	-0.123
GB46035	eukaryotic translation initiation factor 4 gamma 2-like isoform X4	33.922	-0.221	0.030	-0.243	-0.149
GB54608	probable elongator complex protein 2-like	33.814	0.069	0.069	-0.221	-0.131
GB50177	protein TRC8 homolog	33.493	0.095	0.123	-0.173	0.247
GB45258	isocitrate dehydrogenase [NADP] cytoplasmic isoform 2	33.421	-0.014	-0.016	-0.119	-0.099
GB47114	dolichyl-diphosphooligosaccharide–protein glycosyltransferase subunit 2-like	33.137	0.135	0.104	-0.115	-0.111
GB44418	protein suppressor of hairy wing isoform X2	33.080	-0.411	-0.126	-0.272	0.028
GB54573	probable 26S proteasome non-ATPase regulatory subunit 3 isoform X2	31.929	0.054	-0.004	-0.068	-0.905
GB55440	phosphatidylinositol transfer protein alpha isoform	31.910	0.165	0.007	-0.046	0.063
GB41762	derlin-2-like	31.705	0.184	0.087	-0.221	-0.124
GB49939	protein FAM188A homolog	31.552	-0.141	0.114	-0.191	0.039
GB42648	dolichyl-diphosphooligosaccharide–protein glycosyltransferase subunit 1	31.481	0.266	0.160	-0.123	-0.166
GB44670	luciferin 4-monooxygenase-like	31.431	-0.052	0.155	1.103	0.056
GB43131	short-chain dehydrogenase/reductase family 16C member 6-like isoform X4	31.410	0.101	0.087	-0.199	-0.083
GB48692	translocation protein SEC63 homolog isoform 1	31.126	0.286	-0.053	-0.160	0.021
GB49083	casein kinase II subunit beta isoform X1	30.992	0.278	0.047	-0.111	0.070
GB41427	catalase	30.733	0.143	0.074	-0.398	-0.012
GB46735	eukaryotic translation initiation factor 2A-like	30.705	-0.041	0.013	-0.145	-0.011

(continued)

Gene	Name	k	am_fc	bt_fc	lf_fc	ln_fc
GB47296	transmembrane protein 19-like isoform X2	30.673	0.429	-0.055	-0.252	-0.103
GB52168	von Willebrand factor A domain-containing protein 8-like	30.577	0.086	-0.074	0.001	0.070
GB48812	dnaJ homolog subfamily C member 3	30.546	0.113	0.040	-0.220	0.031
102655967	ancient ubiquitous protein 1-like	30.348	0.471	0.042	0.165	0.050
551499	FIT family protein CG10671-like	29.741	0.379	-0.060	-0.318	-0.035
GB49955	vacuole membrane protein 1 isoform X1	29.459	-0.044	-0.007	-0.296	0.198
GB41285	receptor-binding cancer antigen expressed on SiSo cells	29.433	0.024	0.120	-0.140	-0.131
GB54861	LOW QUALITY PROTEIN: counting factor associated protein D-like	29.320	0.230	0.193	-0.294	-0.097
GB47462	protein disulfide-isomerase A3 isoform 2	29.269	0.305	0.077	-0.256	0.137
GB46646	UPF0554 protein C2orf43 homolog	28.708	0.335	-0.050	-0.218	-0.143
GB44396	atlastin isoform X2	28.557	-0.059	-0.178	-0.093	0.320
GB53373	leucine-rich repeat-containing protein 58-like	28.368	0.184	0.057	-0.260	0.158
GB55484	UBX domain-containing protein 7-like	28.345	-0.142	0.086	-0.177	-0.048
GB47134	renin receptor-like isoform X1	28.303	0.330	-0.041	0.372	0.417
GB43912	nicalin-1 isoform X1	28.248	0.629	0.088	-0.325	-0.010
GB46120	aspartate aminotransferase, mitochondrial isoform 1	28.225	0.249	0.024	0.131	0.025
GB49525	RNA-binding protein fusilli	28.209	0.117	-0.074	-0.035	0.053
GB48597	NADH-cytochrome b5 reductase 2-like isoform X2	28.155	0.203	0.001	-0.100	-0.020
GB44205	proteasome subunit beta type-5-like	28.007	0.484	-0.092	-0.434	-0.060
GB52434	probable ribosome production factor 1-like	27.874	0.005	0.015	-0.206	0.018
GB40779	transaldolase	27.761	-0.004	0.016	-0.044	0.034
GB45582	facilitated trehalose transporter Tret1-like isoform 3	27.375	-0.050	-0.134	0.054	-0.007
GB45698	SAGA-associated factor 11 homolog	27.344	-0.265	0.092	-0.138	0.188
GB40265	transcriptional activator protein Pur-beta-B-like isoform X4	27.334	0.126	-0.039	-0.129	-0.078
GB47392	protein BCCIP homolog	27.259	0.258	0.110	0.744	0.133
GB55987	ras-related protein Rab-18-B	27.111	0.032	-0.054	-0.035	-0.050
GB55977	eukaryotic translation initiation factor 4E-1A	27.090	0.171	0.054	-0.095	0.103
GB45251	ubiA prenyltransferase domain-containing protein 1 homolog	26.995	0.229	0.011	-0.116	0.079
GB48008	peroxisomal membrane protein PEX14-like	26.970	0.000	0.022	-0.207	-0.080
GB46977	ribosome biogenesis methyltransferase WBSCR22-like	26.952	0.048	0.090	-0.206	0.089
GB54363	ATPase family AAA domain-containing protein 3 isoform X1	26.942	0.394	0.163	-0.150	-0.056
GB53955	FGFR1 oncogene partner 2 homolog	26.810	0.085	-0.031	-0.144	0.096
GB51282	thioredoxin domain-containing protein 5-like isoform 1	26.692	0.249	0.076	0.146	-0.011
GB46031	vacuolar H+ ATP synthase 16 kDa proteolipid subunit	26.646	-0.048	-0.017	-0.283	0.022
GB54848	tumor suppressor candidate 3-like	26.523	0.485	-0.010	-0.627	-0.001
GB49119	deoxynucleotidyltransferase terminal-interacting protein 2-like	26.458	0.211	-0.014	-0.107	0.060
GB53080	alpha-2-macroglobulin receptor-associated protein-like	26.456	0.006	-0.031	-0.087	0.037
GB49117	heat shock protein cognate 3 precursor	26.361	0.227	-0.040	-0.293	-0.158
GB49240	aldehyde dehydrogenase, mitochondrial isoform 1	26.349	0.155	0.101	-0.038	-0.009
GB55610	MOSC domain-containing protein 2, mitochondrial-like	26.339	-0.063	0.007	0.324	-0.140
GB49307	DNA-directed RNA polymerases I and III subunit RPAC1-like isoform X1	26.294	-0.012	0.067	0.339	-0.037
GB52729	aspartate–tRNA ligase, cytoplasmic	26.270	0.127	-0.010	7.481	-0.036
GB40207	serine–tRNA ligase, mitochondrial	26.269	0.001	-0.072	0.002	-0.045
GB46979	derlin-1-like	26.162	0.681	-0.034	0.510	-0.014
GB42236	patched domain-containing protein 3-like isoform X4	25.938	-0.289	0.090	-0.115	0.114
102653839	histone-lysine N-methyltransferase SETMAR-like	25.496	0.206	-0.064	-0.227	-0.174
GB49180	cysteine-rich secretory protein 1-like, transcript variant X5	25.371	-0.254	-0.039	-0.119	-0.252
GB55537	transketolase isoform 1	25.321	1.036	0.037	-0.248	-0.342
GB54999	NAD kinase 2, mitochondrial-like	25.209	0.454	-0.214	-0.082	-0.092
GB54101	HEAT repeat-containing protein 3-like	24.884	-0.225	-0.087	-0.105	0.060
GB55490	uncharacterized protein LOC410793	24.753	-0.048	-0.068	-0.473	0.262
GB46579	glucose-6-phosphate 1-dehydrogenase isoform X3	24.688	0.648	-0.100	-0.365	-0.159
GB50096	pantothenate kinase 1-like isoform X2	24.587	0.848	-0.087	-0.369	-0.027
GB54112	adenine phosphoribosyltransferase isoform X1	24.536	0.235	0.045	1.476	0.104
GB47432	5-aminolevulinate synthase, erythroid-specific, mitochondrial-like	24.361	0.307	-0.249	-0.361	-0.039
GB40783	glucose-6-phosphate isomerase-like	24.208	-0.244	-0.102	-0.091	-0.001
GB54298	stromal cell-derived factor 2-like protein 1-like isoformX2	24.138	0.318	0.052	-0.217	1.519
GB44457	FGGY carbohydrate kinase domain-containing protein-like isoform X2	23.955	0.302	-0.161	-0.235	-0.071

(continued)

Gene	Name	k	am_fc	bt_fc	lf_fc	ln_fc
GB48408	protein catecholamines up	23.948	0.014	-0.012	-0.109	0.073
GB48847	DNA replication licensing factor Mcm3	23.787	-0.308	0.250	-0.300	0.120
GB46657	galactokinase-like	23.579	0.100	0.068	-0.518	0.020
GB52347	saccharopine dehydrogenase-like oxidoreductase-like isoform 1	23.401	0.365	-0.495	0.253	-0.200
GB51782	carboxypeptidase Q-like isoform 1	23.388	0.335	-0.020	-0.065	-0.166
GB48308	probable pyruvate dehydrogenase E1 component subunit alpha, mitochondrial-like isoform X2	23.312	-0.284	-0.108	0.171	0.518
GB44557	probable ribonuclease ZC3H12C-like isoformX1	23.279	0.074	-0.045	-0.144	-0.327
GB47941	cyclic AMP response element-binding protein A-like	23.226	-0.340	0.129	-0.145	-0.307
GB42732	long-chain-fatty-acid–CoA ligase 3-like isoform X2	23.219	0.499	-0.015	-0.325	-0.157
GB52724	protein 5NUC-like isoform X2	23.028	0.097	0.021	-0.118	0.002
GB46772	very-long-chain enoyl-CoA reductase-like	23.018	0.747	-0.220	-0.201	-0.431
GB44008	BTB/POZ domain-containing protein 17 isoform X1	22.840	0.315	0.117	-0.112	-0.048
GB55511	growth/differentiation factor 8-like isoform 1	22.817	0.435	0.159	-0.487	0.074
GB54601	protein disulfide-isomerase A6-like isoform 1	22.776	0.432	0.125	-0.147	-0.078
GB49342	sugar phosphate exchanger 2-like isoform X3	22.718	-0.224	-0.011	-0.007	0.025
GB49348	transmembrane protein 115-like	22.715	0.339	-0.038	-0.031	-0.029
GB41388	glycerol-3-phosphate dehydrogenase	22.669	-0.085	-0.139	-0.188	0.256
GB49336	acetyl-CoA carboxylase-like isoform X9	22.667	0.395	-0.160	0.187	-0.227
GB54056	serine hydroxymethyltransferase, cytosolic isoform X3	22.549	0.414	0.193	-0.326	-0.261
GB44640	solute carrier family 52, riboflavin transporter, member 3-A-like isoform X2	22.453	-0.128	-0.103	0.188	-0.217
GB49826	sterol O-acyltransferase 1-like	22.192	0.095	0.147	-0.284	0.023
GB47694	globin 1	22.014	-0.051	0.109	-0.152	-0.101
GB52074	6-phosphogluconate dehydrogenase, decarboxylating	21.967	0.810	-0.143	-0.445	-0.603
GB48195	acyl-CoA Delta(11) desaturase-like	21.938	0.494	-0.193	0.700	0.669
GB45213	acyl-CoA synthetase short-chain family member 3, mitochondrial-like isoform X2	21.834	0.651	0.224	-0.564	-0.240
GB50680	mannose-P-dolichol utilization defect 1 protein homolog isoform X2	21.556	0.466	-0.393	-0.221	-0.049
GB45775	pancreatic triacylglycerol lipase-like isoform X2	21.506	0.320	0.051	-0.888	0.069
GB41916	uncharacterized protein LOC726658 isoform 1	21.371	0.336	0.373	-0.028	-0.017
GB52458	cysteine-rich with EGF-like domain protein 2-like	21.265	-0.271	0.050	-0.361	-0.018
GB40278	probable methylmalonate-semialdehyde dehydrogenase [acylating], mitochondrial isoform X4	21.242	-0.158	0.003	0.029	-0.034
GB54216	ATP-citrate synthase isoform X2	21.224	0.846	-0.227	-0.446	-0.326
552211	protein THEM6-like	21.185	0.738	-0.144	0.392	-0.597
GB55533	RNA-binding protein squid-like	20.857	0.065	0.078	-0.091	-0.117
GB48859	UPF0160 protein MYG1, mitochondrial-like isoform X2	20.819	0.236	0.249	-0.208	-0.033
GB49433	H/ACA ribonucleoprotein complex subunit 2-like protein	20.791	0.552	0.120	-0.163	0.002
GB42237	N6-adenosine-methyltransferase 70 kDa subunit-like	20.731	-0.089	0.276	-0.086	-0.053
GB46921	monocarboxylate transporter 12-like	20.715	0.181	0.001	-0.100	-0.215
GB45596	elongation of very long chain fatty acids protein 6-like	20.705	0.525	-0.497	-0.530	-0.508
GB50013	proclotting enzyme	20.602	0.251	-0.977	-0.533	-0.291
GB55263	putative fatty acyl-CoA reductase CG5065-like	20.420	-0.033	-0.262	-0.832	-0.362
GB54404	elongation of very long chain fatty acids protein AAEL008004-like	20.311	2.034	-0.040	-0.182	-0.152
GB55094	protein neuralized isoform X3	20.264	-0.603	0.108	-0.640	-0.024
GB54427	ribonucleoside-diphosphate reductase subunit M2 isoform X2	20.130	0.004	0.160	-0.033	0.018
GB54538	uncharacterized protein LOC411248 isoform X5	20.086	-0.466	-0.229	-0.003	-0.041
GB52768	alkaline phosphatase, tissue-nonspecific isozyme-like isoform X1	20.080	-0.017	0.137	-0.040	-0.039
GB51580	long-chain-fatty-acid–CoA ligase ACSBG2 isoform X1	20.069	-0.099	-0.175	-0.269	-0.122
GB50871	serine/threonine-protein kinase SIK2-like isoform X2	19.917	0.061	-0.144	-0.231	0.213
GB53287	sialin-like isoform X4	19.620	-0.030	-0.106	-0.119	-0.278
GB49653	probable phosphoserine aminotransferase-like	19.478	0.431	0.094	0.059	-0.095
GB47495	nucleotide exchange factor SIL1-like	19.345	-0.152	0.158	-0.010	-0.007
GB51723	60S ribosomal export protein NMD3	19.223	-0.071	0.166	0.176	-0.066
GB48628	RNA-binding protein Nova-1-like isoform X2	19.214	0.052	0.021	-0.516	-0.171
GB50626	phospholipase D3-like isoform X7	19.160	-0.177	-0.095	-0.162	-0.037
GB54331	cathepsin L-like isoform X2	19.064	0.017	-0.079	-0.378	-0.064

(continued)

Gene	Name	k	am_fc	bt_fc	lf_fc	ln_fc
GB53412	fatty acid synthase-like	18.868	1.129	-0.317	0.506	-0.180
GB51753	uncharacterized protein LOC100576760 isoform X2	18.821	0.077	0.140	-0.066	-0.163
100577899	DNA replication complex GINS protein SLD5-like	18.733	0.347	0.116	-0.238	-0.044
GB42899	uncharacterized protein LOC551133 isoform X2	18.387	0.129	0.010	-0.010	-0.056
GB52446	uncharacterized protein LOC726987 isoform X5	18.324	-0.519	-0.207	-0.378	-0.177
GB52351	porphobilinogen deaminase-like	18.240	-0.394	-0.080	-0.511	0.030
GB45381	putative sodium-coupled neutral amino acid transporter 7-like	18.233	0.232	-0.299	-0.145	-0.104
GB41886	protein transport protein Sec61 subunit alpha isoform 2	18.216	0.729	-0.033	-0.260	-0.338
GB52153	U3 small nucleolar RNA-associated protein 15 homolog	18.207	0.174	0.147	-0.071	-0.021
GB48203	laminin subunit beta-1 isoform X2	18.135	-0.069	0.055	-0.182	-0.520
GB51647	4-aminobutyrate aminotransferase, mitochondrial-like isoform X2	18.133	0.568	0.096	-0.109	-0.031
GB52454	mitochondrial pyruvate carrier 2-like	18.032	-0.459	0.054	-0.185	0.031
GB49942	mitochondrial dicarboxylate carrier-like isoform 1	17.738	0.189	-0.036	-0.144	-0.088
GB51614	probable methylthioribulose-1-phosphate dehydratase-like	17.687	-0.036	-0.073	2.451	0.093
GB41011	lateral signaling target protein 2 homolog	17.643	-0.076	-0.174	-0.504	-0.091
GB49869	microsomal triglyceride transfer protein large subunit isoform X1	17.590	0.139	0.237	-0.471	-0.879
GB55432	glucosidase 2 subunit beta-like	17.429	0.492	0.115	-0.178	0.043
GB40071	uncharacterized protein LOC410446	17.398	0.076	0.030	1.217	0.085
GB44888	MATH and LRR domain-containing protein PFE0570w-like	17.235	0.043	-0.025	0.008	-0.130
GB54610	thiamine transporter 2-like, transcript variant X2	17.225	-0.477	0.109	-0.187	-0.321
GB54661	phosphoglucomutase isoform X2	17.098	-0.336	-0.114	-0.181	-0.209
GB46422	proton-coupled amino acid transporter 1	16.930	0.209	-0.188	0.012	-0.167
GB45177	uncharacterized protein LOC725324 isoform X1	16.880	0.407	-0.057	-0.143	0.339
GB49633	RNA 3'-terminal phosphate cyclase-like protein-like isoform X2	16.859	0.158	-0.061	-0.091	0.095
GB40141	venom serine carboxypeptidase	16.809	0.122	-0.323	-0.321	-0.088
GB40280	pyruvate carboxylase, mitochondrial isoform X1	16.743	-0.602	-0.051	-0.215	-0.346
GB49757	fatty acid binding protein	16.705	0.415	-0.176	0.359	-0.157
GB46661	sodium-independent sulfate anion transporter-like isoform X1	16.611	-0.285	0.213	-0.054	-0.001
GB45210	translocon-associated protein subunit gamma-like	16.589	-0.258	-0.062	-0.036	-0.138
GB47383	U4/U6 small nuclear ribonucleoprotein Prp4	16.526	0.018	-0.021	-0.039	0.067
GB42787	dentin sialophosphoprotein-like isoform X4	16.515	0.168	0.304	-0.274	-0.099
102655896	nucleoplasmin-like protein-like isoform X4	16.340	0.168	0.184	-0.105	0.025
GB55474	protein pygopus	16.237	0.268	0.071	-0.055	0.073
GB51125	inositol-3-phosphate synthase 1-B isoform X2	16.234	-0.132	0.045	-0.121	-0.143
GB45968	collagen alpha-1(IV) chain-like isoform 1	16.142	-0.102	-0.285	-0.144	-0.185
GB44537	myosin-IA	16.131	-0.330	-0.051	0.033	-0.109
GB45824	phosphoserine phosphatase isoform X2	15.998	0.136	-0.074	-0.152	0.977
GB53567	branched-chain-amino-acid aminotransferase, cytosolic-like isoform 1	15.899	-0.195	-0.151	-0.119	0.025
724293	protein yellow	15.838	0.149	-0.265	0.905	0.012
GB44138	l-2-hydroxyglutarate dehydrogenase, mitochondrial-like isoform X3	15.464	-0.061	-0.065	-0.058	-0.012
GB45975	LIM/homeobox protein Lhx3	15.448	-1.491	0.170	-0.535	-0.210
GB44420	hydroxymethylglutaryl-CoA synthase 1 isoform X2	15.448	0.032	-1.478	-0.234	-0.220
GB43942	putative serine protease K12H4.7-like isoform X2	15.444	0.673	0.016	-0.140	0.179
GB42629	chromatin accessibility complex protein 1-like	15.274	0.125	0.163	-0.087	-0.009
GB42541	carbonic anhydrase-related protein 10-like isoform X3	15.217	-0.090	-0.207	-0.422	-0.116
GB54391	putative glycogen [starch] synthase-like isoform X1	15.171	-0.157	-0.110	-0.033	-0.155
GB52496	epoxide hydrolase 4-like isoform X4	15.111	0.277	0.012	-0.375	-0.080
GB51598	translocon-associated protein subunit beta isoform 2	14.774	0.558	0.037	-0.040	-0.159
GB49095	high affinity copper uptake protein 1-like isoformX1	14.621	0.523	-0.101	-0.273	-0.023
GB54888	2-acylglycerol O-acyltransferase 1-like isoform X1	14.550	0.669	0.047	-0.092	-0.118
GB42264	myb-like protein X-like	14.522	-0.625	-0.137	1.073	-0.007
GB45943	collagen alpha-5(IV) chain	14.385	-0.014	-0.169	-0.031	0.166
GB51236	acyl-CoA Delta(11) desaturase isoform X2	14.283	0.751	0.047	-0.601	0.489
GB47503	delta-1-pyrroline-5-carboxylate synthase-like isoform X3	14.247	-0.453	-0.118	-0.092	-0.092
GB47839	calumenin	14.228	0.251	0.107	1.134	0.654

(continued)

Gene	Name	k	am_fc	bt_fc	lf_fc	ln_fc
GB40747	GMP reductase 2-like isoform 1	14.074	-0.139	-0.085	0.054	0.006
GB55661	neuronal membrane glycoprotein M6-a-like isoform X2	13.870	0.292	0.045	0.041	0.085
GB49854	alpha-amylase precursor	13.836	0.486	0.084	-1.676	0.917
726965	uncharacterized protein LOC726965	13.828	-0.151	-0.132	0.146	-0.322
102655415	uncharacterized protein LOC102655415	13.761	-0.189	-0.206	-0.876	-0.390
GB52114	protein trachealess-like isoform X7	13.707	-0.601	-0.082	2.821	0.008
GB43216	uncharacterized protein LOC413583 isoform X2	13.522	-0.236	-0.166	-0.429	-0.645
GB47449	nucleoporin NUP188 homolog	13.433	0.040	0.126	-0.358	-0.055
GB53230	adipokinetic hormone receptor	13.346	-0.156	0.049	-0.494	-0.391
GB42738	protein cueball-like	13.219	0.079	0.019	-0.328	-0.186
GB42468	phospholipase B1, membrane-associated-like isoform X1	13.019	0.171	-2.655	-0.239	0.128
GB48521	RNA polymerase II elongation factor ELL2-like isoform X1	12.918	-0.011	-0.158	-0.147	0.021
GB49321	D-arabinitol dehydrogenase 1-like	12.779	0.385	-0.031	-0.128	-0.147
GB53404	protein fork head-like isoform 1	12.777	-2.051	0.055	0.476	-0.211
411557	protein FAM46A-like isoformX2	12.640	-0.372	0.154	-0.170	-0.053
GB44850	origin recognition complex subunit 3-like	12.544	0.206	-0.063	-0.251	0.153
GB51077	dystrotelin-like isoform X1	12.365	-0.261	0.155	-0.474	0.044
GB49543	alanine–glyoxylate aminotransferase 2-like	12.352	0.344	-0.128	-0.175	-0.554
GB52712	serine/arginine repetitive matrix protein 2-like isoform X1	12.310	0.135	0.213	-0.157	0.133
GB53036	serine/threonine-protein kinase Warts-like isoform X1	12.295	-0.176	0.028	-0.251	0.010
GB46917	uncharacterized protein LOC726071	12.250	0.137	0.092	-0.165	0.128
GB53661	methyltransferase-like isoform X3	12.133	-0.227	-0.080	2.059	-0.017
GB51278	innexin inx3	12.092	0.383	0.041	0.017	-0.152
GB52161	cuticular protein 28 precursor	11.933	0.284	-0.685	0.049	-0.022
GB42887	protein NPC2 homolog	11.896	0.493	0.117	0.827	-0.254
GB43984	xenotropic and polytropic retrovirus receptor 1 homolog	11.712	-0.039	0.039	0.049	0.149
GB48252	dihydrofolate reductase isoform X2	11.676	0.248	0.224	-0.196	0.104
GB47270	cytochrome P450 4C1	11.593	0.313	0.187	0.745	0.504
GB48109	retinoid-inducible serine carboxypeptidase-like isoform X3	11.545	0.990	0.153	-0.476	0.224
GB54313	uncharacterized protein LOC413386 isoform X3	11.417	-0.268	0.046	-0.574	-0.032
GB51913	thymidylate kinase-like isoform X2	11.335	-0.373	0.308	-0.108	0.097
GB44503	uncharacterized protein LOC727423 isoform X2	11.287	0.833	-0.206	-0.116	0.080
GB53229	WAS protein family homolog 1-like	11.255	0.001	0.139	0.084	-0.092
GB51834	sodium-dependent nutrient amino acid transporter 1-like	11.166	-0.353	-0.262	0.081	0.051
GB52505	chaoptin-like	10.968	0.459	-0.008	-0.377	0.110
GB52275	pancreatic lipase-related protein 2-like	10.794	0.110	0.257	-0.438	0.160
GB55302	trehalose transporter 1 isoform X6	10.668	-0.068	-0.116	-0.221	0.308
102654789	uncharacterized protein LOC102654789	10.667	-0.058	-0.328	-0.610	0.050
GB42616	beta-hexosaminidase subunit beta-like	10.369	-0.229	0.145	0.062	0.062
GB54153	uncharacterized protein LOC100576236 isoform X1	10.118	-0.081	-0.135	-1.402	-0.132
GB47327	lipid phosphate phosphohydrolase 3-like	10.059	0.286	-0.218	0.106	-0.093
GB50021	exonuclease 3'-5' domain-containing protein 2-like isoform X1	9.842	-0.074	0.235	0.271	0.094
GB40344	uncharacterized protein LOC552242	9.829	0.117	-0.802	-0.224	0.503
GB49929	laminin subunit alpha	9.561	0.534	0.243	-0.537	-0.610
GB44663	homeobox protein Nkx-2.4-like	9.552	0.139	-0.365	0.172	-0.190
GB51107	uncharacterized protein LOC100578731 isoform X1	9.377	0.430	0.183	0.138	-0.171
GB50524	uncharacterized protein LOC726417	8.929	-0.059	0.051	-1.391	-0.161
GB51696	hexamerin 70c precursor	8.783	-0.047	-0.263	0.081	0.519
GB51195	protein abrupt-like isoform X5	8.448	-0.065	0.278	-0.345	0.007
GB46800	uncharacterized protein LOC100577231	8.331	-0.631	-0.070	0.531	0.074
GB52656	uncharacterized protein LOC552154	8.300	0.583	-0.215	-0.553	-0.122
GB42799	protein takeout-like	8.251	-0.287	0.252	-1.520	-1.028
GB42426	glutamyl aminopeptidase-like isoform X2	7.940	0.727	0.437	-0.651	0.056
GB53155	maternal embryonic leucine zipper kinase-like	7.860	-0.064	0.069	3.168	0.091
GB44967	GTP:AMP phosphotransferase AK3, mitochondrial isoform X1	7.715	-0.224	-0.026	1.068	0.309
GB48079	trypsin-7	7.663	0.789	-0.649	-0.927	0.097
102656088	uncharacterized protein LOC102656088	7.565	1.657	-0.768	-0.769	-0.730
GB50434	proton-coupled amino acid transporter 1-like	7.268	0.011	-0.126	-0.012	-0.200
GB49813	SUMO-activating enzyme subunit 1	7.001	0.520	0.234	0.651	0.173
GB43181	uncharacterized protein LOC552799 isoform X2	6.972	0.594	0.719	-0.861	0.042

(continued)

Gene	Name	k	am__fc	bt__fc	lf__fc	ln__fc
GB46693	WD repeat-containing protein 65-like	6.868	0.112	-0.068	0.129	-0.136
GB47181	NADH dehydrogenase [ubiquinone] iron-sulfur protein 4, mitochondrial	6.863	-0.113	-0.075	0.504	0.128
GB52667	uncharacterized protein LOC552202 isoform X6	6.665	-0.244	-0.002	-1.112	-0.077
GB53401	protein fosB isoform X1	6.443	0.023	-0.139	0.014	0.113
GB47507	histone H2A-like	6.020	-0.633	0.279	-0.209	-0.145
GB45458	UDP-glucose 6-dehydrogenase-like isoform X2	5.706	-0.039	-0.053	-0.334	-0.210
GB41782	LOW QUALITY PROTEIN: glycine dehydrogenase [decarboxylating], mitochondrial-like	5.448	0.185	-0.253	-0.007	-0.101
GB54426	transmembrane protein 205-like	5.185	-0.051	0.172	0.086	-0.082
GB43591	uncharacterized protein LOC408443	4.927	0.436	0.220	-0.324	0.107
GB46298	endocuticle structural glycoprotein SgAbd-8-like isoform X2	3.340	0.204	-0.688	-0.083	0.161

Supplementary Table 30: List of all the genes in Module 5, ranked by their within-module connectivity, k. The latter four columns give the Log_2 fold-change in expression in response to queen pheromone in each of the four species.

Gene	Name	k	am_fc	bt_fc	lf_fc	ln_fc
GB51221	ubiquitin-protein ligase E3A isoform X2	24.893	0.405	0.058	0.024	0.057
GB53171	transcription elongation factor B polypeptide 2	23.618	0.197	0.012	-0.006	0.081
GB55522	RING finger protein 11-like isoform X2	21.612	-0.031	0.029	0.069	0.042
GB50246	membrane-associated protein Hem	20.447	0.284	-0.026	0.009	0.113
GB55382	putative oxidoreductase GLYR1 homolog	20.162	-0.029	-0.038	-0.016	-0.004
GB50020	signal transducer and activator of transcription 5B	19.694	0.154	-0.056	-0.008	0.055
GB47341	protein disulfide-isomerase TMX3-like isoform X2	19.377	0.234	0.067	-0.014	0.144
GB54119	vacuolar protein sorting-associated protein 11 homolog isoform X1	19.075	0.300	-0.050	0.016	0.094
GB52529	F-actin-capping protein subunit beta-like	18.873	0.310	-0.022	0.009	0.052
GB44934	nuclear cap-binding protein subunit 1 isoformX1	18.351	0.057	0.062	-0.006	0.191
GB41553	Golgi phosphoprotein 3 homolog rotini-like isoform X1	18.293	0.455	-0.049	0.055	0.014
GB44725	uncharacterized protein LOC552067	18.171	0.733	-0.024	-0.049	0.115
GB52497	mitochondrial import receptor subunit TOM20 homolog	18.004	0.255	-0.005	-0.050	0.097
GB43692	CCR4-NOT transcription complex subunit 11-like	17.936	0.238	-0.087	-0.023	0.158
GB50194	nuclear inhibitor of protein phosphatase 1 isoform X2	17.724	0.089	0.143	0.028	0.053
GB54129	protein LSM12 homolog A-like isoform X2	17.631	0.126	0.005	0.008	0.095
GB43194	syntaxin-8 isoform 2	17.620	0.081	0.044	-0.001	0.149
GB49114	nucleolar complex protein 4 homolog B-like isoform X1	17.503	0.409	0.011	0.046	0.127
GB45042	neuroguidin-A-like	17.295	-0.087	-0.013	0.082	0.122
GB44445	5'-3' exoribonuclease 2 homolog	17.286	0.145	0.003	-0.051	0.191
GB55624	leucine-rich repeat and immunoglobulin-like domain-containing nogo receptor-interacting protein 2-like isoform X2	17.131	0.172	0.094	0.038	0.060
GB42559	oxysterol-binding protein 1 isoform X5	17.015	0.220	0.025	0.029	-0.133
GB45368	serine/threonine-protein phosphatase 2A catalytic subunit alpha isoform isoform 1	16.896	0.267	0.049	0.047	0.152
GB44299	TOM1-like protein 2-like isoform X3	16.833	0.060	-0.006	0.038	0.016
GB53245	UBX domain-containing protein 6	16.807	0.149	-0.049	-0.032	0.169
GB41069	pyridoxal-dependent decarboxylase domain-containing protein 1-like isoform X2	16.772	0.543	-0.062	-0.043	0.034
GB53770	39S ribosomal protein L51, mitochondrial	16.717	0.071	-0.012	0.032	0.160
GB54381	lisH domain-containing protein C1711.05-like isoform X1	16.624	0.036	0.081	0.698	0.057
GB44580	nucleolysin TIAR	16.427	0.181	0.040	-0.018	0.616
GB42961	glutathione S-transferase theta-4	16.398	0.274	-0.152	-0.100	0.119
GB44067	probable phospholipid-transporting ATPase IIB-like	16.331	0.081	-0.009	0.027	0.212
GB46465	alpha/beta hydrolase domain-containing protein 13-like isoform X1	16.287	0.043	0.102	-0.073	0.107
GB52655	signal transducing adapter molecule 1	16.014	0.049	0.011	-0.008	0.041
GB50471	cation-independent mannose-6-phosphate receptor isoform X2	15.866	0.186	-0.139	-0.087	0.028
GB49399	COP9 signalosome complex subunit 4 isoform X2	15.855	0.163	0.109	-0.059	0.190
GB46766	F-actin-capping protein subunit alpha-like	15.851	0.275	0.022	-0.022	0.128
GB51186	probable Ufm1-specific protease 2-like isoform X1	15.841	0.118	-0.044	0.075	0.130
GB50701	pleckstrin homology domain-containing family M member 1-like	15.645	0.093	-0.119	-0.355	0.036
GB40598	LOW QUALITY PROTEIN: bumetanide-sensitive sodium-(potassium)-chloride cotransporter	15.494	0.051	0.061	0.016	0.025
GB41688	cullin-1-like isoformX1	15.486	0.117	0.111	0.029	0.193
GB40451	alanine aminotransferase 2-like	15.330	0.268	-0.098	-0.081	0.136
GB43438	WD repeat-containing protein 89-like	15.321	0.238	0.105	-0.040	0.147
GB55444	serine/threonine-protein phosphatase PP1-beta-like isoform 1	15.311	0.120	0.040	-0.040	0.117
GB50802	putative tyrosine-protein kinase Wsck-like	15.306	-0.197	-0.035	0.017	0.004
GB55431	ubiquitin-conjugating enzyme E2 R2-like isoform X1	15.131	0.223	0.120	-0.095	0.104
GB42155	craniofacial development protein 1 isoform X1	15.106	0.113	0.089	-0.005	0.228
GB41063	protein ariadne-1-like isoform X1	15.035	-0.094	-0.090	0.023	0.164
GB45350	zinc finger CCCH domain-containing protein 10-like isoform X1	15.001	0.109	0.050	0.128	0.108
GB44037	ATP-dependent RNA helicase Ddx1 isoform 1	14.829	0.273	0.044	0.101	-0.298

(continued)

Gene	Name	k	am_fc	bt_fc	lf_fc	ln_fc
GB51197	protein TSSC1-like	14.734	0.235	-0.143	-0.083	0.267
GB42241	membrane-associated guanylate kinase, WW and PDZ domain-containing protein 2 isoform X6	14.712	0.391	-0.043	-0.017	0.089
GB49353	lamin Dm0-like isoform X2	14.657	0.167	0.055	0.356	-0.072
GB44496	probable serine incorporator isoformX1	14.489	0.376	-0.016	0.024	0.127
GB40807	uncharacterized protein LOC724585	14.462	0.322	0.031	-0.127	0.105
102655399	proteasome assembly chaperone 4-like	14.418	0.330	0.065	0.042	0.242
GB48926	NEDD8-conjugating enzyme Ubc12-like	14.367	0.408	0.075	0.049	0.115
GB40888	CAAX prenyl protease 1 homolog isoform X3	14.233	0.378	-0.070	-0.056	0.034
102656934	stress-induced-phosphoprotein 1-like	13.987	0.294	0.116	-0.310	0.253
GB40727	CDP-diacylglycerol–inositol 3-phosphatidyltransferase-like	13.967	0.347	-0.117	0.036	0.102
GB43905	stomatin-like protein 2, mitochondrial-like isoform 1	13.956	0.220	0.098	0.063	0.157
GB48404	transmembrane and coiled-coil domains protein 1-like isoform X1	13.926	0.144	0.153	-0.078	0.283
GB42952	ADP-ribosylation factor-like protein 1-like isoform 1	13.875	0.453	-0.072	-0.034	0.176
GB43554	adapter molecule Crk-like isoform X2	13.846	0.151	0.018	-0.017	0.050
GB55295	bis(5'-nucleosyl)-tetraphosphatase [asymmetrical] isoform X4	13.767	0.517	-0.030	-0.057	0.135
GB53438	histone deacetylase Rpd3 isoform 1	13.656	0.185	0.049	-0.119	0.238
GB52745	thioredoxin domain-containing protein 15-like isoform X3	13.599	0.168	0.089	-0.027	0.112
GB47463	guanine nucleotide-binding protein G(i) subunit alpha-like	13.578	0.505	0.019	-0.088	0.143
GB50980	acyl-protein thioesterase 1-like	13.541	0.552	-0.003	0.056	0.109
GB55852	E3 ubiquitin-protein ligase RNF185-like isoform 1	13.519	0.273	-0.164	-0.043	0.084
GB44896	coiled-coil domain-containing protein 132-like	13.404	0.392	-0.016	-0.086	0.104
GB55090	probable ATP-dependent RNA helicase DDX56 isoform 1	13.300	0.410	0.071	-0.064	0.236
GB40309	peroxisomal biogenesis factor 19-like isoform X1	13.239	-0.043	-0.119	0.017	0.179
GB40895	WD repeat domain-containing protein 83-like isoform 1	13.237	0.204	0.048	-0.087	0.137
GB53380	uncharacterized protein KIAA2013 homolog	13.119	0.225	-0.073	-0.027	0.106
GB42694	AP-1 complex subunit mu-1-like isoform 1	13.031	0.255	0.094	0.120	0.028
GB43203	niemann-Pick C1 protein-like isoform X3	12.974	0.394	0.002	-0.064	0.035
724366	ras-related protein Rap-2a	12.910	0.089	-0.018	0.011	0.626
GB42845	adenylate kinase	12.908	0.136	-0.143	-0.067	0.049
GB42266	UPF0472 protein C16orf72 homolog	12.907	0.044	-0.187	-0.104	0.096
GB52191	mannan-binding lectin serine protease 1	12.893	0.244	0.086	-0.252	0.007
GB42944	ras-related protein Rab-10 isoformX2	12.807	0.282	0.057	-0.043	0.213
GB40289	ATP-dependent (S)-NAD(P)H-hydrate dehydratase-like isoform X1	12.786	0.498	0.006	-0.226	0.084
GB50075	probable palmitoyltransferase ZDHHC16-like isoform X1	12.762	-0.105	0.154	-0.051	0.225
GB41035	ubiquitin carboxyl-terminal hydrolase 31-like isoform 1	12.686	-0.220	0.188	-0.040	0.145
GB42022	ubiquitin carboxyl-terminal hydrolase 34-like	12.645	0.104	-0.118	1.001	0.034
GB43184	iron/zinc purple acid phosphatase-like protein-like	12.629	0.451	0.097	0.427	0.171
GB45944	ras-related protein Rab-14 isoform 1	12.588	0.066	-0.026	-0.010	0.164
GB52762	ceramide-1-phosphate transfer protein-like isoform X3	12.378	0.456	0.118	0.145	0.200
GB42416	solute carrier family 35 member C2-like isoform X3	12.367	0.420	0.196	-0.059	0.017
GB46768	uncharacterized MFS-type transporter C09D4.1-like isoform X6	12.339	0.179	0.006	-0.063	0.052
GB42940	protein lifeguard 1-like isoform X3	12.324	0.246	-0.104	0.424	0.055
GB41046	ubiquitin-conjugating enzyme E2 Q2-like isoform X1	12.309	0.148	0.066	-0.040	0.074
GB49529	proliferation-associated protein 2G4-like	12.301	0.585	0.074	-0.086	-0.454
GB40339	iodotyrosine dehalogenase 1-like isoformX2	12.298	0.215	0.209	-0.058	0.031
GB55478	probable E3 ubiquitin-protein ligase makorin-1 isoform X2	12.284	0.047	0.139	0.009	0.143
GB54251	DCN1-like protein 1-like isoform X2	12.271	-0.175	-0.053	-0.061	0.081
GB50219	DE-cadherin-like isoform X5	12.185	0.179	0.089	-0.734	-0.458
GB43103	transmembrane emp24 domain-containing protein 5	11.984	0.503	0.140	0.012	-0.229
GB45558	ras-related protein Rac1 isoform 1	11.982	0.171	-0.067	-0.015	-0.001
GB51358	TIP41-like protein-like isoform X2	11.943	0.216	0.203	0.013	0.185
GB52754	probable dimethyladenosine transferase-like	11.813	0.319	0.093	-0.178	0.172
102654186	fizzy-related protein homolog	11.797	0.475	-0.106	-0.072	0.062
GB44869	soluble NSF attachment protein isoform X2	11.739	0.433	-0.146	0.037	0.164
GB44773	neural Wiskott-Aldrich syndrome protein isoform X3	11.684	0.467	0.129	-0.113	0.116
GB44181	E3 ubiquitin-protein ligase RNF8-like isoform X2	11.683	0.695	0.052	0.005	0.393
GB49107	uncharacterized threonine-rich GPI-anchored glycoprotein PJ4664.02	11.682	0.007	0.021	-0.028	0.142

(continued)

Gene	Name	k	am_fc	bt_fc	lf_fc	ln_fc
GB55581	membrane-associated progesterone receptor component 1-like isoform 2	11.634	0.513	-0.114	-0.044	-0.240
GB54789	GMP synthase [glutamine-hydrolyzing]	11.615	0.371	0.024	-0.049	0.035
GB50475	E3 ubiquitin-protein ligase RAD18-like	11.542	0.146	0.175	-0.006	0.314
GB47753	transmembrane protein 208-like	11.527	0.184	0.097	-0.031	-0.084
GB53819	uncharacterized protein LOC100577293	11.515	0.048	0.134	1.679	0.222
GB46586	protein phosphatase 1H-like	11.465	0.071	-0.041	1.494	0.289
GB43135	RAC serine/threonine-protein kinase	11.443	0.244	0.000	0.082	0.175
GB46333	glycoprotein-N-acetylgalactosamine 3-beta-galactosyltransferase 1-like	11.435	0.291	0.113	0.043	0.130
GB40911	E3 SUMO-protein ligase PIAS3 isoform X2	11.430	0.097	0.117	-0.027	0.115
GB52059	eukaryotic translation initiation factor 4H-like isoform X1	11.424	0.371	0.056	4.709	0.087
GB46567	eukaryotic translation initiation factor 1A, X-chromosomal	11.355	0.330	-0.005	-0.323	0.399
GB53043	ATP-binding cassette sub-family G member 4 isoform X2	11.317	0.188	-0.187	0.176	0.103
GB43843	thioredoxin-related transmembrane protein 2 homolog	11.208	-0.054	0.188	-0.292	0.267
GB49351	solute carrier family 35 member E2-like isoform X1	11.157	0.048	0.072	0.101	0.150
GB40126	uncharacterized protein LOC410456 isoform X1	11.136	-0.329	-0.110	-0.107	0.285
GB53284	proto-oncogene tyrosine-protein kinase receptor Ret-like isoform X3	11.119	0.530	-0.029	-0.112	-0.467
GB42488	vesicle transport protein GOT1B-like isoform 2	11.102	0.583	0.005	-0.179	0.085
GB54950	uncharacterized protein LOC726431 isoform X1	11.099	-0.094	-0.129	-1.059	0.189
GB42797	protein takeout-like	11.035	1.302	0.150	0.440	0.154
GB47542	eukaryotic translation initiation factor 3 subunit J isoform 1	10.986	0.399	-0.016	-0.128	0.197
GB45071	G-protein-signaling modulator 2	10.925	0.298	-0.118	0.070	0.178
GB54449	DDB1- and CUL4-associated factor 7-like	10.845	0.584	-0.111	0.058	0.232
GB42106	probable histone-binding protein Caf1	10.820	0.492	0.203	-0.111	0.129
GB41158	toll-interacting protein	10.818	0.507	-0.055	0.290	0.146
GB44621	serine/threonine-protein phosphatase 5	10.702	-0.017	-0.050	-0.919	0.094
GB42084	probable G-protein coupled receptor Mth-like 1-like isoform X2	10.547	0.548	-0.098	0.064	-0.049
GB53627	zinc transporter ZIP9-B-like	10.490	0.634	-0.082	-0.015	0.195
GB42313	leishmanolysin-like peptidase	10.383	0.458	-0.084	-0.080	0.337
GB47391	nuclear pore complex protein Nup107	10.264	-0.072	0.162	-0.098	0.142
GB40708	tetraspanin 6 isoform X1	10.222	0.564	-0.049	0.517	0.204
GB44383	histone acetyltransferase Tip60	10.070	0.059	0.143	-0.025	0.316
GB41467	growth arrest-specific protein 2-like isoform X2	10.055	0.006	-0.145	-0.195	0.129
GB51551	myophilin	9.650	0.437	0.104	-0.037	0.176
GB48646	ADP-ribosylation factor-like protein 2-like isoform 1	9.429	0.389	-0.073	-0.058	0.153
GB54084	histone H3.3-like isoform 2	9.328	0.560	0.173	-0.262	0.145
GB47231	exportin-5	9.204	0.245	0.113	-0.328	0.030
GB54172	sodium-independent sulfate anion transporter-like isoformX1	9.122	0.664	0.088	-0.024	0.061
GB45313	uncharacterized protein LOC552058 isoform X4	9.063	-0.007	-0.339	0.033	0.245
GB42817	transmembrane protein 63B-like isoform X3	9.063	0.383	0.027	0.083	0.029
GB42168	receptor expression-enhancing protein 5-like isoform X2	8.991	0.623	-0.092	-0.306	-0.430
GB55219	uncharacterized protein LOC724286 isoform X2	8.564	0.432	-0.154	0.081	0.111
GB54052	72 kDa inositol polyphosphate 5-phosphatase-like isoform X1	8.323	0.039	-0.021	0.664	-0.148
GB40758	icarapin-like	7.942	0.428	0.070	-0.244	0.108
GB41793	cytochrome c-type heme lyase-like	7.878	0.039	-0.123	0.026	0.265
GB43617	uncharacterized membrane protein DDB_G0293934-like isoform X1	7.441	0.659	-0.002	-0.004	0.734
GB46795	papilin-like isoform X7	7.090	0.205	-0.096	0.114	0.029
409791	cAMP-dependent protein kinase catalytic subunit isoform 1	6.924	0.156	-0.280	-0.038	0.161
GB40838	endoglucanase 15-like	6.911	0.397	-0.110	0.326	0.203
GB45983	venom acid phosphatase Acph-1-like isoform X3	6.410	0.403	-0.022	-0.096	-0.015
GB44192	leucine-rich repeat-containing protein 26-like	6.023	0.487	-0.381	-0.109	0.118
GB45973	aromatic-L-amino-acid decarboxylase	5.798	-0.057	-0.279	0.037	0.174
GB45673	alpha-N-acetylgalactosaminidase-like	5.000	0.683	0.027	0.072	0.277
GB42272	Usher syndrome type-1G protein homolog isoform X3	4.748	0.472	0.009	0.033	0.285
GB40566	cuticular protein 6 precursor	4.626	0.972	-0.081	-0.430	0.294

Supplementary Table 31: List of all the genes in Module 6, ranked by their within-module connectivity, k. The latter four columns give the Log_2 fold-change in expression in response to queen pheromone in each of the four species.

Gene	Name	k	am_fc	bt_fc	lf_fc	ln_fc
GB55009	another transcription unit protein	19.372	-0.010	0.116	0.001	0.168
GB46605	E3 ubiquitin-protein ligase CHIP-like isoform X2	19.333	-0.061	0.084	0.052	0.163
GB42203	putative adenosylhomocysteinase 3-like isoform X1	17.466	-0.112	0.093	0.075	0.141
GB49412	transcription factor IIIB 90 kDa subunit-like isoformX2	16.941	-0.299	0.052	0.114	0.172
GB19050	glutathione S-transferase C-terminal domain-containing protein	16.582	-0.074	0.070	0.064	0.240
GB52651	diphthine–ammonia ligase-like isoform X4	16.258	-0.020	0.105	0.064	0.174
GB54650	protein PFC0760c-like isoform X1	16.232	-0.122	-0.183	0.046	0.173
GB44792	PX domain-containing protein kinase-like protein-like isoform X3	15.895	-0.145	-0.075	0.114	0.206
GB41134	Golgi SNAP receptor complex member 1 isoform 1	15.894	0.058	0.137	0.030	0.170
GB44828	uncharacterized protein LOC550822	15.166	-0.017	-0.111	0.045	0.232
GB55940	gamma-tubulin complex component 3-like	15.160	-0.166	-0.006	1.047	0.171
GB47667	dnaJ homolog subfamily C member 16-like	15.072	-0.180	0.053	0.070	0.285
GB49528	exocyst complex component 6B isoform 1	14.847	-0.164	0.050	0.120	0.266
GB52340	uncharacterized protein LOC409246	14.741	-0.285	-0.005	0.036	0.111
GB42246	rho GTPase-activating protein 190 isoform X3	14.584	-0.203	0.042	0.287	0.163
725733	uncharacterized protein LOC725733 isoform X3	14.438	0.022	-0.005	0.088	0.301
GB47841	dnaJ homolog dnj-5-like	14.272	-0.123	0.060	0.085	0.174
GB44615	extended synaptotagmin-1 isoform X1	14.247	0.112	-0.025	0.093	-0.047
GB46907	zinc finger CCHC domain-containing protein 8 homolog	14.234	-0.189	0.035	0.244	0.159
GB40574	serine palmitoyltransferase 1	13.721	-0.390	-0.093	0.016	0.160
GB42753	ras-related protein Rab-7a-like	13.634	0.201	-0.066	0.093	0.116
GB41692	DIS3-like exonuclease 2-like isoform X1	13.601	-0.194	0.112	0.072	-1.026
GB41292	metallophosphoesterase 1 homolog, transcript variant X2	13.584	-0.382	0.050	0.127	0.188
GB49413	COP9 signalosome complex subunit 5 isoform X4	13.559	0.162	0.006	0.030	0.130
GB41738	uncharacterized protein LOC410546 isoform X1	13.211	-0.139	0.254	0.120	0.130
GB45296	amyloid protein-binding protein 2	12.959	-0.069	0.045	-0.023	-0.020
GB47837	putative high mobility group protein 1-like 10-like isoform X1	12.943	0.049	-0.030	0.161	0.244
GB50816	ankyrin repeat domain-containing protein 54-like	12.746	-0.181	0.167	-0.156	0.084
GB42375	solute carrier family 25 member 46-like isoform 1	12.713	0.102	-0.061	0.088	0.193
GB53222	ER membrane protein complex subunit 7-like	12.674	-0.014	-0.054	0.028	0.146
GB54588	transport and Golgi organization protein 11	12.579	-0.073	0.081	0.021	0.181
GB54210	lipoma-preferred partner homolog isoform X4	12.554	-0.002	0.121	0.148	0.109
GB44880	DENN domain-containing protein 1A-like isoformX1	12.490	-0.079	0.068	0.402	0.147
102653759	zinc finger CCHC domain-containing protein 4-like	12.272	-0.520	0.145	-0.183	0.351
GB49366	RNA polymerase II-associated protein 1-like	12.231	-0.111	0.134	0.016	0.102
GB54971	BTB/POZ domain-containing protein 9 isoform X1	12.149	0.013	-0.035	0.109	0.135
412247	putative ribosomal RNA methyltransferase CG11447-like	12.127	-0.068	0.040	-0.002	0.196
GB50128	heparanase-like isoform X3	11.916	-0.102	0.032	0.093	0.205
GB42689	uncharacterized protein LOC100577561	11.681	-0.469	0.129	0.007	0.256
GB46728	leucine-rich repeat-containing protein 49-like	11.540	-0.082	-0.175	0.072	0.080
GB55103	max-like protein X-like	11.450	0.240	0.081	-1.312	0.141
GB55817	uncharacterized protein LOC100577885	11.403	-0.278	0.208	-0.021	0.412
GB45828	uncharacterized J domain-containing protein C4H3.01-like	11.251	-0.324	-0.052	0.031	0.165
GB45314	cGMP-dependent 3',5'-cyclic phosphodiesterase-like isoform 1	11.244	-0.259	0.036	-0.008	0.105
GB50168	uncharacterized protein LOC100576411	11.223	-0.119	0.049	0.325	0.115
102656107	immunoglobulin-binding protein 1-like	11.024	-0.073	-0.016	0.070	0.259
GB55918	tRNA-splicing endonuclease subunit Sen34-like	10.938	-0.614	0.152	0.081	0.359
GB45820	protoheme IX farnesyltransferase, mitochondrial	10.936	-0.329	-0.063	-0.485	0.261
GB43081	FAD synthase-like isoform X2	10.853	-0.220	-0.263	0.538	0.383
GB40107	ATPase WRNIP1-like isoform X2	10.771	-0.068	-0.128	0.065	0.274
GB55997	low density lipoprotein receptor adapter protein 1-B-like isoform X1	10.749	-0.106	-0.163	0.038	0.106
102656249	protein HEXIM1-like	10.728	-0.102	-0.022	0.093	0.126
GB45215	peroxisomal N(1)-acetyl-spermine/spermidine oxidase-like	10.720	-0.225	0.255	0.551	0.243
GB40874	GTP-binding protein 1-like isoform X2	10.705	-0.072	-0.235	0.094	0.170
GB55259	caskin-1-like	10.641	-0.266	0.018	-0.001	0.136

(continued)

Gene	Name	k	am_fc	bt_fc	lf_fc	ln_fc
GB47328	tRNA selenocysteine 1-associated protein 1-like isoform X1	10.631	0.124	0.061	-0.024	0.135
GB41373	protein germ cell-less isoform X2	10.599	0.203	-0.016	0.152	0.230
413672	hexosaminidase D-like isoform 1	10.585	0.192	0.097	0.028	0.181
GB43421	sprouty-related, EVH1 domain-containing protein 2 isoform X4	10.564	-0.169	0.187	0.209	0.135
GB55427	uncharacterized protein LOC100577661 isoform 1	10.557	-0.270	-0.162	-0.035	0.303
GB49081	60 kDa SS-A/Ro ribonucleoprotein-like	10.552	-0.101	-0.058	-0.059	0.175
GB44319	glycerate kinase-like	10.551	-0.425	-0.131	-1.223	0.246
GB40020	ras-related protein Rab-39A isoform X1	10.495	-0.086	-0.055	0.167	0.366
GB48996	putative protein arginine N-methyltransferase 10-like isoform X1	10.471	-0.426	-0.087	0.116	0.166
GB41067	dual specificity mitogen-activated protein kinase kinase dSOR1	10.427	0.081	0.258	0.139	0.244
GB52007	DCN1-like protein 4-like isoform X1	10.392	-0.020	0.090	0.022	0.128
GB55558	biogenesis of lysosome-related organelles complex 1 subunit 3-like isoform 1	10.325	-0.009	-0.033	0.140	0.304
GB42960	protein CIP2A-like isoform X1	10.182	-0.955	0.059	-0.007	0.258
GB44555	uncharacterized protein LOC413653 isoformX1	10.151	-0.041	-0.067	0.066	0.168
GB41398	MATH and LRR domain-containing protein PFE0570w-like isoform X1	10.107	-0.305	-0.126	0.028	0.507
GB41656	putative leucine-rich repeat-containing protein DDB_G0290503-like	10.090	-0.761	0.141	0.019	0.259
GB51592	COP9 signalosome complex subunit 2-like	9.985	0.167	0.068	-0.045	0.496
GB46813	unconventional myosin-Ie-like	9.978	-0.024	-1.577	0.041	0.052
GB54480	probable RISC-loading complex subunit BRAFLDRAFT_242885	9.966	-0.663	0.101	-0.078	0.196
GB51633	protein HIRA homolog	9.920	0.380	0.204	0.084	0.194
GB54677	U3 small nucleolar ribonucleoprotein protein IMP3-like	9.883	0.018	-0.076	0.081	0.287
GB43483	golgin-84	9.846	-0.320	0.192	0.123	0.141
GB46387	zinc finger protein 511-like	9.697	-0.132	-0.115	0.270	0.208
GB49383	CUE domain-containing protein 2-like	9.622	0.405	-0.098	0.100	0.354
GB55269	protein PAT1 homolog 1	9.616	-0.350	0.053	0.386	-0.265
GB40652	protein ST7 homolog isoform 1	9.587	-0.101	0.247	0.087	0.160
GB55873	proteasome inhibitor PI31 subunit-like isoform X1	9.569	0.417	-0.044	0.067	0.239
GB44694	ras-related protein Rab-43	9.546	0.030	0.008	-0.017	1.098
GB42964	beta-1,3-glucosyltransferase-like isoform 2	9.390	0.185	-0.001	0.112	0.292
GB43872	coiled-coil domain-containing protein 50-like	9.373	-0.020	0.008	0.108	0.235
GB55872	DNA repair protein XRCC1-like	9.360	-0.376	-0.031	-0.019	0.305
GB50102	polyadenylate-binding protein-interacting protein 2 isoform X2	9.354	0.216	-0.022	0.210	0.334
GB53962	protein 60A	9.274	-0.313	-0.063	0.050	0.195
GB55722	G/T mismatch-specific thymine DNA glycosylase-like	9.254	0.231	0.263	-0.025	0.201
GB41824	solute carrier family 25 member 36-A-like isoform X4	9.233	-0.483	-0.034	0.058	0.387
GB48579	zinc finger protein 277-like	9.137	-0.535	0.266	0.052	0.141
GB41602	facilitated trehalose transporter Tret1-like isoform X6	9.111	0.153	-0.024	0.231	0.090
GB46023	protein PF14_0175-like	9.107	-0.145	0.075	-0.058	0.160
GB46502	uncharacterized protein LOC724680	9.063	0.258	-0.024	0.045	-0.401
GB49246	protein tincar isoform X5	9.047	-0.163	0.228	1.464	0.210
GB53932	protein phosphatase 1L-like isoform X2	8.915	-0.208	-0.114	0.854	0.134
GB55535	E3 ubiquitin-protein ligase Siah1 isoform X1	8.906	0.057	-0.119	0.022	0.317
GB45679	polycomb protein Asx-like isoform X2	8.872	-0.442	0.148	0.276	0.599
GB55544	endoplasmic reticulum oxidoreductin-1-like	8.869	0.349	-0.038	-0.546	0.167
GB49101	ras-related protein Rab-9A-like isoform X3	8.862	-0.021	-0.141	0.284	0.235
GB54838	post-GPI attachment to proteins factor 2-like isoform X1	8.850	-0.208	-0.065	0.179	0.204
GB53648	tRNA pseudouridine synthase-like 1-like isoform X2	8.838	-0.167	0.082	0.123	0.136
102654261	uncharacterized protein C24H6.02c-like	8.815	0.200	0.035	0.095	0.241
GB53315	uncharacterized protein LOC726215	8.771	-0.214	-0.154	-0.466	0.179
GB54386	cyclin-G2 isoform X1	8.735	-0.123	0.019	0.136	0.155
GB49702	mitoferrin-1 isoformX2	8.583	-0.460	-0.007	-0.076	0.253
GB40705	N(G),N(G)-dimethylarginine dimethylaminohydrolase 1-like isoform X3	8.570	-0.280	-0.241	0.138	0.245
GB43479	cyclin-C	8.543	0.419	0.204	0.030	0.359

(continued)

Gene	Name	k	am_fc	bt_fc	lf_fc	ln_fc
GB49429	calcium channel flower-like isoform X1	8.541	0.595	0.062	0.197	0.229
GB48358	chromosome transmission fidelity protein 8 homolog	8.532	0.155	0.213	0.089	0.451
GB53702	uncharacterized protein C45G9.7-like	8.529	-0.880	0.064	-0.023	0.176
GB44365	germ cell-expressed bHLH-PAS-like protein, transcript variant X3	8.524	-0.357	-0.149	0.084	0.139
GB55564	transcriptional regulator ATRX homolog isoform X2	8.513	0.166	0.076	0.081	0.223
GB47249	E3 ubiquitin-protein ligase Smurf1 isoform X2	8.447	0.328	0.070	0.214	0.206
GB54842	arginine–tRNA ligase, cytoplasmic	8.443	-0.166	0.070	-0.090	0.162
GB51498	myeloid differentiation primary response protein MyD88-A isoform X1	8.412	-0.685	-0.037	-0.043	0.119
GB46148	guanine nucleotide-binding protein subunit alpha homolog	8.391	-0.570	0.189	-0.011	0.259
GB50834	peroxisomal membrane protein 11B-like isoform 2	8.370	-0.342	-0.018	0.062	0.224
GB43388	transcription initiation factor IIA subunit 2	8.153	0.375	-0.018	0.093	0.269
GB44404	retinol dehydrogenase 13-like	8.146	0.466	0.089	-1.579	0.234
GB45142	RB1-inducible coiled-coil protein 1 isoform X1	8.108	-1.007	-0.027	0.112	0.211
GB46745	general transcription factor 3C polypeptide 3-like	8.092	0.220	0.094	1.063	0.158
GB50255	RWD domain-containing protein 1-like isoform X1	8.092	0.125	-0.045	0.545	0.315
GB48642	uncharacterized protein LOC100577967	8.014	-0.657	-0.327	0.073	0.264
GB51251	ADP-ribosylation factor-like protein 8B-A-like isoform X1	8.011	0.457	0.073	0.128	0.246
102653960	magnesium-dependent phosphatase 1-like	7.935	-0.265	-0.150	-0.068	0.244
GB44697	probable serine/threonine-protein kinase DDB_G0283337-like isoform X2	7.879	-0.430	0.420	0.156	0.133
GB55038	protein UXT homolog	7.873	0.303	-0.047	0.025	0.264
GB54108	dual specificity protein phosphatase 3-like isoform X2	7.872	0.034	-0.042	0.081	0.177
GB42078	uncharacterized protein LOC725568	7.845	0.398	0.030	0.246	0.373
GB48617	uncharacterized protein C15orf41 homolog isoform X1	7.839	0.165	0.199	0.042	0.287
100577724	ELMO domain-containing protein 2-like isoform X2	7.791	-0.128	0.077	0.180	0.181
GB55523	LOW QUALITY PROTEIN: leucine-rich repeats and immunoglobulin-like domains 3	7.790	0.243	0.089	0.168	0.128
GB56016	39S ribosomal protein L30, mitochondrial	7.755	0.014	-0.072	0.122	0.199
GB55191	uncharacterized protein LOC100576289	7.708	-1.045	0.218	0.206	0.460
102656444	protein PFC0760c-like isoform X1	7.682	-1.306	0.032	0.120	0.188
GB46146	sodium/potassium-transporting ATPase subunit beta-2	7.675	0.510	0.060	0.239	0.329
GB45376	putative peptidyl-prolyl cis-trans isomerase dodo	7.613	0.375	0.084	0.132	0.281
GB55587	OTU domain-containing protein 7B-like isoform X2	7.609	0.030	-0.055	0.138	0.324
GB48809	proline-, glutamic acid- and leucine-rich protein 1-like	7.580	0.061	0.000	-0.867	0.149
GB41208	cell division control protein 45 homolog isoform X2	7.519	0.096	0.084	0.045	0.296
GB49178	OTU domain-containing protein 6B-like	7.517	-0.095	0.017	0.038	1.506
GB50984	sorting nexin-17 isoform X2	7.516	-0.455	-0.228	0.096	0.074
102656287	transmembrane protein 216-like	7.509	0.191	0.040	0.072	-0.002
GB41970	ras-like protein 2-like isoform X1	7.434	-0.035	-0.126	0.071	0.293
GB51623	adenylate kinase isoenzyme 6 isoform X2	7.399	0.047	0.022	0.108	0.088
GB52210	DDB1- and CUL4-associated factor 12-like isoform X3	7.313	-0.176	-0.075	0.213	0.171
102655090	uncharacterized protein LOC102655090	7.265	-0.651	0.208	0.068	-0.067
GB49727	prostaglandin E2 receptor EP4 subtype-like isoform X3	7.216	-0.460	-0.175	-0.319	0.405
GB54267	serendipity locus protein H-1-like	6.904	0.332	-0.081	0.174	0.386
GB47475	protein lethal(2)essential for life-like isoform 1	6.296	0.258	0.158	0.586	0.305
GB50748	PAX3- and PAX7-binding protein 1-like	5.296	-0.136	0.032	0.039	-0.217
GB49188	intraflagellar transport protein 80 homolog isoform X3	5.170	-0.161	-0.536	0.944	0.260
GB40147	fez family zinc finger protein 1-like	2.799	-0.301	0.302	0.669	0.378

Supplementary Table 32: List of all the genes in Module 7, ranked by their within-module connectivity, k. The latter four columns give the Log_2 fold-change in expression in response to queen pheromone in each of the four species.

Gene	Name	k	am_fc	bt_fc	lf_fc	ln_fc
GB43212	ubiquitin-protein ligase E3C-like isoform X2	19.056	0.253	-0.095	0.045	-0.030
GB43857	importin-13 isoform X1	18.128	0.139	-0.002	0.038	-0.083
GB53778	huntingtin-like	18.038	0.067	-0.065	0.485	0.028
GB47281	kinesin heavy chain isoform 1	17.615	0.027	-0.072	-0.536	-0.293
GB40720	CCR4-NOT transcription complex subunit 3-like isoform X1	15.793	0.105	0.132	0.045	0.325
GB41872	ATP-binding cassette sub-family F member 3-like isoform X2	15.354	0.151	0.204	-0.758	-0.015
GB49120	raf homolog serine/threonine-protein kinase phl	14.819	-0.125	-0.002	0.197	-0.023
GB41128	ubiquitin thioesterase OTU1-like	14.324	0.198	0.035	-0.085	-0.128
GB50061	uncharacterized protein LOC410606	14.206	-0.023	-0.016	0.303	0.053
GB44884	splicing factor 3B subunit 3 isoform 1	14.126	0.301	0.035	0.068	-0.040
GB53220	ubiquitin carboxyl-terminal hydrolase 3-like isoform X1	14.004	0.107	0.035	0.097	-0.085
GB55770	TBC1 domain family member 9	13.728	0.003	0.004	0.125	0.110
GB46321	neurofibromin isoform X1	13.683	0.233	-0.168	1.222	-0.081
GB54557	arginine/serine-rich protein PNISR-like isoform X2	13.593	0.086	0.118	0.139	0.090
GB42838	RNA-binding protein 39-like isoform X5	13.592	-0.086	-0.024	0.144	0.087
GB43304	cadherin-87A-like isoform X1	13.523	0.041	0.056	0.688	0.042
GB42436	protein arginine N-methyltransferase 7-like isoform X2	13.502	0.046	-0.122	0.069	0.079
GB50214	uncharacterized protein LOC409502	13.342	0.193	0.049	0.344	-0.039
GB49152	TBC1 domain family member 24-like isoformX1	13.205	-0.103	0.004	0.053	0.035
GB43236	probable tRNA (uracil-O(2)-)-methyltransferase-like	13.093	-0.030	0.319	0.058	-0.012
GB53708	serine/threonine-protein kinase unc-51	13.071	0.253	0.010	-0.299	0.047
GB50725	vang-like protein 2-like isoform X2	13.069	0.174	0.011	0.176	0.075
GB54230	uncharacterized protein LOC551498	13.030	0.089	0.079	0.894	0.108
GB44899	trithorax group protein osa isoform X6	12.694	-0.215	-0.050	0.163	-0.139
GB44490	hornerin	12.687	0.080	-0.022	0.155	-0.011
GB47322	glycine-rich cell wall structural protein 1.8-like isoform X10	12.633	0.145	0.162	0.479	-0.050
GB43467	rho guanine nucleotide exchange factor 28-like isoform X8	12.586	0.096	-0.050	0.095	-0.047
GB54590	polyadenylate-binding protein 1-like isoform X2	12.504	0.483	0.157	0.168	-0.189
GB45972	neural-cadherin isoform X2	12.370	-0.147	0.002	1.381	-0.018
100576876	intracellular protein transport protein USO1-like isoform X1	12.314	-0.013	-0.063	0.149	-0.075
GB44422	uncharacterized protein LOC412543 isoform X3	12.033	0.602	-0.053	-0.010	0.054
GB40676	phosphatidylinositol 4-kinase beta-like	11.710	0.140	0.010	0.113	-0.009
GB54731	plasmolipin-like isoform 1	11.624	0.405	0.006	0.065	-0.007
GB44761	Rab GTPase activating protein 10 isoform X3	11.326	0.067	0.033	0.133	-0.049
GB50373	endophilin-A isoform X1	11.222	0.171	0.145	0.153	-0.072
102655673	mitochondrial import inner membrane translocase subunit Tim16-like	11.155	-0.085	0.064	0.251	0.086
GB49375	homeobox protein PKNOX2-like isoform X1	10.805	-0.137	0.057	0.149	0.005
GB41423	dosage compensation regulator isoform X3	10.679	0.068	0.015	0.111	-0.073
GB44419	protein peanut isoform X3	10.578	0.256	0.126	0.137	-0.221
GB43899	E3 ubiquitin-protein ligase MIB2-like isoform X2	10.574	0.042	0.163	0.091	0.016
GB44259	aryl hydrocarbon receptor nuclear translocator homolog isoform X3	10.557	0.127	0.110	0.236	0.021
GB52106	tolkin isoform X1	10.504	0.431	-0.203	0.107	-0.120
GB48337	protein crooked neck	10.417	0.020	0.122	0.038	0.082
GB49960	uncharacterized protein LOC100379261	10.137	0.178	-0.175	0.096	-0.029
GB55574	probable phospholipid-transporting ATPase VD-like, transcript variant X3	10.032	-0.154	0.083	0.199	0.049
GB44679	metastasis-associated protein MTA3 isoform X2	9.907	0.012	0.167	0.422	-0.044
GB51337	dedicator of cytokinesis protein 3-like isoform X2	9.891	-0.035	-0.086	0.248	-0.495
GB55162	uncharacterized protein LOC551144 isoform X1	9.834	0.158	0.015	0.187	0.016
GB53787	lysine-specific demethylase 3B-like isoform X4	9.831	-0.227	0.025	-0.137	-0.413
GB52510	putative uncharacterized protein DDB_G0277255 isoform X2	9.724	0.034	0.029	-0.053	-0.132
GB45414	nuclear hormone receptor FTZ-F1 beta isoform X1	9.714	-0.005	-0.131	0.249	0.037
GB50344	beta-1,4-mannosyltransferase egh	9.703	0.256	-0.114	0.225	0.100
GB52075	RNA-binding protein 45-like isoform X1	9.609	0.187	0.123	0.524	-0.095
GB48932	phosphoinositide 3-kinase regulatory subunit 4 isoform X2	9.558	0.003	-0.045	0.001	-0.052
GB51219	eye-specific diacylglycerol kinase isoform X2	9.488	-0.106	0.339	0.670	-0.012

(continued)

Gene	Name	k	am_fc	bt_fc	lf_fc	ln_fc
409956	uncharacterized protein LOC409956 isoform X2	9.483	0.056	-0.088	0.155	-0.301
GB49505	rho-related BTB domain-containing protein 1 isoform X3	9.476	0.057	-0.140	0.127	0.014
GB47816	splicing factor 1-like isoform X2	9.466	0.245	0.084	0.510	0.068
GB51841	multiple inositol polyphosphate phosphatase 1-like	9.400	0.064	0.018	0.229	-0.032
GB54551	slit homolog 3 protein-like isoform X2	9.333	0.328	0.048	0.351	0.065
GB55323	phosphatidylinositol 4-phosphate 5-kinase type-1 gamma isoform X8	9.312	0.232	-0.096	0.250	-0.014
GB44412	protein turtle homolog A-like	9.141	0.321	0.179	0.242	-0.162
GB49921	integrin alpha-PS2 isoform X1	9.125	0.071	-0.129	0.107	0.041
726252	titin-like isoform X2	9.120	0.156	-0.069	0.199	0.007
GB46271	protein BCL9 homolog isoform X1	9.051	-0.161	0.007	-1.649	-0.052
GB45593	zinc finger protein Helios-like	9.040	0.095	-0.029	0.155	-0.096
GB47028	enoyl-CoA hydratase domain-containing protein 3, mitochondrial-like	9.008	0.528	-0.063	0.135	-0.019
GB44534	spastin isoform X1	9.005	0.458	-0.043	0.059	-0.011
GB44779	tyrosine-protein phosphatase non-receptor type 4 isoform X2	8.953	0.014	0.151	0.033	0.046
GB51209	dentin sialophosphoprotein-like isoform X4	8.941	-0.423	-0.017	0.126	-0.104
102654691	protein translation factor SUI1 homolog	8.923	0.445	-0.012	0.647	0.031
102655836	tRNA (adenine(58)-N(1))-methyltransferase non-catalytic subunit TRM6-like	8.899	0.191	-0.021	0.034	-0.156
GB52185	uncharacterized protein LOC100578041 isoformX1	8.889	0.201	-0.183	0.204	0.004
GB41225	aquaporin AQPAn.G-like isoform X2	8.855	0.154	-0.351	0.178	0.494
GB52779	probable cation-transporting ATPase 13A3-like isoform X3	8.696	-0.240	0.097	0.058	-0.134
GB45955	beta-galactosidase-like isoform X2	8.669	0.582	0.065	0.215	-0.164
GB53592	SNF-related serine/threonine-protein kinase-like	8.654	0.290	0.108	0.264	0.519
GB52157	GRAM domain-containing protein 3-like isoform X2	8.647	0.292	-0.118	-0.801	0.065
GB47669	putative uncharacterized protein DDB_G0271606-like	8.568	0.220	-0.117	-0.130	0.099
GB40417	transmembrane protein 98	8.560	0.500	0.002	0.133	-1.490
GB49911	insulin receptor substrate 1 isoform X3	8.484	0.099	-0.173	0.375	-0.030
GB47918	netrin receptor UNC5C isoform X8	8.462	0.240	0.093	0.113	-0.124
GB53437	F-box only protein 32-like isoform X2	8.350	0.191	-0.165	0.295	0.054
GB46918	monocarboxylate transporter 13-like, transcript variant X2	8.342	0.428	0.106	0.223	0.014
GB42976	teneurin-a-like isoform X7	8.326	0.339	-0.050	0.997	0.306
409007	heterogeneous nuclear ribonucleoprotein K isoform X8	8.272	-0.060	-0.071	0.454	-0.336
GB50892	uncharacterized protein LOC100577980 isoform X3	8.196	0.103	-0.307	-0.137	-0.118
GB42979	ankyrin repeat and BTB/POZ domain-containing protein BTBD11-like isoform X3	8.191	0.305	-0.190	0.413	0.010
GB54219	BAG domain-containing protein Samui-like isoform X3	8.182	-0.184	0.411	0.282	-0.148
GB51674	26S proteasome non-ATPase regulatory subunit 10-like	8.110	0.230	0.028	0.010	-0.035
GB54395	uncharacterized protein LOC413385	8.102	0.090	0.071	0.017	-0.101
GB44968	metabotropic glutamate receptor 1	8.096	-0.284	0.151	0.214	-0.072
GB51744	uncharacterized protein LOC724439	8.092	0.811	-0.258	0.289	0.071
724450	uncharacterized protein LOC724450 isoform X3	7.965	-0.006	-0.038	0.222	-0.240
GB40162	chondroitin sulfate synthase 1-like isoform X2	7.953	0.194	0.212	0.226	-0.351
GB40907	putative ferric-chelate reductase 1 homolog isoform X3	7.935	0.122	0.079	0.079	-0.219
726866	uncharacterized protein LOC726866	7.923	0.277	0.070	0.243	-0.085
GB42196	thrombospondin type-1 domain-containing protein 4-like isoform X3	7.898	-0.154	-0.095	0.068	-0.019
GB43567	MAM and LDL-receptor class A domain-containing protein C10orf112-like	7.894	0.442	-0.019	0.223	-0.094
413366	homeobox protein SIX2-like isoform X4	7.890	-0.830	-0.101	0.291	-0.127
GB42035	myosin-I heavy chain isoform X2	7.882	-0.001	0.334	0.413	0.131
GB47029	uncharacterized protein LOC724558	7.860	0.639	-0.050	-0.153	0.020
GB42654	leucine carboxyl methyltransferase 1-like	7.829	0.261	0.034	0.160	-0.284
GB52636	cell growth regulator with RING finger domain protein 1-like isoform X2	7.762	0.280	-0.043	0.146	-0.246
725183	probable palmitoyltransferase ZDHHC24-like isoform X1	7.761	0.018	-0.268	0.291	-0.039
GB43882	alpha-mannosidase 2 isoform X3	7.673	0.433	-0.390	0.254	-0.083
GB51385	nephrin-like isoform 1	7.638	-0.322	0.244	0.187	0.038
GB52082	LOW QUALITY PROTEIN: sn1-specific diacylglycerol lipase alpha	7.610	0.099	0.044	0.235	0.036
GB52986	mitochondrial import inner membrane translocase subunit Tim23-like isoform X1	7.587	0.245	0.009	0.145	-0.006

(continued)

Gene	Name	k	am_fc	bt_fc	lf_fc	ln_fc
102653601	zinc finger protein 729-like	7.550	0.054	0.008	0.251	-0.335
GB46371	tyrosine-protein kinase Src64B-like isoform X3	7.532	-0.066	0.129	0.232	0.228
GB54032	methyltransferase-like protein 9-like isoform X2	7.483	0.372	0.199	0.077	-0.157
GB40928	tripartite motif-containing protein 2-like isoform X1	7.481	-0.190	-0.093	0.160	0.092
102656070	uncharacterized protein LOC102656070	7.386	0.077	-0.379	0.413	-0.164
GB44315	monocarboxylate transporter 9-like isoform X1	7.364	0.448	0.223	0.193	0.117
GB42377	protein giant-lens-like	7.279	0.069	-0.037	0.093	-0.003
GB44984	U5 small nuclear ribonucleoprotein 40 kDa protein-like isoform X1	7.153	0.504	0.063	0.152	-0.027
GB42487	calpain-C isoform X2	7.061	0.264	0.236	0.187	-0.053
GB44041	dachshund homolog 2-like isoform X3	6.950	0.245	-0.055	0.129	-0.099
GB44060	centrosomal protein of 104 kDa-like isoform X2	6.788	0.989	-0.101	0.222	0.054
GB46749	endochitinase-like isoform X1	6.699	0.680	0.181	0.108	-0.017
GB42326	glutamyl aminopeptidase-like isoform X3	6.665	-0.192	-0.227	-0.191	-0.073
GB40531	uncharacterized protein LOC100578051 isoform X3	6.581	-0.178	-0.096	0.241	-0.323
102655815	suppressor protein SRP40-like	6.410	0.852	1.383	0.820	-0.625
GB53146	uncharacterized protein LOC412149 isoform X1	6.402	-0.083	0.105	-0.186	0.062
GB46073	uncharacterized protein LOC551865 isoformX2	6.287	-0.017	0.226	-0.010	0.023
GB43015	myocardin-related transcription factor A-like isoform X6	6.152	-0.037	0.014	0.263	0.063
102654007	uncharacterized protein LOC102654007	5.894	0.242	-0.129	0.208	-0.015
102656939	histone-lysine N-methyltransferase SETMAR-like	5.866	0.150	0.237	1.062	0.600
GB41647	transcription factor Sox-7-like isoform X2	5.796	0.024	-0.280	1.467	-0.388
GB49810	RIB43A-like with coiled-coils protein 1-like	5.283	-0.475	-0.103	-0.159	-0.239
GB44616	EF-hand domain-containing family member C2-like isoform X1	5.223	0.236	0.045	0.070	-0.294
GB50734	uncharacterized protein LOC725625	4.956	0.572	-0.271	0.368	-0.026
GB53798	esterase E4-like	4.764	0.913	-1.190	0.257	-0.094
GB45427	Krueppel homologous protein 1	4.573	-0.017	0.040	0.453	-0.219
GB42800	protein takeout-like isoform X1	4.225	0.788	0.199	0.020	-0.125
GB46290	acetyl-coenzyme A synthetase-like	4.153	0.191	-0.163	0.425	-0.205
GB44976	ataxin-2 homolog isoform X3	4.150	0.047	-0.068	0.267	-0.184
GB40495	zinc finger protein 43-like isoform X1	3.868	0.527	-0.336	0.635	-0.017
GB40554	G1/S-specific cyclin-D2	3.866	0.136	0.094	0.199	-0.353
GB45382	intraflagellar transport protein 74 homolog	3.715	0.036	-0.125	0.245	-0.053
GB43604	uncharacterized protein LOC725033 isoform X4	3.676	0.345	-0.020	0.472	0.108
GB51657	uncharacterized protein LOC100578157	3.638	0.132	-0.534	0.783	-0.392
GB41844	ATP-binding cassette sub-family G member 5-like	3.373	0.766	0.129	0.808	-0.164
GB51376	serotonin receptor	3.060	1.690	0.484	0.148	0.610
GB17921	dopamine receptor 2	2.761	0.283	-0.285	-0.405	-0.083
GB45956	putative succinate dehydrogenase [ubiquinone] cytochrome b small subunit, mitochondrial-like	2.709	-0.203	-0.280	0.586	0.090
102655054	protein tyrosine phosphatase domain-containing protein 1-like isoform X2	2.504	0.850	0.375	0.681	0.128
GB50186	uncharacterized protein LOC100577394 isoform X3	2.177	0.031	0.299	-0.708	0.209
GB46310	cuticular protein 17 precursor	1.920	0.296	0.432	0.372	-0.158

Supplementary Table 33: List of all the genes in Module 8, ranked by their within-module connectivity, k. The latter four columns give the Log_2 fold-change in expression in response to queen pheromone in each of the four species.

Gene	Name	k	am_fc	bt_fc	lf_fc	ln_fc
GB41139	NADH dehydrogenase [ubiquinone] 1 alpha subcomplex subunit 8	4.401	-0.119	-0.106	0.029	0.158
GB54940	growth hormone-inducible transmembrane protein-like isoform X5	4.339	0.016	0.068	0.074	0.053
GB46440	NADH dehydrogenase [ubiquinone] 1 beta subcomplex subunit 11, mitochondrial-like	4.100	-0.143	-0.089	0.110	0.166
GB54961	uncharacterized protein LOC725712 isoform X3	3.922	-0.104	-0.087	0.124	0.135
GB54596	cytochrome b-c1 complex subunit 7-like	3.922	-0.169	-0.326	-0.009	0.178
GB49313	voltage-dependent anion-selective channel	3.756	0.121	-0.035	0.065	0.132
GB55708	NADH dehydrogenase [ubiquinone] 1 beta subcomplex subunit 8, mitochondrial-like	3.733	-0.080	-0.165	0.090	0.159
GB42422	ADP/ATP translocase	3.594	-0.037	-0.283	0.054	0.154
GB46369	cytochrome c1, heme protein, mitochondrial isoform X3	3.588	-0.054	-0.088	0.346	0.171
GB42929	cytochrome c oxidase subunit 4 isoform 1, mitochondrial isoform X1	3.586	0.009	-0.269	0.130	0.144
GB41741	NADH dehydrogenase [ubiquinone] 1 beta subcomplex subunit 5, mitochondrial-like isoform X2	3.574	-0.085	-0.171	0.133	0.183
GB43629	NADH dehydrogenase [ubiquinone] 1 beta subcomplex subunit 10-like	3.519	0.096	-0.164	0.158	0.264
GB41028	ATP synthase subunit alpha, mitochondrial isoform 1	3.507	-0.055	-0.093	0.236	0.375
GB52753	succinate dehydrogenase cytochrome b560 subunit, mitochondrial	3.485	0.081	-0.178	0.274	0.204
GB53749	cytochrome c oxidase subunit 6A1, mitochondrial	3.470	0.128	-0.295	0.118	0.180
GB48784	cytochrome c	3.459	-0.004	-0.398	0.275	0.779
GB46882	NADH dehydrogenase [ubiquinone] iron-sulfur protein 3, mitochondrial	3.429	0.017	-0.023	0.140	0.599
GB55643	ATP synthase subunit O, mitochondrial	3.381	-0.122	-0.389	0.142	0.175
GB47679	putative ATP synthase subunit f, mitochondrial-like	3.371	0.083	-0.152	0.117	0.106
GB52736	ATP synthase subunit beta, mitochondrial isoform X1	3.356	-0.029	-0.003	0.297	-0.006
GB47500	mitochondrial-processing peptidase subunit beta-like	3.345	-0.188	-0.060	0.081	0.203
GB51086	ATP synthase subunit delta, mitochondrial isoform 3	3.341	-0.067	-0.075	0.146	0.270
GB41143	NADH dehydrogenase [ubiquinone] 1 alpha subcomplex subunit 9, mitochondrial	3.297	-0.301	-0.190	0.137	0.111
GB47886	NADH dehydrogenase [ubiquinone] 1 alpha subcomplex subunit 7-like	3.282	0.119	-0.142	0.225	0.180
GB51192	cytochrome b-c1 complex subunit Rieske, mitochondrial	3.207	0.037	-0.104	0.184	0.627
GB54687	phosphate carrier protein, mitochondrial-like isoform 1	3.195	0.044	-0.288	0.077	0.122
GB49306	ATP synthase subunit gamma, mitochondrial isoform X1	3.135	-0.249	-0.110	-0.140	-0.178
GB43704	adenylosuccinate synthetase-like	3.083	0.051	-0.095	0.156	0.161
GB45153	NADH dehydrogenase [ubiquinone] flavoprotein 1, mitochondrial isoform X2	3.049	-0.054	-0.029	0.325	0.111
GB45099	superoxide dismutase 2, mitochondrial	3.036	-0.139	-0.223	0.075	0.154
GB44608	NADH dehydrogenase [ubiquinone] 1 beta subcomplex subunit 3-like isoform 1	2.711	0.175	0.062	0.107	0.139
GB50918	NADH dehydrogenase [ubiquinone] iron-sulfur protein 6, mitochondrial	2.692	-0.245	-0.147	0.061	0.037
GB50554	negative elongation factor A-like	2.586	0.235	0.053	0.069	0.163
GB42871	putative ATP-dependent Clp protease proteolytic subunit, mitochondrial-like isoform X4	2.225	-0.014	-0.048	0.073	0.148
GB43248	alpha glucosidase 2 precursor	2.147	0.132	0.132	-0.258	-0.153
GB50268	NADH dehydrogenase [ubiquinone] iron-sulfur protein 8, mitochondrial isoform 2	2.087	-0.197	-0.074	0.126	0.215
GB45731	NADH dehydrogenase [ubiquinone] 1 alpha subcomplex subunit 5	2.077	0.042	-0.134	0.105	0.187
725253	protein QIL1-like isoform 2	2.004	0.000	0.114	-0.152	0.190
GB50946	uncharacterized protein LOC724626	0.742	-0.198	0.111	0.222	0.153
GB42823	uncharacterized protein LOC100577440 isoform X3	0.715	0.144	0.302	0.042	0.267

Supplementary Table 34: List of all the genes in Module 9, ranked by their within-module connectivity, k. The latter four columns give the Log_2 fold-change in expression in response to queen pheromone in each of the four species.

Gene	Name	k	am_fc	bt_fc	lf_fc	ln_fc
GB49598	RNA-binding protein Rsf1	3.428	0.306	-0.003	0.110	0.085
GB49355	uncharacterized protein LOC100576266 isoform X2	3.262	0.309	0.057	-0.156	0.132
GB51008	metaxin-2-like isoform 2	3.200	0.444	-0.147	0.042	0.145
GB52735	DAZ-associated protein 2-like isoform X2	2.972	0.287	0.023	0.062	-0.175
GB55970	proliferating cell nuclear antigen	2.813	0.378	0.235	0.078	0.078
551833	PAXIP1-associated glutamate-rich protein 1-like isoform 2	2.757	0.496	0.093	0.059	0.249
GB43092	cyclin-dependent kinase 5	2.596	0.384	0.022	0.095	0.143
GB55381	centrosomal protein of 97 kDa isoform X2	2.558	0.296	-0.055	0.187	0.136
GB43232	transmembrane protein 222-like isoform 1	2.340	0.586	0.046	0.193	0.242
GB50724	peptidyl-tRNA hydrolase 2, mitochondrial-like isoform 1	2.248	0.709	-0.076	0.065	0.117
GB51226	tyrosine-protein kinase CSK isoform X4	2.224	0.518	0.004	0.202	0.157
GB56003	methyltransferase-like protein 14 homolog	2.217	0.335	0.148	0.128	0.104
GB48128	DNA-directed RNA polymerase III subunit RPC8-like isoform 1	2.126	0.793	0.181	-0.200	0.194
GB42319	uncharacterized protein LOC409105 isoform 1	2.108	0.761	-0.179	0.094	0.306
GB45649	adenosine 3'-phospho 5'-phosphosulfate transporter 1	2.038	0.533	-0.062	0.149	0.211
GB43086	uncharacterized protein LOC726486	1.969	0.668	0.055	0.124	0.107
GB45657	cdc42 homolog isoform X2	1.944	0.413	0.165	0.173	0.088
GB53270	UPF0428 protein CXorf56 homolog isoformX2	1.944	-0.005	0.090	0.096	0.156
GB48852	heterogeneous nuclear ribonucleoprotein H-like isoform X1	1.917	0.394	-0.024	0.187	0.163
GB45560	2-aminoethanethiol dioxygenase-like isoform X2	1.893	0.565	0.086	0.204	0.196
GB53957	U6 snRNA-associated Sm-like protein LSm1-like	1.873	1.276	0.149	0.065	0.103
GB55241	myosin-9-like isoform X2	1.858	0.383	0.027	0.161	0.336
GB42726	lysosomal protein NCU-G1-A-like	1.854	0.196	-0.116	0.083	0.184
GB52929	soluble guanylyl cyclase alpha 1 subunit	1.820	0.863	0.068	0.247	0.199
GB55098	progestin and adipoQ receptor family member 4-like isoform X3	1.807	1.098	0.096	0.284	0.340
GB50885	uncharacterized protein LOC409648	1.801	0.522	-0.141	0.108	-0.092
GB54279	cleavage and polyadenylation specificity factor subunit 4	1.770	0.312	0.355	1.658	0.252
GB45810	locomotion-related protein Hikaru genki isoform X4	1.746	0.427	-0.050	0.190	0.130
GB54147	loss of heterozygosity 12 chromosomal region 1 protein homolog	1.732	0.442	-0.052	0.112	0.081
GB48175	probable cytochrome P450 305a1	1.716	1.579	0.096	0.067	0.222
GB50090	adenosine deaminase acting on RNA	1.702	0.285	-0.020	0.268	0.316
GB55831	aromatic-L-amino-acid decarboxylase isoform X2	1.640	0.746	0.738	0.093	0.286
GB52236	leucine-rich repeat-containing protein C10orf11 homolog isoform X1	1.604	0.235	0.077	0.024	0.133
GB44143	oxidative stress-induced growth inhibitor 1-like isoform X1	1.578	0.407	0.048	0.090	0.051
GB42224	leucine-rich repeat and calponin homology domain-containing protein 1-like isoform X2	1.575	0.442	-0.069	1.232	0.299
GB46734	mitochondrial import inner membrane translocase subunit TIM14-like isoform X3	1.572	0.117	0.013	0.202	0.273
GB43817	atrial natriuretic peptide receptor 1-like	1.513	0.433	0.124	0.180	0.089
GB50722	phospholipase A1 member A-like	0.753	1.900	0.329	-0.074	0.550
