# Supplementary material for: Comparative transcriptomics of social insect queen pheromones
Source: Nat Commun. 2019 Apr 8;10:1593. doi: 10.1038/s41467-019-09567-2 (PMC6453924; doi:10.1038/s41467-019-09567-2)
Supplement: Supplementary file 3 — Reporting Summary [file 41467_2019_9567_MOESM3_ESM.pdf]

nature research

Corresponding author(s): Luke Holman

Last updated by author(s): Mar 6, 2019

Reporting Summary

Nature Research wishes to improve the reproducibility of the work that we publish. This form provides structure for consistency and transparency in reporting. For further information on Nature Research policies, see Authors & Referees and the Editorial Policy Checklist.

Statistics

For all statistical analyses, confirm that the following items are present in the figure legend, table legend, main text, or Methods section.

n/a	Confirmed	
☐	☒	The exact sample size (*n*) for each experimental group/condition, given as a discrete number and unit of measurement
☐	☒	A statement on whether measurements were taken from distinct samples or whether the same sample was measured repeatedly
☐	☒	The statistical test(s) used AND whether they are one- or two-sided<br>*Only common tests should be described solely by name; describe more complex techniques in the Methods section.*
☐	☒	A description of all covariates tested
☐	☒	A description of any assumptions or corrections, such as tests of normality and adjustment for multiple comparisons
☐	☒	A full description of the statistical parameters including central tendency (e.g. means) or other basic estimates (e.g. regression coefficient) AND variation (e.g. standard deviation) or associated estimates of uncertainty (e.g. confidence intervals)
☐	☒	For null hypothesis testing, the test statistic (e.g. *F*, *t*, *r*) with confidence intervals, effect sizes, degrees of freedom and *P* value noted<br>*Give P values as exact values whenever suitable.*
☐	☒	For Bayesian analysis, information on the choice of priors and Markov chain Monte Carlo settings
☐	☒	For hierarchical and complex designs, identification of the appropriate level for tests and full reporting of outcomes
☐	☒	Estimates of effect sizes (e.g. Cohen's *d*, Pearson's *r*), indicating how they were calculated

Our web collection on statistics for biologists contains articles on many of the points above.

Software and code

Policy information about availability of computer code

Data collection	Bash, Python, and R scripts used to reproduce our bioinformatics pipeline and data analysis are archived on Github (https://github.com/mikheyev/queen-pheromone). A report generated with R Markdown, which was used to produce all our results, figures, and tables, can be viewed online at https://mikheyev.github.io/queen-pheromone.
Data analysis	R and Python

For manuscripts utilizing custom algorithms or software that are central to the research but not yet described in published literature, software must be made available to editors/reviewers. We strongly encourage code deposition in a community repository (e.g. GitHub). See the Nature Research guidelines for submitting code & software for further information.

Data

Policy information about availability of data

All manuscripts must include a data availability statement. This statement should provide the following information, where applicable:

- Accession codes, unique identifiers, or web links for publicly available datasets
- A list of figures that have associated raw data
- A description of any restrictions on data availability

The raw sequencing data have been deposited at NCBI (BioSample ascensions: SAMD00106316 to -58 [https://www.ncbi.nlm.nih.gov/bioproject?LinkName=biosample_bioproject&from_uid=10236766]). All the remaining data used to generate the results in this paper are available at https://github.com/mikheyev/queen-pheromone.

Field-specific reporting

Please select the one below that is the best fit for your research. If you are not sure, read the appropriate sections before making your selection.

☐ Life sciences ☐ Behavioural & social sciences ☒ Ecological, evolutionary & environmental sciences

For a reference copy of the document with all sections, see nature.com/documents/nr-reporting-summary-flat.pdf

Ecological, evolutionary & environmental sciences study design

All studies must disclose on these points even when the disclosure is negative.

Study description	We exposed insects from each of 4 species to a queen pheromone treatment or a control, then collected their mRNA for analysis using RNA sequencing.
Research sample	We used wild-collected ants from Tvarminne, Finland; domestic honey bees from an apiary in the UK, and sourced bumblebees from BioBest, a bee-breeding company in Belgium.
Sampling strategy	Sample sizes were chosen based on our budget (RNA sequencing is very expensive), and to be larger than earlier similar studies.
Data collection	The RNA seq raw data were collected by technicians at Edinburgh Genomics, who passed the data to LH and ASM, who worked together to perform the bioinformatic analyses described in the paper.
Timing and spatial scale	The queen pheromone applications were performed over a number of days that was chosen based on earlier work, e.g. Grozinger 2003 PNAS. We selected the shortest time at which we expected the pheromones would have affected gene expression.
Data exclusions	No data were excluded, except the 4 samples whose RNA was not sequenced properly. This is discussed in the paper and shown in Figures S1 and S2.
Reproducibility	Our entire bioinformatics and statistical analysis is archived and meticulously documented to ensure it is transparent and reproducible from the raw data.
Randomization	We randomly assigned individuals to our 2 treatment groups.
Blinding	The pheromone experiments were conducted blind, and the RNA extraction and sequencing was also blind to treatment.

Did the study involve field work? ☐ Yes ☒ No

Reporting for specific materials, systems and methods

We require information from authors about some types of materials, experimental systems and methods used in many studies. Here, indicate whether each material, system or method listed is relevant to your study. If you are not sure if a list item applies to your research, read the appropriate section before selecting a response.

Materials & experimental systems

n/a	Involved in the study	
☒	☐	Antibodies
☒	☐	Eukaryotic cell lines
☒	☐	Palaeontology
☐	☒	Animals and other organisms
☒	☐	Human research participants
☒	☐	Clinical data

Methods

n/a	Involved in the study	
☒	☐	ChIP-seq
☒	☐	Flow cytometry
☒	☐	MRI-based neuroimaging

Animals and other organisms

Policy information about studies involving animals; ARRIVE guidelines recommended for reporting animal research

Laboratory animals	*For laboratory animals, report species, strain, sex and age OR state that the study did not involve laboratory animals.*
Wild animals	Apis mellifera, Bombus terrestris, Lasius niger, Lasius flavus. 3-8 colonies of each species.
Field-collected samples	*For laboratory work with field-collected samples, describe all relevant parameters such as housing, maintenance, temperature, photoperiod and end-of-experiment protocol OR state that the study did not involve samples collected from the field.*
Ethics oversight	None - ethical oversight is not required for studies of insects

Note that full information on the approval of the study protocol must also be provided in the manuscript.
